# Global, regional, and national burdens of hip osteoarthritis from 1990 to 2019: estimates from the 2019 Global Burden of Disease Study

**DOI:** 10.1186/s13075-021-02705-6

**Published:** 2022-01-03

**Authors:** Ming Fu, Hongming Zhou, Yushi Li, Hai Jin, Xiqing Liu

**Affiliations:** 1 Department of Orthopedics and Surgery, Heilongjiang Provincial Hospital, Harbin Institute of Technology, Harbin, Heilongjiang China; 2 Department of Bone Surgery, Linyi City Central Hospital, Linyi, Shandong China; 3 Department of Hand Surgery, The Fifth Hospital of Harbin, Harbin, Heilongjiang China

**Keywords:** Hip osteoarthritis, Age-standardized rates, Incidence, Disability-adjusted life years

## Abstract

**Background:**

Hip osteoarthritis is a common disabling condition of the hip joint and is associated with a substantial health burden. We assessed the epidemiological patterns of hip osteoarthritis from 1990 to 2019 by sex, age, and socio-demographic index (SDI).

**Methods:**

Age-standardized rates (ASRs) were obtained for the incidence and disability-adjusted life years (DALYs) of hip osteoarthritis from 1990 to 2019 for 21 regions, encompassing a total of 204 countries and territories. The estimated annual percentage changes (EAPCs) of ASRs were calculated to evaluate the trends in the incidence and DALYs of hip osteoarthritis over these 30 years.

**Results:**

Globally, from 1990 to 2019, the age-standardized incidence rate (ASIR) of hip osteoarthritis increased from 17.02 per 100,000 persons to 18.70 per 100,000 persons, with an upward trend in the EAPC of 0.32 (0.29–0.34), whereas the age-standardized DALY rate increased from 11.54 per 100,000 persons to 12.57 per 100,000 persons, with an EAPC of 0.29 (0.27–0.32). In 2019, the EAPCs of the ASIR and age-standardized DALY rate of hip osteoarthritis were positively associated with the SDI of hip osteoarthritis. In 1990 and 2019, the incidence of hip osteoarthritis was unimodally distributed across different age groups, with a peak incidence in the 60–64-year-old age group, whereas the DALYs increased with age.

**Conclusions:**

The incidence and DALYs of hip osteoarthritis have been increasing globally. The EAPCs of the ASIR and age-standardized DALY rate were particularly significant in developed regions and varied across nations and regions, indicating the urgent need for governments and medical institutions to increase the awareness regarding risk factors, consequences of hip osteoarthritis.

**Supplementary Information:**

The online version contains supplementary material available at 10.1186/s13075-021-02705-6.

## Background

Osteoarthritis is a common disabling condition that involves joint pain and stiffness caused by the gradual erosion of cartilage [1] and is associated with a substantial health burden. Hip osteoarthritis is a common form of osteoarthritis and is a major cause of restricted locomotor activity and functional disability that may progress to the point where joint replacement is unavoidable [2, 3]. In recent years, the burden of osteoarthritis by region and country has been reported in several review papers [4–6]. One study [5] reported the global burden of osteoarthritis (knee and hip) for 1990–2017 using the World Health Organization Burden of Diseases Database, but it did not especially focus on hip osteoarthritis or on the association of hip osteoarthritis burden with country, region, sex, age, or social development index (SDI).

The Global Burden of Disease Study (GBD) 2019 [7] is a multinational collaborative research study that estimates the burden of 354 human diseases and injuries, including hip osteoarthritis, in 204 countries and territories worldwide, and provides a public dataset for use in investigations of the trends in the distribution of hip osteoarthritis. To assist policymakers to allocate resources and formulate relevant policies for this condition, we conducted various subgroup analyses (by region, SDI, age, and sex) of GBD 2019 data to assess the burden and variations in the global distribution of hip osteoarthritis.

## Methods

Osteoarthritis is the most common form of arthritis, involving chronic inflammation, breakdown, and structural alterations of the joint. The reference case definition is symptomatic osteoarthritis of the hip radiologically confirmed as Kellgren-Lawrence grade 2–4 [5, 8]. Grade 2 symptomatic OA involves one defined osteophyte in hip and pain for at least 1 month out of the past 12 months. Grade 3–4 symptomatic OA includes osteophytes and joint space narrowing in hip, with grade 4 also including deformity, and pain for at least 1 month out of the past 12 months [8, 9]. Data on the incidence and DALYs of hip osteoarthritis from 1990 to 2019 and the corresponding age-standardized rates (ASRs) were obtained from the Global Health Data Exchange (GHDx) query tool (http://ghdx.healthdata.org/gbd-results-tool). The GHDx is an ongoing global collaboration that collects all available epidemiological data (mainly comprising systematic reviews of published studies, data from government and international organization websites, published reports, primary data (e.g., from Demographic and Health Surveys), and datasets contributed by GBD collaborators) and provides a comparative assessment of disease burdens for 14 age groups, males, females, and for a combination of both sexes. The 204 countries and territories included in the GBD 2019 were grouped into 21 geographic regions and five SDI categories (low, low-middle, middle, high-middle, and high SDI). Nine countries and territories (the Cook Islands, Monaco, San Marino, Nauru, Niue, Palau, Saint Kitts and Nevis, Tokelau, and Tuvalu) were newly added to the GBD 2019 [7]. In addition, bias adjustments were calculated in GBD 2019 using MR-BRT for data that reported on hip osteoarthritis using alternative case-definitions, resulting in a decrease in years lived with disability due to hip osteoarthritis. Adjustments were made to studies reporting hip osteoarthritis identified by radiography alone, by self-reported physician diagnosis with pain, by self-reported physician diagnosis with no mention of pain, and USA claims data [7].

## Statistical analyses

ASRs of the incidence and DALYs of hip osteoarthritis were calculated per 100,000 population, as described in the previous study (GBD 2013) [10]. The ASR (per 100,000 population) was estimated by summing the products of age-specific rates (*a*_i_, where i denotes the *i*th age class) and the number of persons (or weight) (*w*_i_) in the same age subgroup *i* of the designated reference population, divided by the sum of standard population weights. DALYs were estimated by summing the years lived with disability and years of life lost. We also calculated the estimated annual percentage changes (EAPCs) of these parameters, to quantify the trends in the ASRs of hip osteoarthritis incidence and DALYs. The EAPC describes the trends within a specified time interval. The natural logarithm of an ASR is assumed to be linear along time, that is, *Y* = *α* + *βX* + *ε*, where *Y* refers to ln (ASR), *X* refers to the calendar year, and *ε* refers to the error term. Based on this formula, *β* represents positive or negative ASR trends [11]. The EAPC was calculated as EAPC = 100 × [exp(β) − 1]. Its 95% confidence intervals (CIs) could be obtained from the linear model. When the EAPC and lower CI limit are positive, the ASR shows an upward trend. Conversely, when the EAPC and upper CI limit are negative, the ASR shows a downward trend. In addition, to identify factors that may affect the EAPC, we evaluated the relationship between EAPC and the SDI in 2019 and ASRs in 1990. *ρ* represents Pearson’s correlation coefficient. The GBD 2019 complied with the Guidelines for Accurate and Transparent Health Estimates Reporting statement.

## Results

### Analysis of global incidence of hip osteoarthritis

From 1990 to 2019, the global incidence of hip osteoarthritis increased from 0.74 million to 1.58 million, reflecting a total increase of 115.40%. This was consistent with the increase in the age-standardized incidence rate (ASIR) from 17.02 per 100,000 persons in 1990 to 18.70 per 100,000 persons in 2019, reflecting an upward EAPC trend of 0.32 (0.29–0.34) (Table 1). In addition, the incidence of hip osteoarthritis in men was 1.93-fold higher than that in women, which was inconsistent with the trend of ASIR in the two sexes (male to female ratio = 0.96).

**Table 1:** The ASIR of hip osteoarthritis in 1990 and 2019 and its temporal trends

Characteristics	1990		2019		1990–2019	
	ASIR (per 100000)		ASIR (per 100000)		EAPC	***P***
	No. (95%UI)	Male/female ratio	No. (95%UI)	Male/female ratio	No. (95%CI)	
Overall	17.02 (12.67–22.04)	0.94	18.70 (13.98–24.19)	0.96	0.32 (0.29–0.34)	< 0.001
Sex						
Male	16.50 (12.33–21.35)	NA	18.35 (13.73–23.77)	NA	0.31 (0.28–0.33)	< 0.001
Female	17.48 (12.96–22.67)	NA	19.03 (14.27–24.28)	NA	0.33 (0.30–0.37)	< 0.001
**Region**						
East Asia	8.34 (6.13–10.84)	1.15	11.39 (8.40–14.82)	1.18	1.22 (1.11–1.33)	< 0.001
Southeast Asia	10.50 (7.78–13.66)	1.14	12.51 (9.29–16.20)	1.21	0.65 (0.62–0.67)	< 0.001
Oceania	12.40 (9.03–16.02)	1.19	13.60 (9.99–17.61)	1.15	0.20 (0.09–0.31)	< 0.001
Central Asia	18.58 (13.81–24.09)	1.18	20.90 (15.56–27.06)	1.18	0.36 (0.33–0.40)	< 0.001
Central Europe	20.22 (15.03–26.06)	1.21	23.28 (17.31–30.11)	1.14	0.48 (0.47–0.49)	< 0.001
Eastern Europe	20.03 (14.84–25.89)	1.51	23.10 (17.24–29.82)	1.42	0.49 (0.47–0.51)	< 0.001
High–income Asia Pacific	21.65 (16.00–28.02)	1.03	23.72 (17.57–30.75)	0.97	0.30 (0.23–0.36)	< 0.001
Australasia	29.20 (21.66–37.86)	0.93	38.74 (28.74–49.96)	0.95	0.98 (0.90–1.07)	< 0.001
Western Europe	33.44 (24.94–43.01)	0.87	38.36 (28.41–49.67)	0.85	0.51 (0.44–0.57)	< 0.001
Southern Latin America	26.19 (19.21–33.70)	0.91	34.70 (25.79–44.66)	0.97	0.96 (0.86–1.07)	< 0.001
High-income North America	40.04 (29.84–51.77)	0.86	50.23 (39.07–62.80)	0.85	0.59 (0.48–0.69)	< 0.001
Caribbean	14.22 (10.59–18.29)	1.11	16.66 (12.30–21.59)	1.16	0.62 (0.57–0.67)	< 0.001
Andean Latin America	13.38 (9.84–17.36)	1.07	16.52 (12.22–21.49)	1.16	0.73 (0.69–0.76)	< 0.001
Central Latin America	13.13 (9.74–17.07)	1.19	15.12 (11.18–19.66)	1.23	0.20 (0.10–0.30)	< 0.001
Tropical Latin America	14.14 (10.49–18.32)	1.14	17.14 (12.76–22.16)	1.22	0.70 (0.68–0.73)	< 0.001
North Africa and Middle East	11.14 (8.32–14.39)	1.18	13.79 (10.31–17.65)	1.14	0.65 (0.60–0.70)	< 0.001
South Asia	9.74 (7.25–12.62)	0.68	12.36 (9.26–16.02)	0.69	0.96 (0.81–1.11)	< 0.001
Central Sub-Saharan Africa	14.74 (10.86–18.98)	1.09	15.89 (11.71–20.59)	1.15	0.19 (0.14–0.23)	< 0.001
Eastern Sub-Saharan Africa	14.53 (10.77–18.84)	1.11	16.44 (12.16–21.34)	1.25	0.46 (0.43–0.48)	< 0.001
Southern Sub-Saharan Africa	20.17 (14.85–26.18)	1.73	23.08 (16.97–30.23)	1.94	0.48 (0.47–0.50)	< 0.001
Western Sub-Saharan Africa	13.96 (10.33–18.11)	1.10	14.78 (10.91–19.17)	1.11	0.02 (–0.11–0.15)	0.724
**Sociodemographic index**						
High SDI	31.34 (23.35–40.44)	0.89	36.94 (27.85–46.83)	0.86	0.50 (0.47–0.53)	< 0.001
High-middle SDI	17.35 (12.89–22.56)	1.09	18.53 (13.85–24.07)	1.07	0.25 (0.22–0.29)	< 0.001
Middle SDI	10.43 (7.76–13.51)	1.10	12.70 (9.44–16.41)	1.03	0.87 (0.78–0.95)	< 0.001
Low-middle SDI	10.23 (7.62–13.25)	0.85	12.61 (9.43–16.29)	0.85	0.85 (0.74–0.95)	< 0.001
Low SDI	11.95 (8.88–15.41)	0.94	12.80 (9.55–16.52)	0.88	0.46 (0.37–0.54)	< 0.001

*Abbreviations*: ASIR, age-standardized incidence rate; EAPC, estimated annual percentage change; NA, not available; UI, uncertainty interval

**Fig. 1:**
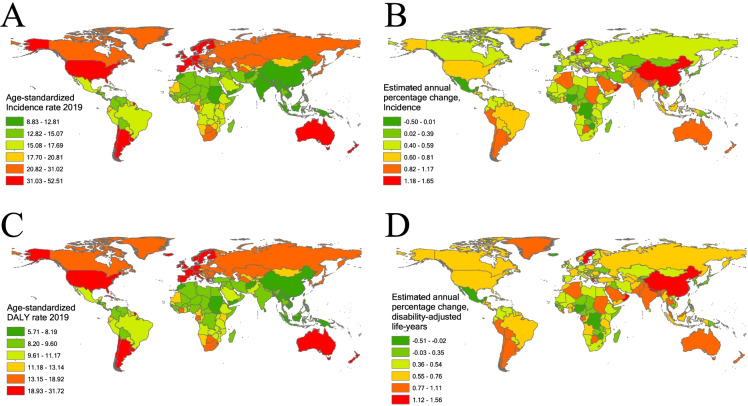
The global ASIR and age-standardized DALY rate (per 100,000) of hip osteoarthritis in 204 countries. **A** The ASIR of hip osteoarthritis in 2019. **B** The EAPC of hip osteoarthritis ASIR from 1990 to 2019. **C** The age-standardized DALY rate of hip osteoarthritis in 2019. **D** The EAPC of hip osteoarthritis age-standardized DALY rate from 1990 to 2019. Countries with an extreme number of cases/evolution were annotated. ASIR, age-standardized incidence rate; EAPC, estimated annual percentage change. DALY, disability-adjusted life years

In 2019, a higher incidence of hip osteoarthritis was observed in the USA, China, and India, whereas a lower incidence was observed in Tokelau, Niue, and Nauru (Supplementary Table 1). In 2019, higher ASIRs of hip osteoarthritis were observed in the USA, Iceland, and the UK, whereas lower ASIRs were observed in the Democratic People’s Republic of Korea, Yemen, and Timor-Leste (Fig. 1A and Supplementary Table 2–5). From 1990 to 2019, ASIRs of hip osteoarthritis increased in 194 countries and decreased in three countries (Denmark, Iceland, and Nigeria) (Fig. 1B and S Table 3). The highest EAPC in the ASIR was in Sweden, and the lowest was in Denmark.

In 2019, high-income North America had the highest incidence of hip osteoarthritis, whereas Oceania had the lowest. High-income North America also had the highest ASIR of hip osteoarthritis, whereas East Asia had the lowest (Table 1 and Supplementary Table 2). Furthermore, the EAPC of the ASIR increased in 20 regions, except in Western Sub-Saharan Africa, which showed no significant change (*P* = 0.724) (Table 1). The highest EAPC was in East Asia, and the lowest was in Central Sub-Saharan Africa (Table 1).

**Fig. 2:**
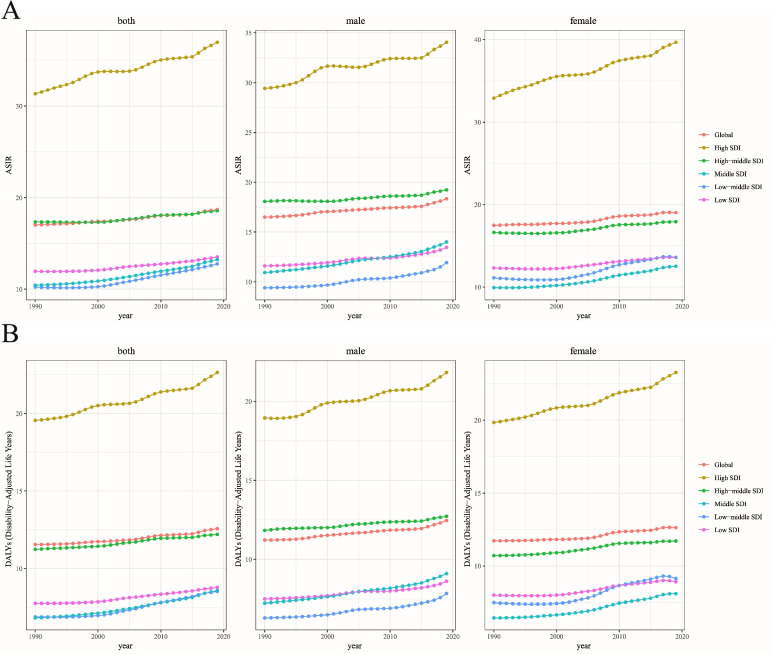
The change trends of age standardized rate among different SDI quintiles and gender from 1990 to 2019. **A** ASIR. **B** Age-standardized DALY rate. ASIR, age-standardized incidence rate; DALY, disability-adjusted life year

**Fig. 3:**
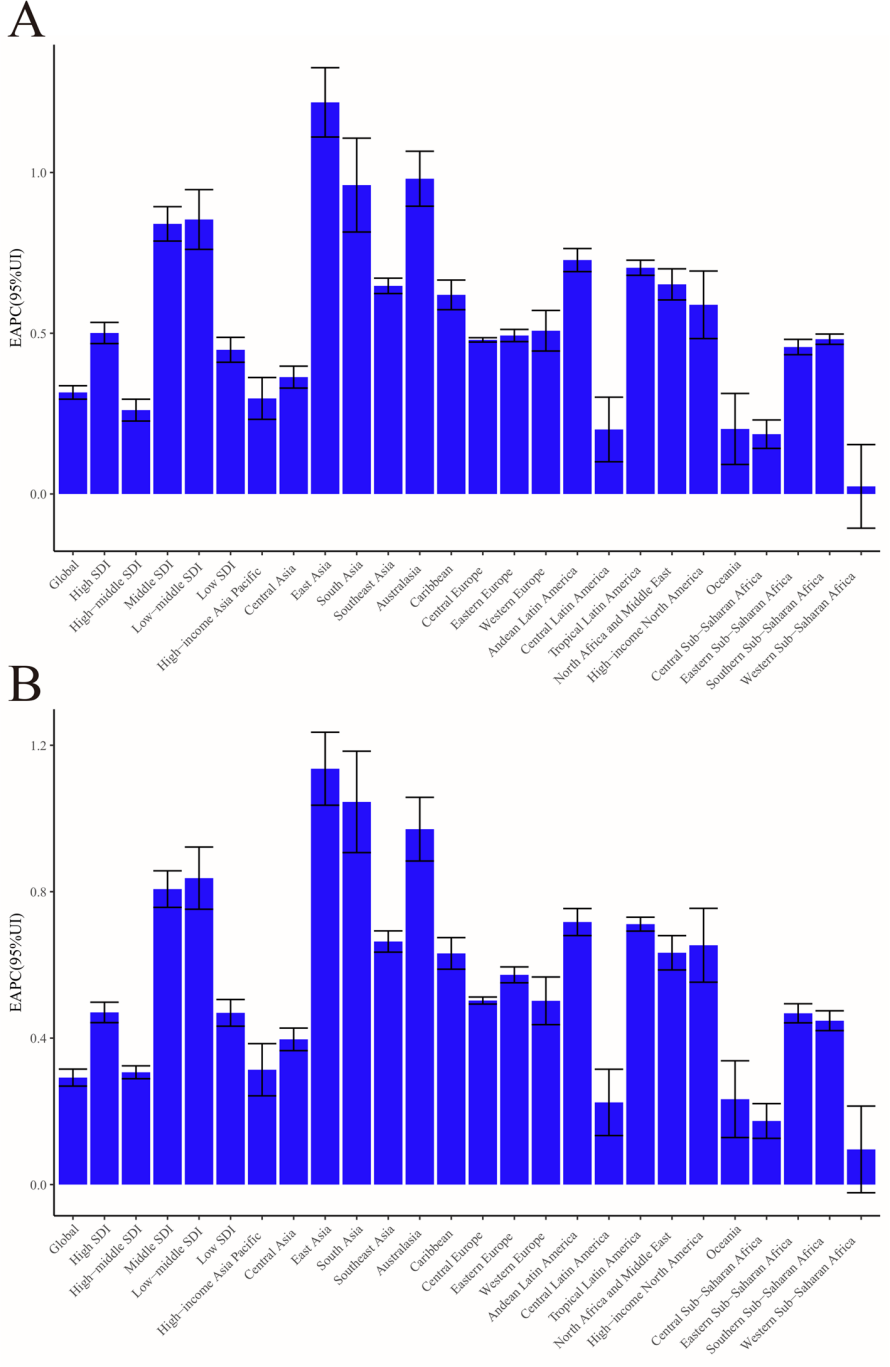
The EAPC of hip osteoarthritis ASIR and age-standardized DALY rate from 1990 to 2019, by region. **A** The EAPC of ASIR. **B** The EAPC of age-standardized DALY rate. ASIR: age standardized incidence rate; EAPC, estimated annual percentage change; DALY, disability-adjusted life year

**Fig. 4:**
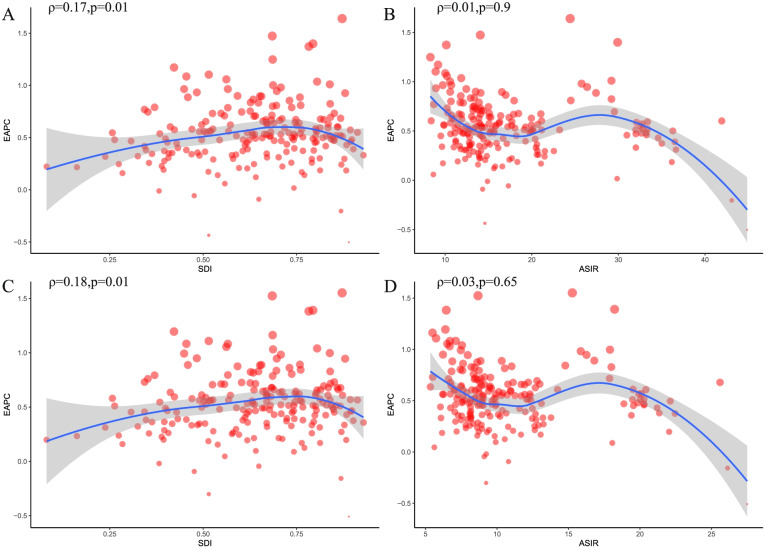
The correlation between EAPC and hip osteoarthritis ASR in 1990 as well as SDI in 2019. **A** EAPC of ASIR and SDI in 2019. **B** EAPC of ASIR and ASIR in 1990. **C** EAPC of age-standardized DALY rate and SDI in 2019. **D** EAPC of age-standardized DALY rate and age-standardized DALY rate in 1990; The smooth curve was fitted by Loess regression. The circles represent countries that were available on SDI data. The size of circle is increased with the cases of hip osteoarthritis. The ρ indices and *P* values presented were derived from Pearson correlation analysis. ASIR, age standardized incidence rate; EAPC, estimated annual percentage change; SDI, socio-demographic index; DALY, disability-adjusted life year

From 1990 to 2019, both the incidence and ASIRs of hip osteoarthritis increased in all five SDI quintiles (Table 1). The specific trends in the ASIRs over these 30 years are presented in Fig. 2A. The middle SDI quantile showed the highest increase in the incidence of hip osteoarthritis, whereas the high-middle SDI quintile showed the lowest increase. The middle SDI quantile showed the highest increase in the ASIR of hip osteoarthritis, whereas the high-middle SDI quintile showed the lowest increase (Table 1 and Fig. 3). In addition, the EAPC was positively associated with the SDI in 2019 (*ρ* = 0.17, *P* = 0.01) (Fig. 4A) but showed no correlation with the ASIR in 1990 (*ρ* = 0.01, *P* = 0.9) (Fig. 4B).

### Analysis of hip osteoarthritis DALYs worldwide

From 1990 to 2019, the DALYs of hip osteoarthritis increased from 0.46 million to 1.04 million, reflecting a total increase of 126.97%. This was consistent with the increase in the age-standardized DALY rate from 11.54 per 100,000 persons to 12.57 per 100,000 persons, reflecting an upward EAPC trend of 0.29 (0.27–0.32) (Table 1). In addition, the increase in the DALYs of hip osteoarthritis in women was 1.13-fold higher than that in men, consistent with the trend in the age-standardized DALY rate in the two sexes (female to male ratio = 1.01).

In 2019, higher DALYs of hip osteoarthritis were observed in the USA, China, and India, whereas lower DALYs were observed in Tokelau, Niue, and Nauru (Supplementary Table 1). In 2019, higher age-standardized DALY rates of hip osteoarthritis were observed in the USA, Iceland, and the UK, whereas lower rates were observed in the Democratic People’s Republic of Korea, Yemen, and Timor-Leste (Fig. 1C and Supplementary Table 6–8). From 1990 to 2019, the age-standardized DALY rates of hip osteoarthritis increased in 196 countries and decreased in four countries (Denmark, Iceland, Nigeria, and Zimbabwe). The highest EAPC in the age-standardized DALY rate was in Equatorial Guinea, and the lowest was in Denmark (Fig. 1D).

In 2019, Western Europe had the highest DALYs of hip osteoarthritis, whereas Oceania had the lowest (Supplementary Table 9). High-income North America had the highest age-standardized DALY rate of hip osteoarthritis, whereas East Asia had the lowest (Table 2 and Supplementary Table 10). In addition, the EAPC of the age-standardized DALY rate increased in 20 regions, except Western Sub-Saharan Africa, which showed no significant change (*P* = 0.108) (Table 2). The highest EAPC was in East Asia, and the lowest was in Central Sub-Saharan Africa (Table 2).

**Table 2:** The age-standardized DALY rate of hip osteoarthritis in 1990 and 2019 and its temporal trends

Characteristics	1990		2019		1990–2019	
	Age-standardized DALY rate (per 100000)		Age-standardized DALY rate (per 100000)		EAPC	*P*
	No. (95% UI)	Female/male ratio	No. (95% UI)	Female/male ratio	No. (95% CI)	
Global	11.54 (5.41–23.64)	0.96	12.57 (5.91–25.79)	0.99	0.29 (0.27–0.32)	< 0.001
Male	11.22 (5.27–23.11)	NA	12.46 (5.89–25.72)	NA	0.32 (0.29–0.34)	< 0.001
Female	11.73 (5.49–24.15)	NA	12.63 (5.91–25.84)	NA	0.30 (0.26–0.33)	< 0.001
**Region**						
East Asia	5.52 (2.55–11.30)	1.17	7.44 (3.43–15.44)	1.19	1.14 (1.04–1.24)	< 0.001
Southeast Asia	6.65 (3.11–13.48)	1.14	7.95 (3.67–16.34)	1.21	0.66 (0.63–0.69)	< 0.001
Oceania	7.82 (3.70–16.16)	1.20	8.63 (4.09–17.73)	1.16	0.23 (0.13–0.34)	< 0.001
Central Asia	11.53 (5.44–24.22)	1.18	13.09 (6.14–26.98)	1.19	0.40 (0.37–0.43)	< 0.001
Central Europe	12.47 (5.84–25.77)	1.21	14.43 (6.80–29.84)	1.14	0.50 (0.49–0.51)	< 0.001
Eastern Europe	12.05 (5.68–24.93)	1.51	14.15 (6.70–29.15)	1.43	0.57 (0.55–0.59)	< 0.001
High-income Asia Pacific	13.31 (6.26–27.30)	1.12	14.68 (6.91–30.43)	1.05	0.31 (0.24–0.38)	< 0.001
Australasia	17.92 (8.34–37.55)	1.00	23.72 (11.28–48.30)	1.02	0.97 (0.88–1.06)	< 0.001
Western Europe	20.42 (9.81–41.80)	0.93	23.41 (11.06–48.01)	0.92	0.50 (0.44–0.57)	< 0.001
Southern Latin America	16.10 (7.68–33.33)	0.99	21.32 (10.21–44.17)	1.05	0.97 (0.86–1.08)	< 0.001
High-income North America	24.51 (11.70–50.53)	0.93	30.34 (15.19–61.47)	0.92	0.65 (0.55–0.75)	< 0.001
Caribbean	9.07 (4.22–18.68)	1.12	10.66 (5.01–22.25)	1.16	0.63 (0.59–0.67)	< 0.001
Andean Latin America	8.48 (3.96–17.48)	1.08	10.45 (4.93–21.83)	1.17	0.72 (0.68–0.75)	< 0.001
Central Latin America	8.36 (3.88–17.40)	1.21	9.61 (4.54–19.84)	1.24	0.22 (0.13–0.31)	< 0.001
Tropical Latin America	8.87 (4.12–18.33)	1.14	10.84 (5.07–22.50)	1.23	0.71 (0.69–0.73)	< 0.001
North Africa and Middle East	7.18 (3.32–14.86)	1.20	8.85 (4.14–18.19)	1.16	0.63 (0.59–0.68)	< 0.001
South Asia	6.74 (3.22–13.63)	0.67	8.62 (4.11–17.49)	0.68	1.05 (0.91–1.18)	< 0.001
Central Sub-Saharan Africa	9.18 (4.31–19.06)	1.09	9.87 (4.74–20.37)	1.16	0.17 (0.13–0.22)	< 0.001
Eastern Sub-Saharan Africa	9.09 (4.25–18.82)	1.11	10.30 (4.79–21.46)	1.25	0.47 (0.44–0.49)	< 0.001
Southern Sub-Saharan Africa	12.53 (5.91–25.77)	1.76	14.19 (6.65–29.65)	1.99	0.45 (0.42–0.47)	< 0.001
Western Sub-Saharan Africa	8.84 (4.13–18.32)	1.10	9.49 (4.41–19.77)	1.11	0.10 (–0.02-0.21)	0.108
**Sociodemographic index**						
High SDI	19.56 (9.27–40.23)	0.96	22.65 (11.01–46.22)	0.94	0.47 (0.44–0.50)	< 0.001
High-middle SDI	11.23 (5.26–23.13)	1.10	12.20 (5.71–25.22)	1.09	0.31 (0.29–0.32)	< 0.001
Middle SDI	6.82 (3.18–13.96)	1.12	8.57 (3.99–17.61)	1.12	0.81 (0.76–0.86)	< 0.001
Low-middle SDI	6.89 (3.25–14.09)	0.84	8.52 (4.01–17.34)	0.86	0.84 (0.75–0.92)	< 0.001
Low SDI	7.75 (3.63–15.83)	0.94	8.77 (4.18–18.10)	0.97	0.47 (0.43–0.51)	< 0.001

*Abbreviations*: DALY, disability-adjusted life years; NA, not available; UI, uncertainty interval

From 1990 to 2019, both the DALYs and age-standardized DALY rate of hip osteoarthritis increased in all five SDI quintiles (Table 2). The specific trends in the age-standardized DALY rate over these 30 years are presented in Fig. 2B. The middle SDI quantile showed the highest increase in the DALYs of hip osteoarthritis, whereas the high-middle SDI quintile showed the lowest increase. Analogously, the low-middle SDI quantile showed the highest increase in the age-standardized DALY rate of hip osteoarthritis, whereas the high-middle SDI quintile showed the lowest increase (Table 2). In addition, the EAPC was positively associated with the SDI in 2019 (*ρ* = 0.18, *P* = 0.01) (Fig. 4C) but showed no correlation with the age-standardized DALY rate in 1990 (*ρ* =0.03, *P* = 0.65) (Fig. 4D).

### Age distribution of the incidence and DALYs of hip osteoarthritis

**Fig. 5:**
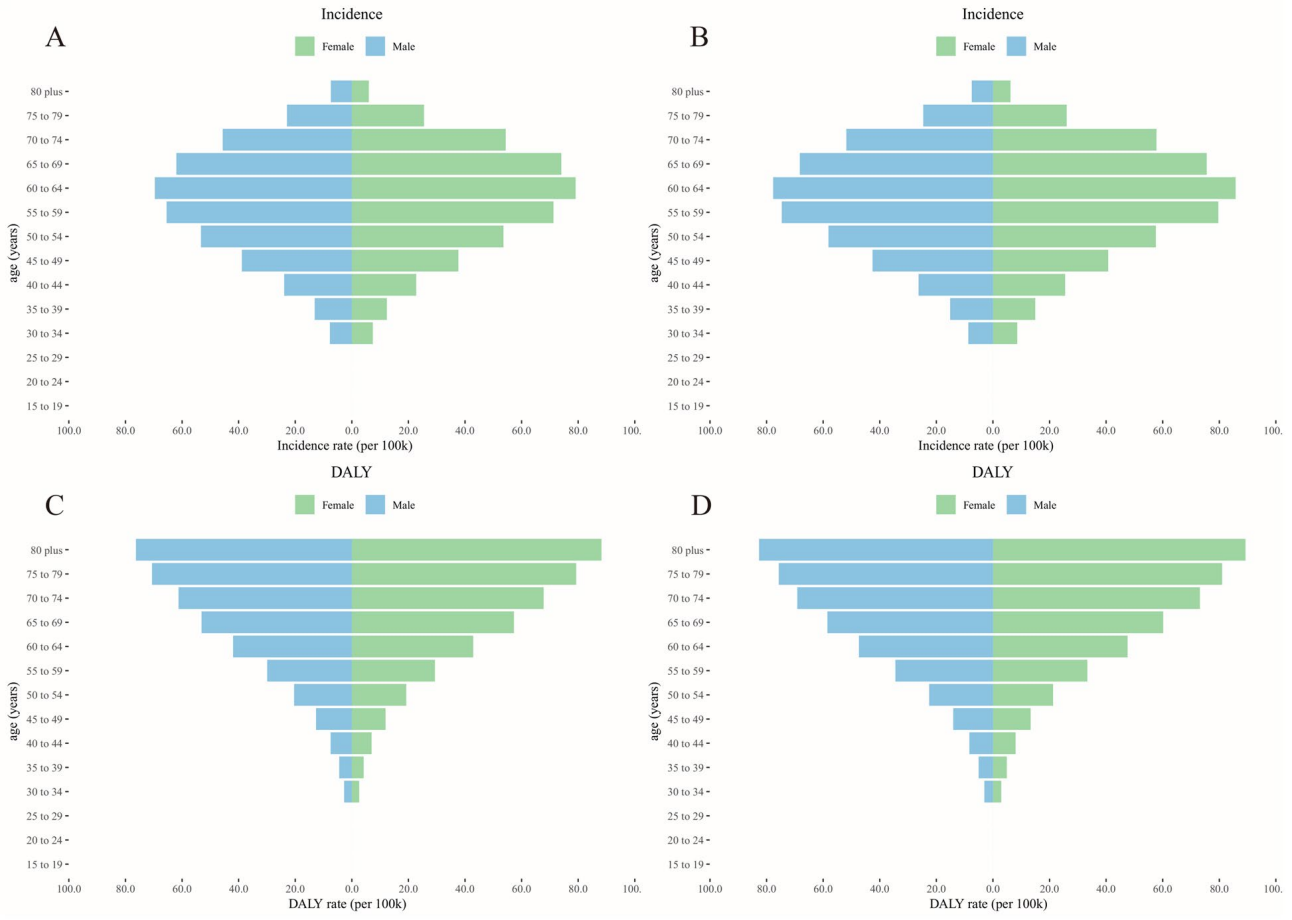
The incidence and DALY rate of hip osteoarthritis among gender and age. **A** Incidence rate in 1990. **B** Incidence rate in 2019. **C** DALY rate in 1990. **D** DALY rate in 2019; DALY, disability-adjusted life year

In 1990 and 2019, the incidence of hip osteoarthritis in both men and women was unimodally distributed across different age groups, with a peak in the 60–64-year-old age group. In 1990 and 2019, the DALYs increased with age in men and women. There was no notable difference in the incidence and DALYs of hip osteoarthritis between the two sexes or across age groups (Fig. 5 and S Tables 11–12).

## Discussion

This study, based on data from the GBD 2019, revealed that the incidence and DALYs of hip osteoarthritis, in addition to their corresponding ASRs, increased from 1990 to 2019, which is consistent with the findings from the GBD 2017 [5]. The incidence and DALYs of hip osteoarthritis have been increasing globally. The EAPCs of the ASIR and age-standardized DALY rate were particularly significant in developed regions and varied across nations and regions. Unlike the previous GBD 2017 study, this study included the EAPCs of the ASIR and age-standardized DALY rate and evaluated the correlation between the EAPC and ASIR of hip osteoarthritis in 1990 and that between the EAPC and SDI in 2019. In addition, this study also included data from nine additional countries and territories (Cook Islands, Monaco, San Marino, Nauru, Niue, Palau, Saint Kitts and Nevis, Tokelau, and Tuvalu) that were newly added to the GBD 2019 [7].

Of all the countries included in the GBD 2019, only Denmark, Iceland, and Nigeria experienced a decrease in the EAPCs of the ASIR and age-standardized DALY rate from 1990 to 2019. Accordingly, only Western Sub-Saharan Africa showed no increase in the ASIR and age-standardized DALY rate of hip osteoarthritis. It should be noted that the low level of basic social medical security in Western Sub-Saharan Africa could have resulted in the underestimation of hip osteoarthritis incidence and DALYs [12]. The burden of hip osteoarthritis was increasing over time. Thus, it is essential that prevention measures, management and treatment of OA are given priority [13, 14]. As excess weight is a risk factor for hip osteoarthritis, maintaining a healthy weight and performing regular exercise are crucial to prevent the development of hip osteoarthritis [15, 16]. Exercise not only helps to manage weight but also strengthens muscles surrounding the joints, which prevents wear and tear of the cartilage [16, 17]. Moreover, stretching exercises can help to improve hip flexibility and relieve joint stiffness and pain in patients with hip osteoarthritis [15]. In addition, several high-impact sports (e.g., weight-lifting, football, and long-distance running) [18] and employment in farming or the construction industry [19] also have been over moderately related with the increased risk of hip osteoarthritis. The association is mainly due to the presence of cam impingement, which can develop during sporting activities or heavy work [18]. The new surgical techniques that can reduce cam impingement should be assessed for preventing hip osteoarthritis.

Globally, the EAPCs of the ASIR and age-standardized DALY rate were positively correlated with the SDI in 2019. The increase in the EAPCs of the ASIR and age-standardized DALY rate concomitant with the increase in the SDI may be attributable to the advances in medical diagnostic technology and increased investment of resources in healthcare in developed economies [13, 20]. It is known that the incidence of hip osteoarthritis is mainly associated with increasing age [21]. Developed countries tend to have an aging population, which may also partly account for the observed relationship between the EAPCs of the ASIR and age-standardized DALY rate and SDI. Interestingly, the EAPC of the ASIR and age-standardized DALY rate both declined when the SDI exceeded 0.70, indicating that the highest EAPC was in the middle SDI regions. The finding is consistent with that of Hunter [22], who reported that although years lived with disability (YLDs) for hip osteoarthritis are higher in high SDI countries than in middle SDI countries, the rate of change in YLDs since 1990 has been far greater in middle SDI countries than in high SDI countries. However, the EAPCs of the ASIR and age-standardized DALY rate in our study showed no associated with the response ASRs in 1990, which is inconsistent with the findings for other highly fatal diseases [23, 24]. This suggests that the governments and health policymakers of countries with higher ASIRs and age-standardized DALY rate do not prioritize prevention plans for hip osteoarthritis. Overall, our results indicate that it is crucial for countries with high ASIRs of hip osteoarthritis to prioritize strategies to mitigate the burden of hip osteoarthritis.

The global incidence of hip osteoarthritis both in 1990 and 2019 showed a unimodal distribution across different age groups, which peaked in the 60–64-year-old age group. A similar distribution of hip osteoarthritis was described in the GBD 2017 [5]. Furthermore, the burden of hip osteoarthritis was higher in women than in men, but there was no notable difference in the incidence and DALYs of hip osteoarthritis, their ASRs, or the EPACs between the sexes across all age groups, suggesting that sex has no association with hip osteoarthritis. Consistent with this finding, a predictive model [25] for the future risk of radiographic hip OA did not include female sex as a risk factor for hip osteoarthritis.

To our knowledge, this study provides a high-quality and recent estimate of global hip osteoarthritis burden. However, this study has several limitations. First, although, the GBD 2019 included nine additional countries and territories, it also lacked data from many sites, and the GBD estimates fill the unavailable vacancies of actual data on disease burden. Second, as GBD data are collected from various databases of uneven quality, they will inevitably contain heterogeneity and bias. Third, the overall trends in the EAPC were calculated on a linear scale and therefore do not reflect the temporal trends in the ASRs. Four, because the diagnosis of hip osteoarthritis is difficult, the global burden of hip osteoarthritis may have been underestimated. Moreover, in GBD 2019, bias adjustments were calculated using MR-BRT for the reported hip osteoarthritis using alternative case-definitions, resulting in a decrease in YLDs due to hip osteoarthritis. Finally, the DALYs of hip osteoarthritis might be underestimated in GBD study, due to time lags in national health information reports.

## Conclusions

Hip osteoarthritis is a major global public-health burden. Although the ASIR and age-standardized DALY rate of hip osteoarthritis vary among countries, the burden of hip osteoarthritis has increased in almost all countries over the past 30 years. This increasing trend is expected to continue, due to the rapid aging of the world’s population. To mitigate the burden of hip osteoarthritis, the governments and health policymakers of all countries must increase the awareness regarding risk factors, consequences of hip osteoarthritis.

## Supplementary Information

**Additional file 1.**

## Data Availability

The datasets used and/or analyzed during the current study are available from the Global Health Data Exchange (GHDx) query tool (http://ghdx.healthdata.org/gbd-results-tool).

## Abbreviations

SDI: Socio-demographic index
ASRs: Age-standardized rates
ASIR: Age-standardized incidence rate
DALYs: Disability-adjusted life years
EAPCs: The estimated annual percentage changes
GHDx: Global Health Data Exchange
