# Supplementary material for: Global, regional, and national burdens of hip osteoarthritis from 1990 to 2019: estimates from the 2019 Global Burden of Disease Study
Source: Arthritis Res Ther. 2022 Jan 3;24:8. doi: 10.1186/s13075-021-02705-6 (PMC8722328; doi:10.1186/s13075-021-02705-6)
Supplement: Supplementary file 1 — Additional file 1. [file 13075_2021_2705_MOESM1_ESM.docx]

S Table1 Three countries with the largest and lowest number of incidence or DALY.

S Table2 Three regions with the largest and lowest number of incidence or DALY.

S Table3 The incident cases and age-standardized incidence rate of national hip osteoarthritis in 1990 and 2019, and its temporal trends from 1990 to 2019.

S Table4 The incident cases and age-standardized incidence rate of national hip osteoarthritis in female in 1990 and 2019, and its temporal trends from 1990 to 2019

S Table5 The incident cases and age-standardized incidence rate of national hip osteoarthritis in male in 1990 and 2019, and its temporal trends from 1990 to 2019

S Table6 The DALY and age-standardized DALY rate of national hip osteoarthritis in 1990 and 2019, and its temporal trends from 1990 to 2019

S Table7 The DALY and age-standardized DALY rate of national hip osteoarthritis in female in 1990 and 2019, and its temporal trends from 1990 to 2019

S Table8 The DALY and age-standardized DALY rate of national hip osteoarthritis in male in 1990 and 2019, and its temporal trends from 1990 to 2019

S Table9 The incidence and DALY of regional hip osteoarthritis in 1990 and 2019, and its change from 1990 to 2019

S Table10 The age-standardized incidence and DALY rate of regional hip osteoarthritis in 1990 and 2019, and its change from 1990 to 2019

S Table11 Age distribution of incidence (per 100,000) for hip osteoarthritis in different countries in 2019.

S Table12 Age distribution of DALYs (per 100,000) for hip osteoarthritis in different countries in 2019.

S Table1 Three countries with the largest and lowest number of incidence or DALY.

| **Measure** | **sex** | **Top three countries** | | | **Bottom three countries** | | |
| --- | --- | --- | --- | --- | --- | --- | --- |
| **2019 ASIR (per 100,000 people)** | | | | | | | |
| **ASIR** | | | | | | | |
|  | both | United Kingdom (41.61) | Iceland (43.07) | United States of America (52.51) | Democratic People's Republic of Korea (8.83) | Yemen (9.81) | Timor-Leste (10.17) |
|  | female | Monaco (44.76) | Iceland (46.26) | United States of America (56.82) | Democratic People's Republic of Korea (8.30) | Yemen (9.35) | Timor-Leste (9.47) |
|  | male | United Kingdom (38.51) | Iceland (39.85) | United States of America (47.80) | Bangladesh (8.96) | Democratic People's Republic of Korea (9.40) | Nepal (9.85) |
| **Age-standardized DALY rate** | | | | | | | |
|  | both | United Kingdom (25.38) | Iceland (26.35) | United States of America (31.72) | Democratic People's Republic of Korea (5.71) | Yemen (6.28) | Timor-Leste (6.40) |
|  | female | Monaco (26.36) | Iceland (27.25) | United States of America (32.95) | Democratic People's Republic of Korea (5.39) | Yemen (5.91) | Timor-Leste (5.98) |
|  | male | United States of America (30.13) | Iceland (25.32) | United Kingdom (24.31) | Democratic People's Republic of Korea (6.17) | Bangladesh (6.23) | Yemen (6.67) |
|  |  |  |  |  |  |  |  |
| **EAPC** | | | | | | | |
| **Incidence** | | | | | | | |
|  | both | Sweden (1.64) | Equatorial Guinea (1.47) | Greece (1.40) | Denmark (-0.50) | Nigeria (-0.44) | Iceland (-0.20) |
|  | female | Sweden (2.39) | Equatorial Guinea (1.46) | Greece (1.36) | Denmark (-0.47) | Nigeria (-0.32) | Democratic Republic of the Congo (-0.13) |
|  | male | Equatorial Guinea (1.61) | Greece (1.53) | Oman (1.30) | Denmark (-0.50) | Iceland (-0.43) | Nigeria (-0.43) |
| **DALY** | | | | | | | |
|  | both | Sweden (1.64) | Equatorial Guinea (1.47) | Greece (1.40) | Denmark (-0.50) | Nigeria (-0.44) | Iceland (-0.20) |
|  | female | Sweden (2.26) | Oman (1.46) | Equatorial Guinea (1.41) | Denmark (-0.45) | Nigeria (-0.23) | Zimbabwe (-0.13) |
|  | male | Equatorial Guinea (1.67) | Greece (1.51) | Oman (1.30) | Denmark (-0.53) | Iceland (-0.34) | Nigeria (-0.31) |

S Table2 Three regions with the largest and lowest number of incidence or DALY.

| **Measure** | **sex** | **Top three regions** | | | **Bottom three regions** | | |
| --- | --- | --- | --- | --- | --- | --- | --- |
| **2019 ASIR (per 100,000 people)** | | | | | | | |
| **ASIR** | | | | | | | |
|  | both | High-income North America(50.23) | Australasia(38.74) | Western Europe(38.36) | East Asia (11.39) | South Asia (12.36) | Southeast Asia(12.51) |
|  | female | High-income North America (54.24) | Western Europe (41.32) | Australasia (39.78) | East Asia (10.46) | Southeast Asia (11.36) | Oceania (12.61) |
|  | male | High-income North America (45.87) | Australasia (37.60) | Western Europe (35.21) | South Asia(10.11) | East Asia(12.32) | Southeast Asia(13.74) |
| **Age-standardized DALY rate** | | | | | | | |
|  | both | High-income North America (30.34) | Australasia(23.72) | Western Europe(23.41) | East Asia (7.44) | Oceania (7.95) | South Asia (8.62) |
|  | female | High-income North America (31.47) | Western Europe (24.32) | Australasia (23.51) | East Asia (6.83) | Southeast Asia (7.24) | Oceania (7.99) |
|  | male | High-income North America (28.89) | Australasia(23.89) | Western Europe (22.27) | South Asia(6.96) | East Asia (8.10) | Southeast Asia(8.78) |
|  |  |  |  |  |  |  |  |
| **EAPC** | | | | | | | |
| **Incidence** | | | | | | | |
|  | both | East Asia (1.22) | Australasia (0.98) | Southern Latin America /South Asia(0.96) | Western Sub-Saharan Africa (0.02) | Central Sub-Saharan Africa (0.19) | Central Latin America (0.20) |
|  | female | East Asia (1.26) | South Asia (1.17) | Australasia (1.01) | Central Sub-Saharan Africa (0.06) | Western Sub-Saharan Africa (0.10) | Central Latin America (0.18) |
|  | male | East Asia (1.20) | Southern Latin America (1.00) | Australasia (0.95) | Western Sub-Saharan Africa (0.01) | High-income Asia Pacific (0.14) | Oceania (0.15) |
| **DALY** | | | | | | | |
|  | both | East Asia (1.14) | South Asia (1.05) | Australasia (0.97) | Western Sub-Saharan Africa (0.22) | Central Sub-Saharan Africa (0.17) | Central Latin America (0.10) |
|  | female | Southern Sub-Saharan Africa (1.24) | East Asia (1.16) | Andean Latin America (1.01) | Eastern Sub-Saharan Africa (0.21) | Central Sub-Saharan Africa (0.15) | Western Sub-Saharan Africa (0.07) |
|  | male | East Asia (1.12) | Southern Latin America (1.00) | Australasia (0.96) | Western Sub-Saharan Africa (0.17) | High-income Asia Pacific (0.14) | Oceania (0.08) |

S Table3 The incident cases and age-standardized incidence rate of national hip osteoarthritis in 1990 and 2019, and its temporal trends from 1990 to 2019.

| **Nation** | **Incident Cases No. (95% UI)** | | **Change in absolute number (%)** | **ASIR No.(95% UI)** | | **1990-2019 EAPC No. (95%CI)** |
| --- | --- | --- | --- | --- | --- | --- |
|  | **1990** | **2019** |  | **1990** | **2019** |  |
| Afghanistan | 678.24(494.65-885.15) | 1845.93(1365.30-2420.01) | 172.17 | 8.68(6.38-11.23) | 10.33(7.66-13.35) | 0.77(0.63-0.91) |
| Albania | 427.01(319.00-546.21) | 798.70(577.15-1062.07) | 87.04 | 17.67(13.16-22.72) | 20.46(15.22-26.54) | 0.56(0.53-0.58) |
| Algeria | 1509.47(1146.31-1937.18) | 5505.72(4166.82-7058.53) | 264.74 | 10.58(7.94-13.70) | 13.72(10.25-17.76) | 0.90(0.85-0.94) |
| American Samoa | 5.18(3.87-6.71) | 10.92(8.07-14.40) | 110.89 | 17.70(13.09-23.26) | 19.95(14.64-25.84) | 0.22(-0.02-0.47) |
| Andorra | 19.80(14.59-25.31) | 53.48(39.04-70.37) | 170.06 | 32.75(24.20-42.15) | 38.96(28.42-50.76) | 0.57(0.48-0.66) |
| Angola | 729.85(551.51-934.44) | 2545.28(1912.57-3231.92) | 248.74 | 13.96(10.24-18.02) | 16.46(12.10-21.31) | 0.58(0.54-0.62) |
| Antigua and Barbuda | 6.72(5.02-8.59) | 18.66(13.63-24.28) | 177.53 | 14.14(10.36-18.35) | 16.60(12.15-21.40) | 0.53(0.51-0.56) |
| Argentina | 8397.13(6106.19-10886.57) | 17139.76(12741.72-22076.69) | 104.11 | 25.75(18.76-33.40) | 34.12(25.40-43.95) | 0.98(0.87-1.09) |
| Armenia | 541.17(393.47-706.75) | 842.29(618.11-1107.36) | 55.64 | 17.38(12.79-22.40) | 20.74(15.57-26.64) | 0.64(0.62-0.65) |
| Australia | 5418.02(4019.73-6970.32) | 13277.79(9717.23-17259.20) | 145.07 | 29.20(21.72-37.81) | 39.05(28.98-50.40) | 1.01(0.92-1.10) |
| Austria | 3254.13(2378.74-4284.97) | 5239.30(3847.39-6843.74) | 61.00 | 32.65(24.26-42.31) | 37.79(28.20-48.82) | 0.48(0.45-0.51) |
| Azerbaijan | 1020.38(747.31-1329.14) | 2597.51(1899.99-3401.64) | 154.56 | 17.90(13.27-23.19) | 21.52(15.97-27.69) | 0.67(0.61-0.73) |
| Bahamas | 28.35(21.26-36.44) | 80.19(59.07-104.15) | 182.88 | 15.93(11.76-20.63) | 17.82(13.14-23.06) | 0.38(0.33-0.43) |
| Bahrain | 40.22(29.99-51.59) | 301.12(221.51-394.62) | 648.67 | 13.27(9.96-17.26) | 16.11(12.07-20.85) | 0.62(0.58-0.65) |
| Bangladesh | 5205.64(3921.40-6670.81) | 16555.04(12482.82-21249.03) | 218.02 | 8.92(6.74-11.56) | 11.29(8.46-14.40) | 0.93(0.88-0.99) |
| Barbados | 37.04(27.61-47.37) | 80.55(59.10-106.27) | 117.44 | 15.65(11.68-20.16) | 17.95(13.17-23.36) | 0.48(0.44-0.52) |
| Belarus | 2466.82(1821.51-3250.76) | 3237.07(2352.54-4261.96) | 31.22 | 19.12(14.24-24.75) | 22.11(16.29-28.74) | 0.51(0.48-0.55) |
| Belgium | 4248.81(3080.16-5600.77) | 6412.18(4646.36-8358.36) | 50.92 | 32.02(23.50-41.41) | 37.10(27.60-47.60) | 0.47(0.43-0.51) |
| Belize | 14.70(10.95-18.78) | 61.96(46.62-79.65) | 321.44 | 15.00(11.10-19.32) | 18.66(13.85-24.05) | 0.75(0.56-0.93) |
| Benin | 299.95(226.74-384.57) | 1020.23(776.48-1301.85) | 240.13 | 13.21(9.78-17.16) | 16.32(12.06-20.92) | 0.75(0.66-0.83) |
| Bermuda | 12.12(9.04-15.62) | 21.25(15.63-28.23) | 75.31 | 18.34(13.49-23.75) | 19.54(14.63-25.28) | 0.19(0.13-0.24) |
| Bhutan | 33.03(24.76-42.08) | 89.34(67.37-114.16) | 170.52 | 10.00(7.50-12.81) | 13.35(10.03-17.20) | 1.09(1.02-1.15) |
| Bolivia (Plurinational State of) | 456.00(341.65-584.71) | 1492.65(1111.61-1911.46) | 227.33 | 12.07(8.85-15.65) | 15.04(11.20-19.34) | 0.79(0.75-0.82) |
| Bosnia and Herzegovina | 857.95(624.97-1124.55) | 1119.45(808.94-1481.57) | 30.48 | 17.70(12.87-22.76) | 20.81(15.44-26.86) | 0.59(0.52-0.67) |
| Botswana | 116.79(87.46-149.57) | 411.67(312.47-523.53) | 252.49 | 16.98(12.47-21.99) | 22.20(16.44-28.41) | 0.86(0.80-0.93) |
| Brazil | 14792.96 (11012.00-18985.80) | 42996.16(31971.72-55870.50) | 190.65 | 14.16(10.50-18.35) | 17.18(12.78-22.19) | 0.71(0.68-0.73) |
| Brunei Darussalam | 29.68 (22.57-37.71) | 110.44(83.98-143.25) | 272.09 | 21.04(15.61-27.08) | 25.38(18.97-32.82) | 0.67(0.60-0.75) |
| Bulgaria | 2572.04(1887.03-3386.95) | 2555.70(1876.81-3338.32) | -0.64 | 21.06(15.74-27.20) | 22.58(16.55-28.93) | 0.17(0.13-0.21) |
| Burkina Faso | 638.51(475.58-821.93) | 1717.04(1302.73-2196.93) | 168.91 | 12.46(9.13-16.13) | 14.63(10.77-18.81) | 0.55(0.52-0.57) |
| Burundi | 375.01(283.36-478.17) | 876.40(661.20-1121.08) | 133.70 | 13.79(10.23-17.86) | 14.40(10.57-18.62) | 0.16(0.15-0.17) |
| Cabo Verde | 27.33(19.86-35.77) | 80.79(60.94-104.41) | 195.63 | 13.47(9.89-17.43) | 16.64(12.34-21.59) | 0.73(0.71-0.75) |
| Cambodia | 524.50(390.08-672.87) | 1486.09(1097.56-1921.26) | 183.34 | 9.63(7.13-12.46) | 10.57(7.76-13.66) | 0.34(0.31-0.37) |
| Cameroon | 896.83(671.96-1153.45) | 2850.29(2153.93-3671.69) | 217.82 | 16.12(12.01-20.97) | 18.05(13.47-23.45) | 0.33(0.31-0.35) |
| Canada | 7181.65(5344.78-9229.61) | 17501.53(12764.14-22974.22) | 143.70 | 23.21(17.30-29.86) | 31.02(22.94-39.31) | 0.51(0.27-0.75) |
| Central African Republic | 208.71(155.98-267.72) | 445.50(333.71-572.50) | 113.46 | 13.90(10.33-17.97) | 14.87(10.92-19.20) | 0.25(0.22-0.27) |
| Chad | 366.92(276.71-471.86) | 928.06(704.09-1196.65) | 152.93 | 11.80(8.69-15.33) | 13.04(9.63-16.90) | 0.32(0.30-0.34) |
| Chile | 2987.39(2241.82-3830.52) | 8430.05(6197.09-10991.78) | 182.19 | 27.41(20.48-35.26) | 35.92(26.72-46.41) | 0.89(0.78-0.99) |
| China | 81539.83(59898.50-106021.11) | 243109.46  (177943.72-319067.90) | 198.15 | 8.29(6.11-10.79) | 11.42(8.42-14.88) | 1.25(1.14-1.36) |
| Colombia | 2504.47(1881.76-3193.82) | 7392.01(5410.99-9690.73) | 195.15 | 11.93(8.79-15.44) | 13.96(10.30-18.11) | 0.52(0.50-0.54) |
| Comoros | 36.99(27.82-47.39) | 92.81(70.05-119.70) | 150.93 | 14.91(11.05-19.29) | 16.60(12.24-21.59) | 0.38(0.36-0.39) |
| Congo | 204.83(153.55-263.07) | 671.68(506.44-866.93) | 227.92 | 15.87(11.78-20.51) | 18.64(13.79-23.95) | 0.56(0.55-0.57) |
| Cook Islands | 2.33(1.73-3.03) | 4.80(3.52-6.37) | 105.75 | 16.22(11.96-21.13) | 20.11(14.82-26.19) | 0.58(0.41-0.74) |
| Costa Rica | 246.99(187.58-314.55) | 790.39(575.83-1025.16) | 220.01 | 12.70(9.39-16.35) | 14.86(10.87-19.23) | 0.52(0.49-0.55) |
| C么te d'Ivoire | 791.21(597.88-1015.37) | 2340.46(1772.95-3011.11) | 195.81 | 19.89(14.78-25.70) | 22.55(16.75-29.35) | 0.35(0.31-0.38) |
| Croatia | 1299.87(954.29-1710.47) | 1532.82(1116.92-2018.51) | 17.92 | 14.08(10.48-18.11) | 16.79(12.40-21.78) | 0.49(0.45-0.53) |
| Cuba | 1433.43(1075.83-1833.25) | 2944.64(2161.63-3845.19) | 105.43 | 29.53(22.04-37.80) | 35.75(26.22-46.14) | 0.67(0.62-0.72) |
| Cyprus | 243.37(181.41-312.11) | 645.80(473.99-837.87) | 165.36 | 20.39(15.09-26.44) | 23.14(17.16-29.80) | 0.69(0.63-0.76) |
| Czechia | 2616.55(1932.87-3410.96) | 3830.54(2812.81-4951.65) | 46.40 | 13.95(10.36-18.15) | 15.72(11.79-20.61) | 0.42(0.39-0.45) |
| Democratic People's Republic of Korea | 1668.49(1220.01-2196.53) | 2972.00(2146.31-3901.34) | 78.12 | 8.63(6.38-11.28) | 8.83(6.47-11.43) | 0.06(0.04-0.08) |
| Democratic Republic of the Congo | 2989.07(2232.39-3822.03) | 7248.05(5446.87-9317.89) | 142.49 | 14.89(10.96-19.21) | 15.35(11.35-19.90) | -0.01(-0.07-0.05) |
| Denmark | 2955.40(2223.41-3789.41) | 3536.01(2564.80-4663.00) | 19.65 | 44.87(33.67-57.44) | 40.25(29.91-51.82) | -0.50(-0.75--0.24) |
| Djibouti | 29.34(22.05-37.45) | 152.85(114.82-195.90) | 421.00 | 14.09(10.49-18.19) | 17.69(13.11-22.93) | 0.89(0.85-0.92) |
| Dominica | 8.43(6.24-10.93) | 14.37(10.64-18.78) | 70.37 | 14.24(10.64-18.44) | 16.74(12.44-21.74) | 0.55(0.48-0.61) |
| Dominican Republic | 579.43(435.00-741.23) | 1719.22(1271.05-2236.18) | 196.71 | 13.52(9.96-17.37) | 17.07(12.59-22.08) | 0.90(0.85-0.95) |
| Ecuador | 925.99(702.32-1187.09) | 2917.24(2161.54-3738.95) | 215.04 | 15.20(11.20-19.75) | 18.06(13.29-23.20) | 0.49(0.42-0.56) |
| Egypt | 4229.56(3212.33-5432.74) | 11637.80(8777.25-14958.91) | 175.15 | 11.75(8.76-15.08) | 14.46(10.73-18.61) | 0.50(0.40-0.59) |
| El Salvador | 383.24(288.98-491.34) | 825.12(604.44-1069.84) | 115.30 | 11.91(8.80-15.43) | 14.30(10.43-18.56) | 0.62(0.52-0.72) |
| Equatorial Guinea | 33.85(25.08-43.63) | 131.26(99.77-167.43) | 287.76 | 14.06(10.22-18.00) | 20.10(14.98-26.00) | 1.47(1.37-1.58) |
| Eritrea | 182.27(136.94-235.46) | 548.00(411.13-701.42) | 200.66 | 13.05(9.56-16.98) | 14.60(10.72-18.72) | 0.37(0.32-0.42) |
| Estonia | 395.80(294.18-515.32) | 456.96(335.79-593.29) | 15.45 | 19.95(14.91-25.79) | 23.16(17.32-29.92) | 0.63(0.58-0.67) |
| Eswatini | 76.54(58.03-97.57) | 172.92(131.33-219.16) | 125.92 | 20.47(15.32-26.36) | 23.90(17.73-30.71) | 0.33(0.13-0.53) |
| Ethiopia | 3648.02(2740.82-4645.05) | 8479.63(6497.61-10773.37) | 132.44 | 14.43(10.83-18.69) | 16.15(12.06-20.98) | 0.42(0.38-0.46) |
| Fiji | 68.42(51.52-88.15) | 157.67(116.23-205.94) | 130.43 | 14.22(10.43-18.49) | 17.32(12.78-22.44) | 0.53(0.41-0.65) |
| Finland | 2155.35(1590.17-2798.18) | 3359.06(2411.29-4436.02) | 55.85 | 33.37(24.69-43.38) | 38.71(28.66-49.87) | 0.52(0.46-0.58) |
| France | 23403.48(17203.17-30786.92) | 38186.15(27739.59-50018.54) | 63.16 | 33.35(24.94-43.62) | 38.74(28.52-50.03) | 0.59(0.34-0.85) |
| Gabon | 102.99(76.02-133.00) | 286.96(216.26-368.68) | 178.63 | 16.55(12.25-21.27) | 21.67(16.19-27.88) | 0.89(0.78-1.00) |
| Gambia | 59.74(45.43-76.28) | 181.69(137.02-230.31) | 204.12 | 13.33(9.85-17.24) | 15.53(11.57-19.98) | 0.49(0.45-0.53) |
| Georgia | 1306.68(943.16-1702.09) | 1108.93(810.75-1446.22) | -15.13 | 20.33(15.04-26.36) | 21.23(16.03-27.33) | 0.13(0.11-0.16) |
| Germany | 36605.08(26508.99-47687.15) | 52788.43(37947.80-69412.96) | 44.21 | 33.20(24.41-42.87) | 38.49(28.80-49.65) | 0.48(0.43-0.53) |
| Ghana | 1055.04(792.55-1343.81) | 3400.90(2591.51-4388.89) | 222.35 | 13.24(9.71-17.05) | 16.46(12.16-21.33) | 0.71(0.64-0.77) |
| Greece | 4180.08(3059.42-5462.44) | 5646.39(4099.95-7373.81) | 35.08 | 29.91(22.16-38.38) | 34.26(25.39-44.39) | 1.40(1.00-1.80) |
| Greenland | 11.28(8.56-14.49) | 23.96(17.37-31.59) | 112.37 | 24.51(18.16-31.35) | 30.89(22.99-39.52) | 0.81(0.74-0.88) |
| Grenada | 7.93(5.80-10.40) | 19.43(14.46-25.59) | 144.97 | 13.06(9.55-17.04) | 15.83(11.79-20.53) | 0.65(0.59-0.71) |
| Guam | 16.46(12.31-21.05) | 37.99(27.57-49.64) | 130.88 | 16.29(11.94-21.06) | 19.30(14.16-24.89) | 0.59(0.54-0.65) |
| Guatemala | 475.04(357.00-610.95) | 1539.51(1148.88-1980.30) | 224.08 | 10.61(7.84-13.70) | 12.60(9.25-16.45) | 0.58(0.54-0.62) |
| Guinea | 472.63(353.53-603.72) | 929.19(697.38-1192.60) | 96.60 | 12.80(9.38-16.53) | 14.15(10.39-18.32) | 0.32(0.31-0.34) |
| Guinea-Bissau | 62.98(47.73-80.27) | 134.93(102.12-172.70) | 114.24 | 12.76(9.37-16.41) | 13.98(10.24-18.11) | 0.26(0.22-0.30) |
| Guyana | 61.62(46.71-79.04) | 117.60(87.14-152.35) | 90.84 | 13.62(10.07-17.77) | 16.00(11.81-20.54) | 0.55(0.49-0.61) |
| Haiti | 438.12(325.29-561.31) | 1079.18(810.52-1377.52) | 146.32 | 11.21(8.26-14.48) | 12.36(9.08-15.92) | 0.40(0.37-0.43) |
| Honduras | 265.64(197.62-338.67) | 917.00(689.66-1165.57) | 245.21 | 11.03(8.12-14.22) | 13.13(9.67-16.88) | 0.62(0.55-0.69) |
| Hungary | 2963.94(2203.46-3857.23) | 3624.95(2654.32-4801.64) | 22.30 | 21.51(16.14-27.76) | 23.71(17.57-30.81) | 0.30(0.26-0.33) |
| Iceland | 109.79(86.34-135.26) | 201.96(147.77-265.72) | 83.95 | 43.08(33.74-53.15) | 43.07(32.29-55.87) | -0.20(-0.34--0.07) |
| India | 57624.38(43037.41-74237.23) | 160500.02  (120106.60-207439.20) | 178.53 | 9.72(7.23-12.60) | 12.33(9.21-15.99) | 0.96(0.79-1.14) |
| Indonesia | 13201.24(9874.38-17057.73) | 34443.21(25609.10-44566.66) | 160.91 | 10.73(7.92-13.95) | 12.79(9.51-16.62) | 0.66(0.62-0.69) |
| Iran (Islamic Republic of) | 3825.83(2838.43-4936.68) | 11933.01(9005.57-15257.11) | 211.91 | 11.68(8.67-15.05) | 13.57(10.04-17.47) | 0.35(0.18-0.51) |
| Iraq | 1135.76(861.58-1445.83) | 4053.58(3081.01-5217.99) | 256.91 | 12.42(9.32-15.97) | 13.56(10.13-17.46) | 0.31(0.26-0.36) |
| Ireland | 1208.96(900.19-1570.72) | 2565.80(1904.49-3331.24) | 112.23 | 32.79(24.35-42.18) | 38.34(28.54-49.51) | 0.52(0.48-0.57) |
| Israel | 1395.17(1036.13-1801.60) | 3670.08(2727.40-4774.40) | 163.06 | 30.98(22.93-39.95) | 36.01(26.72-46.50) | 0.46(0.38-0.53) |
| Italy | 25002.39(18189.26-32867.67) | 35622.93(26080.70-46806.61) | 42.48 | 32.01(23.68-41.50) | 35.47(26.18-45.72) | 0.51(0.35-0.67) |
| Jamaica | 226.85(172.06-290.96) | 498.38(367.05-647.38) | 119.70 | 13.83(10.33-17.83) | 16.65(12.28-21.61) | 0.71(0.65-0.77) |
| Japan | 36689.29(27068.12-47574.39) | 48799.10(35680.35-63833.37) | 33.01 | 21.48(15.92-27.79) | 23.23(17.17-30.08) | 0.24(0.13-0.35) |
| Jordan | 212.00(160.00-270.54) | 1361.19(1020.37-1765.77) | 542.08 | 11.80(8.78-15.14) | 15.07(11.26-19.50) | 0.85(0.81-0.90) |
| Kazakhstan | 2902.46(2183.84-3782.73) | 4682.23(3461.66-6074.73) | 61.32 | 20.59(15.39-26.79) | 23.44(17.24-30.19) | 0.36(0.33-0.40) |
| Kenya | 1591.25(1204.64-2035.21) | 5415.92(4112.88-6898.68) | 240.36 | 15.80(11.78-20.47) | 18.43(13.70-23.82) | 0.55(0.49-0.60) |
| Kiribati | 6.06(4.55-7.83) | 13.46(9.98-17.46) | 122.17 | 13.28(9.74-17.28) | 15.05(11.15-19.51) | 0.29(0.09-0.49) |
| Kuwait | 150.08(112.59-192.70) | 783.32(579.34-1032.71) | 421.92 | 14.07(10.57-18.14) | 17.02(12.73-21.83) | 0.73(0.69-0.77) |
| Kyrgyzstan | 556.97(407.99-724.16) | 1070.89(793.44-1410.32) | 92.27 | 17.41(12.86-22.70) | 19.05(14.38-24.80) | 0.21(0.17-0.25) |
| Lao People's Democratic Republic | 242.82(180.01-313.46) | 626.35(470.17-806.18) | 157.94 | 9.88(7.22-12.83) | 11.40(8.42-14.83) | 0.56(0.53-0.59) |
| Latvia | 695.65(514.88-899.27) | 688.50(501.67-906.44) | -1.03 | 20.17(14.90-25.99) | 23.08(17.14-29.93) | 0.50(0.47-0.52) |
| Lebanon | 287.19(212.20-371.32) | 738.77(550.63-944.86) | 157.24 | 11.14(8.31-14.32) | 14.10(10.38-18.05) | 0.53(0.14-0.92) |
| Lesotho | 182.63(135.94-234.87) | 305.16(231.93-385.75) | 67.09 | 16.41(12.05-21.16) | 19.87(14.75-25.43) | 0.65(0.63-0.67) |
| Liberia | 170.23(127.76-217.63) | 472.35(354.11-609.18) | 177.49 | 13.91(10.25-18.02) | 16.67(12.31-21.73) | 0.79(0.69-0.89) |
| Libya | 283.94(214.66-362.02) | 969.72(727.56-1266.38) | 241.52 | 12.60(9.46-16.29) | 14.41(10.61-18.72) | 0.47(0.43-0.50) |
| Lithuania | 872.96(644.14-1150.52) | 984.39(718.47-1308.75) | 12.76 | 19.74(14.61-25.67) | 22.38(16.80-29.11) | 0.49(0.43-0.54) |
| Luxembourg | 166.14(122.06-215.37) | 342.58(249.51-445.39) | 106.20 | 33.25(24.61-42.72) | 38.20(27.91-49.39) | 0.46(0.43-0.49) |
| Madagascar | 843.50(627.96-1083.97) | 2246.19(1681.95-2877.52) | 166.29 | 13.81(10.25-17.95) | 14.84(10.97-19.23) | 0.28(0.25-0.30) |
| Malawi | 689.89(518.98-879.45) | 1516.85(1148.31-1946.60) | 119.87 | 14.53(10.82-18.76) | 16.60(12.21-21.48) | 0.54(0.48-0.59) |
| Malaysia | 1328.15(997.37-1710.74) | 4472.38(3293.69-5820.47) | 236.74 | 11.75(8.70-15.38) | 14.56(10.74-18.98) | 0.79(0.77-0.81) |
| Maldives | 11.77(8.57-15.35) | 57.22(42.74-73.64) | 386.32 | 10.20(7.49-13.21) | 13.30(9.85-17.39) | 1.06(0.95-1.17) |
| Mali | 601.06(446.13-775.56) | 1478.70(1127.10-1881.95) | 146.02 | 12.19(8.98-15.94) | 13.89(10.32-17.97) | 0.48(0.45-0.51) |
| Malta | 137.35(101.25-177.50) | 271.55(196.45-357.91) | 97.70 | 32.08(23.76-41.51) | 38.67(29.02-49.84) | 0.59(0.48-0.70) |
| Marshall Islands | 2.40(1.82-3.07) | 6.59(4.92-8.51) | 174.31 | 12.04(8.85-15.59) | 14.29(10.59-18.66) | 0.48(0.36-0.59) |
| Mauritania | 171.57(129.53-219.53) | 436.42(328.35-558.69) | 154.36 | 15.13(11.19-19.69) | 17.76(13.13-22.94) | 0.48(0.41-0.54) |
| Mauritius | 101.02(75.51-129.91) | 261.00(187.21-343.79) | 158.37 | 11.86(8.73-15.42) | 14.28(10.47-18.58) | 0.66(0.64-0.69) |
| Mexico | 7208.73(5445.82-9198.38) | 20715.58(15338.89-26779.73) | 187.37 | 14.32(10.59-18.69) | 16.25(12.06-21.13) | -0.09(-0.27-0.09) |
| Micronesia (Federated States of) | 7.78(5.86-10.05) | 14.46(10.73-18.84) | 85.80 | 14.13(10.41-18.41) | 15.98(11.84-20.58) | 0.30(0.12-0.48) |
| Monaco | 18.04(13.01-23.43) | 27.46(19.70-36.18) | 52.21 | 36.51(27.09-47.04) | 41.27(30.63-53.01) | 0.39(0.34-0.44) |
| Mongolia | 201.62(148.55-259.62) | 605.70(451.60-784.10) | 200.42 | 17.11(12.62-22.22) | 19.03(14.18-24.55) | 0.37(0.34-0.41) |
| Montenegro | 138.28(103.64-181.85) | 201.85(148.00-266.37) | 45.97 | 20.77(15.60-27.16) | 22.22(16.59-28.77) | 0.26(0.23-0.29) |
| Morocco | 1645.83(1242.56-2109.06) | 4686.50(3454.12-6079.48) | 184.75 | 10.22(7.56-13.18) | 12.74(9.39-16.36) | 0.73(0.71-0.76) |
| Mozambique | 1046.64(788.23-1346.31) | 2218.72(1664.62-2824.66) | 111.99 | 13.98(10.26-18.13) | 15.74(11.58-20.33) | 0.47(0.44-0.49) |
| Myanmar | 2666.87(1980.46-3463.45) | 6092.95(4465.65-7892.37) | 128.47 | 9.82(7.19-12.78) | 11.31(8.29-14.85) | 0.56(0.51-0.61) |
| Namibia | 133.08(99.19-168.76) | 321.85(243.60-405.11) | 141.85 | 16.69(12.34-21.38) | 19.22(14.22-24.77) | 0.43(0.37-0.48) |
| Nauru | 0.81(0.60-1.06) | 1.09(0.81-1.42) | 35.36 | 14.62(10.71-19.08) | 16.54(12.26-21.38) | 0.20(0.11-0.30) |
| Nepal | 1107.58(829.89-1416.43) | 3194.17(2408.80-4085.16) | 188.39 | 9.18(6.87-11.80) | 12.54(9.50-15.98) | 1.17(1.10-1.25) |
| Netherlands | 6505.08(5013.66-8051.75) | 10377.71(7549.39-13797.15) | 59.53 | 36.17(27.99-44.69) | 38.37(28.49-49.36) | 0.18(0.09-0.28) |
| New Zealand | 1060.03(793.93-1374.57) | 2387.90(1748.53-3140.38) | 125.27 | 29.20(21.86-37.69) | 37.06(27.44-48.09) | 0.82(0.76-0.89) |
| Nicaragua | 209.35(156.83-265.98) | 712.38(533.60-915.00) | 240.29 | 11.43(8.33-14.76) | 13.58(10.07-17.53) | 0.56(0.47-0.65) |
| Niger | 453.21(344.58-576.07) | 1306.67(980.63-1677.45) | 188.32 | 12.30(9.23-15.94) | 13.10(9.65-16.99) | 0.22(0.19-0.25) |
| Nigeria | 7597.88(5730.39-9741.71) | 16282.25(12317.03-20816.74) | 114.30 | 14.58(10.80-19.00) | 14.09(10.38-18.27) | -0.44(-0.68--0.20) |
| Niue | 0.30(0.23-0.39) | 0.40(0.30-0.52) | 32.00 | 15.10(11.22-19.59) | 18.74(13.96-24.12) | 0.68(0.53-0.84) |
| North Macedonia | 402.31(298.76-524.69) | 694.12(511.59-907.82) | 72.53 | 19.27(14.33-24.89) | 21.76(16.24-28.18) | 0.40(0.39-0.40) |
| Northern Mariana Islands | 4.97(3.71-6.55) | 12.12(8.68-16.37) | 143.75 | 16.59(12.27-21.49) | 18.03(13.26-23.34) | 0.14(-0.03-0.31) |
| Norway | 1701.90(1255.60-2190.83) | 2932.85(2163.89-3840.81) | 72.33 | 32.59(24.34-41.99) | 37.98(28.12-48.98) | 0.55(0.53-0.57) |
| Oman | 103.11(75.86-131.67) | 505.06(375.37-651.20) | 389.82 | 10.13(7.53-13.06) | 14.84(11.20-19.05) | 1.37(1.34-1.40) |
| Pakistan | 7038.53(5319.65-8957.03) | 20041.78(15113.68-25788.52) | 184.74 | 10.70(8.01-13.71) | 13.70(10.34-17.75) | 0.96(0.91-1.02) |
| Palau | 1.78(1.36-2.27) | 5.07(3.72-6.68) | 184.40 | 15.66(11.72-20.23) | 18.97(13.98-24.53) | 0.50(0.32-0.68) |
| Palestine | 107.80(80.17-137.76) | 395.89(299.46-508.10) | 267.23 | 10.99(8.19-14.20) | 12.54(9.36-16.22) | 0.34(0.27-0.40) |
| Panama | 175.53(131.24-225.09) | 582.46(433.42-752.49) | 231.82 | 10.67(7.80-13.84) | 13.84(10.33-17.83) | 0.80(0.75-0.85) |
| Papua New Guinea | 272.65(203.86-349.45) | 807.34(600.05-1040.42) | 196.11 | 11.45(8.33-14.87) | 12.49(9.25-16.26) | 0.19(0.12-0.27) |
| Paraguay | 331.81(250.04-420.97) | 955.42(707.34-1228.16) | 187.94 | 13.40(9.86-17.32) | 15.65(11.49-20.14) | 0.53(0.50-0.56) |
| Peru | 1761.28(1310.42-2261.64) | 5402.71(4008.15-7024.12) | 206.75 | 12.93(9.55-16.75) | 16.21(11.93-21.18) | 0.83(0.80-0.86) |
| Philippines | 4095.12(3060.82-5250.71) | 12015.66(8929.14-15569.85) | 193.41 | 11.10(8.17-14.41) | 12.81(9.53-16.65) | 0.48(0.46-0.50) |
| Poland | 8982.61(6695.32-11675.18) | 14335.46(10523.31-18624.59) | 59.59 | 20.69(15.39-26.73) | 24.45(18.10-31.68) | 0.57(0.56-0.57) |
| Portugal | 3853.89(2790.12-5064.11) | 6165.02(4422.78-8117.08) | 59.97 | 29.85(22.13-38.67) | 35.33(26.06-45.76) | 0.02(-0.18-0.21) |
| Puerto Rico | 652.70(484.82-842.32) | 1161.54(851.24-1516.77) | 77.96 | 18.47(13.66-23.89) | 21.92(16.21-28.39) | 0.68(0.62-0.73) |
| Qatar | 44.30(31.94-58.86) | 456.70(330.68-602.79) | 931.03 | 15.35(11.47-19.94) | 18.12(13.59-23.52) | 0.51(0.42-0.61) |
| Republic of Korea | 8352.31(6243.59-10826.15) | 21790.26(15914.24-28761.15) | 160.89 | 22.55(16.63-29.18) | 24.67(18.38-32.14) | 0.30(0.20-0.39) |
| Republic of Moldova | 847.72(626.58-1104.71) | 1215.73(901.53-1593.51) | 43.41 | 17.96(13.21-23.19) | 22.15(16.45-28.51) | 0.81(0.70-0.91) |
| Romania | 5522.29(4016.60-7234.82) | 6799.52(5002.32-8866.61) | 23.13 | 19.28(14.22-24.90) | 22.58(17.00-29.27) | 0.54(0.52-0.56) |
| Russian Federation | 36600.78(27068.45-47643.60) | 51246.09(37924.87-67206.62) | 40.01 | 19.94(14.80-25.84) | 23.49(17.55-30.26) | 0.56(0.54-0.58) |
| Rwanda | 498.08(373.53-640.51) | 1250.56(949.59-1592.86) | 151.08 | 14.30(10.67-18.64) | 16.11(12.14-20.78) | 0.45(0.41-0.48) |
| Saint Kitts and Nevis | 4.67(3.45-6.10) | 14.42(10.66-18.67) | 208.88 | 15.08(11.03-19.50) | 18.22(13.49-23.32) | 0.64(0.60-0.69) |
| Saint Lucia | 12.18(9.01-15.65) | 38.14(28.42-49.65) | 213.04 | 13.89(10.34-17.85) | 16.95(12.61-21.84) | 0.63(0.57-0.69) |
| Saint Vincent and the Grenadines | 9.29(6.94-11.99) | 23.29(17.26-30.33) | 150.85 | 13.37(9.85-17.38) | 16.67(12.34-21.57) | 0.84(0.78-0.89) |
| Samoa | 15.30(11.38-19.74) | 28.56(21.46-37.00) | 86.65 | 15.63(11.69-20.18) | 17.01(12.64-21.93) | 0.12(-0.02-0.27) |
| San Marino | 10.20(7.49-13.27) | 19.30(14.23-25.00) | 89.15 | 34.60(25.60-44.71) | 39.21(29.08-50.27) | 0.43(0.37-0.49) |
| Sao Tome and Principe | 9.67(7.11-12.58) | 22.76(17.20-29.21) | 135.38 | 14.08(10.41-18.25) | 16.65(12.36-21.64) | 0.56(0.54-0.58) |
| Saudi Arabia | 987.22(744.04-1269.72) | 5298.98(3904.01-6961.20) | 436.75 | 11.85(8.88-15.20) | 16.17(12.07-20.87) | 1.04(1.00-1.07) |
| Senegal | 536.45(403.99-684.22) | 1389.26(1053.69-1784.06) | 158.98 | 14.07(10.35-18.14) | 15.35(11.38-19.89) | 0.23(0.19-0.26) |
| Serbia | 2453.22(1803.08-3207.76) | 2979.16(2173.18-3933.51) | 21.44 | 19.94(14.90-25.92) | 22.76(16.86-29.22) | 0.51(0.48-0.53) |
| Seychelles | 7.09(5.28-9.15) | 18.82(14.01-24.55) | 165.43 | 12.82(9.53-16.62) | 14.78(10.90-19.15) | 0.44(0.40-0.47) |
| Sierra Leone | 271.67(205.61-347.34) | 648.77(493.66-829.20) | 138.81 | 12.77(9.42-16.43) | 14.44(10.74-18.66) | 0.38(0.35-0.41) |
| Singapore | 572.94(434.14-731.61) | 2145.42(1578.44-2769.39) | 274.46 | 21.14(15.59-27.40) | 25.33(18.71-32.62) | 0.60(0.54-0.66) |
| Slovakia | 1197.28(889.89-1562.08) | 1914.60(1402.44-2521.14) | 59.91 | 20.63(15.33-26.81) | 23.07(17.18-30.01) | 0.30(0.26-0.34) |
| Slovenia | 486.24(362.88-635.59) | 781.16(569.93-1022.77) | 60.65 | 20.12(15.16-26.15) | 23.34(17.29-30.13) | 0.52(0.48-0.56) |
| Solomon Islands | 21.46(15.84-27.75) | 57.36(43.26-73.30) | 167.36 | 12.24(8.95-15.87) | 13.81(10.27-17.97) | 0.31(0.14-0.47) |
| Somalia | 499.24(375.86-642.29) | 1331.88(1008.51-1707.68) | 166.78 | 13.95(10.33-17.94) | 14.64(10.75-18.89) | 0.22(0.20-0.24) |
| South Africa | 5050.90(3776.22-6481.58) | 12564.17(9328.84-16333.48) | 148.75 | 21.14(15.54-27.43) | 24.19(17.71-31.67) | 0.51(0.48-0.54) |
| South Sudan | 411.21(311.11-525.47) | 820.07(611.80-1055.48) | 99.43 | 14.54(10.82-18.97) | 15.89(11.68-20.60) | 0.34(0.32-0.37) |
| Spain | 17010.68(12461.14-22586.36) | 29392.85(21534.87-38133.04) | 72.79 | 35.01(26.05-45.37) | 40.24(29.43-51.90) | 0.37(0.24-0.51) |
| Sri Lanka | 1378.49(1030.96-1785.18) | 3233.80(2381.25-4248.47) | 134.59 | 10.63(7.83-13.97) | 12.06(8.95-15.65) | 0.47(0.45-0.49) |
| Sudan | 965.94(732.71-1234.94) | 2938.63(2202.48-3746.30) | 204.22 | 8.89(6.63-11.48) | 12.22(8.96-15.71) | 1.10(1.02-1.19) |
| Suriname | 41.71(30.51-54.35) | 114.78(84.64-150.67) | 175.18 | 14.41(10.50-18.64) | 17.50(12.88-22.83) | 0.70(0.68-0.73) |
| Sweden | 2851.48(2137.37-3639.26) | 5483.87(3934.83-7328.44) | 92.32 | 24.43(18.45-30.96) | 37.14(27.19-48.89) | 1.64(1.21-2.08) |
| Switzerland | 2826.65(2076.43-3671.89) | 4758.81(3415.53-6294.49) | 68.36 | 32.02(23.79-41.48) | 35.16(26.01-45.68) | 0.33(0.32-0.35) |
| Syrian Arab Republic | 712.21(539.97-913.77) | 1947.48(1448.88-2525.33) | 173.44 | 11.21(8.43-14.51) | 13.00(9.68-16.75) | 0.39(0.31-0.47) |
| Taiwan (Province of China) | 1885.20(1400.29-2454.73) | 4553.00(3249.31-5994.24) | 141.51 | 10.31(7.57-13.42) | 12.03(8.81-15.60) | 0.68(0.60-0.77) |
| Tajikistan | 479.78(353.82-624.90) | 1186.02(877.45-1559.68) | 147.20 | 15.77(11.69-20.38) | 16.74(12.29-21.73) | 0.14(0.10-0.18) |
| Thailand | 4610.65(3414.98-5937.48) | 14763.56(10983.64-19451.36) | 220.21 | 10.56(7.79-13.79) | 13.96(10.31-18.27) | 1.00(0.97-1.03) |
| Timor-Leste | 39.52(29.89-51.19) | 91.14(67.63-116.45) | 130.63 | 9.74(7.17-12.70) | 10.17(7.51-13.08) | 0.17(0.14-0.21) |
| Togo | 207.51(157.99-264.97) | 718.43(545.05-919.10) | 246.21 | 12.86(9.42-16.61) | 14.55(10.78-18.94) | 0.35(0.31-0.39) |
| Tokelau | 0.17(0.12-0.22) | 0.23(0.17-0.30) | 39.02 | 13.54(9.97-17.50) | 16.92(12.43-21.80) | 0.75(0.63-0.88) |
| Tonga | 9.30(6.81-12.16) | 14.22(10.49-18.34) | 52.87 | 14.81(10.88-19.33) | 17.05(12.60-22.06) | 0.25(0.00-0.50) |
| Trinidad and Tobago | 137.29(102.91-174.82) | 333.03(245.33-436.84) | 142.57 | 15.27(11.35-19.59) | 17.81(13.24-23.09) | 0.64(0.58-0.70) |
| Tunisia | 619.02(464.41-800.67) | 1792.04(1332.78-2305.92) | 189.50 | 10.81(8.12-14.00) | 13.08(9.75-16.79) | 0.63(0.59-0.66) |
| Turkey | 4896.71(3664.00-6293.68) | 13688.44(10198.37-17612.15) | 179.54 | 11.61(8.69-15.09) | 14.37(10.71-18.50) | 0.67(0.63-0.71) |
| Turkmenistan | 397.30(297.32-508.15) | 1031.82(766.91-1348.58) | 159.71 | 18.05(13.49-23.23) | 21.35(15.96-27.70) | 0.53(0.50-0.56) |
| Tuvalu | 1.00(0.74-1.29) | 1.74(1.27-2.27) | 73.39 | 13.12(9.73-17.00) | 15.53(11.36-20.21) | 0.51(0.37-0.66) |
| Uganda | 1093.32(818.87-1406.50) | 2895.77(2193.02-3752.14) | 164.86 | 14.08(10.44-18.23) | 15.79(11.68-20.51) | 0.45(0.43-0.48) |
| Ukraine | 14288.73(10547.59-18707.19) | 15046.28(11038.98-19654.13) | 5.30 | 20.58(15.27-26.52) | 22.02(16.32-28.43) | 0.26(0.23-0.28) |
| United Arab Emirates | 135.09(95.28-180.15) | 1819.33(1292.62-2459.11) | 1246.75 | 12.43(9.18-16.03) | 16.23(12.17-20.88) | 0.92(0.88-0.96) |
| United Kingdom | 26808.15(19692.27-35027.17) | 40397.09(29691.81-52869.61) | 50.69 | 36.28(27.11-46.75) | 41.61(30.98-53.74) | 0.50(0.46-0.54) |
| United Republic of Tanzania | 1956.58(1472.74-2502.57) | 5445.34(4103.14-7028.08) | 178.31 | 15.06(11.15-19.37) | 17.60(12.90-23.16) | 0.57(0.55-0.60) |
| United States of America | 115765.86(86999.07-148302.23) | 245800.42(184796.23-311183.89) | 112.33 | 41.93(31.28-54.43) | 52.51(40.96-65.39) | 0.60(0.50-0.70) |
| United States Virgin Islands | 18.65(14.03-24.02) | 33.49(24.43-44.13) | 79.57 | 18.21(13.37-23.53) | 20.76(15.37-26.76) | 0.45(0.39-0.51) |
| Uruguay | 940.27(687.85-1244.10) | 1516.06(1108.43-1960.67) | 61.24 | 26.39(19.48-34.23) | 34.72(25.89-44.69) | 0.95(0.84-1.05) |
| Uzbekistan | 2161.03(1612.98-2802.57) | 6049.91(4513.63-7887.03) | 179.95 | 17.23(12.72-22.30) | 20.39(15.24-26.47) | 0.54(0.49-0.59) |
| Vanuatu | 10.45(7.81-13.36) | 29.18(22.00-37.55) | 179.35 | 12.89(9.45-16.65) | 14.37(10.58-18.64) | 0.36(0.32-0.39) |
| Venezuela (Bolivarian Republic of) | 1448.94(1100.13-1843.25) | 4574.38(3363.11-5933.85) | 215.71 | 12.60(9.31-16.37) | 14.53(10.73-18.76) | 0.48(0.45-0.51) |
| Viet Nam | 4090.76(3049.12-5337.81) | 11629.01(8447.49-15243.97) | 184.28 | 9.49(7.08-12.29) | 10.63(7.75-13.85) | 0.45(0.41-0.49) |
| Yemen | 522.12(392.17-664.31) | 1721.00(1294.93-2176.19) | 229.62 | 8.38(6.18-10.85) | 9.81(7.23-12.57) | 0.60(0.57-0.63) |
| Zambia | 545.94(411.32-698.69) | 1532.81(1159.16-1966.54) | 180.76 | 15.17(11.23-19.67) | 16.98(12.53-21.99) | 0.36(0.32-0.41) |
| Zimbabwe | 858.62(656.98-1095.36) | 1586.77(1204.66-2012.56) | 84.80 | 17.30(12.91-22.26) | 17.76(13.15-22.75) | -0.06(-0.14-0.02) |

S Table4 The incident cases and age-standardized incidence rate of national hip osteoarthritis in female in 1990 and 2019, and its temporal trends from 1990 to 2019

| **Nation** | **Incident Cases No. (95% UI)** | | **Change in absolute number (%)** | **ASIR No.(95% UI)** | | **1990-2019 EAPC No. (95%CI)** |
| --- | --- | --- | --- | --- | --- | --- |
|  | **1990** | **2019** |  | **1990** | **2019** |  |
| Afghanistan | 317.87(230.07-410.10) | 869.81(646.99-1143.04) | 173.63 | 8.11(5.99-10.47) | 9.82(7.24-12.72) | 0.84(0.69-0.99) |
| Albania | 187.40(139.39-240.41) | 388.33(279.60-518.17) | 107.22 | 16.27(12.07-21.09) | 19.37(14.33-25.18) | 0.67(0.63-0.70) |
| Algeria | 720.38(544.07-923.87) | 2588.23(1952.83-3307.61) | 259.29 | 9.91(7.40-12.83) | 13.03(9.73-16.75) | 0.96(0.90-1.03) |
| American Samoa | 2.21(1.67-2.86) | 5.12(3.75-6.81) | 131.21 | 15.98(12.03-20.77) | 18.48(13.52-24.14) | 0.33(0.08-0.58) |
| Andorra | 10.02(7.47-12.85) | 27.87(20.51-36.49) | 178.17 | 35.28(26.36-45.49) | 42.34(31.08-54.87) | 0.60(0.50-0.70) |
| Angola | 342.15(257.12-437.82) | 1266.80(951.47-1611.05) | 270.25 | 13.36(9.74-17.31) | 15.39(11.36-19.98) | 0.49(0.44-0.53) |
| Antigua and Barbuda | 3.41(2.52-4.40) | 9.03(6.61-11.74) | 164.93 | 13.40(9.81-17.30) | 15.43(11.29-20.01) | 0.46(0.44-0.48) |
| Argentina | 4626.67(3345.50-6016.04) | 9132.94(6744.77-11868.44) | 97.40 | 26.79(19.56-34.70) | 34.46(25.55-44.75) | 0.95(0.85-1.05) |
| Armenia | 266.67(194.44-350.24) | 426.87(310.47-562.80) | 60.08 | 16.05(11.92-20.68) | 19.19(14.39-25.14) | 0.66(0.64-0.67) |
| Australia | 2848.09(2092.88-3656.88) | 7097.51(5173.72-9398.99) | 149.20 | 30.24(22.46-39.14) | 40.13(29.85-51.83) | 1.04(0.94-1.13) |
| Austria | 1905.13(1389.03-2522.60) | 2929.42(2150.17-3831.39) | 53.77 | 34.74(25.78-45.27) | 40.77(30.49-52.30) | 0.53(0.49-0.56) |
| Azerbaijan | 507.02(373.14-665.18) | 1260.86(919.54-1654.47) | 148.68 | 16.53(12.40-21.52) | 19.85(14.84-25.80) | 0.68(0.61-0.74) |
| Bahamas | 14.28(10.75-18.33) | 39.43(28.83-51.84) | 176.13 | 15.12(11.23-19.58) | 16.59(12.16-21.57) | 0.32(0.28-0.36) |
| Bahrain | 14.03(10.54-18.16) | 91.98(68.60-119.53) | 555.47 | 12.23(9.23-15.85) | 15.03(11.38-19.37) | 0.68(0.63-0.72) |
| Bangladesh | 2946.16(2230.67-3789.45) | 10020.02(7617.62-12898.47) | 240.10 | 10.87(8.31-14.20) | 13.57(10.19-17.34) | 0.88(0.83-0.93) |
| Barbados | 19.35(14.45-25.01) | 39.93(29.37-52.83) | 106.41 | 14.83(11.05-19.05) | 16.71(12.38-21.74) | 0.42(0.39-0.45) |
| Belarus | 1268.22(920.43-1680.44) | 1649.05(1180.52-2202.33) | 30.03 | 17.13(12.82-22.00) | 20.11(14.67-26.37) | 0.63(0.59-0.68) |
| Belgium | 2394.57(1731.13-3167.57) | 3554.37(2559.50-4656.73) | 48.43 | 34.30(24.88-44.27) | 40.00(29.17-51.37) | 0.51(0.47-0.54) |
| Belize | 6.68(4.98-8.53) | 28.68(21.53-36.97) | 329.19 | 14.12(10.34-18.14) | 17.30(12.84-22.52) | 0.69(0.51-0.87) |
| Benin | 153.08(115.04-196.28) | 503.74(381.48-640.53) | 229.06 | 12.74(9.51-16.60) | 15.62(11.53-20.07) | 0.74(0.67-0.81) |
| Bermuda | 6.07(4.47-7.83) | 10.23(7.42-13.48) | 68.41 | 17.39(12.82-22.46) | 18.10(13.32-23.32) | 0.11(0.06-0.16) |
| Bhutan | 19.44(14.63-24.73) | 52.26(39.64-66.66) | 168.81 | 12.11(9.05-15.34) | 16.28(12.19-20.77) | 1.12(1.05-1.19) |
| Bolivia (Plurinational State of) | 229.23(172.11-295.20) | 707.45(523.20-906.55) | 208.63 | 11.67(8.62-15.13) | 13.96(10.36-17.88) | 0.65(0.61-0.68) |
| Bosnia and Herzegovina | 419.26(307.81-542.26) | 553.17(399.52-732.17) | 31.94 | 16.39(12.21-20.83) | 19.70(14.48-25.51) | 0.69(0.61-0.77) |
| Botswana | 52.89(39.42-67.98) | 175.90(134.06-225.24) | 232.55 | 14.32(10.56-18.63) | 18.02(13.41-23.41) | 0.74(0.68-0.80) |
| Brazil | 7182.05(5337.29-9212.50) | 20521.51(15337.83-26730.32) | 185.73 | 13.27(9.83-17.19) | 15.53(11.61-20.04) | 0.57(0.55-0.58) |
| Brunei Darussalam | 13.42(10.19-16.89) | 54.22(40.33-70.73) | 304.04 | 21.45(15.84-27.66) | 25.85(19.03-33.71) | 0.74(0.66-0.83) |
| Bulgaria | 1236.79(890.15-1628.77) | 1270.01(919.67-1655.09) | 2.69 | 19.49(14.35-25.16) | 21.37(15.64-27.43) | 0.27(0.23-0.30) |
| Burkina Faso | 328.08(244.56-419.15) | 886.76(667.64-1125.99) | 170.29 | 12.02(8.84-15.48) | 14.00(10.39-18.12) | 0.55(0.54-0.56) |
| Burundi | 194.24(146.05-247.98) | 373.54(282.23-474.59) | 92.31 | 13.23(9.82-17.11) | 13.05(9.66-16.92) | -0.05(-0.09--0.01) |
| Cabo Verde | 15.46(11.33-20.30) | 39.84(29.71-51.94) | 157.62 | 13.05(9.63-16.99) | 15.93(11.78-20.77) | 0.70(0.69-0.72) |
| Cambodia | 284.62(211.52-366.11) | 767.76(562.60-993.88) | 169.74 | 9.22(6.80-11.99) | 9.91(7.21-12.81) | 0.28(0.25-0.31) |
| Cameroon | 441.35(330.23-573.63) | 1369.91(1030.10-1772.23) | 210.39 | 15.59(11.52-20.34) | 17.24(12.89-22.33) | 0.32(0.29-0.35) |
| Canada | 3927.16(2884.25-5060.37) | 9572.15(6920.16-12670.25) | 143.74 | 24.49(18.21-31.50) | 32.37(23.76-41.72) | 0.50(0.28-0.73) |
| Central African Republic | 105.22(78.58-135.98) | 212.50(158.88-272.92) | 101.96 | 13.32(9.85-17.26) | 13.83(10.08-17.88) | 0.13(0.11-0.15) |
| Chad | 186.85(140.77-241.53) | 424.07(320.86-542.50) | 126.96 | 11.40(8.43-14.83) | 12.43(9.19-16.10) | 0.29(0.28-0.31) |
| Chile | 1631.97(1230.27-2101.97) | 4512.33(3294.38-5875.98) | 176.50 | 28.51(21.35-36.82) | 36.35(26.86-46.87) | 0.87(0.77-0.96) |
| China | 36796.74(26896.09-47796.72) | 111360.38  (81141.44-146693.30) | 202.64 | 7.69(5.64-9.99) | 10.48(7.73-13.66) | 1.29(1.14-1.44) |
| Colombia | 1198.76(899.84-1531.60) | 3683.31(2662.56-4854.22) | 207.26 | 11.23(8.36-14.62) | 13.04(9.47-17.03) | 0.51(0.48-0.53) |
| Comoros | 18.10(13.63-23.36) | 43.76(32.45-56.70) | 141.82 | 14.26(10.61-18.58) | 15.17(11.14-19.84) | 0.22(0.19-0.25) |
| Congo | 106.19(78.83-135.44) | 307.27(233.28-390.00) | 189.36 | 15.21(11.23-19.42) | 17.31(12.81-22.10) | 0.43(0.42-0.44) |
| Cook Islands | 0.98(0.73-1.27) | 2.24(1.64-2.94) | 127.66 | 14.66(10.72-18.98) | 18.64(13.74-24.30) | 0.67(0.51-0.84) |
| Costa Rica | 118.14(88.72-150.59) | 392.18(289.02-513.78) | 231.96 | 11.98(8.74-15.45) | 13.92(10.28-18.13) | 0.49(0.46-0.52) |
| C么te d'Ivoire | 349.52(264.37-450.98) | 1028.82(786.73-1316.08) | 194.35 | 18.47(13.84-23.96) | 21.36(16.04-27.68) | 0.34(0.32-0.36) |
| Croatia | 651.15(477.51-860.47) | 756.98(550.24-1000.90) | 16.25 | 13.29(9.85-17.27) | 15.62(11.53-20.12) | 0.57(0.53-0.62) |
| Cuba | 681.84(508.24-879.13) | 1408.52(1030.99-1836.08) | 106.58 | 31.56(23.80-40.51) | 38.56(28.50-49.65) | 0.62(0.57-0.67) |
| Cyprus | 135.78(100.39-175.38) | 360.63(262.94-468.73) | 165.60 | 18.94(14.08-24.60) | 21.93(16.35-28.71) | 0.74(0.68-0.81) |
| Czechia | 1306.82(956.53-1708.98) | 1859.99(1349.44-2441.96) | 42.33 | 13.41(9.91-17.40) | 14.94(11.15-19.53) | 0.51(0.47-0.54) |
| Democratic People's Republic of Korea | 880.47(638.81-1159.14) | 1460.06(1042.19-1908.85) | 65.83 | 8.15(6.03-10.68) | 8.30(6.03-10.78) | 0.06(0.04-0.08) |
| Democratic Republic of the Congo | 1549.59(1139.36-1991.16) | 3450.90(2605.81-4453.29) | 122.70 | 14.29(10.51-18.44) | 14.30(10.68-18.52) | -0.13(-0.20--0.07) |
| Denmark | 1660.23(1243.11-2176.27) | 1960.70(1417.30-2594.89) | 18.10 | 48.49(36.02-63.50) | 43.63(32.44-56.74) | -0.47(-0.72--0.22) |
| Djibouti | 13.08(9.95-16.77) | 62.67(46.63-80.66) | 379.18 | 13.38(9.92-17.26) | 15.96(11.55-20.76) | 0.68(0.64-0.72) |
| Dominica | 4.43(3.21-5.75) | 6.37(4.71-8.25) | 43.97 | 13.55(10.09-17.68) | 15.48(11.56-19.90) | 0.45(0.39-0.50) |
| Dominican Republic | 277.98(207.78-356.25) | 800.49(591.32-1045.38) | 187.97 | 12.77(9.40-16.50) | 15.80(11.80-20.63) | 0.84(0.79-0.89) |
| Ecuador | 450.51(340.95-579.51) | 1393.32(1022.14-1816.16) | 209.28 | 14.65(10.74-18.98) | 16.81(12.29-21.93) | 0.35(0.28-0.42) |
| Egypt | 1953.68(1466.23-2500.05) | 5224.77(3907.33-6751.92) | 167.43 | 10.99(8.11-14.12) | 13.71(10.13-17.63) | 0.57(0.49-0.65) |
| El Salvador | 191.09(144.12-245.66) | 446.22(328.14-580.49) | 133.51 | 11.23(8.26-14.55) | 13.46(9.85-17.53) | 0.62(0.52-0.72) |
| Equatorial Guinea | 17.87(13.02-22.95) | 67.77(51.97-86.52) | 279.19 | 13.52(9.86-17.40) | 18.82(13.96-24.29) | 1.36(1.26-1.47) |
| Eritrea | 94.70(71.25-122.49) | 262.54(197.65-335.14) | 177.22 | 12.54(9.24-16.22) | 13.39(9.84-17.27) | 0.21(0.14-0.29) |
| Estonia | 204.27(149.72-270.86) | 226.42(162.30-298.17) | 10.84 | 17.94(13.33-23.17) | 21.07(15.75-27.49) | 0.71(0.65-0.77) |
| Eswatini | 34.09(26.03-43.23) | 77.16(57.71-98.16) | 126.31 | 17.20(12.72-22.33) | 19.50(14.24-25.13) | 0.23(0.01-0.45) |
| Ethiopia | 1675.68(1253.91-2136.96) | 3702.03(2806.81-4727.19) | 120.93 | 13.64(9.91-17.62) | 14.30(10.64-18.47) | 0.18(0.16-0.20) |
| Fiji | 31.05(23.33-39.95) | 72.91(53.82-94.05) | 134.78 | 12.96(9.52-16.93) | 16.02(11.93-20.61) | 0.59(0.47-0.71) |
| Finland | 1249.09(910.17-1642.17) | 1896.66(1364.73-2520.17) | 51.84 | 35.94(26.65-46.86) | 42.19(31.53-54.94) | 0.56(0.50-0.63) |
| France | 13360.94(9811.56-17920.63) | 21757.25(15674.21-28384.86) | 62.84 | 36.03(26.92-47.41) | 42.00(31.22-53.91) | 0.64(0.36-0.91) |
| Gabon | 51.87(38.45-67.46) | 134.99(101.77-173.74) | 160.23 | 15.87(11.78-20.56) | 20.15(15.01-25.85) | 0.76(0.66-0.86) |
| Gambia | 25.96(19.74-33.26) | 86.58(65.71-110.00) | 233.52 | 12.76(9.35-16.55) | 14.83(11.06-19.17) | 0.50(0.48-0.52) |
| Georgia | 669.01(484.18-887.22) | 561.97(404.38-748.69) | -16.00 | 18.78(13.90-24.32) | 19.58(14.58-25.37) | 0.15(0.12-0.17) |
| Germany | 21046.27(15179.76-27694.62) | 29339.02(20924.83-38808.43) | 39.40 | 35.29(26.12-45.90) | 41.52(31.01-53.79) | 0.54(0.48-0.59) |
| Ghana | 516.23(383.06-655.69) | 1754.83(1325.97-2258.21) | 239.93 | 12.76(9.45-16.36) | 15.76(11.73-20.37) | 0.72(0.67-0.76) |
| Greece | 2364.87(1717.86-3105.21) | 3211.43(2329.13-4193.79) | 35.80 | 32.19(24.06-41.62) | 37.19(27.40-48.06) | 1.30(0.95-1.66) |
| Greenland | 5.18(3.90-6.61) | 11.60(8.50-15.33) | 123.79 | 25.83(19.31-33.04) | 32.60(24.31-42.03) | 0.81(0.74-0.89) |
| Grenada | 4.10(2.93-5.40) | 8.74(6.52-11.25) | 113.35 | 12.39(9.00-16.28) | 14.62(10.86-18.77) | 0.56(0.51-0.61) |
| Guam | 6.95(5.25-8.87) | 17.02(12.21-22.40) | 144.81 | 14.76(10.93-18.88) | 17.80(13.02-23.10) | 0.67(0.62-0.72) |
| Guatemala | 226.10(169.47-291.77) | 791.73(591.63-1020.95) | 250.17 | 9.99(7.39-12.88) | 11.78(8.71-15.40) | 0.57(0.53-0.61) |
| Guinea | 230.94(172.36-295.21) | 452.33(340.11-579.42) | 95.87 | 12.31(8.93-15.91) | 13.54(10.01-17.44) | 0.32(0.30-0.34) |
| Guinea-Bissau | 32.09(24.27-40.81) | 68.66(51.33-87.56) | 113.96 | 12.32(9.05-15.75) | 13.41(9.76-17.39) | 0.25(0.23-0.28) |
| Guyana | 29.57(22.11-37.86) | 56.29(41.50-73.15) | 90.33 | 12.86(9.42-16.66) | 14.88(10.97-19.32) | 0.50(0.45-0.55) |
| Haiti | 209.83(154.92-269.13) | 533.86(397.19-684.47) | 154.42 | 10.62(7.66-13.63) | 11.55(8.48-14.95) | 0.36(0.32-0.40) |
| Honduras | 127.08(94.90-163.12) | 453.43(340.57-579.70) | 256.81 | 10.38(7.63-13.44) | 12.26(9.06-15.91) | 0.59(0.52-0.66) |
| Hungary | 1502.78(1109.81-1955.44) | 1845.17(1341.80-2430.52) | 22.78 | 19.97(14.88-25.65) | 22.50(16.70-28.98) | 0.39(0.36-0.41) |
| Iceland | 57.39(45.16-70.94) | 109.31(79.83-143.44) | 90.48 | 44.34(34.89-54.58) | 46.26(34.65-59.62) | 0.02(-0.08-0.11) |
| India | 33219.55(25044.96-42875.91) | 95004.08(71580.06-122420.44) | 185.99 | 11.65(8.78-15.03) | 14.60(11.00-18.91) | 1.24(0.99-1.49) |
| Indonesia | 6202.60(4643.64-7965.26) | 15108.94(11243.83-19602.59) | 143.59 | 9.90(7.32-12.88) | 11.21(8.27-14.56) | 0.46(0.44-0.49) |
| Iran (Islamic Republic of) | 1499.91(1118.41-1925.10) | 5205.12(3896.00-6663.63) | 247.03 | 9.68(7.17-12.52) | 11.93(8.82-15.41) | 0.63(0.43-0.83) |
| Iraq | 523.82(398.10-669.86) | 1890.09(1419.18-2418.30) | 260.83 | 11.57(8.62-14.92) | 12.87(9.59-16.69) | 0.39(0.33-0.45) |
| Ireland | 666.19(496.72-867.85) | 1414.45(1052.68-1851.37) | 112.32 | 35.19(25.96-45.55) | 41.48(31.09-54.05) | 0.55(0.51-0.60) |
| Israel | 790.93(590.53-1028.29) | 2048.66(1504.18-2680.47) | 159.02 | 32.91(24.77-42.29) | 38.68(28.98-49.95) | 0.50(0.43-0.57) |
| Italy | 14346.49(10299.16-19018.33) | 20070.28(14545.50-26411.65) | 39.90 | 34.32(25.22-44.55) | 38.28(28.33-49.67) | 0.55(0.39-0.72) |
| Jamaica | 111.22(83.51-142.66) | 232.61(173.40-299.76) | 109.14 | 13.10(9.74-16.85) | 15.42(11.50-19.84) | 0.64(0.58-0.70) |
| Japan | 18660.08(13681.70-24408.73) | 25133.15(18063.35-33386.52) | 34.69 | 20.72(15.39-26.84) | 23.01(16.87-30.05) | 0.39(0.15-0.62) |
| Jordan | 93.67(70.45-120.72) | 586.59(442.52-753.91) | 526.25 | 10.97(8.13-14.36) | 14.22(10.54-18.21) | 0.94(0.89-0.98) |
| Kazakhstan | 1495.03(1110.77-1966.37) | 2352.00(1752.47-3056.31) | 57.32 | 19.05(14.24-24.88) | 21.64(16.10-27.94) | 0.37(0.34-0.40) |
| Kenya | 738.42(558.80-946.56) | 2230.92(1694.40-2832.71) | 202.12 | 14.50(10.73-18.75) | 15.11(11.24-19.53) | 0.13(0.12-0.15) |
| Kiribati | 2.92(2.19-3.76) | 6.74(4.97-8.81) | 130.59 | 12.17(8.89-15.83) | 14.04(10.35-18.26) | 0.36(0.16-0.56) |
| Kuwait | 47.35(35.38-61.04) | 317.73(235.53-418.91) | 571.02 | 12.83(9.70-16.50) | 16.06(11.91-20.70) | 0.87(0.84-0.90) |
| Kyrgyzstan | 281.93(206.39-370.25) | 526.31(388.58-690.73) | 86.68 | 16.08(11.86-20.98) | 17.56(13.22-22.83) | 0.22(0.18-0.25) |
| Lao People's Democratic Republic | 119.85(88.60-153.53) | 295.40(222.06-377.61) | 146.47 | 9.40(6.85-12.13) | 10.65(7.87-13.83) | 0.48(0.45-0.51) |
| Latvia | 361.84(265.09-468.35) | 350.79(249.55-462.75) | -3.05 | 18.14(13.42-23.41) | 20.98(15.53-26.85) | 0.59(0.55-0.64) |
| Lebanon | 137.14(101.28-176.69) | 389.58(290.50-500.33) | 184.07 | 10.55(7.79-13.63) | 13.67(10.12-17.75) | 0.65(0.29-1.01) |
| Lesotho | 80.19(59.21-103.06) | 134.85(100.78-171.95) | 68.16 | 13.78(10.13-17.85) | 16.22(12.10-20.76) | 0.56(0.51-0.61) |
| Liberia | 77.24(58.50-98.85) | 214.33(161.50-277.11) | 177.50 | 13.36(9.95-17.28) | 15.91(11.77-20.83) | 0.78(0.67-0.90) |
| Libya | 116.12(87.68-150.12) | 444.85(334.77-576.56) | 283.10 | 11.66(8.71-15.19) | 13.72(10.09-17.72) | 0.57(0.52-0.61) |
| Lithuania | 447.38(328.45-585.87) | 502.83(360.06-672.38) | 12.39 | 17.75(13.27-22.97) | 20.43(15.32-26.81) | 0.58(0.52-0.65) |
| Luxembourg | 93.65(67.82-122.88) | 183.25(134.30-238.89) | 95.67 | 35.47(26.33-45.78) | 41.32(30.24-53.53) | 0.53(0.49-0.57) |
| Madagascar | 404.39(301.76-516.58) | 1033.19(771.26-1332.03) | 155.49 | 13.19(9.78-16.97) | 13.52(10.07-17.52) | 0.11(0.08-0.13) |
| Malawi | 342.62(258.50-439.16) | 723.62(551.38-923.53) | 111.20 | 13.88(10.36-17.93) | 15.17(11.19-19.57) | 0.38(0.30-0.46) |
| Malaysia | 633.73(477.86-809.14) | 2041.57(1505.25-2654.30) | 222.15 | 11.16(8.22-14.45) | 13.54(9.94-17.62) | 0.72(0.70-0.74) |
| Maldives | 4.93(3.61-6.44) | 21.37(15.99-27.64) | 333.41 | 9.63(7.06-12.54) | 12.27(9.08-16.03) | 0.98(0.88-1.08) |
| Mali | 291.65(217.85-376.66) | 691.69(525.93-880.95) | 137.16 | 11.74(8.64-15.31) | 13.22(9.81-17.10) | 0.47(0.45-0.49) |
| Malta | 77.89(58.05-100.13) | 150.03(109.35-198.87) | 92.63 | 34.20(25.63-43.89) | 41.77(31.06-54.03) | 0.64(0.53-0.76) |
| Marshall Islands | 1.06(0.79-1.35) | 3.00(2.21-3.88) | 182.64 | 10.95(8.10-14.25) | 13.24(9.65-17.21) | 0.55(0.45-0.65) |
| Mauritania | 84.51(63.43-108.39) | 212.70(160.04-270.60) | 151.69 | 14.58(10.87-18.95) | 16.97(12.49-21.79) | 0.48(0.43-0.52) |
| Mauritius | 49.64(37.37-63.91) | 125.34(91.11-165.54) | 152.49 | 11.27(8.40-14.62) | 13.35(9.81-17.30) | 0.59(0.57-0.61) |
| Mexico | 3308.63(2486.88-4227.26) | 9570.33(7035.86-12382.88) | 189.25 | 12.76(9.43-16.58) | 14.25(10.56-18.49) | -0.12(-0.29-0.06) |
| Micronesia (Federated States of) | 3.44(2.58-4.40) | 6.70(4.89-8.74) | 94.76 | 12.83(9.42-16.64) | 14.80(10.93-19.13) | 0.36(0.18-0.55) |
| Monaco | 10.32(7.46-13.53) | 15.40(11.00-20.29) | 49.27 | 39.05(29.19-50.46) | 44.76(33.46-57.13) | 0.43(0.38-0.48) |
| Mongolia | 94.50(70.30-122.18) | 296.48(218.90-381.06) | 213.74 | 15.72(11.56-20.39) | 17.57(13.10-22.63) | 0.39(0.36-0.43) |
| Montenegro | 67.59(50.00-89.85) | 99.01(72.45-131.91) | 46.49 | 19.26(14.31-25.49) | 21.04(15.83-27.27) | 0.35(0.31-0.39) |
| Morocco | 774.31(584.44-994.46) | 2229.36(1640.73-2894.84) | 187.92 | 9.52(7.08-12.27) | 12.13(8.94-15.65) | 0.81(0.78-0.85) |
| Mozambique | 522.57(389.66-667.12) | 1083.25(818.81-1379.78) | 107.29 | 13.38(9.78-17.33) | 14.42(10.67-18.51) | 0.30(0.26-0.35) |
| Myanmar | 1315.22(971.69-1719.02) | 3113.04(2259.98-4069.36) | 136.69 | 9.33(6.80-12.24) | 10.60(7.78-13.92) | 0.52(0.47-0.57) |
| Namibia | 58.79(44.00-75.18) | 141.98(107.36-179.87) | 141.51 | 14.04(10.42-18.21) | 15.67(11.71-20.03) | 0.33(0.25-0.41) |
| Nauru | 0.34(0.25-0.44) | 0.53(0.40-0.68) | 56.51 | 13.24(9.66-17.09) | 15.39(11.39-19.69) | 0.34(0.26-0.41) |
| Nepal | 661.47(498.56-842.96) | 2035.54(1541.26-2621.32) | 207.73 | 11.06(8.36-14.19) | 14.85(11.20-19.11) | 1.13(1.05-1.20) |
| Netherlands | 3921.23(2928.47-5037.92) | 5774.49(4186.37-7701.87) | 47.26 | 41.89(31.32-53.04) | 41.79(31.05-54.26) | -0.06(-0.25-0.14) |
| New Zealand | 558.65(413.33-724.38) | 1297.44(940.06-1714.19) | 132.25 | 30.09(22.42-39.09) | 37.97(27.92-49.42) | 0.89(0.82-0.96) |
| Nicaragua | 101.81(76.55-130.41) | 355.77(267.03-457.20) | 249.43 | 10.75(7.90-13.96) | 12.69(9.44-16.43) | 0.55(0.45-0.64) |
| Niger | 207.40(155.91-267.74) | 644.30(485.30-827.88) | 210.66 | 11.80(8.77-15.44) | 12.52(9.25-16.20) | 0.23(0.21-0.25) |
| Nigeria | 3091.74(2319.75-3963.26) | 8279.06(6250.02-10602.04) | 167.78 | 13.71(10.16-17.90) | 13.39(9.84-17.32) | -0.32(-0.51--0.14) |
| Niue | 0.14(0.11-0.19) | 0.19(0.14-0.24) | 28.72 | 13.78(10.27-17.92) | 17.38(12.86-22.29) | 0.74(0.59-0.89) |
| North Macedonia | 191.57(142.47-250.73) | 326.95(240.44-432.42) | 70.67 | 17.86(13.36-22.95) | 20.55(15.42-26.81) | 0.49(0.48-0.50) |
| Northern Mariana Islands | 1.70(1.26-2.22) | 5.35(3.83-7.24) | 215.41 | 14.79(11.01-19.18) | 16.67(12.33-21.78) | 0.26(0.09-0.43) |
| Norway | 945.76(693.13-1236.85) | 1601.22(1172.76-2085.83) | 69.31 | 34.90(25.94-45.12) | 41.54(30.88-53.57) | 0.66(0.64-0.68) |
| Oman | 34.98(26.32-44.19) | 156.71(117.61-202.30) | 347.99 | 9.31(6.92-11.97) | 13.90(10.49-18.09) | 1.46(1.41-1.50) |
| Pakistan | 3974.21(3034.89-5079.41) | 11662.76(8811.61-14922.73) | 193.46 | 12.78(9.62-16.33) | 16.17(12.24-20.81) | 0.92(0.87-0.97) |
| Palau | 0.80(0.60-1.02) | 2.19(1.59-2.89) | 172.68 | 14.25(10.66-18.34) | 17.44(12.87-22.55) | 0.56(0.39-0.73) |
| Palestine | 55.25(40.99-70.90) | 184.79(137.97-237.36) | 234.45 | 10.34(7.67-13.40) | 11.91(8.80-15.29) | 0.39(0.32-0.46) |
| Panama | 80.70(59.72-103.54) | 272.62(201.46-351.49) | 237.83 | 10.01(7.28-12.95) | 12.89(9.45-16.62) | 0.78(0.73-0.82) |
| Papua New Guinea | 118.41(87.44-151.90) | 352.93(262.76-451.24) | 198.07 | 10.43(7.68-13.61) | 11.52(8.47-14.84) | 0.24(0.18-0.31) |
| Paraguay | 160.94(120.15-204.57) | 453.06(328.80-585.24) | 181.51 | 12.91(9.55-16.55) | 14.80(10.69-19.15) | 0.46(0.44-0.48) |
| Peru | 859.66(636.96-1097.50) | 2579.50(1924.60-3364.64) | 200.06 | 12.50(9.26-16.19) | 15.04(11.14-19.65) | 0.69(0.66-0.73) |
| Philippines | 1958.52(1461.78-2521.63) | 5510.38(4092.89-7128.67) | 181.36 | 10.46(7.72-13.60) | 11.60(8.56-15.08) | 0.35(0.33-0.37) |
| Poland | 4213.16(3111.66-5518.62) | 6883.98(4953.87-9036.39) | 63.39 | 17.94(13.43-23.20) | 22.29(16.56-28.99) | 0.79(0.76-0.81) |
| Portugal | 2228.05(1619.96-2964.77) | 3548.78(2529.05-4773.29) | 59.28 | 31.82(23.53-41.46) | 37.81(28.10-49.96) | 0.02(-0.18-0.22) |
| Puerto Rico | 329.99(242.60-429.46) | 583.62(429.54-765.42) | 76.86 | 17.51(12.82-22.77) | 20.43(15.12-26.36) | 0.62(0.57-0.68) |
| Qatar | 10.05(7.19-13.23) | 94.83(69.66-125.32) | 843.84 | 13.86(10.36-17.84) | 16.73(12.53-21.44) | 0.61(0.53-0.68) |
| Republic of Korea | 4609.10(3408.39-6030.74) | 11678.67(8382.70-15696.37) | 153.38 | 23.68(17.52-31.18) | 26.10(19.18-34.16) | 0.40(0.25-0.54) |
| Republic of Moldova | 427.43(311.23-566.25) | 610.88(445.05-812.25) | 42.92 | 16.13(11.77-21.06) | 20.16(15.10-26.01) | 0.91(0.79-1.02) |
| Romania | 2687.05(1964.60-3516.06) | 3375.75(2466.39-4457.48) | 25.63 | 17.86(13.18-23.20) | 21.42(16.05-27.92) | 0.65(0.62-0.67) |
| Russian Federation | 17090.72(12471.09-22562.14) | 24183.60(17449.76-31836.78) | 41.50 | 16.08(11.94-20.87) | 19.61(14.57-25.31) | 0.79(0.74-0.84) |
| Rwanda | 260.52(197.72-337.41) | 630.67(479.33-807.71) | 142.08 | 13.72(10.29-17.87) | 14.82(11.07-19.02) | 0.30(0.28-0.32) |
| Saint Kitts and Nevis | 2.39(1.74-3.16) | 6.59(4.81-8.62) | 175.85 | 14.33(10.57-18.53) | 16.85(12.41-21.59) | 0.56(0.52-0.60) |
| Saint Lucia | 6.18(4.57-7.99) | 17.89(13.46-23.28) | 189.33 | 13.16(9.77-17.10) | 15.71(11.77-20.30) | 0.55(0.50-0.61) |
| Saint Vincent and the Grenadines | 4.65(3.41-5.99) | 10.33(7.55-13.43) | 122.29 | 12.65(9.30-16.28) | 15.44(11.36-20.11) | 0.75(0.70-0.80) |
| Samoa | 6.85(5.13-8.76) | 12.71(9.42-16.51) | 85.66 | 14.19(10.61-18.23) | 15.68(11.66-20.29) | 0.18(0.03-0.32) |
| San Marino | 5.63(4.10-7.34) | 10.78(7.95-14.00) | 91.31 | 37.18(27.55-47.68) | 42.47(31.74-54.74) | 0.48(0.41-0.54) |
| Sao Tome and Principe | 4.82(3.57-6.26) | 10.83(8.16-13.77) | 124.77 | 13.61(10.13-17.63) | 15.90(11.71-20.46) | 0.55(0.53-0.56) |
| Saudi Arabia | 344.02(258.32-439.21) | 1921.77(1423.89-2507.52) | 458.62 | 10.88(8.04-14.03) | 15.21(11.43-19.63) | 1.13(1.12-1.15) |
| Senegal | 259.84(196.21-332.19) | 685.88(517.48-872.65) | 163.97 | 13.55(10.02-17.46) | 14.67(10.82-18.86) | 0.23(0.20-0.26) |
| Serbia | 1182.37(863.81-1567.28) | 1468.46(1063.40-1940.05) | 24.20 | 18.47(13.89-24.01) | 21.63(16.21-27.84) | 0.60(0.57-0.63) |
| Seychelles | 3.49(2.59-4.56) | 8.40(6.09-11.01) | 140.29 | 12.18(9.01-15.92) | 13.74(10.08-17.80) | 0.35(0.31-0.39) |
| Sierra Leone | 129.41(97.42-165.48) | 302.24(229.50-388.81) | 133.55 | 12.29(9.01-15.90) | 13.77(10.18-17.90) | 0.38(0.34-0.43) |
| Singapore | 296.80(220.96-382.58) | 1046.94(768.63-1361.83) | 252.74 | 21.55(15.78-27.94) | 25.79(19.12-33.39) | 0.67(0.61-0.73) |
| Slovakia | 599.77(440.36-789.29) | 947.65(688.98-1252.04) | 58.00 | 19.13(14.09-24.90) | 21.86(16.12-28.49) | 0.38(0.35-0.42) |
| Slovenia | 244.99(177.82-322.15) | 371.04(269.68-491.35) | 51.45 | 18.66(13.70-24.28) | 22.06(16.40-28.47) | 0.60(0.56-0.64) |
| Solomon Islands | 8.91(6.61-11.55) | 26.10(19.49-33.38) | 192.89 | 11.08(8.08-14.36) | 12.79(9.47-16.57) | 0.39(0.22-0.56) |
| Somalia | 246.14(183.07-315.68) | 643.87(486.32-827.59) | 161.59 | 13.35(9.94-17.29) | 13.40(9.86-17.51) | 0.07(0.03-0.11) |
| South Africa | 1973.71(1466.18-2576.05) | 4551.86(3352.26-5991.09) | 130.62 | 15.24(11.22-20.04) | 16.28(11.94-21.46) | 0.26(0.21-0.31) |
| South Sudan | 168.54(127.35-213.15) | 367.34(275.16-474.66) | 117.96 | 13.80(10.13-17.94) | 14.48(10.70-18.72) | 0.18(0.14-0.23) |
| Spain | 9718.72(7013.95-12890.37) | 16391.00(11841.66-21339.47) | 68.65 | 37.62(28.02-48.77) | 43.46(31.89-56.15) | 0.39(0.23-0.55) |
| Sri Lanka | 649.52(489.61-837.67) | 1609.34(1164.61-2131.13) | 147.78 | 10.07(7.44-13.19) | 11.26(8.30-14.70) | 0.43(0.42-0.45) |
| Sudan | 437.12(326.88-560.32) | 1344.92(1015.29-1704.24) | 207.68 | 8.30(6.16-10.78) | 11.58(8.54-14.86) | 1.17(1.06-1.27) |
| Suriname | 20.07(14.62-25.85) | 54.82(40.36-72.03) | 173.14 | 13.65(9.93-17.56) | 16.23(11.98-21.13) | 0.64(0.61-0.67) |
| Sweden | 1292.95(974.34-1663.29) | 2749.46(1942.00-3745.17) | 112.65 | 20.89(15.77-26.58) | 36.63(26.37-49.01) | 2.39(1.84-2.94) |
| Switzerland | 1590.43(1153.11-2065.50) | 2613.26(1860.45-3474.86) | 64.31 | 34.28(25.30-44.26) | 38.03(27.73-49.15) | 0.37(0.36-0.39) |
| Syrian Arab Republic | 320.47(237.91-411.38) | 928.37(690.98-1208.77) | 189.69 | 10.45(7.68-13.47) | 12.39(9.14-15.91) | 0.47(0.41-0.53) |
| Taiwan (Province of China) | 824.59(616.52-1064.10) | 2200.84(1570.46-2918.63) | 166.90 | 9.63(7.08-12.46) | 11.29(8.26-14.78) | 0.71(0.63-0.79) |
| Tajikistan | 229.87(169.25-297.87) | 553.62(411.68-723.90) | 140.85 | 14.54(10.74-18.72) | 15.37(11.32-19.93) | 0.15(0.10-0.19) |
| Thailand | 2276.62(1694.38-2954.26) | 7230.42(5334.27-9496.61) | 217.59 | 10.06(7.42-13.15) | 13.05(9.66-16.93) | 0.94(0.91-0.97) |
| Timor-Leste | 18.16(13.58-23.43) | 42.00(31.16-53.84) | 131.28 | 9.21(6.80-11.98) | 9.47(6.96-12.11) | 0.12(0.09-0.15) |
| Togo | 106.60(80.32-135.89) | 369.43(279.52-471.69) | 246.54 | 12.41(9.08-16.04) | 13.95(10.27-18.11) | 0.36(0.32-0.39) |
| Tokelau | 0.09(0.06-0.11) | 0.11(0.08-0.14) | 25.10 | 12.46(9.14-16.07) | 15.65(11.69-20.36) | 0.79(0.67-0.91) |
| Tonga | 4.39(3.25-5.67) | 6.77(5.04-8.75) | 54.26 | 13.53(10.04-17.61) | 15.82(11.66-20.54) | 0.30(0.06-0.55) |
| Trinidad and Tobago | 65.69(49.05-83.99) | 154.92(114.60-203.54) | 135.83 | 14.40(10.71-18.51) | 16.54(12.37-21.53) | 0.58(0.52-0.64) |
| Tunisia | 285.12(211.14-365.81) | 864.06(629.33-1110.05) | 203.06 | 10.11(7.50-13.03) | 12.45(9.07-15.94) | 0.70(0.65-0.74) |
| Turkey | 2300.44(1712.41-2991.48) | 6582.17(4912.02-8529.02) | 186.13 | 10.84(8.07-14.10) | 13.65(10.23-17.65) | 0.74(0.69-0.79) |
| Turkmenistan | 196.12(147.62-252.32) | 494.26(361.95-645.89) | 152.02 | 16.64(12.46-21.36) | 19.63(14.57-25.37) | 0.54(0.52-0.56) |
| Tuvalu | 0.51(0.38-0.66) | 0.82(0.60-1.06) | 59.37 | 12.08(8.93-15.65) | 14.40(10.58-18.53) | 0.54(0.40-0.68) |
| Uganda | 528.71(393.62-680.17) | 1416.40(1074.31-1807.71) | 167.90 | 13.47(9.86-17.53) | 14.46(10.69-18.65) | 0.31(0.28-0.34) |
| Ukraine | 6728.58(4857.51-8837.03) | 7166.74(5189.08-9487.45) | 6.51 | 16.73(12.39-21.59) | 18.32(13.46-23.72) | 0.38(0.34-0.43) |
| United Arab Emirates | 30.14(21.98-38.86) | 360.08(256.22-480.50) | 1094.55 | 11.32(8.48-14.59) | 14.95(11.22-19.20) | 1.02(0.97-1.07) |
| United Kingdom | 14528.93(10557.60-18867.60) | 22249.78(16255.34-29194.61) | 53.14 | 37.37(27.71-48.09) | 44.58(33.20-57.42) | 0.65(0.58-0.71) |
| United Republic of Tanzania | 960.31(719.83-1229.14) | 2552.84(1918.30-3244.61) | 165.84 | 14.40(10.72-18.52) | 16.07(11.86-20.67) | 0.40(0.37-0.43) |
| United States of America | 65325.33(48988.35-84421.52) | 140028.91  (105284.21-177860.34) | 114.36 | 44.80(33.25-58.05) | 56.82(44.02-70.72) | 0.60(0.51-0.70) |
| United States Virgin Islands | 9.20(6.87-11.89) | 16.81(12.08-22.22) | 82.60 | 17.20(12.65-22.17) | 19.37(14.22-25.09) | 0.41(0.36-0.45) |
| Uruguay | 524.53(378.00-698.05) | 828.11(593.97-1077.66) | 57.88 | 27.46(20.13-35.72) | 35.14(25.87-45.48) | 0.92(0.82-1.02) |
| Uzbekistan | 1061.82(786.29-1379.98) | 2930.57(2203.28-3856.10) | 175.99 | 15.89(11.82-20.66) | 18.81(14.22-24.65) | 0.55(0.51-0.60) |
| Vanuatu | 4.36(3.25-5.59) | 12.98(9.66-16.60) | 197.90 | 11.66(8.62-15.07) | 13.27(9.75-17.08) | 0.44(0.41-0.47) |
| Venezuela (Bolivarian Republic of) | 698.22(524.04-902.49) | 2195.95(1605.18-2843.90) | 214.51 | 11.87(8.66-15.48) | 13.53(9.94-17.52) | 0.44(0.42-0.47) |
| Viet Nam | 2158.44(1608.47-2793.57) | 5732.95(4146.60-7584.58) | 165.61 | 9.07(6.78-11.69) | 9.95(7.27-13.03) | 0.38(0.34-0.42) |
| Yemen | 239.30(176.70-305.22) | 823.31(614.76-1044.95) | 244.05 | 7.84(5.74-10.11) | 9.35(6.93-11.96) | 0.69(0.64-0.73) |
| Zambia | 258.35(193.76-330.49) | 693.15(525.34-892.68) | 168.30 | 14.47(10.59-18.78) | 15.49(11.46-20.10) | 0.20(0.17-0.23) |
| Zimbabwe | 359.28(271.85-456.01) | 716.52(541.05-914.28) | 99.43 | 14.44(10.79-18.55) | 14.52(10.62-18.68) | -0.10(-0.21-0.00) |

S Table5 The incident cases and age-standardized incidence rate of national hip osteoarthritis in male in 1990 and 2019, and its temporal trends from 1990 to 2019

| **Nation** | **Incident Cases No. (95% UI)** | | **Change in absolute number (%)** | **ASIR No.(95% UI)** | | **1990-2019 EAPC No. (95%CI)** |
| --- | --- | --- | --- | --- | --- | --- |
|  | **1990** | **2019** |  | **1990** | **2019** |  |
| Afghanistan | 360.37(259.75-472.79) | 976.13(716.16-1293.80) | 170.87 | 9.27(6.85-11.96) | 10.86(8.09-13.97) | 0.70(0.58-0.82) |
| Albania | 239.61(178.37-311.14) | 410.37(292.05-549.05) | 71.26 | 18.94(14.15-24.39) | 21.62(16.10-28.14) | 0.50(0.48-0.51) |
| Algeria | 789.09(592.72-1013.27) | 2917.48(2201.20-3756.91) | 269.73 | 11.28(8.31-14.58) | 14.40(10.74-18.63) | 0.83(0.79-0.86) |
| American Samoa | 2.96(2.20-3.85) | 5.80(4.24-7.52) | 95.71 | 19.23(14.11-25.28) | 21.46(15.88-27.52) | 0.18(-0.07-0.43) |
| Andorra | 9.78(7.33-12.48) | 25.61(18.64-33.61) | 161.76 | 30.41(22.40-38.94) | 35.94(26.48-46.66) | 0.55(0.46-0.64) |
| Angola | 387.70(291.77-492.95) | 1278.48(959.55-1648.18) | 229.76 | 14.54(10.79-18.70) | 17.68(12.85-23.01) | 0.70(0.67-0.73) |
| Antigua and Barbuda | 3.32(2.48-4.26) | 9.63(7.00-12.55) | 190.48 | 14.99(11.10-19.45) | 17.87(13.04-23.17) | 0.60(0.57-0.63) |
| Argentina | 3770.45(2797.55-4885.54) | 8006.82(5924.36-10309.04) | 112.36 | 24.47(18.16-31.69) | 33.62(24.88-43.15) | 1.02(0.90-1.14) |
| Armenia | 274.50(198.68-364.17) | 415.42(303.03-549.61) | 51.34 | 18.89(14.05-24.37) | 22.59(16.93-29.28) | 0.63(0.61-0.65) |
| Australia | 2569.93(1933.33-3357.55) | 6180.29(4579.21-8081.58) | 140.48 | 28.06(20.97-36.63) | 37.87(28.12-48.84) | 0.98(0.89-1.08) |
| Austria | 1349.01(997.34-1748.45) | 2309.88(1691.80-3024.81) | 71.23 | 29.89(22.16-38.55) | 34.58(25.83-44.83) | 0.47(0.44-0.50) |
| Azerbaijan | 513.36(373.33-673.10) | 1336.65(981.14-1747.24) | 160.37 | 19.50(14.38-25.03) | 23.39(17.40-30.19) | 0.65(0.59-0.72) |
| Bahamas | 14.07(10.59-18.09) | 40.77(30.20-53.39) | 189.72 | 16.85(12.34-21.77) | 19.20(14.14-24.81) | 0.43(0.37-0.49) |
| Bahrain | 26.19(19.34-33.84) | 209.14(153.83-273.12) | 698.61 | 13.95(10.44-17.94) | 16.66(12.33-21.64) | 0.54(0.51-0.57) |
| Bangladesh | 2259.49(1671.85-2859.30) | 6535.02(4871.10-8373.35) | 189.23 | 7.26(5.39-9.33) | 8.96(6.69-11.48) | 0.82(0.78-0.86) |
| Barbados | 17.70(13.23-22.59) | 40.62(29.64-53.43) | 129.51 | 16.64(12.44-21.32) | 19.34(14.10-25.19) | 0.52(0.48-0.57) |
| Belarus | 1198.60(871.19-1595.13) | 1588.02(1156.60-2092.53) | 32.49 | 21.65(16.24-28.23) | 24.52(18.38-31.85) | 0.38(0.35-0.42) |
| Belgium | 1854.24(1341.19-2428.08) | 2857.81(2081.77-3730.21) | 54.12 | 29.41(21.47-38.03) | 34.07(25.21-43.88) | 0.45(0.42-0.49) |
| Belize | 8.02(6.02-10.30) | 33.28(24.63-43.02) | 314.99 | 15.81(11.76-20.63) | 20.02(14.73-26.01) | 0.80(0.62-0.99) |
| Benin | 146.87(110.49-188.73) | 516.49(392.65-659.00) | 251.67 | 13.74(10.08-17.80) | 17.07(12.66-21.92) | 0.74(0.64-0.85) |
| Bermuda | 6.05(4.54-7.74) | 11.02(8.12-14.71) | 82.24 | 19.38(14.29-24.84) | 21.06(15.85-27.44) | 0.24(0.18-0.31) |
| Bhutan | 13.59(10.15-17.51) | 37.09(27.97-47.68) | 172.98 | 8.10(6.05-10.63) | 10.72(8.07-13.88) | 1.05(0.99-1.11) |
| Bolivia (Plurinational State of) | 226.78(168.81-289.61) | 785.20(589.48-1017.15) | 246.24 | 12.51(9.15-16.25) | 16.18(12.10-20.94) | 0.91(0.88-0.95) |
| Bosnia and Herzegovina | 438.69(318.21-579.08) | 566.28(409.54-751.51) | 29.09 | 19.17(13.98-24.85) | 21.98(16.36-28.73) | 0.49(0.42-0.57) |
| Botswana | 63.90(47.30-83.72) | 235.77(177.78-299.74) | 268.99 | 20.05(14.76-26.35) | 27.02(20.04-34.50) | 0.98(0.90-1.06) |
| Brazil | 7610.91(5683.89-9772.32) | 22474.64(16636.90-29065.51) | 195.29 | 15.11(11.21-19.55) | 19.01(14.06-24.57) | 0.85(0.80-0.89) |
| Brunei Darussalam | 16.26(12.30-20.94) | 56.22(42.38-72.79) | 245.73 | 20.59(15.36-26.72) | 24.78(18.62-32.09) | 0.60(0.53-0.68) |
| Bulgaria | 1335.25(980.14-1754.71) | 1285.69(941.33-1689.77) | -3.71 | 22.74(16.88-29.53) | 23.83(17.71-30.80) | 0.07(0.03-0.11) |
| Burkina Faso | 310.44(229.16-401.56) | 830.28(629.36-1070.86) | 167.46 | 12.97(9.60-16.74) | 15.36(11.48-19.88) | 0.55(0.50-0.59) |
| Burundi | 180.77(137.79-229.32) | 502.86(376.24-649.15) | 178.17 | 14.45(10.70-18.56) | 15.60(11.36-20.25) | 0.27(0.25-0.30) |
| Cabo Verde | 11.87(8.63-15.54) | 40.96(31.20-53.19) | 245.16 | 14.08(10.37-18.21) | 17.40(13.05-22.53) | 0.71(0.66-0.75) |
| Cambodia | 239.87(177.37-305.94) | 718.34(531.97-931.60) | 199.46 | 10.16(7.49-13.09) | 11.39(8.38-14.79) | 0.40(0.37-0.43) |
| Cameroon | 455.48(341.66-589.23) | 1480.38(1100.71-1915.39) | 225.02 | 16.68(12.39-21.69) | 18.88(13.91-24.52) | 0.33(0.30-0.36) |
| Canada | 3254.49(2452.44-4169.92) | 7929.38(5770.14-10408.01) | 143.64 | 21.76(16.30-27.98) | 29.58(22.11-37.96) | 0.54(0.28-0.80) |
| Central African Republic | 103.49(77.22-133.96) | 233.00(173.27-305.91) | 125.15 | 14.54(10.81-18.93) | 15.99(11.96-20.81) | 0.35(0.31-0.38) |
| Chad | 180.07(134.53-231.68) | 503.99(379.20-654.51) | 179.88 | 12.25(9.03-15.89) | 13.59(10.00-17.69) | 0.30(0.26-0.34) |
| Chile | 1355.42(1024.14-1746.21) | 3917.72(2889.68-5102.02) | 189.04 | 26.07(19.43-33.72) | 35.31(26.37-45.88) | 0.92(0.80-1.04) |
| China | 44743.09(32879.08-57917.67) | 131749.08(96482.85-173132.15) | 194.46 | 8.86(6.54-11.56) | 12.36(9.05-16.13) | 1.23(1.14-1.32) |
| Colombia | 1305.71(979.92-1663.50) | 3708.70(2749.25-4790.00) | 184.04 | 12.64(9.34-16.31) | 15.02(11.15-19.37) | 0.56(0.54-0.58) |
| Comoros | 18.89(14.11-24.23) | 49.05(36.91-63.47) | 159.65 | 15.60(11.50-20.09) | 18.13(13.37-23.39) | 0.54(0.51-0.57) |
| Congo | 98.64(74.56-128.27) | 364.41(270.61-479.01) | 269.44 | 16.65(12.43-21.84) | 19.93(14.87-26.02) | 0.64(0.63-0.65) |
| Cook Islands | 1.35(1.00-1.76) | 2.56(1.84-3.43) | 89.76 | 17.58(13.05-22.66) | 21.64(15.79-28.00) | 0.54(0.38-0.71) |
| Costa Rica | 128.85(98.30-165.15) | 398.21(286.89-517.30) | 209.05 | 13.45(9.99-17.37) | 15.93(11.54-20.62) | 0.57(0.54-0.60) |
| C么te d'Ivoire | 441.69(333.47-570.60) | 1311.64(978.53-1687.95) | 196.96 | 21.48(15.84-27.92) | 23.79(17.80-30.96) | 0.36(0.30-0.41) |
| Croatia | 648.72(471.44-855.73) | 775.84(563.69-1023.26) | 19.60 | 14.89(11.16-19.17) | 18.02(13.23-23.53) | 0.40(0.35-0.44) |
| Cuba | 751.59(565.69-964.49) | 1536.11(1124.70-2017.44) | 104.38 | 27.21(20.00-34.48) | 32.85(23.86-42.51) | 0.73(0.68-0.77) |
| Cyprus | 107.59(80.89-137.38) | 285.17(209.10-371.37) | 165.05 | 22.01(16.34-28.76) | 24.35(17.90-31.23) | 0.68(0.62-0.75) |
| Czechia | 1309.73(965.99-1714.55) | 1970.55(1453.05-2527.80) | 50.45 | 14.42(10.64-18.69) | 16.38(12.19-21.44) | 0.31(0.28-0.34) |
| Democratic People's Republic of Korea | 788.02(580.23-1051.34) | 1511.94(1101.20-1977.55) | 91.86 | 9.28(6.86-12.15) | 9.40(6.89-12.19) | 0.02(0.00-0.05) |
| Democratic Republic of the Congo | 1439.48(1091.53-1853.79) | 3797.14(2829.84-4864.78) | 163.79 | 15.59(11.49-20.10) | 16.46(12.10-21.40) | 0.08(0.03-0.14) |
| Denmark | 1295.17(983.08-1639.69) | 1575.32(1147.84-2053.63) | 21.63 | 40.77(30.89-51.35) | 36.78(27.50-47.20) | -0.50(-0.75--0.24) |
| Djibouti | 16.26(12.15-20.90) | 90.17(67.74-115.82) | 454.64 | 14.73(11.01-19.03) | 19.09(14.37-24.53) | 1.01(0.96-1.06) |
| Dominica | 4.01(3.01-5.16) | 7.99(5.83-10.53) | 99.55 | 15.09(11.28-19.64) | 17.90(13.20-23.28) | 0.56(0.50-0.63) |
| Dominican Republic | 301.45(224.97-385.51) | 918.73(682.08-1178.50) | 204.77 | 14.28(10.56-18.38) | 18.37(13.50-23.78) | 0.94(0.90-0.99) |
| Ecuador | 475.48(356.77-612.23) | 1523.92(1130.44-1950.77) | 220.50 | 15.75(11.63-20.44) | 19.39(14.34-24.87) | 0.63(0.56-0.70) |
| Egypt | 2275.89(1727.16-2884.07) | 6413.03(4869.08-8256.47) | 181.78 | 12.49(9.41-16.00) | 15.13(11.28-19.47) | 0.42(0.31-0.53) |
| El Salvador | 192.14(144.18-245.40) | 378.90(277.84-491.16) | 97.20 | 12.68(9.45-16.44) | 15.45(11.25-20.11) | 0.67(0.57-0.77) |
| Equatorial Guinea | 15.98(11.87-20.68) | 63.49(47.65-82.24) | 297.34 | 14.71(10.90-18.97) | 21.75(16.02-28.17) | 1.61(1.50-1.72) |
| Eritrea | 87.56(65.42-113.37) | 285.46(214.82-374.86) | 226.01 | 13.69(10.05-17.79) | 15.98(11.80-20.58) | 0.52(0.48-0.57) |
| Estonia | 191.52(141.15-249.40) | 230.54(170.48-299.28) | 20.37 | 22.53(16.63-29.18) | 25.52(19.04-32.77) | 0.50(0.47-0.52) |
| Eswatini | 42.45(31.56-55.48) | 95.76(72.59-124.06) | 125.60 | 24.10(17.95-31.33) | 29.24(21.92-37.48) | 0.48(0.29-0.66) |
| Ethiopia | 1972.35(1488.70-2516.49) | 4777.60(3665.22-6125.44) | 142.23 | 15.17(11.30-19.60) | 17.94(13.26-23.28) | 0.62(0.55-0.69) |
| Fiji | 37.37(27.99-47.94) | 84.76(61.71-111.59) | 126.82 | 15.48(11.46-19.96) | 18.60(13.75-24.24) | 0.48(0.36-0.61) |
| Finland | 906.26(676.44-1174.43) | 1462.40(1041.67-1948.14) | 61.37 | 30.17(22.21-39.14) | 35.06(25.86-45.31) | 0.51(0.45-0.57) |
| France | 10042.54(7407.93-13006.45) | 16428.90(12062.35-21613.52) | 63.59 | 30.26(22.50-39.09) | 35.18(26.12-45.84) | 0.56(0.32-0.80) |
| Gabon | 51.12(37.42-65.43) | 151.98(112.92-197.91) | 197.29 | 17.30(12.59-22.23) | 23.23(17.19-30.30) | 0.98(0.86-1.11) |
| Gambia | 33.78(25.60-43.21) | 95.11(71.27-121.11) | 181.53 | 13.78(10.28-17.91) | 16.23(11.94-20.94) | 0.51(0.45-0.57) |
| Georgia | 637.67(460.73-840.33) | 546.96(404.95-714.38) | -14.23 | 22.19(16.27-28.82) | 23.13(17.26-29.88) | 0.11(0.09-0.13) |
| Germany | 15558.82(11326.66-20145.13) | 23449.41(17037.28-30941.03) | 50.71 | 30.48(22.33-39.18) | 35.31(26.52-45.54) | 0.46(0.41-0.51) |
| Ghana | 538.81(408.73-694.75) | 1646.07(1235.70-2117.35) | 205.50 | 13.72(10.10-17.74) | 17.28(12.77-22.45) | 0.72(0.64-0.81) |
| Greece | 1815.21(1348.66-2362.44) | 2434.95(1782.53-3170.98) | 34.14 | 27.36(20.47-35.34) | 31.06(23.22-40.00) | 1.53(1.06-1.99) |
| Greenland | 6.10(4.53-7.97) | 12.37(8.97-16.18) | 102.67 | 23.00(17.08-29.64) | 29.63(22.25-38.02) | 0.90(0.81-0.99) |
| Grenada | 3.84(2.81-4.99) | 10.69(7.86-14.15) | 178.74 | 13.84(10.12-17.99) | 16.98(12.64-22.32) | 0.68(0.61-0.75) |
| Guam | 9.50(7.05-12.21) | 20.97(15.25-27.30) | 120.68 | 17.64(12.87-22.95) | 20.71(15.12-26.51) | 0.54(0.47-0.61) |
| Guatemala | 248.94(185.78-323.46) | 747.78(557.07-957.02) | 200.39 | 11.24(8.24-14.64) | 13.60(10.03-17.63) | 0.64(0.59-0.68) |
| Guinea | 241.69(178.82-309.93) | 476.86(354.43-612.85) | 97.30 | 13.30(9.84-17.15) | 14.78(10.93-19.01) | 0.32(0.29-0.35) |
| Guinea-Bissau | 30.89(22.97-39.95) | 66.27(50.18-85.33) | 114.52 | 13.25(9.82-17.11) | 14.61(10.71-19.12) | 0.26(0.21-0.32) |
| Guyana | 32.05(24.16-41.44) | 61.31(45.73-79.36) | 91.31 | 14.41(10.64-18.91) | 17.19(12.80-22.14) | 0.60(0.53-0.66) |
| Haiti | 228.29(168.63-294.08) | 545.32(410.32-696.51) | 138.87 | 11.82(8.71-15.31) | 13.27(9.88-17.05) | 0.46(0.44-0.49) |
| Honduras | 138.56(103.05-176.61) | 463.57(345.84-590.65) | 234.56 | 11.70(8.58-15.15) | 14.11(10.41-18.16) | 0.67(0.60-0.73) |
| Hungary | 1461.17(1079.35-1911.28) | 1779.79(1305.99-2332.28) | 21.81 | 23.30(17.28-30.30) | 25.02(18.56-32.41) | 0.18(0.14-0.22) |
| Iceland | 52.40(40.76-65.01) | 92.65(68.27-121.52) | 76.80 | 41.68(32.37-51.77) | 39.85(29.44-52.03) | -0.43(-0.61--0.26) |
| India | 24404.83(18033.22-31457.04) | 65495.94(48497.61-85204.62) | 168.37 | 7.99(5.94-10.33) | 10.10(7.48-13.15) | 0.48(0.31-0.65) |
| Indonesia | 6998.64(5225.52-8983.58) | 19334.27(14365.73-24957.11) | 176.26 | 11.59(8.59-15.04) | 14.38(10.70-18.64) | 0.81(0.77-0.85) |
| Iran (Islamic Republic of) | 2325.92(1736.10-3009.56) | 6727.90(5089.85-8580.52) | 189.26 | 13.50(10.01-17.41) | 15.19(11.35-19.59) | 0.19(0.05-0.34) |
| Iraq | 611.93(459.76-783.98) | 2163.49(1614.81-2830.99) | 253.55 | 13.25(9.96-17.07) | 14.24(10.59-18.45) | 0.24(0.20-0.28) |
| Ireland | 542.76(403.06-699.13) | 1151.35(844.63-1496.16) | 112.13 | 30.22(22.30-39.08) | 35.14(26.02-45.18) | 0.50(0.46-0.55) |
| Israel | 604.23(451.99-775.87) | 1621.42(1198.50-2104.27) | 168.34 | 28.65(21.07-37.05) | 33.05(24.44-42.54) | 0.43(0.35-0.51) |
| Italy | 10655.90(7840.41-13904.92) | 15552.65(11388.18-20362.43) | 45.95 | 29.30(21.85-38.13) | 32.43(24.13-42.17) | 0.49(0.33-0.64) |
| Jamaica | 115.63(86.82-148.71) | 265.77(194.59-346.52) | 129.85 | 14.62(10.99-18.90) | 17.90(13.12-23.25) | 0.77(0.71-0.83) |
| Japan | 18029.21(13260.81-23542.19) | 23665.95(17545.82-30674.89) | 31.26 | 22.13(16.45-28.59) | 23.39(17.45-30.21) | 0.11(0.07-0.14) |
| Jordan | 118.33(88.81-150.64) | 774.59(571.74-1009.83) | 554.61 | 12.55(9.36-15.95) | 15.78(11.90-20.43) | 0.78(0.72-0.83) |
| Kazakhstan | 1407.43(1047.45-1838.99) | 2330.23(1726.18-3032.58) | 65.57 | 22.51(16.94-29.41) | 25.56(18.88-33.00) | 0.35(0.32-0.39) |
| Kenya | 852.84(647.29-1088.25) | 3185.00(2397.55-4080.70) | 273.46 | 17.14(12.83-22.26) | 21.80(16.22-28.20) | 0.86(0.76-0.97) |
| Kiribati | 3.13(2.35-4.07) | 6.71(4.97-8.75) | 114.30 | 14.51(10.72-18.93) | 16.23(12.06-20.85) | 0.24(0.03-0.44) |
| Kuwait | 102.73(76.68-132.47) | 465.59(345.14-616.96) | 353.20 | 14.72(10.99-18.98) | 17.73(13.33-22.65) | 0.71(0.67-0.76) |
| Kyrgyzstan | 275.04(198.45-360.33) | 544.58(406.56-714.87) | 98.00 | 19.00(14.17-24.78) | 20.76(15.57-27.08) | 0.20(0.16-0.24) |
| Lao People's Democratic Republic | 122.97(91.32-159.44) | 330.95(247.89-430.79) | 169.12 | 10.40(7.63-13.60) | 12.16(8.95-15.86) | 0.61(0.58-0.65) |
| Latvia | 333.81(248.68-435.57) | 337.72(248.74-447.47) | 1.17 | 22.81(16.83-29.63) | 25.55(18.94-33.03) | 0.37(0.35-0.39) |
| Lebanon | 150.05(111.92-195.63) | 349.19(262.39-442.79) | 132.72 | 11.74(8.84-15.17) | 14.61(10.89-18.66) | 0.43(0.02-0.86) |
| Lesotho | 102.44(75.85-133.02) | 170.31(127.50-216.31) | 66.25 | 19.26(14.18-25.13) | 24.23(18.03-31.07) | 0.80(0.78-0.83) |
| Liberia | 92.99(69.23-118.66) | 258.02(192.88-337.56) | 177.47 | 14.39(10.65-18.49) | 17.36(12.98-22.63) | 0.79(0.71-0.88) |
| Libya | 167.83(127.27-215.77) | 524.87(387.30-677.74) | 212.75 | 13.35(10.07-17.24) | 15.06(11.13-19.42) | 0.43(0.39-0.46) |
| Lithuania | 425.58(311.89-563.19) | 481.56(354.17-635.81) | 13.15 | 22.27(16.58-28.87) | 24.70(18.45-31.97) | 0.36(0.31-0.42) |
| Luxembourg | 72.49(53.47-94.16) | 159.33(115.83-207.05) | 119.81 | 30.54(22.44-39.55) | 35.17(25.89-45.51) | 0.44(0.41-0.48) |
| Madagascar | 439.12(328.70-564.05) | 1213.00(899.70-1555.40) | 176.24 | 14.44(10.77-18.67) | 16.18(11.93-21.09) | 0.43(0.38-0.48) |
| Malawi | 347.27(259.96-444.22) | 793.23(598.36-1027.99) | 128.42 | 15.24(11.32-19.67) | 18.17(13.39-23.76) | 0.70(0.66-0.74) |
| Malaysia | 694.42(521.63-893.21) | 2430.81(1800.33-3151.20) | 250.05 | 12.34(9.12-16.10) | 15.54(11.36-20.21) | 0.84(0.82-0.86) |
| Maldives | 6.84(4.98-8.94) | 35.86(26.31-46.09) | 424.48 | 10.64(7.80-13.77) | 14.07(10.42-18.35) | 1.11(1.00-1.22) |
| Mali | 309.41(229.05-399.76) | 787.01(596.64-1013.72) | 154.36 | 12.63(9.27-16.42) | 14.52(10.79-18.67) | 0.48(0.44-0.52) |
| Malta | 59.47(44.53-76.41) | 121.51(88.54-163.82) | 104.34 | 29.52(21.66-38.16) | 35.48(26.60-45.65) | 0.58(0.47-0.68) |
| Marshall Islands | 1.34(1.01-1.71) | 3.60(2.69-4.69) | 167.74 | 13.06(9.64-16.85) | 15.31(11.44-20.04) | 0.43(0.31-0.54) |
| Mauritania | 87.06(66.10-111.28) | 223.72(168.67-287.86) | 156.96 | 15.70(11.66-20.36) | 18.58(13.77-24.03) | 0.48(0.40-0.57) |
| Mauritius | 51.38(38.60-65.92) | 135.66(98.27-180.39) | 164.04 | 12.49(9.14-16.10) | 15.25(11.06-19.85) | 0.72(0.69-0.76) |
| Mexico | 3900.10(2926.35-4980.22) | 11145.25(8249.29-14440.53) | 185.77 | 15.98(11.82-20.79) | 18.48(13.67-24.01) | -0.05(-0.23-0.14) |
| Micronesia (Federated States of) | 4.34(3.29-5.65) | 7.76(5.75-10.17) | 78.71 | 15.37(11.40-19.96) | 17.17(12.84-22.09) | 0.25(0.06-0.44) |
| Monaco | 7.73(5.62-10.08) | 12.06(8.62-16.21) | 56.14 | 33.66(24.98-43.32) | 37.77(28.36-48.82) | 0.36(0.32-0.41) |
| Mongolia | 107.12(79.84-136.97) | 309.22(227.75-401.40) | 188.67 | 18.54(13.64-23.92) | 20.72(15.42-26.91) | 0.39(0.36-0.42) |
| Montenegro | 70.70(52.28-92.76) | 102.84(75.26-134.68) | 45.47 | 22.45(16.92-29.29) | 23.45(17.54-30.05) | 0.16(0.13-0.19) |
| Morocco | 871.52(657.32-1109.84) | 2457.14(1814.31-3163.00) | 181.94 | 10.94(8.08-14.06) | 13.35(9.90-17.11) | 0.65(0.63-0.67) |
| Mozambique | 524.07(397.97-680.88) | 1135.48(845.68-1463.37) | 116.66 | 14.62(10.94-19.12) | 17.24(12.60-22.52) | 0.63(0.60-0.67) |
| Myanmar | 1351.65(1003.37-1750.93) | 2979.91(2191.15-3892.33) | 120.46 | 10.34(7.64-13.40) | 12.16(8.92-15.83) | 0.63(0.58-0.68) |
| Namibia | 74.29(55.38-95.18) | 179.87(134.41-231.80) | 142.13 | 19.59(14.51-25.36) | 23.46(17.24-30.29) | 0.56(0.52-0.60) |
| Nauru | 0.47(0.34-0.61) | 0.56(0.41-0.73) | 20.02 | 15.83(11.76-20.67) | 17.87(13.28-23.31) | 0.20(0.11-0.29) |
| Nepal | 446.11(333.03-577.93) | 1158.62(868.80-1505.60) | 159.72 | 7.37(5.50-9.63) | 9.85(7.37-12.82) | 1.07(1.01-1.13) |
| Netherlands | 2583.85(2003.82-3251.16) | 4603.22(3330.15-6030.00) | 78.15 | 29.83(23.06-37.73) | 34.90(25.95-44.47) | 0.56(0.52-0.61) |
| New Zealand | 501.39(377.79-656.10) | 1090.46(797.43-1433.25) | 117.49 | 28.23(21.16-36.88) | 36.12(26.96-46.91) | 0.76(0.69-0.83) |
| Nicaragua | 107.53(79.99-137.25) | 356.61(266.82-460.62) | 231.63 | 12.15(8.89-15.71) | 14.61(10.81-19.01) | 0.60(0.52-0.68) |
| Niger | 245.81(185.41-312.10) | 662.37(495.10-856.28) | 169.47 | 12.75(9.43-16.50) | 13.72(10.00-17.89) | 0.23(0.19-0.28) |
| Nigeria | 4506.13(3396.28-5812.17) | 8003.19(6041.03-10301.87) | 77.61 | 15.22(11.29-19.78) | 14.90(11.04-19.38) | -0.43(-0.70--0.15) |
| Niue | 0.16(0.12-0.21) | 0.22(0.16-0.29) | 34.96 | 16.50(12.15-21.39) | 20.08(15.07-25.81) | 0.60(0.44-0.77) |
| North Macedonia | 210.75(156.07-274.82) | 367.17(269.81-478.80) | 74.22 | 20.77(15.47-26.84) | 22.94(17.03-29.80) | 0.29(0.27-0.31) |
| Northern Mariana Islands | 3.28(2.41-4.31) | 6.77(4.84-9.16) | 106.66 | 17.67(13.10-23.02) | 19.27(14.25-24.94) | 0.14(-0.04-0.32) |
| Norway | 756.13(562.53-970.59) | 1331.63(982.28-1731.57) | 76.11 | 30.07(22.48-39.07) | 34.48(25.66-44.62) | 0.45(0.42-0.49) |
| Oman | 68.13(49.42-88.11) | 348.35(258.61-451.98) | 411.31 | 10.65(7.89-13.79) | 15.34(11.52-19.80) | 1.30(1.28-1.32) |
| Pakistan | 3064.32(2266.78-3951.92) | 8379.02(6313.97-10840.15) | 173.44 | 8.83(6.50-11.40) | 11.37(8.45-14.73) | 0.99(0.92-1.06) |
| Palau | 0.98(0.73-1.26) | 2.88(2.13-3.76) | 194.00 | 17.04(12.74-22.19) | 20.32(14.91-26.40) | 0.43(0.25-0.61) |
| Palestine | 52.55(39.47-67.35) | 211.10(159.91-271.94) | 301.69 | 11.79(8.82-15.18) | 13.16(9.86-17.01) | 0.24(0.18-0.30) |
| Panama | 94.84(71.36-121.83) | 309.84(230.70-400.63) | 226.71 | 11.30(8.35-14.62) | 14.80(10.94-19.13) | 0.83(0.78-0.88) |
| Papua New Guinea | 154.24(114.58-197.12) | 454.40(337.26-591.00) | 194.60 | 12.37(8.89-16.00) | 13.35(9.86-17.43) | 0.14(0.06-0.23) |
| Paraguay | 170.87(131.32-217.67) | 502.36(379.92-645.84) | 193.99 | 13.91(10.33-17.91) | 16.50(12.19-21.28) | 0.59(0.55-0.63) |
| Peru | 901.62(663.13-1155.79) | 2823.21(2078.55-3681.37) | 213.13 | 13.36(9.76-17.32) | 17.45(12.69-22.87) | 0.97(0.94-1.00) |
| Philippines | 2136.60(1602.66-2736.59) | 6505.28(4818.21-8404.21) | 204.47 | 11.77(8.66-15.29) | 14.06(10.44-18.30) | 0.59(0.57-0.62) |
| Poland | 4769.45(3516.45-6166.39) | 7451.48(5515.73-9762.91) | 56.23 | 23.84(17.65-30.63) | 26.74(19.74-34.65) | 0.35(0.32-0.37) |
| Portugal | 1625.83(1198.67-2107.81) | 2616.25(1887.77-3424.15) | 60.92 | 27.48(20.27-35.42) | 32.48(24.18-41.60) | 0.03(-0.16-0.22) |
| Puerto Rico | 322.72(239.01-416.98) | 577.92(427.03-753.10) | 79.08 | 19.56(14.47-25.27) | 23.63(17.56-30.56) | 0.73(0.67-0.79) |
| Qatar | 34.25(24.70-45.78) | 361.87(261.84-477.21) | 956.60 | 15.91(11.82-20.59) | 18.52(13.92-24.25) | 0.45(0.35-0.55) |
| Republic of Korea | 3743.21(2814.91-4819.51) | 10111.59(7470.75-13256.32) | 170.13 | 20.93(15.43-27.08) | 23.13(17.37-29.82) | 0.25(0.21-0.29) |
| Republic of Moldova | 420.29(312.51-547.27) | 604.85(443.70-792.97) | 43.91 | 20.26(15.13-26.09) | 24.52(18.07-31.72) | 0.69(0.59-0.79) |
| Romania | 2835.24(2073.22-3718.29) | 3423.78(2544.96-4466.44) | 20.76 | 20.82(15.47-26.86) | 23.79(17.72-30.85) | 0.43(0.42-0.45) |
| Russian Federation | 19510.06(14274.84-25530.31) | 27062.48(20004.03-35266.42) | 38.71 | 24.95(18.53-32.21) | 28.23(20.94-36.25) | 0.35(0.32-0.37) |
| Rwanda | 237.56(178.54-303.45) | 619.89(468.80-793.12) | 160.94 | 15.01(11.09-19.49) | 17.69(13.35-22.78) | 0.61(0.55-0.67) |
| Saint Kitts and Nevis | 2.28(1.69-2.93) | 7.83(5.76-10.17) | 243.48 | 15.94(11.67-20.57) | 19.56(14.52-25.00) | 0.69(0.63-0.74) |
| Saint Lucia | 6.00(4.42-7.64) | 20.25(15.04-26.64) | 237.46 | 14.75(10.88-19.00) | 18.21(13.55-23.79) | 0.68(0.61-0.75) |
| Saint Vincent and the Grenadines | 4.64(3.50-6.04) | 12.96(9.65-16.78) | 179.49 | 14.16(10.57-18.41) | 17.80(13.24-22.85) | 0.87(0.81-0.93) |
| Samoa | 8.46(6.23-10.95) | 15.85(11.78-20.64) | 87.44 | 17.03(12.48-22.05) | 18.25(13.52-23.46) | 0.06(-0.09-0.21) |
| San Marino | 4.57(3.36-5.93) | 8.52(6.25-11.10) | 86.47 | 31.77(23.49-41.06) | 36.01(26.60-46.33) | 0.43(0.37-0.49) |
| Sao Tome and Principe | 4.85(3.58-6.36) | 11.93(9.04-15.33) | 145.93 | 14.58(10.73-18.92) | 17.40(12.92-22.73) | 0.56(0.52-0.60) |
| Saudi Arabia | 643.21(483.41-831.04) | 3377.20(2474.32-4425.81) | 425.06 | 12.45(9.35-15.95) | 16.79(12.37-21.75) | 0.98(0.94-1.03) |
| Senegal | 276.61(206.11-352.06) | 703.38(532.90-905.46) | 154.29 | 14.60(10.71-18.82) | 16.07(12.02-20.77) | 0.23(0.18-0.29) |
| Serbia | 1270.85(926.74-1681.34) | 1510.69(1105.51-1963.83) | 18.87 | 21.53(15.85-27.81) | 23.93(17.60-30.62) | 0.41(0.39-0.43) |
| Seychelles | 3.59(2.71-4.62) | 10.42(7.69-13.74) | 189.87 | 13.51(10.09-17.49) | 15.75(11.68-20.43) | 0.48(0.44-0.51) |
| Sierra Leone | 142.26(107.99-181.20) | 346.53(261.95-449.17) | 143.58 | 13.24(9.85-17.00) | 15.06(11.20-19.50) | 0.37(0.34-0.40) |
| Singapore | 276.14(208.62-355.38) | 1098.49(802.54-1432.66) | 297.80 | 20.64(15.18-26.81) | 24.83(18.20-32.11) | 0.54(0.48-0.60) |
| Slovakia | 597.51(446.26-775.62) | 966.95(714.87-1256.49) | 61.83 | 22.36(16.69-29.04) | 24.33(18.11-31.06) | 0.20(0.15-0.24) |
| Slovenia | 241.25(179.08-317.70) | 410.12(298.99-538.05) | 70.00 | 21.82(16.36-28.56) | 24.58(18.01-31.71) | 0.41(0.37-0.46) |
| Solomon Islands | 12.54(9.23-16.21) | 31.26(23.50-40.06) | 149.22 | 13.21(9.70-17.20) | 14.80(11.00-19.18) | 0.27(0.10-0.44) |
| Somalia | 253.10(188.41-332.68) | 688.01(513.26-879.30) | 171.83 | 14.60(10.80-18.90) | 16.10(11.82-20.78) | 0.40(0.37-0.42) |
| South Africa | 3077.20(2305.16-3952.17) | 8012.32(5956.03-10419.92) | 160.38 | 28.20(20.69-36.86) | 33.50(24.59-44.12) | 0.65(0.58-0.72) |
| South Sudan | 242.67(183.24-312.20) | 452.73(337.15-591.97) | 86.56 | 15.09(11.19-19.66) | 17.24(12.78-22.45) | 0.51(0.49-0.53) |
| Spain | 7291.96(5371.06-9674.63) | 13001.85(9566.91-16943.78) | 78.30 | 32.01(23.82-41.47) | 36.78(27.51-47.74) | 0.37(0.27-0.48) |
| Sri Lanka | 728.97(543.70-945.48) | 1624.46(1192.67-2126.82) | 122.84 | 11.19(8.28-14.67) | 12.97(9.55-16.88) | 0.53(0.52-0.55) |
| Sudan | 528.82(402.12-673.59) | 1593.72(1190.79-2039.04) | 201.37 | 9.45(7.04-12.17) | 12.80(9.44-16.45) | 1.04(0.96-1.13) |
| Suriname | 21.64(16.04-28.15) | 59.96(43.87-79.56) | 177.08 | 15.20(11.23-19.75) | 18.84(13.87-24.85) | 0.77(0.74-0.79) |
| Sweden | 1558.53(1174.68-2018.72) | 2734.41(1999.33-3573.45) | 75.45 | 27.95(21.15-36.05) | 37.59(27.84-48.34) | 0.97(0.62-1.32) |
| Switzerland | 1236.22(911.45-1583.17) | 2145.55(1570.88-2798.99) | 73.56 | 29.36(21.65-37.52) | 32.23(24.02-41.48) | 0.33(0.31-0.34) |
| Syrian Arab Republic | 391.73(299.54-501.88) | 1019.11(750.63-1332.40) | 160.15 | 11.92(9.01-15.35) | 13.62(10.23-17.68) | 0.33(0.24-0.42) |
| Taiwan (Province of China) | 1060.61(782.38-1400.93) | 2352.16(1696.03-3093.89) | 121.78 | 10.91(7.99-14.22) | 12.81(9.43-16.56) | 0.70(0.61-0.78) |
| Tajikistan | 249.92(184.23-326.43) | 632.40(466.27-830.56) | 153.04 | 17.09(12.73-22.10) | 18.18(13.57-23.60) | 0.14(0.10-0.18) |
| Thailand | 2334.02(1705.86-3025.34) | 7533.14(5529.39-9991.00) | 222.75 | 11.11(8.07-14.42) | 14.95(11.06-19.44) | 1.06(1.03-1.09) |
| Timor-Leste | 21.36(16.04-27.64) | 49.15(36.28-63.35) | 130.08 | 10.23(7.50-13.37) | 10.85(7.96-14.07) | 0.23(0.19-0.26) |
| Togo | 100.91(76.64-129.15) | 349.00(260.42-450.28) | 245.86 | 13.37(9.92-17.31) | 15.24(11.43-19.82) | 0.35(0.30-0.41) |
| Tokelau | 0.08(0.06-0.11) | 0.13(0.09-0.16) | 53.56 | 14.91(11.10-19.38) | 18.15(13.27-23.50) | 0.64(0.51-0.77) |
| Tonga | 4.91(3.60-6.48) | 7.44(5.53-9.67) | 51.62 | 16.18(11.87-21.26) | 18.34(13.57-23.79) | 0.18(-0.07-0.44) |
| Trinidad and Tobago | 71.60(53.33-91.70) | 178.11(130.33-229.60) | 148.76 | 16.15(11.98-20.84) | 19.08(14.12-24.46) | 0.68(0.62-0.75) |
| Tunisia | 333.90(248.66-434.63) | 927.98(686.96-1199.81) | 177.92 | 11.50(8.56-14.94) | 13.73(10.26-17.65) | 0.57(0.54-0.60) |
| Turkey | 2596.27(1935.82-3350.59) | 7106.27(5284.49-9136.49) | 173.71 | 12.38(9.20-15.95) | 15.09(11.20-19.41) | 0.61(0.58-0.64) |
| Turkmenistan | 201.18(150.96-260.08) | 537.56(397.23-697.03) | 167.20 | 19.67(14.84-25.46) | 23.25(17.20-30.08) | 0.52(0.49-0.55) |
| Tuvalu | 0.49(0.36-0.64) | 0.92(0.68-1.20) | 88.08 | 14.43(10.61-18.77) | 16.67(12.21-21.70) | 0.43(0.28-0.57) |
| Uganda | 564.62(423.51-729.04) | 1479.37(1115.41-1907.99) | 162.01 | 14.70(11.01-19.13) | 17.33(12.78-22.62) | 0.62(0.59-0.66) |
| Ukraine | 7560.15(5532.53-9852.93) | 7879.53(5840.52-10239.77) | 4.22 | 25.52(18.93-32.97) | 26.69(19.88-34.61) | 0.16(0.14-0.17) |
| United Arab Emirates | 104.95(73.26-141.57) | 1459.25(1033.84-1991.22) | 1290.46 | 12.87(9.44-16.70) | 16.60(12.34-21.40) | 0.87(0.83-0.92) |
| United Kingdom | 12279.22(9113.10-15860.50) | 18147.31(13447.30-23707.90) | 47.79 | 34.99(26.14-45.30) | 38.51(28.69-49.56) | 0.35(0.33-0.36) |
| United Republic of Tanzania | 996.28(744.57-1272.07) | 2892.50(2157.28-3724.33) | 190.33 | 15.76(11.75-20.11) | 19.22(14.03-24.98) | 0.73(0.69-0.77) |
| United States of America | 50440.53(37953.73-64453.10) | 105771.51(80203.68-133616.58) | 109.70 | 38.57(28.87-49.83) | 47.80(37.16-59.81) | 0.64(0.52-0.75) |
| United States Virgin Islands | 9.45(7.03-12.17) | 16.68(12.34-22.11) | 76.61 | 19.31(14.16-25.02) | 22.36(16.70-28.88) | 0.51(0.44-0.57) |
| Uruguay | 415.74(307.00-542.09) | 687.95(509.85-884.01) | 65.47 | 25.09(18.72-32.31) | 34.10(25.62-43.31) | 0.98(0.86-1.09) |
| Uzbekistan | 1099.21(805.86-1432.72) | 3119.34(2306.05-4085.11) | 183.78 | 18.74(13.88-24.32) | 22.16(16.57-28.64) | 0.54(0.49-0.58) |
| Vanuatu | 6.09(4.52-7.80) | 16.20(12.35-21.03) | 166.08 | 13.91(10.21-17.84) | 15.38(11.49-19.99) | 0.32(0.28-0.37) |
| Venezuela (Bolivarian Republic of) | 750.73(568.65-951.05) | 2378.43(1773.43-3099.48) | 216.82 | 13.37(9.95-17.28) | 15.60(11.62-20.26) | 0.51(0.48-0.54) |
| Viet Nam | 1932.32(1433.51-2531.11) | 5896.07(4274.16-7686.56) | 205.13 | 10.01(7.41-13.00) | 11.40(8.33-14.82) | 0.50(0.46-0.55) |
| Yemen | 282.82(213.27-359.55) | 897.69(674.60-1132.43) | 217.40 | 8.90(6.66-11.45) | 10.28(7.52-13.22) | 0.55(0.53-0.56) |
| Zambia | 287.60(216.09-370.29) | 839.66(632.18-1079.25) | 191.95 | 15.85(11.69-20.35) | 18.47(13.73-23.84) | 0.51(0.45-0.58) |
| Zimbabwe | 499.35(379.92-636.38) | 870.25(651.80-1102.85) | 74.28 | 20.12(15.22-25.70) | 21.84(16.29-27.99) | 0.13(0.07-0.20) |

S Table6 The DALY and age-standardized DALY rate of national hip osteoarthritis in 1990 and 2019, and its temporal trends from 1990 to 2019

| **Nation** | **DALY No. (95% UI)** | | **Change in absolute number (%)** | **Age-standardized DALY rate No.(95% UI)** | | **1990-2019 EAPC No. (95%CI)** |
| --- | --- | --- | --- | --- | --- | --- |
|  | **1990** | **2019** |  | **1990** | **2019** |  |
| Afghanistan | 393.78(182.39-810.20) | 893.55(412.63-1885.20) | 126.92 | 5.50(2.57-11.25) | 6.47(3.01-13.39) | 0.73(0.59-0.87) |
| Albania | 234.56(108.55-486.30) | 537.61(254.87-1113.43) | 129.19 | 10.96(5.12-22.77) | 12.78(6.06-26.53) | 0.60(0.57-0.62) |
| Algeria | 873.78(403.72-1842.44) | 3131.12(1468.42-6477.96) | 258.34 | 6.87(3.22-14.37) | 8.85(4.18-18.33) | 0.88(0.84-0.93) |
| American Samoa | 2.68(1.25-5.61) | 6.10(2.88-12.60) | 127.82 | 11.12(5.18-23.03) | 12.45(5.92-25.71) | 0.21(-0.04-0.45) |
| Andorra | 11.39(5.31-23.66) | 33.19(15.82-68.27) | 191.47 | 20.08(9.38-41.83) | 23.85(11.40-48.86) | 0.57(0.47-0.66) |
| Angola | 358.56(165.81-760.30) | 1231.00(570.93-2555.14) | 243.32 | 8.74(4.07-18.46) | 10.27(4.82-21.14) | 0.58(0.54-0.61) |
| Antigua and Barbuda | 4.63(2.18-9.53) | 10.89(5.15-22.66) | 135.35 | 8.94(4.20-18.65) | 10.45(4.99-21.63) | 0.52(0.50-0.54) |
| Argentina | 5120.95(2423.37-10674.21) | 11113.62(5348.40-22846.14) | 117.02 | 15.86(7.49-32.96) | 20.99(10.07-42.99) | 0.98(0.87-1.09) |
| Armenia | 296.87(139.90-625.00) | 536.14(252.74-1106.46) | 80.60 | 10.76(5.14-22.36) | 12.94(6.11-26.58) | 0.66(0.65-0.68) |
| Australia | 3457.52(1610.97-7256.46) | 9333.64(4488.14-18894.10) | 169.95 | 17.90(8.32-37.79) | 23.88(11.38-48.61) | 1.00(0.91-1.09) |
| Austria | 2242.19(1081.89-4591.74) | 3772.77(1787.80-7740.64) | 68.26 | 19.84(9.56-40.47) | 22.98(10.91-47.49) | 0.48(0.45-0.51) |
| Azerbaijan | 563.09(267.72-1169.97) | 1334.03(609.92-2838.61) | 136.91 | 11.12(5.27-23.13) | 13.46(6.27-28.84) | 0.68(0.62-0.74) |
| Bahamas | 15.98(7.57-33.36) | 45.26(21.01-93.97) | 183.26 | 10.08(4.76-21.30) | 11.24(5.23-23.12) | 0.36(0.31-0.42) |
| Bahrain | 18.81(8.61-39.55) | 136.65(63.27-282.00) | 626.40 | 8.48(3.92-17.69) | 10.25(4.72-21.15) | 0.60(0.56-0.64) |
| Bangladesh | 3142.23(1459.05-6524.84) | 10754.34(5067.46-22256.04) | 242.25 | 6.22(2.89-12.70) | 7.90(3.72-16.26) | 0.95(0.89-1.01) |
| Barbados | 27.47(12.97-57.00) | 54.36(25.93-112.60) | 97.91 | 9.94(4.64-20.78) | 11.33(5.37-23.42) | 0.46(0.43-0.50) |
| Belarus | 1514.47(718.36-3125.30) | 2125.83(1024.77-4343.42) | 40.37 | 11.69(5.54-24.09) | 13.63(6.60-28.09) | 0.55(0.51-0.58) |
| Belgium | 2888.62(1367.99-5901.76) | 4672.74(2215.59-9702.40) | 61.76 | 19.54(9.20-39.92) | 22.52(10.58-46.63) | 0.45(0.41-0.49) |
| Belize | 8.94(4.21-18.44) | 33.93(15.99-70.01) | 279.30 | 9.54(4.44-19.87) | 11.83(5.56-24.70) | 0.73(0.54-0.91) |
| Benin | 170.77(78.83-358.17) | 536.34(246.88-1139.86) | 214.06 | 8.36(3.87-17.53) | 10.39(4.84-21.78) | 0.76(0.67-0.85) |
| Bermuda | 7.40(3.57-15.39) | 15.22(7.15-31.88) | 105.56 | 11.67(5.59-24.15) | 12.39(5.82-25.81) | 0.17(0.11-0.23) |
| Bhutan | 19.21(9.06-38.88) | 56.61(27.82-115.64) | 194.71 | 7.11(3.37-14.52) | 9.45(4.57-19.37) | 1.08(1.02-1.15) |
| Bolivia (Plurinational State of) | 251.35(116.25-516.52) | 858.83(408.60-1759.07) | 241.68 | 7.58(3.51-15.53) | 9.46(4.50-19.45) | 0.80(0.76-0.83) |
| Bosnia and Herzegovina | 460.18(217.57-965.47) | 744.17(354.17-1549.49) | 61.71 | 10.94(5.23-22.81) | 12.82(6.11-26.68) | 0.60(0.51-0.68) |
| Botswana | 62.51(29.39-128.74) | 202.37(96.23-414.14) | 223.73 | 10.54(4.92-22.03) | 13.68(6.53-28.15) | 0.82(0.75-0.89) |
| Brazil | 8212.52(3842.70-16919.03) | 26108.03(12253.24-54241.90) | 217.91 | 8.88(4.13-18.36) | 10.86(5.08-22.53) | 0.72(0.70-0.73) |
| Brunei Darussalam | 13.71(6.50-28.29) | 51.65(24.34-106.25) | 276.71 | 12.80(6.05-26.50) | 15.46(7.34-31.87) | 0.69(0.62-0.76) |
| Bulgaria | 1619.58(763.46-3347.06) | 1911.17(910.20-4049.58) | 18.00 | 13.05(6.11-26.95) | 14.04(6.55-29.52) | 0.17(0.13-0.21) |
| Burkina Faso | 353.85(165.85-739.51) | 900.75(411.37-1865.91) | 154.56 | 7.88(3.72-16.44) | 9.30(4.37-19.36) | 0.58(0.56-0.61) |
| Burundi | 206.20(96.78-423.24) | 436.39(202.94-905.49) | 111.64 | 8.62(4.11-17.79) | 8.99(4.23-18.80) | 0.16(0.15-0.16) |
| Cabo Verde | 19.16(8.85-40.19) | 46.49(22.00-97.57) | 142.65 | 8.63(4.01-18.20) | 10.60(5.04-22.06) | 0.71(0.68-0.73) |
| Cambodia | 281.85(132.82-575.52) | 823.11(381.76-1719.99) | 192.04 | 6.08(2.87-12.39) | 6.66(3.12-14.01) | 0.33(0.30-0.36) |
| Cameroon | 475.89(222.78-986.91) | 1460.97(671.91-2979.76) | 207.00 | 10.22(4.76-21.12) | 11.46(5.30-23.38) | 0.35(0.32-0.37) |
| Canada | 4556.09(2161.98-9407.98) | 12234.25(5876.99-25207.64) | 168.52 | 14.23(6.72-29.42) | 18.92(8.99-39.11) | 0.61(0.43-0.79) |
| Central African Republic | 103.66(48.02-216.22) | 209.85(97.43-436.89) | 102.43 | 8.60(4.04-17.98) | 9.18(4.36-19.21) | 0.24(0.21-0.26) |
| Chad | 214.28(101.34-433.01) | 488.79(226.15-1009.64) | 128.11 | 7.48(3.57-15.22) | 8.27(3.88-17.10) | 0.31(0.29-0.33) |
| Chile | 1700.26(797.22-3495.71) | 5274.21(2499.44-10802.68) | 210.20 | 16.80(7.87-34.41) | 22.03(10.41-45.00) | 0.89(0.78-1.00) |
| China | 48358.10(22107.00-98907.26) | 153024.73(70238.43-319575.97) | 216.44 | 5.49(2.53-11.26) | 7.46(3.43-15.48) | 1.16(1.06-1.26) |
| Colombia | 1397.74(633.67-2928.93) | 4719.20(2254.70-9672.68) | 237.63 | 7.61(3.50-15.92) | 8.92(4.25-18.23) | 0.53(0.51-0.56) |
| Comoros | 21.03(9.90-43.87) | 52.05(24.07-106.62) | 147.52 | 9.35(4.44-19.61) | 10.43(4.85-21.65) | 0.39(0.38-0.39) |
| Congo | 108.19(50.35-222.12) | 326.27(150.97-670.94) | 201.56 | 9.89(4.67-20.26) | 11.62(5.45-24.14) | 0.56(0.56-0.57) |
| Cook Islands | 1.34(0.63-2.79) | 3.11(1.48-6.42) | 132.61 | 10.32(4.84-21.58) | 12.67(6.08-26.01) | 0.55(0.39-0.71) |
| Costa Rica | 146.30(67.92-302.92) | 492.53(230.14-1013.71) | 236.65 | 8.13(3.77-16.90) | 9.49(4.43-19.45) | 0.52(0.49-0.54) |
| C么te d'Ivoire | 383.44(179.12-792.77) | 1156.77(544.19-2455.65) | 201.68 | 8.76(4.08-18.02) | 9.96(4.66-21.00) | 0.38(0.34-0.41) |
| Croatia | 782.81(368.12-1610.73) | 1128.21(544.56-2344.12) | 44.12 | 12.18(5.75-24.99) | 13.90(6.63-29.02) | 0.52(0.47-0.56) |
| Cuba | 910.97(424.92-1850.95) | 1952.22(931.45-4009.97) | 114.30 | 8.91(4.15-18.16) | 10.57(5.05-21.73) | 0.65(0.60-0.69) |
| Cyprus | 147.93(71.28-307.40) | 414.15(197.50-846.75) | 179.97 | 17.87(8.63-37.19) | 21.71(10.35-44.01) | 0.72(0.65-0.78) |
| Czechia | 1686.21(803.23-3516.62) | 2779.21(1323.94-5791.70) | 64.82 | 12.47(5.96-26.17) | 14.20(6.83-29.37) | 0.43(0.39-0.46) |
| Democratic People's Republic of Korea | 930.82(432.40-1897.65) | 1845.71(862.49-3820.08) | 98.29 | 5.61(2.63-11.44) | 5.71(2.66-11.80) | 0.05(0.02-0.07) |
| Democratic Republic of the Congo | 1524.32(706.09-3160.95) | 3593.24(1700.28-7394.92) | 135.73 | 9.25(4.32-18.99) | 9.51(4.61-19.42) | -0.02(-0.08-0.04) |
| Denmark | 2101.61(1010.57-4300.69) | 2603.55(1234.27-5352.64) | 23.88 | 27.50(13.26-56.00) | 24.60(11.62-50.22) | -0.51(-0.77--0.25) |
| Djibouti | 13.71(6.48-28.36) | 73.41(33.99-151.86) | 435.24 | 8.85(4.18-18.20) | 11.15(5.24-23.19) | 0.89(0.85-0.92) |
| Dominica | 6.14(2.87-12.67) | 9.31(4.35-19.34) | 51.79 | 9.01(4.26-18.47) | 10.49(4.87-21.79) | 0.51(0.46-0.57) |
| Dominican Republic | 329.94(153.40-685.56) | 1018.45(473.82-2106.00) | 208.68 | 8.62(4.02-18.07) | 10.81(5.02-22.26) | 0.88(0.83-0.92) |
| Ecuador | 528.01(246.57-1081.51) | 1749.74(823.17-3633.03) | 231.39 | 9.64(4.56-19.71) | 11.40(5.36-23.78) | 0.47(0.39-0.54) |
| Egypt | 2333.39(1091.81-4882.05) | 6340.46(2949.11-12971.01) | 171.73 | 7.61(3.58-15.87) | 9.37(4.34-19.30) | 0.49(0.39-0.59) |
| El Salvador | 228.56(107.08-475.97) | 533.28(254.24-1102.15) | 133.32 | 7.61(3.56-15.66) | 9.09(4.33-18.78) | 0.61(0.50-0.71) |
| Equatorial Guinea | 17.56(8.28-36.50) | 64.99(30.59-136.11) | 270.11 | 8.67(4.14-17.92) | 12.54(5.95-26.31) | 1.52(1.41-1.63) |
| Eritrea | 85.13(39.45-178.24) | 260.23(119.71-536.82) | 205.69 | 8.11(3.80-16.91) | 9.04(4.29-18.68) | 0.36(0.31-0.42) |
| Estonia | 247.64(115.00-508.82) | 346.62(163.51-722.58) | 39.97 | 12.18(5.65-24.96) | 14.41(6.79-30.40) | 0.68(0.64-0.72) |
| Eswatini | 38.85(18.41-81.48) | 88.03(41.04-182.76) | 126.60 | 12.62(6.04-26.12) | 14.49(6.87-29.99) | 0.25(0.05-0.45) |
| Ethiopia | 1878.17(869.98-3853.65) | 4482.53(2100.26-9221.12) | 138.67 | 9.05(4.27-18.74) | 10.22(4.85-21.26) | 0.46(0.42-0.50) |
| Fiji | 34.46(16.42-70.96) | 83.87(39.39-174.43) | 143.36 | 8.97(4.26-18.59) | 10.75(5.16-22.06) | 0.49(0.38-0.61) |
| Finland | 1407.85(660.05-2878.00) | 2591.86(1226.70-5321.13) | 84.10 | 20.28(9.49-41.80) | 23.56(11.27-48.07) | 0.52(0.46-0.57) |
| France | 16174.84(7656.73-32895.82) | 29073.08(13965.09-59757.15) | 79.74 | 20.41(9.65-42.00) | 23.75(11.28-48.80) | 0.61(0.34-0.87) |
| Gabon | 58.59(27.86-123.23) | 148.90(69.20-310.61) | 154.14 | 10.33(4.95-21.77) | 13.53(6.33-28.15) | 0.89(0.82-0.96) |
| Gambia | 31.62(14.59-65.03) | 99.00(46.11-204.74) | 213.07 | 8.48(3.92-17.60) | 9.87(4.63-20.46) | 0.49(0.45-0.53) |
| Georgia | 783.82(370.11-1610.55) | 762.06(357.80-1571.31) | -2.78 | 12.64(5.98-25.84) | 13.14(6.15-27.05) | 0.11(0.09-0.13) |
| Germany | 24731.89(11874.07-51137.52) | 40137.51(19190.80-81780.86) | 62.29 | 20.28(9.72-41.53) | 23.42(11.11-47.54) | 0.47(0.41-0.52) |
| Ghana | 554.60(259.95-1142.22) | 1784.15(823.70-3759.26) | 221.70 | 8.42(3.96-17.68) | 10.46(4.92-21.55) | 0.71(0.65-0.77) |
| Greece | 2716.47(1295.94-5544.57) | 4394.65(2095.12-8990.75) | 61.78 | 18.22(8.76-37.06) | 20.90(10.02-42.72) | 1.39(1.00-1.79) |
| Greenland | 5.35(2.55-10.93) | 13.39(6.26-27.77) | 150.43 | 14.77(7.07-30.37) | 18.78(8.94-38.61) | 0.84(0.76-0.92) |
| Grenada | 5.69(2.70-11.73) | 11.32(5.26-23.81) | 98.77 | 8.24(3.85-17.03) | 9.90(4.58-20.92) | 0.61(0.55-0.66) |
| Guam | 8.58(3.99-17.64) | 23.39(10.95-48.70) | 172.67 | 10.40(4.87-21.56) | 12.24(5.77-25.36) | 0.57(0.52-0.63) |
| Guatemala | 257.67(122.75-520.07) | 907.66(433.35-1855.97) | 252.25 | 6.73(3.20-13.67) | 7.97(3.79-16.56) | 0.57(0.53-0.60) |
| Guinea | 275.30(128.18-579.51) | 514.24(242.47-1080.75) | 86.79 | 8.15(3.83-17.17) | 9.00(4.26-18.66) | 0.32(0.31-0.34) |
| Guinea-Bissau | 33.83(15.68-69.77) | 68.21(31.66-140.29) | 101.63 | 8.09(3.77-16.58) | 8.86(4.08-18.29) | 0.26(0.22-0.30) |
| Guyana | 33.40(15.54-69.29) | 64.20(30.21-136.23) | 92.20 | 8.49(3.94-17.82) | 9.95(4.66-20.97) | 0.53(0.47-0.60) |
| Haiti | 235.92(110.94-490.06) | 566.71(264.52-1161.30) | 140.21 | 7.04(3.33-14.48) | 7.73(3.69-16.07) | 0.39(0.36-0.42) |
| Honduras | 150.41(69.44-313.09) | 523.89(239.29-1077.18) | 248.30 | 7.04(3.26-14.73) | 8.35(3.86-16.96) | 0.61(0.54-0.68) |
| Hungary | 1915.48(910.95-4005.07) | 2645.22(1248.58-5486.53) | 38.10 | 13.21(6.27-27.39) | 14.64(6.99-30.20) | 0.33(0.30-0.37) |
| Iceland | 72.19(35.05-144.47) | 138.14(66.49-282.11) | 91.37 | 26.13(12.69-52.11) | 26.35(12.73-54.28) | -0.16(-0.28--0.03) |
| India | 33306.85(15990.73-68081.14) | 102627.82(49223.86-209693.26) | 208.13 | 6.70(3.21-13.55) | 8.60(4.12-17.37) | 1.08(0.92-1.25) |
| Indonesia | 7041.11(3256.02-14453.51) | 18579.41(8603.43-38019.42) | 163.87 | 6.79(3.14-13.79) | 8.08(3.76-16.65) | 0.64(0.61-0.68) |
| Iran (Islamic Republic of) | 2094.97(969.86-4322.40) | 6773.96(3163.09-13967.69) | 223.34 | 7.53(3.50-15.59) | 8.75(4.07-18.12) | 0.35(0.21-0.49) |
| Iraq | 638.67(301.23-1321.67) | 2137.57(1010.39-4360.83) | 234.69 | 7.91(3.73-16.31) | 8.66(4.10-17.72) | 0.32(0.28-0.36) |
| Ireland | 797.95(377.88-1646.81) | 1683.60(802.17-3442.86) | 110.99 | 20.03(9.53-41.78) | 23.34(11.07-47.70) | 0.51(0.46-0.56) |
| Israel | 898.42(421.39-1838.19) | 2438.67(1153.06-5021.10) | 171.44 | 18.87(8.87-38.52) | 21.98(10.31-45.22) | 0.47(0.40-0.54) |
| Italy | 16735.27(7964.07-34136.81) | 27492.32(13060.54-56426.03) | 64.28 | 19.48(9.23-39.97) | 21.73(10.26-44.60) | 0.51(0.37-0.65) |
| Jamaica | 153.43(72.35-317.30) | 312.93(148.68-641.85) | 103.96 | 8.81(4.16-18.25) | 10.49(4.98-21.55) | 0.68(0.62-0.74) |
| Japan | 22547.18(10657.23-46811.61) | 43060.56(20492.78-88679.78) | 90.98 | 13.25(6.24-27.43) | 14.47(6.81-29.95) | 0.27(0.17-0.38) |
| Jordan | 110.54(51.14-230.50) | 709.15(332.76-1470.67) | 541.53 | 7.62(3.52-15.71) | 9.75(4.54-20.14) | 0.86(0.82-0.91) |
| Kazakhstan | 1621.90(757.70-3378.55) | 2576.51(1211.03-5278.81) | 58.86 | 12.69(5.99-26.34) | 14.49(6.78-29.60) | 0.37(0.34-0.40) |
| Kenya | 852.66(397.76-1775.03) | 2728.80(1285.03-5653.82) | 220.03 | 9.90(4.59-20.63) | 11.47(5.38-23.79) | 0.53(0.46-0.59) |
| Kiribati | 3.16(1.47-6.45) | 6.73(3.13-13.97) | 113.20 | 8.32(3.91-17.04) | 9.39(4.50-19.41) | 0.28(0.07-0.48) |
| Kuwait | 69.77(32.18-143.65) | 362.23(172.84-755.27) | 419.16 | 9.13(4.28-18.95) | 11.04(5.11-23.13) | 0.73(0.69-0.77) |
| Kyrgyzstan | 328.20(155.88-688.65) | 566.26(270.66-1173.82) | 72.54 | 10.78(5.10-22.61) | 11.93(5.62-24.46) | 0.25(0.21-0.29) |
| Lao People's Democratic Republic | 133.14(62.65-276.67) | 334.60(152.50-701.28) | 151.32 | 6.25(2.90-13.07) | 7.26(3.38-15.27) | 0.56(0.53-0.59) |
| Latvia | 437.03(203.64-914.55) | 519.19(245.81-1078.41) | 18.80 | 12.32(5.72-25.61) | 14.21(6.78-29.65) | 0.54(0.51-0.58) |
| Lebanon | 167.80(78.10-344.61) | 470.58(220.25-970.07) | 180.44 | 7.19(3.33-14.68) | 9.03(4.23-18.66) | 0.52(0.16-0.88) |
| Lesotho | 101.80(47.17-209.16) | 158.90(75.21-332.15) | 56.09 | 10.12(4.75-20.73) | 12.05(5.73-24.97) | 0.59(0.57-0.61) |
| Liberia | 98.84(45.92-202.80) | 232.55(108.37-485.19) | 135.28 | 8.74(4.11-17.82) | 10.46(4.91-21.78) | 0.79(0.69-0.90) |
| Libya | 159.19(74.31-325.04) | 511.45(240.83-1054.76) | 221.28 | 8.14(3.79-16.68) | 9.24(4.28-19.10) | 0.44(0.41-0.47) |
| Lithuania | 541.98(257.13-1140.73) | 729.84(343.02-1515.04) | 34.66 | 12.07(5.73-25.29) | 13.78(6.58-28.46) | 0.51(0.46-0.57) |
| Luxembourg | 108.44(51.71-222.31) | 223.39(106.35-455.99) | 106.00 | 20.29(9.59-41.91) | 23.26(11.05-47.35) | 0.46(0.42-0.49) |
| Madagascar | 456.01(209.50-944.67) | 1091.54(515.84-2279.92) | 139.37 | 8.66(4.03-17.84) | 9.32(4.43-19.40) | 0.29(0.25-0.32) |
| Malawi | 361.90(167.39-752.84) | 788.71(368.05-1610.75) | 117.94 | 9.05(4.29-18.50) | 10.34(4.85-21.38) | 0.54(0.49-0.60) |
| Malaysia | 720.95(336.62-1489.05) | 2562.72(1193.90-5383.54) | 255.46 | 7.44(3.51-15.17) | 9.24(4.24-19.41) | 0.80(0.79-0.82) |
| Maldives | 6.13(2.85-12.98) | 29.15(13.49-60.56) | 375.33 | 6.52(3.04-13.74) | 8.47(3.95-17.66) | 1.05(0.94-1.17) |
| Mali | 328.54(152.18-679.90) | 794.02(372.80-1629.41) | 141.68 | 7.71(3.61-15.85) | 8.84(4.14-18.21) | 0.51(0.48-0.54) |
| Malta | 83.36(39.49-169.26) | 201.59(96.61-410.38) | 141.83 | 19.51(9.25-39.56) | 23.59(11.10-48.09) | 0.61(0.50-0.72) |
| Marshall Islands | 1.29(0.61-2.63) | 3.27(1.53-6.72) | 154.17 | 7.53(3.53-15.61) | 8.96(4.23-18.23) | 0.48(0.37-0.59) |
| Mauritania | 98.92(46.21-203.54) | 245.38(113.10-504.59) | 148.07 | 9.65(4.56-19.84) | 11.37(5.33-23.90) | 0.50(0.43-0.57) |
| Mauritius | 57.03(26.32-118.32) | 158.71(73.96-327.82) | 178.31 | 7.48(3.51-15.33) | 8.96(4.21-18.45) | 0.63(0.61-0.66) |
| Mexico | 4069.58(1906.70-8348.62) | 12353.99(5760.97-25503.43) | 203.57 | 9.10(4.24-18.93) | 10.33(4.86-21.38) | -0.04(-0.21-0.12) |
| Micronesia (Federated States of) | 4.27(2.01-8.77) | 7.36(3.43-15.31) | 72.40 | 8.91(4.26-18.52) | 10.01(4.70-20.81) | 0.26(0.08-0.45) |
| Monaco | 14.29(6.78-29.61) | 21.45(10.09-44.68) | 50.14 | 22.45(10.65-46.86) | 25.24(11.92-52.42) | 0.38(0.33-0.42) |
| Mongolia | 112.79(52.75-232.15) | 294.04(135.73-604.66) | 160.71 | 10.64(4.96-22.12) | 11.87(5.56-24.28) | 0.38(0.34-0.42) |
| Montenegro | 81.24(37.21-168.87) | 133.19(62.94-274.74) | 63.94 | 12.90(5.93-26.82) | 13.78(6.47-28.66) | 0.26(0.23-0.29) |
| Morocco | 934.87(425.21-1947.50) | 2668.62(1262.64-5489.87) | 185.45 | 6.60(3.06-13.52) | 8.19(3.85-16.58) | 0.71(0.69-0.73) |
| Mozambique | 541.85(253.72-1127.66) | 1118.86(527.06-2306.31) | 106.49 | 8.67(4.02-17.97) | 9.72(4.63-20.01) | 0.45(0.42-0.48) |
| Myanmar | 1472.51(691.30-3064.48) | 3414.43(1622.28-7116.04) | 131.88 | 6.20(2.91-12.83) | 7.13(3.38-14.77) | 0.56(0.52-0.61) |
| Namibia | 76.36(36.27-155.84) | 173.00(81.09-354.88) | 126.54 | 10.37(4.91-21.22) | 11.85(5.62-24.60) | 0.41(0.36-0.46) |
| Nauru | 0.38(0.18-0.79) | 0.48(0.23-0.98) | 26.54 | 9.30(4.40-19.08) | 10.39(4.94-21.36) | 0.19(0.11-0.28) |
| Nepal | 651.05(306.58-1338.14) | 2044.41(959.07-4138.22) | 214.02 | 6.39(3.02-13.06) | 8.78(4.10-17.80) | 1.20(1.12-1.27) |
| Netherlands | 4286.54(2111.97-8653.37) | 7428.34(3503.92-15542.03) | 73.29 | 22.04(10.78-44.42) | 23.44(11.13-48.46) | 0.20(0.11-0.28) |
| New Zealand | 692.46(330.36-1418.39) | 1680.81(802.33-3465.73) | 142.73 | 18.00(8.57-37.16) | 22.84(10.81-46.98) | 0.83(0.76-0.90) |
| Nicaragua | 116.52(54.40-236.92) | 399.91(185.91-820.78) | 243.22 | 7.28(3.44-15.10) | 8.63(4.05-18.00) | 0.56(0.47-0.65) |
| Niger | 231.81(106.36-473.28) | 687.24(315.90-1432.56) | 196.47 | 7.82(3.72-16.06) | 8.36(3.91-17.47) | 0.23(0.20-0.27) |
| Nigeria | 4211.88(1969.17-8673.78) | 8618.98(3992.75-17862.50) | 104.63 | 9.24(4.29-19.17) | 9.16(4.24-19.15) | -0.30(-0.52--0.08) |
| Niue | 0.20(0.10-0.42) | 0.25(0.12-0.52) | 24.00 | 9.51(4.49-19.52) | 11.68(5.55-24.06) | 0.64(0.49-0.80) |
| North Macedonia | 227.70(106.71-479.76) | 432.00(205.58-888.02) | 89.72 | 11.97(5.61-25.17) | 13.45(6.39-27.72) | 0.39(0.39-0.40) |
| Northern Mariana Islands | 2.20(1.01-4.47) | 6.39(2.97-13.08) | 190.13 | 10.54(4.89-21.80) | 11.40(5.34-23.08) | 0.12(-0.05-0.30) |
| Norway | 1255.47(601.02-2574.46) | 2067.54(979.95-4258.41) | 64.68 | 19.91(9.39-40.68) | 23.24(10.90-47.67) | 0.57(0.54-0.59) |
| Oman | 50.30(23.19-104.27) | 223.24(102.87-472.05) | 343.79 | 6.45(2.99-13.31) | 9.47(4.34-19.88) | 1.38(1.36-1.41) |
| Pakistan | 4468.00(2155.10-9116.90) | 11778.79(5682.38-23974.71) | 163.63 | 7.47(3.58-15.24) | 9.60(4.59-19.35) | 0.99(0.93-1.05) |
| Palau | 1.00(0.48-2.07) | 2.66(1.26-5.56) | 165.35 | 9.90(4.67-20.36) | 11.83(5.57-24.74) | 0.45(0.27-0.63) |
| Palestine | 62.98(29.01-130.53) | 206.56(96.35-431.09) | 227.98 | 7.09(3.28-14.68) | 7.99(3.75-16.57) | 0.30(0.23-0.36) |
| Panama | 104.33(48.18-216.75) | 366.82(171.35-761.91) | 251.60 | 6.82(3.21-13.97) | 8.83(4.13-18.39) | 0.79(0.74-0.83) |
| Papua New Guinea | 139.33(65.39-287.93) | 397.89(184.58-824.09) | 185.58 | 7.17(3.44-14.61) | 7.84(3.66-16.29) | 0.20(0.12-0.28) |
| Paraguay | 192.40(88.93-391.74) | 560.74(266.83-1141.06) | 191.44 | 8.48(3.95-17.31) | 9.89(4.70-20.15) | 0.52(0.49-0.55) |
| Peru | 1005.66(466.30-2080.50) | 3316.83(1532.39-6842.39) | 229.81 | 8.21(3.86-17.16) | 10.28(4.75-21.28) | 0.83(0.80-0.86) |
| Philippines | 2231.68(1047.82-4563.47) | 6618.61(3094.65-13545.58) | 196.57 | 7.09(3.31-14.59) | 8.12(3.80-16.85) | 0.46(0.45-0.48) |
| Poland | 5535.99(2598.48-11404.40) | 10112.83(4784.29-21058.91) | 82.67 | 12.68(5.94-26.03) | 15.20(7.16-31.54) | 0.63(0.62-0.64) |
| Portugal | 2477.89(1180.32-5056.58) | 4650.38(2240.33-9546.48) | 87.68 | 18.06(8.63-37.00) | 21.49(10.26-44.15) | 0.09(-0.09-0.26) |
| Puerto Rico | 422.53(199.28-869.27) | 899.11(434.47-1856.94) | 112.79 | 11.70(5.51-24.17) | 13.82(6.52-28.63) | 0.66(0.61-0.71) |
| Qatar | 17.77(8.31-37.94) | 184.46(85.82-386.69) | 938.06 | 9.79(4.58-20.02) | 11.58(5.45-24.12) | 0.52(0.43-0.61) |
| Republic of Korea | 4263.49(1961.28-8752.12) | 13659.86(6492.02-28490.65) | 220.39 | 13.78(6.39-28.08) | 15.26(7.25-31.70) | 0.34(0.24-0.43) |
| Republic of Moldova | 489.95(227.61-1023.68) | 784.93(370.70-1632.55) | 60.21 | 11.02(5.13-23.00) | 13.71(6.49-28.41) | 0.84(0.73-0.94) |
| Romania | 3350.48(1623.46-6927.20) | 4913.46(2339.48-10092.16) | 46.65 | 11.93(5.82-24.79) | 14.01(6.63-28.78) | 0.56(0.54-0.58) |
| Russian Federation | 21537.10(10052.12-44235.73) | 33247.77(15706.46-68870.98) | 54.37 | 11.93(5.59-24.47) | 14.37(6.76-29.71) | 0.66(0.64-0.68) |
| Rwanda | 265.87(124.29-553.34) | 642.06(297.74-1326.80) | 141.49 | 8.93(4.21-18.78) | 10.05(4.69-20.93) | 0.45(0.41-0.50) |
| Saint Kitts and Nevis | 3.41(1.63-7.13) | 7.91(3.72-16.53) | 131.60 | 9.50(4.56-19.78) | 11.42(5.39-23.78) | 0.63(0.58-0.67) |
| Saint Lucia | 7.66(3.58-15.95) | 23.01(10.65-47.39) | 200.31 | 8.73(4.09-18.18) | 10.60(4.93-21.76) | 0.62(0.56-0.68) |
| Saint Vincent and the Grenadines | 5.99(2.82-12.39) | 14.28(6.72-29.89) | 138.24 | 8.43(3.97-17.50) | 10.48(4.95-21.89) | 0.82(0.77-0.87) |
| Samoa | 8.80(4.07-18.34) | 15.93(7.53-32.66) | 81.14 | 9.91(4.59-20.55) | 10.69(5.06-21.80) | 0.10(-0.05-0.24) |
| San Marino | 6.83(3.21-14.02) | 14.02(6.77-28.98) | 105.32 | 21.20(9.93-43.22) | 23.99(11.55-49.13) | 0.43(0.37-0.49) |
| Sao Tome and Principe | 5.83(2.68-12.11) | 11.91(5.63-24.66) | 104.21 | 8.98(4.12-18.53) | 10.60(5.00-21.90) | 0.55(0.54-0.57) |
| Saudi Arabia | 498.96(227.80-1016.43) | 2334.82(1071.92-4855.58) | 367.94 | 7.55(3.52-15.43) | 10.30(4.84-21.29) | 1.04(1.00-1.08) |
| Senegal | 298.22(137.87-622.44) | 761.79(356.22-1582.30) | 155.45 | 8.95(4.19-18.73) | 9.74(4.55-20.12) | 0.23(0.19-0.26) |
| Serbia | 1432.28(690.22-2992.20) | 2144.53(1001.48-4498.46) | 49.73 | 12.38(5.95-25.81) | 14.13(6.71-29.45) | 0.50(0.48-0.52) |
| Seychelles | 4.59(2.12-9.48) | 10.64(4.97-21.83) | 131.75 | 8.17(3.76-16.97) | 9.32(4.37-19.11) | 0.40(0.36-0.43) |
| Sierra Leone | 158.32(73.25-329.69) | 346.38(164.92-719.94) | 118.78 | 8.12(3.74-16.77) | 9.17(4.40-19.12) | 0.38(0.35-0.42) |
| Singapore | 300.79(142.47-618.60) | 1268.94(590.95-2619.74) | 321.87 | 12.99(6.28-27.09) | 15.76(7.38-32.47) | 0.66(0.61-0.72) |
| Slovakia | 755.64(356.13-1569.72) | 1284.35(610.33-2674.11) | 69.97 | 12.74(5.99-26.31) | 14.29(6.74-29.56) | 0.31(0.27-0.35) |
| Slovenia | 299.68(143.11-622.37) | 573.81(276.08-1189.10) | 91.48 | 12.31(5.89-25.64) | 14.44(6.97-30.14) | 0.57(0.53-0.60) |
| Solomon Islands | 10.97(5.03-22.47) | 28.04(13.31-57.97) | 155.73 | 7.81(3.62-15.98) | 8.69(4.19-18.13) | 0.26(0.09-0.43) |
| Somalia | 235.87(110.69-492.55) | 642.64(293.84-1325.26) | 172.45 | 8.73(4.06-18.26) | 9.09(4.21-18.37) | 0.20(0.18-0.22) |
| South Africa | 2806.54(1330.07-5693.93) | 6898.01(3242.76-14259.48) | 145.78 | 13.10(6.16-26.92) | 14.83(6.93-30.76) | 0.47(0.43-0.52) |
| South Sudan | 220.03(101.41-462.03) | 404.51(191.10-827.65) | 83.85 | 8.95(4.21-18.86) | 9.83(4.70-20.29) | 0.36(0.34-0.38) |
| Spain | 11341.87(5511.72-23140.53) | 21403.33(10270.12-43528.88) | 88.71 | 21.35(10.33-42.97) | 24.62(11.69-50.53) | 0.38(0.24-0.52) |
| Sri Lanka | 761.96(361.37-1557.44) | 1962.51(937.72-4048.91) | 157.56 | 6.75(3.23-13.78) | 7.57(3.61-15.57) | 0.43(0.41-0.45) |
| Sudan | 551.78(256.08-1135.95) | 1575.24(740.18-3250.41) | 185.49 | 5.74(2.67-11.81) | 7.87(3.69-16.38) | 1.11(1.02-1.20) |
| Suriname | 24.11(11.02-49.94) | 66.77(31.99-138.56) | 176.95 | 9.12(4.15-19.02) | 10.92(5.20-22.48) | 0.66(0.63-0.68) |
| Sweden | 2103.81(1029.41-4290.54) | 4239.94(1986.90-8772.75) | 101.54 | 15.27(7.46-31.10) | 22.82(10.67-46.96) | 1.55(1.18-1.92) |
| Switzerland | 1922.18(909.19-3983.20) | 3427.43(1616.29-7083.93) | 78.31 | 19.40(9.12-40.39) | 21.50(10.08-44.51) | 0.36(0.34-0.37) |
| Syrian Arab Republic | 398.59(185.82-820.64) | 1091.51(505.63-2276.33) | 173.84 | 7.27(3.40-15.11) | 8.36(3.87-17.33) | 0.36(0.27-0.44) |
| Taiwan (Province of China) | 1115.35(504.89-2273.50) | 3074.12(1412.88-6373.63) | 175.62 | 6.75(3.08-13.90) | 7.87(3.63-16.14) | 0.68(0.60-0.76) |
| Tajikistan | 278.38(131.86-587.15) | 567.13(264.56-1186.06) | 103.73 | 9.83(4.66-20.82) | 10.47(4.88-21.61) | 0.15(0.12-0.19) |
| Thailand | 2497.29(1161.69-5194.14) | 9179.39(4239.26-18654.71) | 267.57 | 6.69(3.10-14.00) | 8.89(4.13-18.09) | 1.03(1.00-1.06) |
| Timor-Leste | 19.19(8.71-40.14) | 52.94(24.83-106.48) | 175.95 | 6.16(2.82-12.92) | 6.40(3.02-12.92) | 0.19(0.14-0.24) |
| Togo | 107.87(50.44-223.97) | 363.24(170.98-755.69) | 236.74 | 8.18(3.84-16.94) | 9.23(4.34-19.08) | 0.35(0.31-0.38) |
| Tokelau | 0.11(0.05-0.24) | 0.14(0.07-0.30) | 26.96 | 8.48(3.98-17.54) | 10.70(4.95-22.37) | 0.72(0.60-0.84) |
| Tonga | 5.34(2.52-11.27) | 8.57(4.11-17.85) | 60.44 | 9.44(4.43-19.64) | 10.78(5.17-22.17) | 0.21(-0.04-0.47) |
| Trinidad and Tobago | 82.11(38.48-167.89) | 208.80(97.97-431.45) | 154.29 | 9.61(4.52-19.83) | 11.17(5.28-22.98) | 0.62(0.56-0.68) |
| Tunisia | 364.11(164.93-760.20) | 1086.32(507.11-2235.92) | 198.35 | 7.01(3.18-14.57) | 8.41(3.95-17.21) | 0.60(0.58-0.63) |
| Turkey | 2792.41(1305.68-5687.09) | 8361.98(3946.44-17425.35) | 199.45 | 7.49(3.53-15.42) | 9.26(4.34-19.22) | 0.65(0.61-0.68) |
| Turkmenistan | 218.18(100.96-447.09) | 540.35(253.06-1127.38) | 147.66 | 11.25(5.30-22.81) | 13.36(6.28-27.75) | 0.55(0.52-0.58) |
| Tuvalu | 0.57(0.27-1.18) | 1.01(0.48-2.08) | 78.48 | 8.14(3.83-16.84) | 9.75(4.60-20.10) | 0.49(0.35-0.64) |
| Uganda | 585.02(273.28-1215.12) | 1470.91(677.14-3039.33) | 151.43 | 8.76(4.16-18.22) | 9.86(4.62-20.73) | 0.47(0.44-0.49) |
| Ukraine | 8820.69(4181.16-18400.51) | 10071.23(4829.63-20783.83) | 14.18 | 12.46(5.90-25.95) | 13.59(6.50-27.73) | 0.33(0.30-0.35) |
| United Arab Emirates | 54.28(25.19-116.65) | 696.11(316.47-1448.73) | 1182.39 | 7.95(3.67-16.69) | 10.37(4.91-21.08) | 0.95(0.92-0.97) |
| United Kingdom | 18948.88(9041.30-38938.90) | 29212.88(13921.56-60064.02) | 54.17 | 22.12(10.48-45.34) | 25.38(12.10-52.28) | 0.50(0.46-0.54) |
| United Republic of Tanzania | 1056.96(486.84-2190.59) | 2842.92(1311.35-5901.89) | 168.97 | 9.41(4.37-19.60) | 11.03(5.08-22.75) | 0.59(0.57-0.62) |
| United States of America | 78761.76(37919.90-163232.47) | 168667.02(84231.40-344196.52) | 114.15 | 25.60(12.26-52.77) | 31.72(15.90-64.24) | 0.68(0.58-0.78) |
| United States Virgin Islands | 10.28(4.86-21.26) | 23.66(11.04-50.05) | 130.22 | 11.57(5.47-23.92) | 13.10(6.07-27.72) | 0.42(0.36-0.49) |
| Uruguay | 621.70(295.40-1283.11) | 1082.71(512.15-2216.67) | 74.15 | 16.26(7.74-33.66) | 21.36(10.07-43.66) | 0.95(0.84-1.05) |
| Uzbekistan | 1224.75(573.29-2528.54) | 2868.55(1332.14-5869.61) | 134.22 | 10.72(5.09-22.12) | 12.72(5.99-26.07) | 0.56(0.51-0.60) |
| Vanuatu | 5.58(2.62-11.66) | 16.10(7.60-33.16) | 188.33 | 8.18(3.87-16.99) | 9.07(4.29-18.66) | 0.34(0.30-0.37) |
| Venezuela (Bolivarian Republic of) | 813.81(380.81-1672.67) | 2754.22(1307.98-5617.89) | 238.43 | 8.03(3.75-16.64) | 9.26(4.38-18.92) | 0.48(0.45-0.50) |
| Viet Nam | 2455.75(1149.23-5065.18) | 6527.26(3008.68-13355.15) | 165.80 | 6.04(2.85-12.52) | 6.75(3.09-13.68) | 0.44(0.40-0.48) |
| Yemen | 278.22(129.03-579.50) | 907.25(416.29-1884.50) | 226.09 | 5.34(2.52-11.04) | 6.28(2.93-12.94) | 0.64(0.61-0.66) |
| Zambia | 281.94(131.10-585.62) | 756.27(352.91-1556.06) | 168.23 | 9.52(4.45-19.75) | 10.61(4.97-22.14) | 0.35(0.31-0.39) |
| Zimbabwe | 459.31(213.16-946.75) | 809.76(379.64-1691.35) | 76.30 | 10.80(5.13-22.39) | 11.03(5.23-22.91) | -0.09(-0.17--0.02) |

S Table7 The DALY and age-standardized DALY rate of national hip osteoarthritis in female in 1990 and 2019, and its temporal trends from 1990 to 2019

| **Nation** | **DALY No. (95% UI)** | | **Change in absolute number (%)** | **Age-standardized DALY rate No.(95% UI)** | | **1990-2019 EAPC No. (95%CI)** |
| --- | --- | --- | --- | --- | --- | --- |
|  | **1990** | **2019** |  | **1990** | **2019** |  |
| Afghanistan | 176.12(82.12-365.54) | 423.98(197.62-884.49) | 140.74 | 5.06(2.40-10.46) | 6.07(2.90-12.45) | 0.83(0.68-0.97) |
| Albania | 110.78(52.31-233.94) | 265.43(125.66-552.03) | 139.59 | 10.20(4.78-21.58) | 12.10(5.79-25.21) | 0.67(0.64-0.70) |
| Algeria | 411.24(192.40-867.71) | 1451.03(686.63-3004.64) | 252.84 | 6.35(2.99-13.38) | 8.32(3.91-17.44) | 0.96(0.90-1.02) |
| American Samoa | 1.18(0.55-2.47) | 2.88(1.37-6.03) | 144.25 | 10.05(4.69-21.03) | 11.42(5.48-23.85) | 0.28(0.03-0.53) |
| Andorra | 5.68(2.67-11.91) | 17.12(8.24-35.24) | 201.57 | 20.86(9.82-43.77) | 24.92(12.01-51.42) | 0.59(0.49-0.69) |
| Angola | 170.70(79.32-354.77) | 625.57(290.77-1318.50) | 266.48 | 8.37(3.97-17.28) | 9.63(4.49-20.07) | 0.49(0.44-0.54) |
| Antigua and Barbuda | 2.48(1.14-5.12) | 5.32(2.53-10.98) | 114.45 | 8.49(3.92-17.70) | 9.71(4.60-20.06) | 0.44(0.42-0.46) |
| Argentina | 2841.78(1330.36-5860.49) | 6029.20(2884.91-12163.96) | 112.16 | 15.90(7.44-32.92) | 20.43(9.79-41.48) | 0.95(0.85-1.05) |
| Armenia | 155.17(72.59-323.86) | 282.04(133.48-574.89) | 81.76 | 10.02(4.72-20.91) | 11.97(5.72-24.16) | 0.65(0.63-0.67) |
| Australia | 1874.26(877.01-3922.98) | 4923.32(2374.63-9956.52) | 162.68 | 17.84(8.33-37.33) | 23.67(11.36-48.14) | 1.03(0.93-1.12) |
| Austria | 1402.74(677.06-2861.72) | 2163.63(1034.63-4406.21) | 54.24 | 20.44(9.86-42.03) | 23.97(11.38-49.35) | 0.54(0.50-0.57) |
| Azerbaijan | 302.29(143.36-630.05) | 665.20(307.28-1420.86) | 120.06 | 10.33(4.87-21.30) | 12.39(5.81-26.21) | 0.66(0.59-0.73) |
| Bahamas | 8.38(3.95-17.43) | 22.82(10.66-47.07) | 172.27 | 9.56(4.61-19.83) | 10.46(4.92-21.59) | 0.31(0.26-0.35) |
| Bahrain | 6.96(3.20-14.68) | 43.38(20.47-89.48) | 523.36 | 7.76(3.59-16.41) | 9.47(4.47-19.36) | 0.66(0.62-0.70) |
| Bangladesh | 1761.94(822.88-3680.54) | 6435.80(3049.13-13352.56) | 265.27 | 7.68(3.53-15.91) | 9.65(4.60-20.03) | 0.90(0.85-0.95) |
| Barbados | 14.99(7.09-31.77) | 27.50(13.04-56.50) | 83.41 | 9.43(4.44-19.75) | 10.52(4.98-21.73) | 0.40(0.37-0.43) |
| Belarus | 858.39(402.05-1799.96) | 1195.75(572.42-2475.84) | 39.30 | 10.62(5.07-22.23) | 12.53(5.98-25.86) | 0.65(0.61-0.69) |
| Belgium | 1711.46(802.22-3511.83) | 2649.29(1255.61-5515.20) | 54.80 | 20.21(9.49-41.49) | 23.40(11.09-48.32) | 0.49(0.45-0.52) |
| Belize | 4.17(1.99-8.66) | 15.50(7.24-32.31) | 271.90 | 8.96(4.25-18.79) | 10.91(5.22-22.65) | 0.67(0.49-0.85) |
| Benin | 85.75(38.61-178.65) | 268.75(125.96-556.93) | 213.41 | 8.04(3.70-16.84) | 9.89(4.69-20.72) | 0.76(0.68-0.83) |
| Bermuda | 3.91(1.91-8.12) | 7.74(3.66-16.16) | 98.00 | 11.10(5.41-23.13) | 11.52(5.52-23.79) | 0.11(0.06-0.16) |
| Bhutan | 11.60(5.64-24.00) | 33.45(16.26-68.76) | 188.47 | 8.57(4.16-18.17) | 11.57(5.59-23.70) | 1.14(1.07-1.22) |
| Bolivia (Plurinational State of) | 128.28(60.41-267.68) | 412.82(194.79-849.74) | 221.81 | 7.32(3.37-15.18) | 8.75(4.14-17.92) | 0.65(0.62-0.69) |
| Bosnia and Herzegovina | 237.26(111.86-488.57) | 387.97(185.87-800.80) | 63.52 | 10.20(4.82-20.94) | 12.18(5.81-25.28) | 0.68(0.59-0.76) |
| Botswana | 29.30(13.49-60.13) | 91.26(42.85-188.73) | 211.43 | 8.98(4.18-18.64) | 11.22(5.31-23.46) | 0.70(0.64-0.75) |
| Brazil | 4058.98(1879.59-8399.23) | 12841.73(6048.77-26658.02) | 216.38 | 8.31(3.89-17.24) | 9.82(4.63-20.39) | 0.58(0.57-0.60) |
| Brunei Darussalam | 6.31(2.98-13.07) | 25.20(11.77-52.96) | 299.56 | 12.48(5.95-25.95) | 15.17(7.17-31.14) | 0.77(0.69-0.86) |
| Bulgaria | 808.36(379.81-1663.99) | 1029.20(486.54-2152.74) | 27.32 | 12.16(5.71-25.05) | 13.37(6.24-28.06) | 0.26(0.22-0.30) |
| Burkina Faso | 179.55(84.06-370.79) | 467.91(212.09-976.86) | 160.60 | 7.57(3.55-15.65) | 8.87(4.12-18.47) | 0.58(0.57-0.59) |
| Burundi | 107.36(50.18-220.54) | 187.93(86.12-389.51) | 75.04 | 8.26(3.90-16.98) | 8.15(3.81-16.79) | -0.03(-0.06-0.00) |
| Cabo Verde | 10.69(4.88-22.31) | 24.50(11.39-51.23) | 129.31 | 8.34(3.81-17.46) | 10.14(4.71-21.17) | 0.69(0.68-0.70) |
| Cambodia | 152.94(71.14-316.46) | 442.41(209.10-927.29) | 189.28 | 5.80(2.71-12.10) | 6.26(2.96-13.07) | 0.30(0.27-0.32) |
| Cameroon | 234.06(107.82-484.69) | 711.74(323.64-1450.00) | 204.08 | 9.85(4.55-20.28) | 10.93(5.03-22.22) | 0.34(0.30-0.38) |
| Canada | 2552.23(1209.10-5258.73) | 6606.08(3138.22-13599.43) | 158.84 | 14.43(6.82-29.61) | 19.02(8.96-38.97) | 0.59(0.41-0.77) |
| Central African Republic | 53.29(24.69-110.66) | 105.04(49.04-216.42) | 97.11 | 8.27(3.90-17.08) | 8.59(4.09-17.77) | 0.14(0.12-0.16) |
| Chad | 106.89(50.26-221.20) | 215.85(99.88-444.63) | 101.94 | 7.20(3.45-14.76) | 7.81(3.65-16.38) | 0.28(0.27-0.30) |
| Chile | 924.00(426.91-1904.44) | 2803.52(1336.82-5868.17) | 203.41 | 16.84(7.74-34.79) | 21.48(10.18-44.60) | 0.87(0.77-0.97) |
| China | 22563.20(10314.54-46242.61) | 71895.49(33315.78-148629.28) | 218.64 | 5.09(2.33-10.39) | 6.85(3.18-14.11) | 1.18(1.05-1.32) |
| Colombia | 673.64(304.57-1393.91) | 2391.15(1140.43-4900.31) | 254.96 | 7.15(3.26-14.98) | 8.33(3.98-17.01) | 0.52(0.49-0.55) |
| Comoros | 10.34(4.86-21.17) | 25.47(11.99-53.04) | 146.44 | 8.94(4.24-18.56) | 9.54(4.51-19.78) | 0.23(0.21-0.26) |
| Congo | 57.37(26.62-119.06) | 153.38(72.02-320.39) | 167.36 | 9.47(4.44-19.51) | 10.76(5.06-21.88) | 0.42(0.41-0.44) |
| Cook Islands | 0.57(0.27-1.16) | 1.45(0.69-3.02) | 155.52 | 9.28(4.42-18.95) | 11.65(5.59-24.26) | 0.65(0.49-0.81) |
| Costa Rica | 70.21(33.09-145.84) | 247.06(116.28-510.98) | 251.87 | 7.62(3.60-15.81) | 8.87(4.17-18.23) | 0.50(0.47-0.53) |
| C么te d'Ivoire | 168.76(78.94-344.50) | 514.55(240.62-1079.71) | 204.89 | 8.37(3.92-17.36) | 9.42(4.35-19.59) | 0.37(0.35-0.39) |
| Croatia | 425.13(204.42-884.43) | 601.93(286.97-1255.29) | 41.59 | 11.40(5.48-23.51) | 13.22(6.37-27.70) | 0.58(0.54-0.63) |
| Cuba | 435.01(200.98-903.01) | 956.68(455.52-2019.30) | 119.92 | 8.39(3.90-17.47) | 9.83(4.63-20.82) | 0.61(0.56-0.66) |
| Cyprus | 81.46(39.86-167.95) | 226.24(106.98-463.67) | 177.74 | 18.51(9.07-38.18) | 22.60(10.71-46.08) | 0.75(0.69-0.82) |
| Czechia | 915.64(432.27-1911.01) | 1457.79(690.64-3042.12) | 59.21 | 11.69(5.45-24.30) | 13.55(6.41-28.14) | 0.51(0.47-0.54) |
| Democratic People's Republic of Korea | 523.81(244.99-1052.31) | 994.87(463.43-2057.12) | 89.93 | 5.32(2.49-10.67) | 5.39(2.50-11.18) | 0.04(0.02-0.06) |
| Democratic Republic of the Congo | 780.96(361.47-1633.51) | 1795.38(858.60-3705.27) | 129.90 | 8.84(4.14-18.35) | 8.89(4.23-18.29) | -0.11(-0.18--0.04) |
| Denmark | 1227.09(586.46-2481.46) | 1447.15(684.00-2929.61) | 17.93 | 28.47(13.56-57.40) | 25.63(12.24-52.69) | -0.45(-0.70--0.20) |
| Djibouti | 6.24(2.90-12.91) | 30.05(13.56-62.14) | 381.67 | 8.41(3.94-17.46) | 10.04(4.69-20.90) | 0.69(0.66-0.73) |
| Dominica | 3.39(1.59-7.05) | 4.34(2.05-9.11) | 28.21 | 8.59(3.98-17.79) | 9.73(4.57-20.17) | 0.42(0.38-0.47) |
| Dominican Republic | 157.59(74.01-324.44) | 484.67(226.59-1023.18) | 207.55 | 8.12(3.82-17.03) | 9.99(4.68-20.98) | 0.81(0.76-0.86) |
| Ecuador | 257.46(119.68-527.99) | 841.94(395.28-1749.37) | 227.02 | 9.27(4.32-19.10) | 10.56(4.98-21.98) | 0.32(0.26-0.39) |
| Egypt | 1074.07(500.44-2259.76) | 2742.38(1241.06-5690.30) | 155.33 | 7.04(3.29-14.88) | 8.76(4.08-18.19) | 0.55(0.46-0.64) |
| El Salvador | 115.06(53.44-237.98) | 289.36(138.10-611.23) | 151.48 | 7.17(3.33-14.81) | 8.55(4.07-18.08) | 0.61(0.51-0.72) |
| Equatorial Guinea | 9.40(4.47-19.73) | 34.50(16.10-71.52) | 267.09 | 8.33(3.97-17.40) | 11.73(5.50-23.99) | 1.41(1.30-1.52) |
| Eritrea | 46.30(21.25-97.24) | 132.50(62.02-268.65) | 186.16 | 7.82(3.63-16.13) | 8.36(3.98-17.04) | 0.23(0.15-0.30) |
| Estonia | 142.02(65.96-291.28) | 193.63(91.02-398.20) | 36.34 | 11.12(5.17-23.14) | 13.20(6.22-28.04) | 0.74(0.68-0.80) |
| Eswatini | 18.29(8.72-38.57) | 42.64(19.70-89.30) | 133.11 | 10.76(5.07-22.52) | 12.07(5.60-25.32) | 0.18(-0.04-0.40) |
| Ethiopia | 850.03(397.65-1742.40) | 1941.79(913.57-3993.94) | 128.44 | 8.53(4.03-17.30) | 9.02(4.26-18.67) | 0.22(0.19-0.24) |
| Fiji | 15.74(7.50-33.36) | 40.14(19.11-83.84) | 155.01 | 8.15(3.86-17.02) | 9.96(4.75-20.55) | 0.56(0.44-0.67) |
| Finland | 869.96(418.21-1784.39) | 1497.19(717.57-3043.48) | 72.10 | 21.03(10.07-43.10) | 24.72(12.03-50.48) | 0.57(0.51-0.63) |
| France | 9685.33(4582.93-19550.75) | 16915.60(8114.50-34719.24) | 74.65 | 21.23(10.08-42.76) | 24.82(11.89-50.89) | 0.65(0.37-0.93) |
| Gabon | 30.83(14.60-64.12) | 72.26(33.87-148.77) | 134.36 | 9.92(4.70-20.73) | 12.57(5.89-25.94) | 0.75(0.69-0.82) |
| Gambia | 14.02(6.58-28.47) | 47.88(22.17-98.11) | 241.61 | 8.07(3.78-16.64) | 9.36(4.42-19.50) | 0.49(0.48-0.51) |
| Georgia | 434.44(202.53-889.56) | 416.14(196.16-857.20) | -4.21 | 11.77(5.51-24.07) | 12.17(5.77-25.39) | 0.11(0.09-0.13) |
| Germany | 15282.19(7239.23-31480.67) | 22762.19(10788.50-46963.30) | 48.95 | 20.78(9.88-42.65) | 24.31(11.61-50.10) | 0.53(0.47-0.58) |
| Ghana | 274.37(128.37-566.96) | 938.86(437.64-1945.48) | 242.18 | 8.08(3.78-16.90) | 9.98(4.71-20.63) | 0.72(0.68-0.76) |
| Greece | 1529.52(735.76-3152.93) | 2518.66(1198.42-5187.90) | 64.67 | 18.95(9.10-38.71) | 21.91(10.65-45.37) | 1.30(0.95-1.65) |
| Greenland | 2.55(1.21-5.25) | 6.17(2.93-12.70) | 142.42 | 14.89(7.10-31.03) | 18.89(8.95-38.75) | 0.84(0.77-0.91) |
| Grenada | 3.11(1.49-6.44) | 5.38(2.51-11.28) | 73.31 | 7.82(3.70-16.29) | 9.19(4.29-19.27) | 0.54(0.49-0.60) |
| Guam | 3.73(1.74-7.71) | 10.95(5.11-22.65) | 193.78 | 9.41(4.43-19.44) | 11.29(5.28-23.19) | 0.65(0.60-0.71) |
| Guatemala | 121.78(59.23-248.92) | 464.81(221.45-970.88) | 281.68 | 6.31(3.05-12.90) | 7.43(3.56-15.34) | 0.57(0.53-0.61) |
| Guinea | 132.32(61.28-278.66) | 244.09(115.21-510.23) | 84.47 | 7.80(3.67-16.39) | 8.55(4.09-17.77) | 0.31(0.29-0.33) |
| Guinea-Bissau | 16.86(7.86-34.52) | 35.18(16.23-71.76) | 108.67 | 7.76(3.67-16.08) | 8.47(3.93-17.29) | 0.25(0.23-0.28) |
| Guyana | 16.27(7.77-33.65) | 31.46(14.68-65.01) | 93.30 | 8.01(3.82-16.72) | 9.27(4.34-19.11) | 0.49(0.44-0.55) |
| Haiti | 114.92(54.03-237.33) | 279.34(129.33-582.03) | 143.08 | 6.67(3.14-13.69) | 7.20(3.39-15.13) | 0.34(0.29-0.38) |
| Honduras | 71.94(33.07-151.16) | 258.47(116.29-528.62) | 259.30 | 6.61(3.05-13.97) | 7.77(3.53-16.12) | 0.59(0.52-0.66) |
| Hungary | 1037.21(491.43-2137.68) | 1470.67(692.62-3014.43) | 41.79 | 12.35(5.83-25.37) | 13.96(6.61-28.71) | 0.42(0.38-0.45) |
| Iceland | 38.75(18.68-77.51) | 74.44(35.49-151.87) | 92.12 | 26.22(12.67-52.61) | 27.25(12.98-55.89) | 0.01(-0.09-0.11) |
| India | 19300.87(9234.35-39038.54) | 61824.71(30020.90-124738.26) | 220.32 | 8.06(3.89-16.18) | 10.20(4.94-20.61) | 1.33(1.08-1.58) |
| Indonesia | 3350.43(1550.06-6893.96) | 8381.92(3870.62-17284.34) | 150.17 | 6.26(2.94-12.77) | 7.10(3.34-14.72) | 0.46(0.44-0.49) |
| Iran (Islamic Republic of) | 814.53(377.71-1660.46) | 2941.64(1384.01-5982.78) | 261.15 | 6.20(2.89-12.66) | 7.61(3.58-15.63) | 0.63(0.45-0.81) |
| Iraq | 297.82(141.29-613.27) | 1010.57(471.27-2071.00) | 239.33 | 7.31(3.45-15.03) | 8.15(3.80-16.83) | 0.40(0.34-0.45) |
| Ireland | 449.61(212.55-935.13) | 920.21(442.01-1866.57) | 104.67 | 20.70(9.83-43.43) | 24.34(11.66-49.60) | 0.55(0.50-0.60) |
| Israel | 501.61(235.79-1011.42) | 1362.42(647.99-2841.13) | 171.61 | 19.40(9.08-39.40) | 22.76(10.86-47.07) | 0.51(0.44-0.58) |
| Italy | 9877.31(4735.36-20253.83) | 15909.42(7523.11-32785.77) | 61.07 | 20.23(9.65-40.98) | 22.72(10.76-46.31) | 0.56(0.42-0.71) |
| Jamaica | 77.40(36.76-162.64) | 150.50(70.10-311.99) | 94.44 | 8.33(3.94-17.50) | 9.74(4.53-20.23) | 0.61(0.55-0.67) |
| Japan | 11695.06(5555.25-23884.28) | 22998.16(10903.13-47530.64) | 96.65 | 12.35(5.87-25.20) | 13.83(6.57-28.34) | 0.41(0.19-0.62) |
| Jordan | 49.21(22.77-105.51) | 307.44(145.78-638.51) | 524.74 | 7.02(3.33-14.80) | 9.08(4.21-18.80) | 0.95(0.91-0.99) |
| Kazakhstan | 916.93(430.79-1925.04) | 1382.96(644.08-2850.28) | 50.83 | 11.84(5.51-24.85) | 13.43(6.28-27.69) | 0.36(0.33-0.39) |
| Kenya | 397.09(186.42-818.50) | 1163.46(547.72-2397.13) | 193.00 | 9.08(4.23-18.96) | 9.48(4.46-19.80) | 0.15(0.14-0.15) |
| Kiribati | 1.59(0.74-3.28) | 3.50(1.63-7.46) | 120.93 | 7.66(3.59-15.75) | 8.78(4.15-18.48) | 0.34(0.13-0.54) |
| Kuwait | 22.63(10.56-46.91) | 141.91(66.68-294.37) | 526.96 | 8.25(3.92-16.85) | 10.30(4.78-21.26) | 0.87(0.83-0.91) |
| Kyrgyzstan | 181.56(85.71-380.12) | 290.27(137.40-602.03) | 59.88 | 10.03(4.69-20.95) | 10.99(5.23-22.77) | 0.23(0.19-0.26) |
| Lao People's Democratic Republic | 66.13(30.79-140.48) | 160.33(74.33-338.33) | 142.44 | 5.94(2.81-12.65) | 6.78(3.12-14.30) | 0.49(0.46-0.52) |
| Latvia | 251.02(118.22-524.65) | 296.87(141.28-610.52) | 18.27 | 11.25(5.35-23.51) | 13.06(6.22-27.22) | 0.61(0.56-0.66) |
| Lebanon | 79.44(36.61-164.95) | 248.51(113.39-517.82) | 212.83 | 6.74(3.14-13.71) | 8.71(3.98-18.14) | 0.65(0.32-0.99) |
| Lesotho | 48.23(22.49-99.52) | 75.69(35.58-156.40) | 56.93 | 8.65(4.03-17.96) | 9.99(4.75-20.71) | 0.50(0.45-0.55) |
| Liberia | 43.85(20.05-90.31) | 106.11(49.68-222.47) | 141.99 | 8.35(3.89-17.20) | 9.91(4.65-20.30) | 0.80(0.68-0.92) |
| Libya | 67.05(30.83-138.59) | 233.87(111.07-484.00) | 248.78 | 7.49(3.45-15.56) | 8.69(4.13-18.14) | 0.54(0.49-0.58) |
| Lithuania | 299.32(142.95-631.62) | 414.81(194.15-852.70) | 38.58 | 10.98(5.22-23.07) | 12.69(5.95-25.74) | 0.60(0.54-0.66) |
| Luxembourg | 64.43(30.67-133.19) | 122.12(58.08-248.01) | 89.55 | 20.85(9.86-43.21) | 24.20(11.47-48.87) | 0.52(0.48-0.56) |
| Madagascar | 214.31(98.19-443.47) | 505.61(234.14-1059.51) | 135.92 | 8.24(3.86-17.21) | 8.46(4.00-17.84) | 0.12(0.09-0.14) |
| Malawi | 182.35(86.31-372.88) | 394.08(186.25-804.84) | 116.11 | 8.64(4.13-17.70) | 9.49(4.49-19.57) | 0.41(0.33-0.48) |
| Malaysia | 349.15(165.96-713.79) | 1183.06(550.32-2467.48) | 238.84 | 7.07(3.33-14.39) | 8.58(3.97-18.00) | 0.72(0.70-0.74) |
| Maldives | 2.45(1.13-5.20) | 11.49(5.25-23.66) | 368.05 | 6.11(2.82-12.55) | 7.81(3.60-16.02) | 1.00(0.90-1.11) |
| Mali | 158.63(75.04-333.43) | 365.18(168.25-766.47) | 130.20 | 7.39(3.51-15.39) | 8.35(3.93-17.44) | 0.49(0.47-0.51) |
| Malta | 47.61(22.31-95.82) | 112.73(54.49-231.02) | 136.77 | 20.15(9.45-40.72) | 24.50(11.77-49.81) | 0.64(0.53-0.76) |
| Marshall Islands | 0.59(0.28-1.23) | 1.47(0.68-3.02) | 148.79 | 6.88(3.26-14.36) | 8.21(3.92-16.99) | 0.50(0.40-0.60) |
| Mauritania | 49.37(22.80-102.33) | 116.72(52.97-241.40) | 136.43 | 9.25(4.27-19.03) | 10.79(4.96-22.61) | 0.48(0.43-0.53) |
| Mauritius | 29.29(13.43-60.82) | 79.61(37.42-166.46) | 171.83 | 7.14(3.31-14.83) | 8.40(3.93-17.45) | 0.56(0.54-0.58) |
| Mexico | 1870.14(879.56-3805.64) | 5756.56(2713.29-11908.23) | 207.81 | 8.06(3.78-16.56) | 9.03(4.25-18.71) | -0.05(-0.21-0.10) |
| Micronesia (Federated States of) | 1.93(0.91-4.03) | 3.50(1.65-7.24) | 81.40 | 8.09(3.83-16.76) | 9.25(4.36-19.08) | 0.33(0.15-0.52) |
| Monaco | 8.49(4.06-17.35) | 12.09(5.66-25.02) | 42.40 | 23.19(11.05-47.97) | 26.36(12.19-54.21) | 0.42(0.37-0.47) |
| Mongolia | 56.02(26.41-116.23) | 149.88(68.44-312.35) | 167.56 | 9.82(4.68-20.45) | 11.01(5.07-22.65) | 0.40(0.36-0.44) |
| Montenegro | 41.90(19.36-87.13) | 68.73(32.49-144.54) | 64.03 | 12.04(5.58-24.98) | 13.08(6.21-27.39) | 0.33(0.29-0.37) |
| Morocco | 434.49(202.68-909.85) | 1262.54(602.93-2593.14) | 190.58 | 6.08(2.88-12.48) | 7.71(3.67-15.55) | 0.80(0.77-0.83) |
| Mozambique | 271.92(125.85-564.43) | 566.26(261.56-1163.62) | 108.24 | 8.29(3.83-17.19) | 8.97(4.24-18.62) | 0.32(0.28-0.36) |
| Myanmar | 741.28(345.30-1503.31) | 1790.46(841.01-3741.48) | 141.54 | 5.89(2.77-12.05) | 6.69(3.17-13.77) | 0.51(0.46-0.55) |
| Namibia | 34.90(16.46-72.60) | 80.63(37.31-167.50) | 131.02 | 8.81(4.17-18.33) | 9.77(4.63-20.21) | 0.32(0.25-0.39) |
| Nauru | 0.16(0.07-0.34) | 0.24(0.11-0.50) | 47.53 | 8.39(3.95-17.56) | 9.64(4.54-20.12) | 0.31(0.23-0.38) |
| Nepal | 388.79(184.82-802.86) | 1303.12(618.42-2662.27) | 235.18 | 7.76(3.73-16.05) | 10.55(5.00-21.62) | 1.16(1.09-1.23) |
| Netherlands | 2674.71(1276.34-5373.61) | 4141.00(1988.11-8522.35) | 54.82 | 24.16(11.63-48.54) | 24.57(11.70-50.25) | 0.02(-0.14-0.19) |
| New Zealand | 376.63(180.58-773.34) | 891.96(426.89-1819.91) | 136.83 | 17.87(8.52-37.06) | 22.64(10.78-46.06) | 0.91(0.83-0.98) |
| Nicaragua | 57.74(26.86-120.57) | 203.31(95.34-421.25) | 252.11 | 6.83(3.14-14.29) | 8.06(3.81-16.46) | 0.54(0.45-0.64) |
| Niger | 105.56(48.04-216.30) | 335.61(151.69-705.49) | 217.95 | 7.48(3.46-15.48) | 7.94(3.67-16.56) | 0.23(0.21-0.25) |
| Nigeria | 1848.88(864.72-3771.24) | 4329.16(2010.19-9044.04) | 134.15 | 8.71(4.03-17.92) | 8.66(4.02-17.98) | -0.23(-0.39--0.07) |
| Niue | 0.11(0.05-0.22) | 0.12(0.06-0.26) | 18.15 | 8.71(4.12-17.93) | 10.80(5.16-22.08) | 0.69(0.54-0.84) |
| North Macedonia | 109.85(52.60-231.65) | 211.35(100.55-442.70) | 92.39 | 11.11(5.35-23.49) | 12.72(6.03-26.52) | 0.49(0.48-0.50) |
| Northern Mariana Islands | 0.78(0.36-1.61) | 2.85(1.31-5.98) | 266.01 | 9.40(4.30-19.61) | 10.51(4.83-21.45) | 0.23(0.06-0.40) |
| Norway | 732.62(354.20-1484.22) | 1144.58(546.28-2347.13) | 56.23 | 20.64(9.89-42.20) | 24.53(11.57-50.38) | 0.67(0.65-0.69) |
| Oman | 18.58(8.72-38.63) | 74.94(35.02-159.78) | 303.43 | 5.89(2.77-12.19) | 8.83(4.07-18.51) | 1.46(1.42-1.50) |
| Pakistan | 2484.04(1206.92-5034.22) | 6819.32(3262.24-13929.11) | 174.53 | 9.06(4.39-18.33) | 11.40(5.49-23.15) | 0.91(0.86-0.96) |
| Palau | 0.46(0.21-0.96) | 1.19(0.56-2.57) | 160.49 | 9.00(4.20-18.70) | 10.85(5.09-22.78) | 0.51(0.34-0.68) |
| Palestine | 32.02(14.76-66.60) | 98.59(46.52-203.55) | 207.92 | 6.60(3.07-13.77) | 7.55(3.59-15.39) | 0.37(0.29-0.44) |
| Panama | 48.15(22.33-99.45) | 174.40(82.51-362.64) | 262.21 | 6.37(2.95-13.22) | 8.20(3.88-17.08) | 0.77(0.74-0.81) |
| Papua New Guinea | 60.88(28.50-128.09) | 172.78(81.09-358.55) | 183.82 | 6.52(3.11-13.65) | 7.19(3.41-14.97) | 0.24(0.17-0.31) |
| Paraguay | 95.85(44.65-198.53) | 273.22(126.79-563.13) | 185.05 | 8.15(3.75-16.82) | 9.33(4.29-19.25) | 0.46(0.44-0.48) |
| Peru | 492.71(228.16-1021.93) | 1585.15(735.96-3321.51) | 221.72 | 7.89(3.73-16.44) | 9.49(4.35-19.98) | 0.69(0.66-0.72) |
| Philippines | 1083.01(506.91-2214.80) | 3173.33(1475.87-6519.26) | 193.01 | 6.69(3.12-13.75) | 7.39(3.44-15.23) | 0.34(0.32-0.36) |
| Poland | 2813.83(1336.83-5787.60) | 5273.46(2513.20-10983.97) | 87.41 | 11.15(5.22-23.00) | 13.99(6.62-29.10) | 0.82(0.80-0.85) |
| Portugal | 1445.87(693.02-2988.40) | 2717.87(1320.22-5557.54) | 87.97 | 18.59(8.92-38.49) | 22.19(10.74-45.29) | 0.09(-0.09-0.27) |
| Puerto Rico | 214.98(100.53-441.09) | 465.24(222.71-962.94) | 116.41 | 11.08(5.13-22.69) | 12.88(6.04-26.84) | 0.61(0.56-0.66) |
| Qatar | 4.33(2.01-8.91) | 37.82(17.74-79.50) | 774.44 | 8.81(4.13-18.08) | 10.50(4.96-21.64) | 0.58(0.51-0.65) |
| Republic of Korea | 2414.96(1127.03-4913.66) | 7442.10(3510.16-15274.85) | 208.17 | 13.92(6.49-28.12) | 15.52(7.32-31.70) | 0.43(0.30-0.56) |
| Republic of Moldova | 259.76(121.17-542.03) | 420.74(197.74-880.93) | 61.97 | 9.96(4.67-20.61) | 12.56(5.88-25.64) | 0.92(0.81-1.03) |
| Romania | 1719.01(830.18-3563.09) | 2644.06(1250.25-5424.92) | 53.81 | 11.15(5.43-23.11) | 13.37(6.33-27.41) | 0.65(0.62-0.68) |
| Russian Federation | 11445.84(5423.69-23635.17) | 17248.62(8140.28-35676.80) | 50.70 | 9.97(4.67-20.72) | 12.20(5.74-25.35) | 0.81(0.76-0.86) |
| Rwanda | 140.07(65.57-289.73) | 334.82(155.00-682.92) | 139.04 | 8.57(4.03-17.97) | 9.27(4.32-19.07) | 0.32(0.29-0.35) |
| Saint Kitts and Nevis | 1.80(0.86-3.73) | 3.73(1.73-7.81) | 107.93 | 9.02(4.28-18.77) | 10.60(4.96-22.21) | 0.56(0.51-0.60) |
| Saint Lucia | 4.01(1.84-8.49) | 11.10(5.12-23.18) | 176.64 | 8.27(3.83-17.41) | 9.80(4.50-20.45) | 0.54(0.49-0.59) |
| Saint Vincent and the Grenadines | 3.13(1.48-6.48) | 6.40(3.05-13.16) | 104.42 | 7.99(3.73-16.44) | 9.69(4.62-19.85) | 0.73(0.68-0.78) |
| Samoa | 4.05(1.86-8.32) | 7.34(3.47-14.83) | 81.26 | 9.01(4.19-18.55) | 9.82(4.71-19.67) | 0.13(-0.01-0.27) |
| San Marino | 3.84(1.79-7.86) | 7.78(3.80-16.01) | 102.62 | 22.00(10.30-44.70) | 25.07(11.96-51.53) | 0.47(0.41-0.53) |
| Sao Tome and Principe | 2.94(1.35-6.18) | 5.77(2.71-11.93) | 96.21 | 8.67(4.01-18.15) | 10.07(4.80-20.55) | 0.54(0.53-0.56) |
| Saudi Arabia | 180.28(83.35-373.47) | 845.75(396.13-1785.30) | 369.12 | 6.86(3.17-14.13) | 9.60(4.71-19.91) | 1.14(1.13-1.16) |
| Senegal | 141.92(64.49-292.38) | 374.90(174.64-773.17) | 164.16 | 8.58(3.95-17.66) | 9.26(4.41-18.87) | 0.22(0.19-0.25) |
| Serbia | 711.76(331.93-1450.34) | 1103.37(512.85-2334.88) | 55.02 | 11.51(5.35-23.31) | 13.45(6.33-28.19) | 0.59(0.56-0.62) |
| Seychelles | 2.43(1.13-5.02) | 5.05(2.38-10.49) | 108.12 | 7.80(3.62-16.20) | 8.71(4.15-18.04) | 0.32(0.28-0.36) |
| Sierra Leone | 74.00(34.83-153.04) | 162.63(77.34-336.41) | 119.78 | 7.78(3.66-15.97) | 8.71(4.19-18.08) | 0.37(0.33-0.42) |
| Singapore | 155.50(74.01-327.76) | 622.52(297.16-1263.65) | 300.33 | 12.75(6.08-26.53) | 15.41(7.37-31.28) | 0.72(0.66-0.78) |
| Slovakia | 401.73(188.14-825.65) | 690.50(319.80-1426.10) | 71.88 | 11.90(5.50-24.31) | 13.64(6.37-28.32) | 0.39(0.35-0.43) |
| Slovenia | 165.68(79.22-343.95) | 297.54(142.19-614.93) | 79.58 | 11.55(5.51-24.20) | 13.71(6.53-28.19) | 0.62(0.58-0.65) |
| Solomon Islands | 4.41(2.03-9.16) | 12.71(6.01-26.64) | 188.51 | 6.99(3.22-14.17) | 7.99(3.80-16.83) | 0.36(0.19-0.53) |
| Somalia | 117.71(55.00-241.99) | 325.61(150.99-664.01) | 176.63 | 8.33(3.90-17.20) | 8.34(3.88-16.99) | 0.07(0.03-0.11) |
| South Africa | 1153.80(543.89-2348.46) | 2654.84(1245.26-5459.87) | 130.10 | 9.54(4.48-19.50) | 10.11(4.76-20.82) | 0.23(0.19-0.28) |
| South Sudan | 91.53(42.39-191.61) | 175.47(82.43-366.98) | 91.71 | 8.46(3.95-17.73) | 8.85(4.26-18.02) | 0.19(0.15-0.23) |
| Spain | 6649.15(3200.26-13635.99) | 12250.82(5865.09-24641.58) | 84.25 | 22.17(10.58-45.09) | 25.58(12.20-51.77) | 0.38(0.22-0.55) |
| Sri Lanka | 359.26(170.83-745.51) | 1009.52(475.20-2085.93) | 181.00 | 6.39(3.06-13.43) | 7.10(3.34-14.63) | 0.40(0.38-0.42) |
| Sudan | 242.55(109.29-498.03) | 691.03(314.75-1415.70) | 184.89 | 5.27(2.40-10.96) | 7.35(3.41-15.08) | 1.18(1.08-1.27) |
| Suriname | 11.81(5.29-25.02) | 33.04(15.77-67.14) | 179.72 | 8.61(3.87-18.12) | 10.13(4.84-20.60) | 0.61(0.58-0.64) |
| Sweden | 984.87(481.38-2012.11) | 2124.38(986.08-4376.62) | 115.70 | 12.72(6.30-26.24) | 21.58(10.01-44.38) | 2.26(1.78-2.75) |
| Switzerland | 1135.94(538.93-2334.65) | 1914.37(902.14-3936.33) | 68.53 | 20.05(9.50-41.57) | 22.37(10.59-45.88) | 0.39(0.37-0.41) |
| Syrian Arab Republic | 174.90(81.04-364.78) | 506.21(235.23-1049.07) | 189.43 | 6.68(3.14-13.83) | 7.86(3.66-16.39) | 0.46(0.39-0.52) |
| Taiwan (Province of China) | 485.71(221.51-996.02) | 1520.88(703.96-3186.91) | 213.13 | 6.28(2.87-12.93) | 7.39(3.40-15.27) | 0.72(0.64-0.80) |
| Tajikistan | 141.94(66.94-296.74) | 268.44(120.97-546.80) | 89.13 | 9.10(4.29-19.18) | 9.61(4.36-19.74) | 0.14(0.10-0.18) |
| Thailand | 1265.59(592.44-2686.15) | 4623.41(2152.81-9404.92) | 265.32 | 6.39(2.98-13.39) | 8.32(3.90-16.88) | 0.97(0.94-0.99) |
| Timor-Leste | 8.91(4.11-18.95) | 24.84(11.33-50.96) | 178.77 | 5.84(2.70-12.14) | 5.98(2.74-12.12) | 0.14(0.10-0.19) |
| Togo | 54.87(25.28-115.29) | 193.65(91.07-399.42) | 252.93 | 7.86(3.72-16.58) | 8.83(4.23-18.15) | 0.36(0.33-0.39) |
| Tokelau | 0.06(0.03-0.12) | 0.07(0.03-0.14) | 16.74 | 7.73(3.62-16.44) | 9.82(4.60-20.86) | 0.76(0.64-0.88) |
| Tonga | 2.51(1.20-5.26) | 4.20(2.01-8.57) | 67.74 | 8.59(4.05-18.20) | 9.93(4.73-20.30) | 0.28(0.03-0.53) |
| Trinidad and Tobago | 40.31(18.75-82.23) | 100.22(46.52-205.86) | 148.64 | 9.06(4.19-18.28) | 10.38(4.83-21.14) | 0.58(0.52-0.64) |
| Tunisia | 163.19(75.32-344.88) | 525.33(241.09-1078.50) | 221.91 | 6.48(3.02-13.76) | 7.96(3.69-16.32) | 0.70(0.66-0.73) |
| Turkey | 1330.67(621.88-2772.79) | 4113.89(1914.43-8509.58) | 209.16 | 6.93(3.20-14.44) | 8.73(4.07-18.06) | 0.72(0.68-0.77) |
| Turkmenistan | 115.57(54.85-238.19) | 272.06(126.90-570.02) | 135.40 | 10.42(4.99-21.45) | 12.31(5.80-25.58) | 0.54(0.52-0.57) |
| Tuvalu | 0.29(0.14-0.61) | 0.49(0.23-1.00) | 67.37 | 7.47(3.52-15.39) | 8.99(4.27-18.27) | 0.51(0.37-0.65) |
| Uganda | 285.49(132.98-600.81) | 745.17(343.22-1557.50) | 161.02 | 8.37(3.94-17.56) | 9.07(4.27-18.92) | 0.34(0.31-0.36) |
| Ukraine | 4641.59(2209.75-9615.13) | 5254.87(2496.94-10890.90) | 13.21 | 10.42(4.97-21.55) | 11.50(5.48-23.88) | 0.41(0.37-0.45) |
| United Arab Emirates | 13.21(6.10-27.17) | 139.60(63.52-300.77) | 956.49 | 7.18(3.31-14.64) | 9.43(4.40-19.42) | 1.02(0.98-1.06) |
| United Kingdom | 10816.94(5203.22-22387.21) | 16164.30(7773.15-33313.19) | 49.44 | 22.08(10.55-45.29) | 26.28(12.50-53.96) | 0.64(0.58-0.71) |
| United Republic of Tanzania | 516.88(239.73-1086.11) | 1343.45(615.85-2818.51) | 159.91 | 8.97(4.17-18.64) | 10.04(4.67-20.80) | 0.42(0.39-0.45) |
| United States of America | 46210.17(22014.16-96252.48) | 95165.26(46845.38-193404.40) | 105.94 | 26.32(12.56-54.57) | 32.95(16.44-67.20) | 0.67(0.57-0.76) |
| United States Virgin Islands | 5.18(2.44-10.75) | 12.19(5.80-25.62) | 135.44 | 10.94(5.14-22.75) | 12.23(5.78-25.71) | 0.39(0.34-0.44) |
| Uruguay | 351.23(167.36-729.17) | 609.54(289.34-1250.23) | 73.54 | 16.33(7.74-34.07) | 20.86(9.96-42.80) | 0.92(0.82-1.02) |
| Uzbekistan | 647.20(308.55-1344.88) | 1426.85(672.89-2960.20) | 120.46 | 9.94(4.75-20.67) | 11.74(5.49-24.20) | 0.55(0.50-0.59) |
| Vanuatu | 2.27(1.05-4.71) | 7.03(3.23-14.62) | 209.16 | 7.35(3.46-15.17) | 8.28(3.84-17.03) | 0.41(0.37-0.44) |
| Venezuela (Bolivarian Republic of) | 399.56(184.70-832.32) | 1354.42(637.97-2795.28) | 238.97 | 7.54(3.50-15.76) | 8.61(4.09-17.80) | 0.45(0.42-0.48) |
| Viet Nam | 1350.95(614.42-2783.49) | 3407.61(1581.01-7046.20) | 152.24 | 5.80(2.65-11.98) | 6.35(2.93-13.06) | 0.38(0.33-0.42) |
| Yemen | 130.99(60.27-269.56) | 429.45(203.21-894.60) | 227.85 | 4.95(2.30-10.16) | 5.91(2.81-12.28) | 0.71(0.66-0.76) |
| Zambia | 129.24(58.46-271.36) | 354.44(165.99-733.66) | 174.25 | 9.05(4.23-18.79) | 9.71(4.61-20.23) | 0.21(0.18-0.24) |
| Zimbabwe | 196.80(90.71-415.09) | 381.66(179.12-803.08) | 93.93 | 9.07(4.28-19.20) | 9.12(4.28-19.47) | -0.13(-0.23--0.04) |

S Table8 The DALY and age-standardized DALY rate of national hip osteoarthritis in male in 1990 and 2019, and its temporal trends from 1990 to 2019

| **Nation** | **DALY No. (95% UI)** | | **Change in absolute number (%)** | **Age-standardized DALY rate No.(95% UI)** | | **1990-2019 EAPC No. (95%CI)** |
| --- | --- | --- | --- | --- | --- | --- |
|  | **1990** | **2019** |  | **1990** | **2019** |  |
| Afghanistan | 217.66(99.77-457.78) | 469.56(217.34-989.22) | 115.73 | 5.92(2.74-12.25) | 6.91(3.20-14.45) | 0.68(0.56-0.80) |
| Albania | 123.78(58.82-252.49) | 272.18(127.21-564.01) | 119.89 | 11.78(5.59-24.06) | 13.52(6.35-27.69) | 0.53(0.50-0.55) |
| Algeria | 462.53(217.12-954.38) | 1680.09(776.53-3468.01) | 263.24 | 7.39(3.42-15.30) | 9.36(4.36-19.37) | 0.80(0.77-0.84) |
| American Samoa | 1.50(0.70-3.10) | 3.22(1.51-6.67) | 114.91 | 12.18(5.68-25.02) | 13.55(6.43-28.10) | 0.17(-0.07-0.42) |
| Andorra | 5.71(2.68-11.60) | 16.07(7.61-32.90) | 181.43 | 19.27(9.05-39.21) | 22.79(10.85-46.58) | 0.55(0.46-0.64) |
| Angola | 187.86(86.95-402.62) | 605.42(281.05-1257.00) | 222.28 | 9.13(4.27-19.22) | 11.07(5.24-22.80) | 0.70(0.67-0.73) |
| Antigua and Barbuda | 2.15(1.00-4.48) | 5.58(2.60-11.65) | 159.46 | 9.52(4.41-19.82) | 11.29(5.22-23.56) | 0.57(0.54-0.60) |
| Argentina | 2279.17(1086.01-4667.68) | 5084.43(2462.67-10638.51) | 123.08 | 15.66(7.49-32.07) | 21.51(10.42-45.10) | 1.02(0.90-1.14) |
| Armenia | 141.70(66.47-293.86) | 254.10(117.78-531.55) | 79.32 | 11.76(5.53-24.14) | 14.21(6.59-29.55) | 0.66(0.64-0.68) |
| Australia | 1583.26(743.29-3329.70) | 4410.32(2103.00-9094.68) | 178.56 | 17.78(8.33-37.36) | 24.04(11.35-49.27) | 0.99(0.90-1.08) |
| Austria | 839.46(398.03-1712.93) | 1609.14(750.38-3334.96) | 91.69 | 18.65(8.84-38.08) | 21.70(10.08-45.12) | 0.48(0.45-0.51) |
| Azerbaijan | 260.80(121.20-545.17) | 668.83(310.24-1400.21) | 156.45 | 12.28(5.88-25.72) | 14.78(6.84-30.84) | 0.65(0.59-0.71) |
| Bahamas | 7.60(3.53-15.71) | 22.44(10.31-45.95) | 195.37 | 10.74(5.07-22.33) | 12.18(5.59-24.99) | 0.41(0.35-0.48) |
| Bahrain | 11.85(5.30-25.09) | 93.26(42.42-192.79) | 686.90 | 9.06(4.15-18.90) | 10.73(4.96-22.32) | 0.51(0.48-0.55) |
| Bangladesh | 1380.29(620.45-2877.15) | 4318.54(2031.29-8890.37) | 212.87 | 5.02(2.29-10.32) | 6.23(2.95-12.81) | 0.87(0.82-0.92) |
| Barbados | 12.47(5.87-25.59) | 26.86(12.62-55.68) | 115.34 | 10.62(4.99-21.84) | 12.28(5.76-25.68) | 0.51(0.47-0.55) |
| Belarus | 656.08(305.51-1361.82) | 930.08(446.20-1939.08) | 41.76 | 13.44(6.25-27.93) | 15.30(7.35-31.44) | 0.39(0.35-0.43) |
| Belgium | 1177.16(557.26-2417.40) | 2023.46(955.80-4152.31) | 71.89 | 18.46(8.78-37.89) | 21.41(10.04-43.75) | 0.45(0.41-0.49) |
| Belize | 4.78(2.22-9.94) | 18.43(8.59-37.88) | 285.76 | 10.11(4.69-20.97) | 12.73(5.94-25.87) | 0.77(0.58-0.96) |
| Benin | 85.03(38.96-176.69) | 267.59(123.75-571.44) | 214.71 | 8.69(4.08-18.19) | 10.95(5.07-22.80) | 0.77(0.67-0.88) |
| Bermuda | 3.49(1.67-7.17) | 7.47(3.47-15.84) | 114.02 | 12.39(5.92-25.53) | 13.40(6.22-28.50) | 0.22(0.15-0.29) |
| Bhutan | 7.61(3.48-15.64) | 23.15(10.96-48.01) | 204.22 | 5.61(2.59-11.44) | 7.48(3.50-15.36) | 1.08(1.02-1.14) |
| Bolivia (Plurinational State of) | 123.07(57.50-252.93) | 446.02(208.32-925.64) | 262.40 | 7.88(3.69-16.44) | 10.23(4.79-21.39) | 0.93(0.89-0.96) |
| Bosnia and Herzegovina | 222.92(104.55-474.52) | 356.21(167.24-738.94) | 59.79 | 11.90(5.58-24.85) | 13.58(6.40-28.13) | 0.48(0.40-0.56) |
| Botswana | 33.21(15.53-69.21) | 111.11(52.57-227.42) | 234.57 | 12.54(5.95-26.23) | 16.99(8.13-35.05) | 0.97(0.89-1.05) |
| Brazil | 4153.53(1939.29-8422.14) | 13266.31(6230.16-27518.34) | 219.40 | 9.52(4.42-19.57) | 12.10(5.62-25.11) | 0.86(0.82-0.90) |
| Brunei Darussalam | 7.40(3.51-15.38) | 26.45(12.45-54.34) | 257.24 | 12.95(6.14-26.95) | 15.61(7.43-32.40) | 0.62(0.55-0.69) |
| Bulgaria | 811.22(378.79-1677.45) | 881.97(418.03-1882.05) | 8.72 | 14.09(6.55-29.08) | 14.85(7.00-31.32) | 0.08(0.04-0.12) |
| Burkina Faso | 174.30(81.12-363.83) | 432.84(198.39-904.86) | 148.33 | 8.23(3.86-17.00) | 9.83(4.60-20.64) | 0.60(0.56-0.64) |
| Burundi | 98.83(45.95-203.40) | 248.46(115.87-519.39) | 151.39 | 9.06(4.26-18.87) | 9.78(4.56-20.52) | 0.27(0.25-0.30) |
| Cabo Verde | 8.47(3.95-17.64) | 21.99(10.30-46.63) | 159.48 | 9.03(4.23-18.86) | 11.16(5.21-23.35) | 0.71(0.66-0.75) |
| Cambodia | 128.91(60.93-264.40) | 380.70(175.94-790.37) | 195.31 | 6.46(3.11-13.11) | 7.21(3.33-14.79) | 0.39(0.35-0.42) |
| Cameroon | 241.83(112.98-505.09) | 749.23(349.07-1557.57) | 209.82 | 10.60(5.01-21.89) | 12.03(5.64-24.81) | 0.36(0.33-0.39) |
| Canada | 2003.86(960.01-4119.99) | 5628.17(2681.61-11781.94) | 180.87 | 13.84(6.63-28.57) | 18.73(9.03-39.12) | 0.67(0.48-0.85) |
| Central African Republic | 50.37(23.48-105.21) | 104.81(47.81-218.24) | 108.06 | 9.02(4.27-18.75) | 9.95(4.64-20.86) | 0.36(0.33-0.39) |
| Chad | 107.39(50.65-217.01) | 272.95(126.18-572.74) | 154.15 | 7.79(3.70-15.78) | 8.66(4.00-17.89) | 0.30(0.26-0.34) |
| Chile | 776.25(370.20-1607.69) | 2470.69(1188.12-5166.46) | 218.28 | 16.66(7.96-34.41) | 22.55(10.80-46.96) | 0.92(0.80-1.04) |
| China | 25794.90 (11836.74-52680.99) | 81129.24 (36960.30-168843.19) | 214.52 | 5.93(2.74-12.19) | 8.12(3.71-16.90) | 1.14(1.06-1.22) |
| Colombia | 724.10(338.18-1500.60) | 2328.05(1079.14-4820.90) | 221.51 | 8.10(3.79-16.83) | 9.63(4.47-19.86) | 0.57(0.55-0.60) |
| Comoros | 10.69(5.03-22.38) | 26.58(12.36-55.60) | 148.56 | 9.80(4.60-20.38) | 11.47(5.34-23.69) | 0.56(0.53-0.59) |
| Congo | 50.83(23.56-103.61) | 172.90(80.19-359.09) | 240.17 | 10.42(4.81-21.47) | 12.55(5.89-25.96) | 0.67(0.65-0.68) |
| Cook Islands | 0.77(0.35-1.61) | 1.66(0.79-3.38) | 115.69 | 11.26(5.15-23.49) | 13.73(6.60-28.16) | 0.52(0.36-0.68) |
| Costa Rica | 76.09(35.35-154.67) | 245.46(115.90-513.87) | 222.61 | 8.67(4.02-17.80) | 10.20(4.83-21.37) | 0.55(0.52-0.58) |
| C么te d'Ivoire | 214.68(100.11-453.45) | 642.22(295.26-1368.11) | 199.16 | 9.09(4.21-19.46) | 10.45(4.90-22.20) | 0.39(0.33-0.45) |
| Croatia | 357.68(167.08-741.17) | 526.28(247.49-1106.65) | 47.14 | 13.26(6.21-27.28) | 14.72(6.87-30.73) | 0.40(0.36-0.45) |
| Cuba | 475.97(221.62-964.59) | 995.54(478.77-2068.77) | 109.16 | 9.45(4.40-19.12) | 11.38(5.44-23.63) | 0.70(0.66-0.74) |
| Cyprus | 66.47(31.54-137.45) | 187.91(90.23-383.31) | 182.70 | 17.12(8.11-35.42) | 20.72(9.94-42.26) | 0.68(0.62-0.75) |
| Czechia | 770.57(364.15-1598.71) | 1321.42(638.08-2778.48) | 71.49 | 13.54(6.44-28.04) | 14.95(7.20-31.09) | 0.30(0.26-0.33) |
| Democratic People's Republic of Korea | 407.01(189.78-843.35) | 850.84(400.87-1775.36) | 109.05 | 6.11(2.80-12.86) | 6.17(2.90-12.55) | 0.01(-0.02-0.04) |
| Democratic Republic of the Congo | 743.37(343.05-1566.91) | 1797.86(829.25-3740.33) | 141.85 | 9.71(4.54-20.42) | 10.29(4.86-21.22) | 0.10(0.05-0.15) |
| Denmark | 874.53(407.35-1781.05) | 1156.41(545.93-2402.56) | 32.23 | 26.02(12.12-53.15) | 23.38(11.11-48.81) | -0.53(-0.79--0.26) |
| Djibouti | 7.47(3.34-15.55) | 43.35(20.27-91.96) | 479.96 | 9.29(4.27-19.39) | 12.10(5.65-25.38) | 1.01(0.96-1.06) |
| Dominica | 2.75(1.30-5.62) | 4.97(2.30-10.28) | 80.86 | 9.57(4.56-19.43) | 11.24(5.24-23.17) | 0.53(0.46-0.60) |
| Dominican Republic | 172.35(79.94-359.66) | 533.78(249.94-1105.31) | 209.71 | 9.12(4.28-18.80) | 11.68(5.49-23.97) | 0.94(0.89-0.98) |
| Ecuador | 270.55(125.70-560.73) | 907.81(428.40-1887.91) | 235.54 | 10.02(4.67-20.79) | 12.30(5.82-25.68) | 0.61(0.53-0.69) |
| Egypt | 1259.32(579.27-2611.68) | 3598.08(1722.13-7408.40) | 185.72 | 8.18(3.83-16.63) | 9.87(4.65-20.35) | 0.40(0.29-0.52) |
| El Salvador | 113.50(53.70-236.32) | 243.92(117.28-502.04) | 114.91 | 8.11(3.86-16.85) | 9.82(4.71-20.18) | 0.64(0.54-0.74) |
| Equatorial Guinea | 8.16(3.75-16.98) | 30.49(14.43-63.52) | 273.58 | 9.12(4.25-18.75) | 13.67(6.51-29.00) | 1.67(1.55-1.79) |
| Eritrea | 38.83(17.79-80.87) | 127.73(60.21-272.54) | 228.98 | 8.56(3.97-18.32) | 9.99(4.76-20.62) | 0.53(0.48-0.57) |
| Estonia | 105.62(49.72-218.44) | 153.00(72.24-318.12) | 44.86 | 13.94(6.61-28.66) | 16.09(7.60-33.29) | 0.54(0.51-0.56) |
| Eswatini | 20.55(9.60-43.07) | 45.39(21.33-94.05) | 120.81 | 15.10(7.07-31.56) | 18.19(8.65-37.29) | 0.43(0.25-0.61) |
| Ethiopia | 1028.14(480.40-2129.93) | 2540.74(1196.79-5250.95) | 147.12 | 9.53(4.54-19.76) | 11.36(5.40-23.64) | 0.65(0.58-0.72) |
| Fiji | 18.72(8.61-38.56) | 43.73(20.51-90.38) | 133.57 | 9.81(4.57-20.41) | 11.65(5.60-23.70) | 0.45(0.33-0.57) |
| Finland | 537.89(250.45-1109.03) | 1094.66(510.16-2205.41) | 103.51 | 18.89(8.83-39.12) | 22.10(10.23-44.05) | 0.53(0.48-0.57) |
| France | 6489.51(3065.17-13500.92) | 12157.47(5816.73-25007.29) | 87.34 | 19.13(9.04-39.40) | 22.39(10.71-46.35) | 0.59(0.35-0.83) |
| Gabon | 27.75(13.26-59.10) | 76.64(35.19-160.13) | 176.12 | 10.85(5.20-22.79) | 14.64(6.91-30.74) | 1.00(0.92-1.08) |
| Gambia | 17.61(8.09-36.86) | 51.12(24.07-106.70) | 190.36 | 8.85(4.05-18.41) | 10.41(4.98-21.35) | 0.51(0.46-0.57) |
| Georgia | 349.38(163.02-709.57) | 345.92(159.97-723.55) | -0.99 | 13.97(6.53-28.53) | 14.45(6.70-30.29) | 0.08(0.06-0.10) |
| Germany | 9449.71(4493.38-19645.12) | 17375.32(8264.60-35323.97) | 83.87 | 19.21(9.19-39.69) | 22.28(10.62-45.47) | 0.47(0.42-0.52) |
| Ghana | 280.23(128.10-583.10) | 845.29(388.44-1759.41) | 201.65 | 8.78(4.12-18.40) | 11.06(5.17-22.70) | 0.73(0.65-0.81) |
| Greece | 1186.95(563.32-2442.48) | 1875.99(901.55-3869.36) | 58.05 | 17.29(8.22-35.49) | 19.67(9.43-40.78) | 1.51(1.06-1.97) |
| Greenland | 2.80(1.33-5.85) | 7.22(3.41-15.16) | 157.70 | 14.36(6.87-29.29) | 18.64(8.81-38.72) | 0.91(0.82-1.01) |
| Grenada | 2.59(1.21-5.45) | 5.93(2.80-12.49) | 129.31 | 8.77(4.06-18.35) | 10.68(5.01-22.57) | 0.64(0.57-0.70) |
| Guam | 4.85(2.25-10.09) | 12.44(5.76-25.30) | 156.45 | 11.38(5.28-23.77) | 13.24(6.17-26.88) | 0.52(0.46-0.59) |
| Guatemala | 135.89(63.23-279.85) | 442.85(207.86-906.42) | 225.88 | 7.16(3.35-14.69) | 8.64(4.08-17.74) | 0.61(0.57-0.66) |
| Guinea | 142.98(66.77-298.08) | 270.15(127.32-569.84) | 88.94 | 8.51(3.99-17.71) | 9.46(4.43-19.66) | 0.33(0.30-0.36) |
| Guinea-Bissau | 16.97(7.81-34.80) | 33.03(15.43-68.83) | 94.63 | 8.44(3.94-17.29) | 9.33(4.32-19.24) | 0.28(0.22-0.34) |
| Guyana | 17.13(7.97-35.81) | 32.74(15.22-69.63) | 91.15 | 9.01(4.20-18.82) | 10.73(4.96-22.83) | 0.58(0.51-0.65) |
| Haiti | 121.00(56.42-256.15) | 287.37(136.36-588.31) | 137.49 | 7.45(3.48-15.68) | 8.33(3.97-16.98) | 0.45(0.42-0.47) |
| Honduras | 78.48(36.60-165.75) | 265.42(123.24-542.75) | 238.21 | 7.50(3.54-15.79) | 9.00(4.21-18.66) | 0.65(0.58-0.71) |
| Hungary | 878.27(412.70-1831.07) | 1174.55(560.45-2438.73) | 33.73 | 14.38(6.73-29.95) | 15.53(7.39-32.08) | 0.22(0.18-0.26) |
| Iceland | 33.44(16.38-67.35) | 63.71(30.29-132.51) | 90.49 | 25.92(12.66-52.12) | 25.32(11.99-53.05) | -0.34(-0.50--0.18) |
| India | 14005.98(6672.16-28768.34) | 40803.11(19202.96-82451.07) | 191.33 | 5.45(2.58-11.24) | 6.96(3.23-14.04) | 0.57(0.42-0.72) |
| Indonesia | 3690.68(1679.42-7634.48) | 10197.49(4712.38-20907.55) | 176.30 | 7.38(3.40-15.09) | 9.16(4.28-18.75) | 0.81(0.77-0.85) |
| Iran (Islamic Republic of) | 1280.44(586.63-2632.68) | 3832.31(1796.61-7913.89) | 199.30 | 8.79(4.09-18.05) | 9.90(4.57-20.45) | 0.20(0.08-0.33) |
| Iraq | 340.86(160.66-698.61) | 1127.00(531.75-2316.37) | 230.64 | 8.53(4.02-17.56) | 9.18(4.29-18.83) | 0.25(0.22-0.28) |
| Ireland | 348.34(164.75-728.24) | 763.40(361.12-1555.94) | 119.15 | 19.09(9.01-39.78) | 22.20(10.53-45.42) | 0.50(0.46-0.54) |
| Israel | 396.81(189.33-809.82) | 1076.25(501.96-2200.34) | 171.23 | 18.20(8.71-37.32) | 20.98(9.75-43.14) | 0.43(0.35-0.50) |
| Italy | 6857.96(3246.68-14070.88) | 11582.91(5481.35-24155.67) | 68.90 | 18.37(8.65-37.51) | 20.49(9.67-42.37) | 0.48(0.34-0.61) |
| Jamaica | 76.03(35.57-155.78) | 162.44(75.84-335.91) | 113.65 | 9.36(4.38-19.40) | 11.30(5.32-23.32) | 0.73(0.67-0.79) |
| Japan | 10852.12(5085.95-22460.62) | 20062.40(9478.59-41527.67) | 84.87 | 14.23(6.65-29.49) | 15.10(7.12-31.24) | 0.12(0.09-0.15) |
| Jordan | 61.33(28.21-126.11) | 401.71(182.43-842.97) | 554.99 | 8.20(3.79-16.77) | 10.33(4.77-21.47) | 0.78(0.72-0.83) |
| Kazakhstan | 704.97(328.19-1466.64) | 1193.55(553.23-2470.87) | 69.31 | 14.09(6.59-28.85) | 16.02(7.66-33.29) | 0.35(0.31-0.39) |
| Kenya | 455.58(213.38-941.77) | 1565.33(733.01-3241.14) | 243.59 | 10.76(4.98-22.26) | 13.72(6.42-28.45) | 0.88(0.77-0.99) |
| Kiribati | 1.57(0.73-3.24) | 3.23(1.50-6.76) | 105.40 | 9.16(4.30-18.68) | 10.23(4.92-21.14) | 0.23(0.02-0.44) |
| Kuwait | 47.14(21.71-97.53) | 220.32(104.35-467.13) | 367.40 | 9.67(4.49-20.15) | 11.56(5.38-24.24) | 0.69(0.64-0.73) |
| Kyrgyzstan | 146.64(69.64-301.24) | 275.99(126.80-582.67) | 88.21 | 11.91(5.73-24.95) | 13.16(6.16-27.38) | 0.22(0.18-0.27) |
| Lao People's Democratic Republic | 67.00(31.95-135.87) | 174.27(80.18-363.81) | 160.09 | 6.61(3.16-13.46) | 7.78(3.65-16.14) | 0.62(0.59-0.65) |
| Latvia | 186.02(88.45-392.14) | 222.33(104.48-463.20) | 19.52 | 14.09(6.68-29.81) | 15.90(7.57-32.94) | 0.41(0.39-0.43) |
| Lebanon | 88.36(40.41-181.45) | 222.07(104.82-463.79) | 151.32 | 7.64(3.55-15.67) | 9.41(4.43-19.73) | 0.42(0.04-0.81) |
| Lesotho | 53.57(25.13-113.05) | 83.21(38.93-172.45) | 55.33 | 12.10(5.75-25.25) | 15.12(7.18-31.05) | 0.76(0.75-0.78) |
| Liberia | 54.99(25.71-114.53) | 126.44(58.99-265.04) | 129.93 | 9.08(4.27-18.92) | 10.97(5.13-23.02) | 0.80(0.71-0.89) |
| Libya | 92.14(42.66-189.79) | 277.57(129.98-575.70) | 201.27 | 8.71(4.04-18.06) | 9.76(4.54-20.35) | 0.39(0.35-0.42) |
| Lithuania | 242.66(115.43-499.25) | 315.03(148.38-661.45) | 29.83 | 13.73(6.47-28.15) | 15.36(7.25-32.07) | 0.39(0.34-0.45) |
| Luxembourg | 44.02(20.60-91.18) | 101.27(48.70-208.58) | 130.08 | 19.26(8.99-39.93) | 22.13(10.65-45.57) | 0.45(0.42-0.48) |
| Madagascar | 241.70(112.79-507.69) | 585.93(270.10-1240.01) | 142.42 | 9.06(4.26-18.80) | 10.24(4.81-21.57) | 0.46(0.41-0.51) |
| Malawi | 179.55(83.38-373.40) | 394.63(181.70-803.33) | 119.79 | 9.52(4.44-19.66) | 11.42(5.26-23.41) | 0.72(0.69-0.76) |
| Malaysia | 371.80(172.56-762.34) | 1379.66(631.21-2868.12) | 271.07 | 7.83(3.67-16.11) | 9.90(4.62-20.43) | 0.86(0.84-0.88) |
| Maldives | 3.68(1.72-7.80) | 17.66(8.10-36.30) | 380.19 | 6.81(3.21-14.05) | 9.02(4.14-18.74) | 1.13(1.02-1.25) |
| Mali | 169.91(78.16-355.63) | 428.85(200.95-877.84) | 152.40 | 8.03(3.76-16.75) | 9.29(4.37-19.03) | 0.51(0.47-0.55) |
| Malta | 35.75(16.79-73.57) | 88.85(41.68-180.94) | 148.58 | 18.60(8.72-38.19) | 22.45(10.49-45.95) | 0.59(0.49-0.70) |
| Marshall Islands | 0.70(0.33-1.41) | 1.81(0.83-3.78) | 158.71 | 8.24(3.83-17.00) | 9.66(4.51-20.00) | 0.42(0.30-0.53) |
| Mauritania | 49.55(23.58-102.43) | 128.65(60.35-267.43) | 159.66 | 10.10(4.79-20.87) | 11.95(5.64-24.80) | 0.49(0.41-0.58) |
| Mauritius | 27.74(12.77-57.31) | 79.10(36.81-161.46) | 185.16 | 7.90(3.67-16.22) | 9.61(4.47-19.82) | 0.70(0.67-0.73) |
| Mexico | 2199.44(1027.95-4546.62) | 6597.42(3083.11-13613.29) | 199.96 | 10.22(4.76-21.43) | 11.81(5.57-24.49) | -0.01(-0.18-0.16) |
| Micronesia (Federated States of) | 2.34(1.10-4.82) | 3.86(1.78-8.09) | 64.96 | 9.77(4.58-20.34) | 10.88(5.15-22.76) | 0.23(0.04-0.42) |
| Monaco | 5.80(2.75-12.26) | 9.36(4.41-19.50) | 61.49 | 21.36(10.14-44.18) | 23.94(11.25-49.85) | 0.35(0.30-0.39) |
| Mongolia | 56.77(26.48-117.68) | 144.17(67.54-302.92) | 153.95 | 11.63(5.40-24.04) | 13.01(6.07-27.23) | 0.39(0.35-0.42) |
| Montenegro | 39.34(18.51-81.14) | 64.46(30.27-132.94) | 63.85 | 13.98(6.56-28.76) | 14.60(6.84-30.06) | 0.16(0.13-0.19) |
| Morocco | 500.38(228.51-1051.56) | 1406.09(648.44-2920.25) | 181.00 | 7.14(3.29-14.81) | 8.68(4.06-17.80) | 0.63(0.60-0.65) |
| Mozambique | 269.93(124.23-568.86) | 552.60(259.61-1134.38) | 104.72 | 9.10(4.17-18.88) | 10.69(5.04-22.12) | 0.63(0.59-0.66) |
| Myanmar | 731.23(337.38-1524.17) | 1623.97(750.38-3396.43) | 122.09 | 6.55(3.07-13.75) | 7.71(3.59-16.04) | 0.65(0.59-0.70) |
| Namibia | 41.46(19.64-86.79) | 92.37(44.00-192.88) | 122.77 | 12.26(5.88-25.82) | 14.68(7.02-31.00) | 0.56(0.52-0.59) |
| Nauru | 0.22(0.10-0.45) | 0.24(0.11-0.50) | 10.86 | 10.14(4.78-21.16) | 11.30(5.40-22.80) | 0.18(0.10-0.27) |
| Nepal | 262.27(121.00-542.73) | 741.29(348.35-1511.15) | 182.65 | 5.07(2.37-10.40) | 6.80(3.23-13.89) | 1.12(1.05-1.18) |
| Netherlands | 1611.82(777.69-3300.47) | 3287.34(1555.11-6808.23) | 103.95 | 19.00(9.16-38.81) | 22.14(10.51-45.30) | 0.55(0.51-0.59) |
| New Zealand | 315.84(148.04-643.74) | 788.86(373.19-1624.79) | 149.77 | 17.99(8.45-36.56) | 23.06(10.90-47.76) | 0.77(0.69-0.84) |
| Nicaragua | 58.78(27.19-120.48) | 196.60(90.91-416.09) | 234.49 | 7.79(3.65-16.09) | 9.34(4.32-19.54) | 0.59(0.50-0.67) |
| Niger | 126.25(57.59-260.65) | 351.63(163.25-741.15) | 178.51 | 8.14(3.82-16.78) | 8.80(4.15-18.49) | 0.26(0.21-0.30) |
| Nigeria | 2363.00(1099.86-4905.30) | 4289.81(1985.64-8921.10) | 81.54 | 9.72(4.53-20.02) | 9.73(4.48-20.33) | -0.31(-0.56--0.06) |
| Niue | 0.10(0.05-0.20) | 0.13(0.06-0.27) | 30.29 | 10.51(4.96-21.88) | 12.70(5.95-26.46) | 0.58(0.41-0.74) |
| North Macedonia | 117.85(54.32-245.09) | 220.66(103.01-468.71) | 87.23 | 12.93(5.93-27.09) | 14.23(6.72-30.36) | 0.29(0.27-0.31) |
| Northern Mariana Islands | 1.43(0.67-2.93) | 3.55(1.65-7.36) | 148.76 | 11.35(5.35-23.51) | 12.26(5.81-24.95) | 0.12(-0.05-0.30) |
| Norway | 522.85(246.86-1074.73) | 922.96(436.95-1926.72) | 76.52 | 18.87(8.96-38.66) | 21.79(10.37-45.36) | 0.48(0.45-0.51) |
| Oman | 31.73(14.83-65.50) | 148.30(68.30-313.10) | 367.42 | 6.91(3.18-14.33) | 9.91(4.59-21.02) | 1.30(1.28-1.32) |
| Pakistan | 1983.96(941.58-4103.74) | 4959.47(2360.42-10128.49) | 149.98 | 6.14(2.89-12.68) | 7.91(3.75-16.08) | 1.01(0.94-1.07) |
| Palau | 0.55(0.25-1.12) | 1.47(0.69-3.05) | 169.44 | 10.84(5.09-22.41) | 12.82(6.04-26.44) | 0.40(0.22-0.58) |
| Palestine | 30.96(13.95-64.27) | 107.97(50.12-223.92) | 248.72 | 7.69(3.44-15.92) | 8.46(3.93-17.61) | 0.20(0.14-0.26) |
| Panama | 56.18(25.64-117.80) | 192.42(90.45-402.64) | 242.51 | 7.26(3.31-15.10) | 9.48(4.45-19.89) | 0.81(0.77-0.86) |
| Papua New Guinea | 78.45(37.25-161.96) | 225.11(104.41-479.88) | 186.95 | 7.80(3.68-16.10) | 8.43(4.03-17.60) | 0.15(0.07-0.24) |
| Paraguay | 96.55(45.33-202.88) | 287.51(136.18-587.23) | 197.78 | 8.83(4.16-18.46) | 10.48(5.02-21.43) | 0.58(0.54-0.62) |
| Peru | 512.95(238.28-1048.87) | 1731.68(802.48-3559.69) | 237.59 | 8.54(3.96-17.47) | 11.13(5.14-22.79) | 0.96(0.93-0.99) |
| Philippines | 1148.67(536.05-2349.16) | 3445.28(1608.91-7102.56) | 199.94 | 7.53(3.51-15.47) | 8.98(4.18-18.64) | 0.60(0.58-0.62) |
| Poland | 2722.16(1281.47-5638.87) | 4839.37(2288.01-10037.26) | 77.78 | 14.78(6.97-30.69) | 16.72(7.91-34.55) | 0.38(0.35-0.41) |
| Portugal | 1032.02(484.37-2123.00) | 1932.51(925.64-4001.00) | 87.26 | 17.25(8.10-35.62) | 20.52(9.78-42.45) | 0.10(-0.07-0.28) |
| Puerto Rico | 207.55(97.82-425.27) | 433.88(205.59-900.83) | 109.05 | 12.41(5.82-25.60) | 14.94(7.10-30.87) | 0.71(0.65-0.78) |
| Qatar | 13.44(6.23-28.48) | 146.63(68.14-305.98) | 990.71 | 10.33(4.83-21.34) | 11.92(5.58-24.80) | 0.42(0.32-0.51) |
| Republic of Korea | 1848.53(840.91-3866.75) | 6217.76(2945.65-12983.83) | 236.36 | 13.31(6.15-27.60) | 14.81(7.04-30.72) | 0.27(0.22-0.31) |
| Republic of Moldova | 230.19(109.97-485.66) | 364.19(171.60-757.60) | 58.21 | 12.57(5.96-26.51) | 15.32(7.20-31.77) | 0.72(0.62-0.81) |
| Romania | 1631.47(775.91-3400.78) | 2269.40(1081.00-4730.67) | 39.10 | 12.89(6.16-26.82) | 14.80(7.02-30.75) | 0.46(0.44-0.48) |
| Russian Federation | 10091.26(4746.68-20706.91) | 15999.14(7596.25-33116.14) | 58.54 | 15.47(7.30-31.96) | 17.69(8.40-36.41) | 0.39(0.37-0.41) |
| Rwanda | 125.80(58.56-263.88) | 307.23(144.59-639.75) | 144.22 | 9.38(4.43-19.73) | 11.14(5.23-23.08) | 0.63(0.57-0.70) |
| Saint Kitts and Nevis | 1.62(0.77-3.42) | 4.17(1.96-8.77) | 157.85 | 10.09(4.81-21.07) | 12.30(5.82-25.56) | 0.66(0.60-0.71) |
| Saint Lucia | 3.65(1.69-7.52) | 11.91(5.55-24.55) | 226.35 | 9.31(4.31-19.03) | 11.47(5.30-23.31) | 0.66(0.59-0.74) |
| Saint Vincent and the Grenadines | 2.86(1.36-5.98) | 7.88(3.69-16.50) | 175.19 | 8.96(4.31-18.86) | 11.23(5.28-23.54) | 0.85(0.79-0.91) |
| Samoa | 4.75(2.22-9.98) | 8.60(4.02-17.75) | 81.04 | 10.87(5.06-22.56) | 11.59(5.51-23.83) | 0.06(-0.09-0.20) |
| San Marino | 2.99(1.42-6.17) | 6.24(2.93-12.89) | 108.79 | 20.11(9.60-41.26) | 22.84(10.71-46.62) | 0.43(0.37-0.49) |
| Sao Tome and Principe | 2.90(1.34-5.97) | 6.15(2.86-12.64) | 112.33 | 9.33(4.35-19.20) | 11.16(5.22-23.53) | 0.56(0.52-0.60) |
| Saudi Arabia | 318.68(149.32-656.12) | 1489.07(679.84-3096.43) | 367.27 | 8.03(3.78-16.59) | 10.77(5.08-22.40) | 0.96(0.91-1.02) |
| Senegal | 156.30(73.55-327.97) | 386.89(181.40-812.98) | 147.54 | 9.31(4.36-19.36) | 10.27(4.85-21.39) | 0.24(0.19-0.30) |
| Serbia | 720.52(338.27-1521.74) | 1041.17(492.15-2152.45) | 44.50 | 13.40(6.30-28.10) | 14.91(7.11-30.78) | 0.41(0.39-0.42) |
| Seychelles | 2.16(1.02-4.47) | 5.59(2.59-11.58) | 158.25 | 8.64(4.12-17.90) | 9.97(4.74-20.23) | 0.44(0.41-0.48) |
| Sierra Leone | 84.33(38.57-175.11) | 183.75(87.01-383.59) | 117.90 | 8.44(3.90-17.29) | 9.63(4.64-20.03) | 0.40(0.37-0.42) |
| Singapore | 145.29(68.66-304.27) | 646.42(297.05-1331.76) | 344.92 | 13.18(6.28-27.55) | 16.01(7.42-33.08) | 0.58(0.52-0.63) |
| Slovakia | 353.91(168.36-729.53) | 593.85(282.58-1236.87) | 67.80 | 13.83(6.60-28.44) | 15.10(7.23-31.30) | 0.21(0.16-0.25) |
| Slovenia | 133.99(63.91-275.94) | 276.28(133.83-574.20) | 106.18 | 13.39(6.40-27.57) | 15.26(7.43-31.71) | 0.45(0.41-0.49) |
| Solomon Islands | 6.56(3.11-13.39) | 15.33(7.20-31.72) | 133.71 | 8.45(3.95-17.35) | 9.35(4.49-19.49) | 0.24(0.07-0.42) |
| Somalia | 118.17(53.54-249.52) | 317.03(143.74-652.32) | 168.29 | 9.19(4.23-19.39) | 10.12(4.66-20.88) | 0.39(0.36-0.42) |
| South Africa | 1652.74(780.88-3387.61) | 4243.17(2005.58-8760.60) | 156.74 | 17.90(8.50-36.92) | 21.29(9.99-43.66) | 0.65(0.58-0.73) |
| South Sudan | 128.49(59.09-268.66) | 229.04(107.31-472.75) | 78.25 | 9.34(4.36-19.38) | 10.71(5.08-22.01) | 0.51(0.49-0.54) |
| Spain | 4692.72(2231.43-9581.65) | 9152.50(4346.65-18959.85) | 95.04 | 20.14(9.53-41.01) | 23.37(11.11-48.36) | 0.40(0.29-0.51) |
| Sri Lanka | 402.70(189.03-843.38) | 952.99(446.63-1994.15) | 136.65 | 7.10(3.36-14.64) | 8.15(3.83-16.93) | 0.51(0.49-0.53) |
| Sudan | 309.22(143.91-643.78) | 884.22(416.11-1848.55) | 185.95 | 6.17(2.85-12.74) | 8.32(3.89-17.10) | 1.04(0.95-1.12) |
| Suriname | 12.30(5.70-25.64) | 33.73(15.99-70.71) | 174.29 | 9.67(4.46-20.21) | 11.84(5.53-24.56) | 0.72(0.69-0.75) |
| Sweden | 1118.94(540.48-2302.94) | 2115.57(995.06-4372.14) | 89.07 | 18.14(8.73-36.98) | 24.04(11.49-49.35) | 0.86(0.58-1.14) |
| Switzerland | 786.24(370.67-1638.58) | 1513.06(713.14-3148.32) | 92.44 | 18.36(8.67-38.44) | 20.44(9.70-42.16) | 0.36(0.34-0.38) |
| Syrian Arab Republic | 223.69(103.83-461.04) | 585.29(271.70-1228.77) | 161.66 | 7.82(3.69-15.86) | 8.83(4.12-18.43) | 0.29(0.19-0.38) |
| Taiwan (Province of China) | 629.64(288.26-1319.76) | 1553.24(712.70-3196.95) | 146.69 | 7.18(3.30-15.01) | 8.41(3.86-17.18) | 0.69(0.61-0.78) |
| Tajikistan | 136.44(64.14-284.33) | 298.69(141.03-625.94) | 118.92 | 10.76(5.06-22.67) | 11.42(5.47-24.02) | 0.13(0.08-0.17) |
| Thailand | 1231.70(574.83-2567.47) | 4555.98(2111.83-9433.82) | 269.89 | 7.03(3.31-14.70) | 9.55(4.42-19.78) | 1.10(1.06-1.13) |
| Timor-Leste | 10.28(4.67-21.24) | 28.10(13.10-56.57) | 173.50 | 6.47(2.97-13.33) | 6.82(3.22-13.63) | 0.23(0.18-0.29) |
| Togo | 53.00(24.46-109.31) | 169.59(79.04-354.29) | 219.97 | 8.53(4.01-17.54) | 9.76(4.54-20.16) | 0.37(0.31-0.42) |
| Tokelau | 0.06(0.03-0.12) | 0.08(0.04-0.16) | 37.10 | 9.39(4.40-19.54) | 11.56(5.37-24.13) | 0.62(0.50-0.75) |
| Tonga | 2.84(1.33-5.94) | 4.37(2.04-9.11) | 53.99 | 10.35(4.85-21.65) | 11.73(5.51-24.29) | 0.17(-0.08-0.43) |
| Trinidad and Tobago | 41.80(19.91-87.26) | 108.58(50.97-227.22) | 159.73 | 10.22(4.84-21.21) | 12.01(5.68-25.16) | 0.66(0.59-0.72) |
| Tunisia | 200.92(89.85-410.08) | 560.99(266.22-1151.91) | 179.22 | 7.52(3.35-15.46) | 8.89(4.24-18.39) | 0.54(0.51-0.56) |
| Turkey | 1461.75(672.58-2990.47) | 4248.09(1978.82-8889.54) | 190.62 | 8.11(3.74-16.63) | 9.85(4.62-20.44) | 0.57(0.54-0.61) |
| Turkmenistan | 102.61(47.17-210.49) | 268.30(125.72-566.73) | 161.48 | 12.42(5.79-25.41) | 14.69(6.94-30.66) | 0.53(0.49-0.56) |
| Tuvalu | 0.27(0.13-0.57) | 0.52(0.24-1.07) | 90.37 | 9.07(4.23-18.66) | 10.61(4.93-21.90) | 0.41(0.27-0.56) |
| Uganda | 299.53(139.47-621.83) | 725.73(331.32-1493.32) | 142.29 | 9.18(4.30-18.99) | 10.89(5.01-22.36) | 0.65(0.61-0.69) |
| Ukraine | 4179.10(1919.69-8633.37) | 4816.36(2305.98-9953.92) | 15.25 | 15.90(7.37-32.90) | 16.81(8.05-34.69) | 0.18(0.16-0.20) |
| United Arab Emirates | 41.07(18.73-89.38) | 556.51(250.04-1176.93) | 1255.07 | 8.37(3.87-17.69) | 10.71(5.01-21.99) | 0.86(0.82-0.89) |
| United Kingdom | 8131.93(3856.13-16733.39) | 13048.58(6225.92-27076.10) | 60.46 | 21.97(10.36-45.06) | 24.31(11.63-50.16) | 0.35(0.34-0.37) |
| United Republic of Tanzania | 540.08(249.53-1120.27) | 1499.47(683.14-3126.18) | 177.64 | 9.88(4.64-20.47) | 12.13(5.52-25.10) | 0.76(0.72-0.80) |
| United States of America | 32551.59(15583.52-66341.05) | 73501.76(36596.65-149712.33) | 125.80 | 24.40(11.69-49.80) | 30.13(14.98-60.96) | 0.74(0.63-0.86) |
| United States Virgin Islands | 5.10(2.39-10.53) | 11.47(5.30-23.90) | 124.92 | 12.32(5.75-25.38) | 14.17(6.52-29.26) | 0.47(0.40-0.54) |
| Uruguay | 270.47(128.75-549.98) | 473.17(223.77-986.52) | 74.95 | 16.03(7.64-32.64) | 21.80(10.33-45.29) | 0.98(0.86-1.10) |
| Uzbekistan | 577.54(266.82-1195.68) | 1441.70(673.63-3008.29) | 149.63 | 11.82(5.52-24.71) | 13.98(6.56-29.14) | 0.54(0.49-0.59) |
| Vanuatu | 3.31(1.55-6.96) | 9.07(4.30-18.68) | 174.01 | 8.85(4.18-18.53) | 9.78(4.69-20.41) | 0.32(0.28-0.36) |
| Venezuela (Bolivarian Republic of) | 414.25(189.90-837.96) | 1399.80(662.60-2889.89) | 237.91 | 8.58(4.00-17.28) | 10.00(4.75-20.55) | 0.50(0.47-0.53) |
| Viet Nam | 1104.80(511.13-2281.10) | 3119.65(1448.48-6426.98) | 182.37 | 6.39(3.02-13.19) | 7.27(3.41-14.77) | 0.50(0.45-0.54) |
| Yemen | 147.23(67.14-315.63) | 477.80(215.56-1000.58) | 224.53 | 5.77(2.69-12.07) | 6.67(2.98-13.80) | 0.55(0.53-0.57) |
| Zambia | 152.71(72.49-313.53) | 401.83(188.89-831.46) | 163.14 | 9.94(4.72-20.41) | 11.61(5.43-24.15) | 0.52(0.46-0.59) |
| Zimbabwe | 262.51(122.46-539.74) | 428.09(203.77-890.12) | 63.08 | 12.71(5.95-25.88) | 13.75(6.62-28.32) | 0.12(0.06-0.18) |

S Table9 The incidence and DALY of regional hip osteoarthritis in 1990 and 2019, and its change from 1990 to 2019

| location_id | incidence_90 | incidence_19 | nDALY_90 | nDALY_19 | time_incidence | time_DALY |
| --- | --- | --- | --- | --- | --- | --- |
| Global | 735780.03 | 1584873.56 | 456515.32 | 1036169.89 | 1.15 | 1.27 |
| East Asia | 85093.52 | 250634.46 | 50404.27 | 157944.56 | 1.95 | 2.13 |
| Southeast Asia | 32340.98 | 89308.21 | 17706.70 | 50319.41 | 1.76 | 1.84 |
| Oceania | 473.00 | 1262.85 | 243.74 | 641.61 | 1.67 | 1.63 |
| Central Asia | 9567.38 | 19175.31 | 5427.97 | 10045.08 | 1.00 | 0.85 |
| Central Europe | 29919.61 | 41168.04 | 18381.83 | 29339.77 | 0.38 | 0.60 |
| Eastern Europe | 56168.47 | 72875.02 | 33588.85 | 47825.41 | 0.30 | 0.42 |
| High-income Asia Pacific | 45644.22 | 72845.23 | 27125.18 | 58041.01 | 0.60 | 1.14 |
| Australasia | 6478.05 | 15665.70 | 4149.98 | 11014.46 | 1.42 | 1.65 |
| Western Europe | 166809.86 | 258321.84 | 113570.48 | 192504.35 | 0.55 | 0.70 |
| Southern Latin America | 12325.29 | 27087.23 | 7443.20 | 17471.43 | 1.20 | 1.35 |
| High-income North America | 122961.60 | 263330.10 | 83325.10 | 180917.53 | 1.14 | 1.17 |
| Caribbean | 3859.83 | 8667.73 | 2375.13 | 5525.50 | 1.25 | 1.33 |
| Andean Latin America | 3143.27 | 9812.60 | 1785.03 | 5925.41 | 2.12 | 2.32 |
| Central Latin America | 12917.93 | 38048.84 | 7284.92 | 23051.48 | 1.95 | 2.16 |
| Tropical Latin America | 15124.77 | 43951.58 | 8404.92 | 26668.77 | 1.91 | 2.17 |
| North Africa and Middle East | 23107.18 | 74455.86 | 12873.62 | 40844.30 | 2.22 | 2.17 |
| South Asia | 71009.16 | 200380.34 | 41587.34 | 127261.97 | 1.82 | 2.06 |
| Central Sub-Saharan Africa | 4269.29 | 11328.72 | 2170.89 | 5574.24 | 1.65 | 1.57 |
| Eastern Sub-Saharan Africa | 13457.17 | 34851.66 | 7067.55 | 17806.06 | 1.59 | 1.52 |
| Southern Sub-Saharan Africa | 6418.56 | 15362.54 | 3545.38 | 8330.06 | 1.39 | 1.35 |
| Western Sub-Saharan Africa | 14690.88 | 36339.71 | 8053.24 | 19117.48 | 1.47 | 1.37 |
| High-middle SDI | 196558.80 | 373308.55 | 120333.66 | 248140.77 | 0.90 | 1.06 |
| High SDI | 300888.06 | 555943.14 | 200247.43 | 398010.08 | 0.85 | 0.99 |
| Low-middle SDI | 74911.64 | 196261.41 | 43441.31 | 120845.69 | 1.62 | 1.78 |
| Low SDI | 35086.28 | 83533.21 | 19249.03 | 48368.60 | 1.38 | 1.51 |
| Middle SDI | 127969.79 | 351905.33 | 73017.13 | 220293.77 | 1.75 | 2.02 |

S Table10 The age-standardized incidence and DALY rate of regional hip osteoarthritis in 1990 and 2019, and its change from 1990 to 2019

| LocationName | ASIR_1990 | ASIR_2019 | ASR of DALY_2019 | ASR of DALY_2019 | ASIRC | ASR of DALYC |
| --- | --- | --- | --- | --- | --- | --- |
| Global | 17.02 | 18.70 | 11.54 | 12.57 | 0.10 | 0.09 |
| East Asia | 8.34 | 11.39 | 5.52 | 7.44 | 0.37 | 0.35 |
| Southeast Asia | 10.50 | 12.51 | 6.65 | 7.95 | 0.19 | 0.20 |
| Oceania | 12.40 | 13.60 | 7.82 | 8.63 | 0.10 | 0.10 |
| Central Asia | 18.58 | 20.90 | 11.53 | 13.09 | 0.13 | 0.14 |
| Central Europe | 20.22 | 23.28 | 12.47 | 14.43 | 0.15 | 0.16 |
| Eastern Europe | 20.03 | 23.10 | 12.05 | 14.15 | 0.15 | 0.17 |
| High-income Asia Pacific | 21.65 | 23.72 | 13.31 | 14.68 | 0.10 | 0.10 |
| Australasia | 29.20 | 38.74 | 17.92 | 23.72 | 0.33 | 0.32 |
| Western Europe | 33.44 | 38.36 | 20.42 | 23.41 | 0.15 | 0.15 |
| Southern Latin America | 26.19 | 34.70 | 16.10 | 21.32 | 0.32 | 0.32 |
| High-income North America | 40.04 | 50.23 | 24.51 | 30.34 | 0.25 | 0.24 |
| Caribbean | 14.22 | 16.66 | 9.07 | 10.66 | 0.17 | 0.18 |
| Andean Latin America | 13.38 | 16.52 | 8.48 | 10.45 | 0.23 | 0.23 |
| Central Latin America | 13.13 | 15.12 | 8.36 | 9.61 | 0.15 | 0.15 |
| Tropical Latin America | 14.14 | 17.14 | 8.87 | 10.84 | 0.21 | 0.22 |
| North Africa and Middle East | 11.14 | 13.79 | 7.18 | 8.85 | 0.24 | 0.23 |
| South Asia | 9.74 | 12.36 | 6.74 | 8.62 | 0.27 | 0.28 |
| Central Sub-Saharan Africa | 14.74 | 15.89 | 9.18 | 9.87 | 0.08 | 0.08 |
| Eastern Sub-Saharan Africa | 14.53 | 16.44 | 9.09 | 10.30 | 0.13 | 0.13 |
| Southern Sub-Saharan Africa | 20.17 | 23.08 | 12.53 | 14.19 | 0.14 | 0.13 |
| Western Sub-Saharan Africa | 13.96 | 14.63 | 8.84 | 9.49 | 0.06 | 0.07 |
| High-middle SDI | 17.35 | 18.53 | 11.23 | 12.20 | 0.07 | 0.09 |
| High SDI | 31.34 | 36.94 | 19.56 | 22.65 | 0.18 | 0.16 |
| Low-middle SDI | 10.23 | 12.61 | 6.89 | 8.52 | 0.23 | 0.24 |
| Low SDI | 11.95 | 12.80 | 7.75 | 8.77 | 0.07 | 0.13 |
| Middle SDI | 10.43 | 12.70 | 6.82 | 8.57 | 0.22 | 0.26 |

S Table11 Age distribution of incidence (per 100,000) for hip osteoarthritis in different countries in 2019.

| LocationName | 15 to 19 | 20 to 24 | 25 to 29 | 30 to 34 | 35 to 39 | 40 to 44 | 45 to 49 | 50 to 54 | 55 to 59 | 60 to 64 | 65 to 69 | 70 to 74 | 75 to 79 | 80 plus | All Ages |
| --- | --- | --- | --- | --- | --- | --- | --- | --- | --- | --- | --- | --- | --- | --- | --- |
| Cambodia | 0.00 | 0.00 | 0.00 | 5.27 | 8.48 | 14.67 | 23.90 | 33.09 | 42.18 | 44.68 | 40.98 | 31.19 | 15.16 | 5.35 | 8.95 |
| Canada | 0.00 | 0.00 | 0.00 | 11.56 | 22.40 | 44.08 | 76.54 | 101.12 | 117.89 | 127.94 | 131.50 | 101.19 | 37.79 | 3.90 | 47.92 |
| United States of America | 0.00 | 0.00 | 0.00 | 18.83 | 37.93 | 76.45 | 134.16 | 183.79 | 224.88 | 225.19 | 186.36 | 127.31 | 48.59 | 5.67 | 74.94 |
| Antigua and Barbuda | 0.00 | 0.00 | 0.00 | 0.01 | 0.03 | 0.05 | 0.09 | 0.13 | 0.15 | 0.16 | 0.12 | 0.08 | 0.03 | 0.01 | 0.04 |
| Bahamas | 0.00 | 0.00 | 0.00 | 32.62 | 57.09 | 105.36 | 178.85 | 238.89 | 309.42 | 311.12 | 230.09 | 148.57 | 72.76 | 21.98 | 90.63 |
| Barbados | 0.00 | 0.00 | 0.00 | 5.24 | 9.87 | 18.12 | 33.84 | 52.10 | 77.07 | 92.73 | 87.58 | 72.12 | 30.80 | 10.22 | 21.37 |
| Belize | 0.00 | 0.00 | 0.00 | 13.13 | 19.01 | 31.99 | 44.60 | 51.35 | 54.25 | 44.41 | 32.56 | 19.45 | 8.72 | 2.49 | 20.81 |
| Cuba | 0.00 | 0.00 | 0.00 | 189.59 | 304.17 | 691.78 | 1832.99 | 3260.91 | 4404.13 | 4081.97 | 4184.46 | 3671.92 | 1965.69 | 563.61 | 718.04 |
| Indonesia | 0.00 | 0.00 | 0.00 | 166.21 | 317.14 | 487.71 | 517.81 | 590.50 | 713.55 | 836.13 | 567.35 | 319.28 | 142.04 | 41.15 | 303.24 |
| Dominica | 0.00 | 0.00 | 0.00 | 0.00 | 0.00 | 0.00 | 0.01 | 0.02 | 0.02 | 0.03 | 0.02 | 0.02 | 0.01 | 0.01 | 0.01 |
| Dominican Republic | 0.00 | 0.00 | 0.00 | 1245.42 | 2172.33 | 4171.23 | 5682.49 | 6448.40 | 7633.45 | 7322.48 | 6235.57 | 4645.13 | 1767.04 | 475.27 | 2503.18 |
| Grenada | 0.00 | 0.00 | 0.00 | 0.06 | 0.10 | 0.21 | 0.44 | 0.72 | 0.92 | 0.87 | 0.70 | 0.53 | 0.26 | 0.05 | 0.18 |
| Guyana | 0.00 | 0.00 | 0.00 | 50.44 | 96.46 | 170.09 | 234.90 | 286.48 | 382.75 | 423.62 | 360.09 | 191.69 | 78.94 | 31.07 | 113.93 |
| Haiti | 0.00 | 0.00 | 0.00 | 110.46 | 172.22 | 251.17 | 356.96 | 439.36 | 518.87 | 549.48 | 504.25 | 396.16 | 182.67 | 53.83 | 140.02 |
| Jamaica | 0.00 | 0.00 | 0.00 | 1.61 | 2.78 | 5.71 | 12.36 | 19.85 | 27.06 | 28.41 | 23.47 | 20.56 | 10.09 | 4.95 | 4.02 |
| Saint Lucia | 0.00 | 0.00 | 0.00 | 0.43 | 0.88 | 1.82 | 3.19 | 4.60 | 5.53 | 5.34 | 4.25 | 3.24 | 1.51 | 0.34 | 1.36 |
| Saint Vincent and the Grenadines | 0.00 | 0.00 | 0.00 | 4.45 | 7.46 | 13.99 | 22.57 | 31.88 | 44.94 | 49.89 | 41.10 | 25.99 | 12.21 | 3.57 | 13.34 |
| Suriname | 0.00 | 0.00 | 0.00 | 41.25 | 66.37 | 116.06 | 208.95 | 302.68 | 358.07 | 321.41 | 260.36 | 181.51 | 85.45 | 24.30 | 101.45 |
| Trinidad and Tobago | 0.00 | 0.00 | 0.00 | 21.69 | 39.55 | 67.31 | 102.05 | 141.42 | 215.20 | 245.97 | 233.06 | 159.21 | 63.33 | 18.06 | 57.83 |
| Lao People's Democratic Republic | 0.00 | 0.00 | 0.00 | 28.54 | 38.76 | 66.58 | 102.93 | 120.73 | 121.31 | 113.14 | 88.46 | 57.69 | 29.99 | 8.26 | 45.14 |
| Bolivia (Plurinational State of) | 0.00 | 0.00 | 0.00 | 1.26 | 2.05 | 3.68 | 5.93 | 8.00 | 10.21 | 10.90 | 9.33 | 6.53 | 2.86 | 0.67 | 2.35 |
| Ecuador | 0.00 | 0.00 | 0.00 | 11.69 | 21.02 | 39.00 | 69.64 | 100.03 | 130.65 | 126.15 | 104.55 | 76.82 | 37.24 | 12.77 | 24.29 |
| Peru | 0.00 | 0.00 | 0.00 | 14.50 | 25.44 | 48.29 | 81.53 | 114.30 | 137.25 | 142.02 | 119.14 | 84.34 | 39.64 | 14.44 | 30.72 |
| Colombia | 0.00 | 0.00 | 0.00 | 1.28 | 2.23 | 3.74 | 6.35 | 9.65 | 12.68 | 12.95 | 11.36 | 8.17 | 3.97 | 1.44 | 2.96 |
| Costa Rica | 0.00 | 0.00 | 0.00 | 0.79 | 1.25 | 2.24 | 3.50 | 4.79 | 6.09 | 6.30 | 5.43 | 3.90 | 1.73 | 0.47 | 1.65 |
| El Salvador | 0.00 | 0.00 | 0.00 | 7.60 | 12.91 | 25.31 | 41.14 | 49.35 | 56.62 | 59.53 | 56.70 | 45.39 | 23.60 | 7.20 | 17.49 |
| Guatemala | 0.00 | 0.00 | 0.00 | 19.07 | 30.57 | 45.16 | 64.26 | 80.03 | 99.95 | 103.92 | 88.94 | 62.92 | 28.75 | 8.62 | 24.61 |
| Honduras | 0.00 | 0.00 | 0.00 | 3.36 | 5.57 | 10.55 | 18.57 | 25.31 | 30.99 | 31.38 | 27.72 | 20.68 | 9.12 | 2.27 | 5.16 |
| Malaysia | 0.00 | 0.00 | 0.00 | 27.88 | 45.67 | 78.25 | 135.19 | 212.15 | 289.39 | 308.64 | 254.72 | 168.07 | 67.50 | 24.49 | 45.57 |
| Mexico | 0.00 | 0.00 | 0.00 | 27.01 | 49.15 | 98.47 | 177.48 | 233.30 | 275.00 | 272.67 | 218.12 | 175.69 | 109.70 | 45.95 | 66.18 |
| Nicaragua | 0.00 | 0.00 | 0.00 | 0.36 | 0.57 | 0.90 | 1.32 | 1.77 | 2.21 | 2.19 | 1.93 | 1.32 | 0.60 | 0.17 | 0.57 |
| Panama | 0.00 | 0.00 | 0.00 | 3.84 | 6.93 | 13.56 | 25.48 | 36.89 | 46.82 | 49.38 | 46.63 | 37.11 | 18.71 | 7.65 | 8.95 |
| Venezuela (Bolivarian Republic of) | 0.00 | 0.00 | 0.00 | 49.77 | 90.70 | 163.78 | 249.95 | 346.76 | 448.75 | 482.85 | 415.06 | 264.04 | 109.05 | 30.57 | 109.95 |
| Brazil | 0.00 | 0.00 | 0.00 | 7.77 | 12.97 | 23.56 | 39.61 | 55.56 | 71.31 | 72.23 | 58.68 | 41.68 | 21.12 | 7.65 | 19.23 |
| Paraguay | 0.00 | 0.00 | 0.00 | 0.23 | 0.34 | 0.57 | 0.92 | 1.23 | 1.57 | 1.57 | 1.31 | 0.88 | 0.43 | 0.13 | 0.44 |
| Algeria | 0.00 | 0.00 | 0.00 | 0.49 | 0.89 | 1.50 | 2.45 | 3.31 | 3.94 | 4.15 | 4.03 | 2.82 | 1.56 | 0.48 | 0.90 |
| Maldives | 0.00 | 0.00 | 0.00 | 0.01 | 0.01 | 0.02 | 0.03 | 0.03 | 0.04 | 0.04 | 0.03 | 0.02 | 0.01 | 0.00 | 0.01 |
| Bahrain | 0.00 | 0.00 | 0.00 | 0.30 | 0.94 | 1.61 | 2.19 | 2.59 | 2.95 | 2.55 | 1.49 | 0.74 | 0.25 | 0.07 | 0.72 |
| Egypt | 0.00 | 0.00 | 0.00 | 841.53 | 1675.03 | 3408.36 | 6017.15 | 9114.04 | 11680.65 | 13069.28 | 15043.49 | 10497.34 | 3608.61 | 753.41 | 2334.97 |
| Iran (Islamic Republic of) | 0.00 | 0.00 | 0.00 | 454.76 | 455.42 | 741.46 | 1342.11 | 1958.27 | 2437.59 | 3050.53 | 3540.89 | 4113.93 | 2673.07 | 1750.07 | 827.14 |
| Iraq | 0.00 | 0.00 | 0.00 | 2.93 | 4.51 | 8.70 | 15.23 | 16.36 | 17.02 | 17.24 | 15.96 | 13.17 | 6.31 | 2.53 | 4.09 |
| Jordan | 0.00 | 0.00 | 0.00 | 0.75 | 1.36 | 2.64 | 4.51 | 5.62 | 6.00 | 4.90 | 4.31 | 3.66 | 1.69 | 0.32 | 1.61 |
| Kuwait | 0.00 | 0.00 | 0.00 | 1.49 | 3.34 | 5.59 | 7.20 | 9.46 | 8.74 | 7.30 | 4.63 | 3.79 | 1.53 | 0.72 | 1.86 |
| Lebanon | 0.00 | 0.00 | 0.00 | 4.01 | 6.32 | 10.19 | 16.73 | 22.61 | 32.82 | 38.17 | 43.74 | 36.11 | 19.18 | 9.57 | 6.35 |
| Libya | 0.00 | 0.00 | 0.00 | 7.92 | 15.05 | 28.22 | 51.16 | 66.99 | 92.00 | 104.12 | 120.65 | 86.76 | 53.18 | 13.75 | 21.91 |
| Morocco | 0.00 | 0.00 | 0.00 | 38.57 | 77.88 | 149.43 | 223.58 | 305.61 | 369.49 | 406.71 | 296.66 | 167.50 | 73.50 | 21.68 | 90.52 |
| Palestine | 0.00 | 0.00 | 0.00 | 3.53 | 5.06 | 8.06 | 12.78 | 19.16 | 25.30 | 25.48 | 23.93 | 16.71 | 7.07 | 1.82 | 5.88 |
| Myanmar | 0.00 | 0.00 | 0.00 | 8.13 | 13.27 | 23.29 | 38.97 | 53.80 | 64.37 | 67.10 | 65.28 | 51.67 | 23.06 | 8.10 | 16.95 |
| Oman | 0.00 | 0.00 | 0.00 | 15.74 | 25.82 | 33.13 | 41.45 | 42.65 | 46.90 | 42.77 | 32.69 | 25.54 | 10.14 | 2.27 | 10.19 |
| Qatar | 0.00 | 0.00 | 0.00 | 1.07 | 1.54 | 2.09 | 2.70 | 2.60 | 2.56 | 1.74 | 0.95 | 0.46 | 0.13 | 0.02 | 0.84 |
| Saudi Arabia | 0.00 | 0.00 | 0.00 | 49.33 | 119.39 | 267.47 | 462.92 | 563.94 | 612.71 | 652.28 | 588.53 | 353.47 | 154.90 | 66.25 | 115.60 |
| Syrian Arab Republic | 0.00 | 0.00 | 0.00 | 9.16 | 33.03 | 77.70 | 162.81 | 294.02 | 400.04 | 562.26 | 816.21 | 871.26 | 732.25 | 516.58 | 67.99 |
| Tunisia | 0.00 | 0.00 | 0.00 | 1.46 | 2.74 | 5.05 | 9.41 | 17.84 | 28.94 | 38.36 | 42.29 | 38.60 | 24.79 | 10.54 | 5.02 |
| Turkey | 0.00 | 0.00 | 0.00 | 74.69 | 87.71 | 147.56 | 202.20 | 260.89 | 331.35 | 375.76 | 343.65 | 308.40 | 133.34 | 68.39 | 94.46 |
| United Arab Emirates | 0.00 | 0.00 | 0.00 | 13.98 | 27.69 | 46.17 | 52.88 | 44.20 | 27.41 | 18.84 | 10.18 | 3.60 | 1.06 | 0.19 | 15.72 |
| Yemen | 0.00 | 0.00 | 0.00 | 1.77 | 2.72 | 3.67 | 4.76 | 5.12 | 5.48 | 5.70 | 5.48 | 3.95 | 1.98 | 0.45 | 2.12 |
| Philippines | 0.00 | 0.00 | 0.00 | 22.67 | 37.28 | 74.59 | 156.42 | 253.16 | 337.68 | 350.32 | 284.81 | 195.46 | 103.27 | 52.21 | 38.14 |
| Afghanistan | 0.00 | 0.00 | 0.00 | 0.08 | 0.12 | 0.20 | 0.45 | 0.48 | 0.32 | 0.22 | 0.25 | 0.22 | 0.11 | 0.04 | 0.10 |
| Bangladesh | 0.00 | 0.00 | 0.00 | 0.85 | 1.40 | 1.95 | 2.33 | 2.83 | 3.42 | 3.55 | 3.28 | 2.79 | 1.47 | 0.57 | 0.92 |
| Bhutan | 0.00 | 0.00 | 0.00 | 0.09 | 0.15 | 0.18 | 0.20 | 0.23 | 0.28 | 0.30 | 0.31 | 0.27 | 0.15 | 0.05 | 0.08 |
| India | 0.00 | 0.00 | 0.00 | 517.64 | 995.09 | 1381.87 | 1280.04 | 1932.64 | 4161.31 | 6539.68 | 5060.49 | 3467.27 | 1828.67 | 850.48 | 419.31 |
| Nepal | 0.00 | 0.00 | 0.00 | 1.82 | 3.16 | 4.14 | 4.96 | 5.99 | 7.30 | 7.81 | 7.64 | 5.31 | 2.40 | 0.76 | 2.01 |
| Pakistan | 0.00 | 0.00 | 0.00 | 2420.75 | 3989.83 | 6034.20 | 8042.96 | 9423.43 | 10529.66 | 9969.27 | 7971.12 | 5557.11 | 2794.99 | 1056.06 | 2657.18 |
| Angola | 0.00 | 0.00 | 0.00 | 5.99 | 9.84 | 18.69 | 28.08 | 36.52 | 42.12 | 35.62 | 26.14 | 14.36 | 7.26 | 1.78 | 8.37 |
| Central African Republic | 0.00 | 0.00 | 0.00 | 0.14 | 0.25 | 0.48 | 0.80 | 1.11 | 1.33 | 1.30 | 0.98 | 0.64 | 0.27 | 0.07 | 0.20 |
| Sri Lanka | 0.00 | 0.00 | 0.00 | 0.13 | 0.26 | 0.52 | 0.98 | 1.66 | 2.53 | 3.06 | 3.14 | 2.47 | 1.09 | 0.39 | 0.30 |
| Congo | 0.00 | 0.00 | 0.00 | 0.35 | 0.72 | 1.48 | 2.59 | 3.25 | 3.73 | 3.66 | 3.09 | 2.36 | 1.04 | 0.29 | 0.51 |
| Democratic Republic of the Congo | 0.00 | 0.00 | 0.00 | 20.35 | 36.88 | 62.31 | 113.56 | 156.17 | 193.05 | 206.79 | 167.91 | 142.87 | 57.46 | 23.44 | 24.05 |
| Equatorial Guinea | 0.00 | 0.00 | 0.00 | 2.60 | 4.15 | 6.62 | 10.43 | 13.49 | 16.37 | 16.75 | 16.62 | 11.29 | 6.58 | 2.30 | 2.48 |
| Gabon | 0.00 | 0.00 | 0.00 | 0.77 | 1.14 | 2.04 | 3.35 | 4.04 | 4.35 | 3.66 | 2.54 | 1.38 | 0.73 | 0.21 | 1.31 |
| Burundi | 0.00 | 0.00 | 0.00 | 0.85 | 1.56 | 2.71 | 4.19 | 5.81 | 7.60 | 8.27 | 7.29 | 4.74 | 2.17 | 0.67 | 1.00 |
| Comoros | 0.00 | 0.00 | 0.00 | 3.63 | 7.60 | 18.03 | 34.63 | 50.74 | 65.88 | 69.23 | 55.99 | 55.94 | 23.21 | 7.91 | 6.54 |
| Djibouti | 0.00 | 0.00 | 0.00 | 5.87 | 11.01 | 20.32 | 31.46 | 38.89 | 43.58 | 43.10 | 32.99 | 26.69 | 9.04 | 1.94 | 8.73 |
| Eritrea | 0.00 | 0.00 | 0.00 | 0.12 | 0.20 | 0.38 | 0.65 | 0.91 | 1.11 | 1.07 | 0.83 | 0.50 | 0.24 | 0.06 | 0.13 |
| Ethiopia | 0.00 | 0.00 | 0.00 | 64.04 | 110.68 | 217.95 | 357.30 | 445.47 | 517.47 | 529.72 | 491.19 | 447.42 | 253.28 | 95.08 | 71.05 |
| Thailand | 0.00 | 0.00 | 0.00 | 616.22 | 1311.35 | 2683.25 | 5181.07 | 8708.26 | 13058.20 | 14664.37 | 12067.47 | 7516.16 | 3563.25 | 1528.80 | 2066.71 |
| Kenya | 0.00 | 0.00 | 0.00 | 284.83 | 464.96 | 840.19 | 1444.04 | 2056.71 | 2745.06 | 2821.51 | 2340.93 | 1699.18 | 943.61 | 400.22 | 450.28 |
| Madagascar | 0.00 | 0.00 | 0.00 | 24.10 | 43.32 | 82.54 | 142.31 | 200.97 | 257.94 | 268.32 | 219.48 | 170.74 | 67.39 | 25.17 | 33.47 |
| Malawi | 0.00 | 0.00 | 0.00 | 1.26 | 2.19 | 4.00 | 7.13 | 9.30 | 11.66 | 12.83 | 11.32 | 7.72 | 3.05 | 0.89 | 1.41 |
| Mauritius | 0.00 | 0.00 | 0.00 | 0.12 | 0.20 | 0.34 | 0.49 | 0.76 | 1.07 | 1.10 | 1.03 | 0.67 | 0.28 | 0.08 | 0.37 |
| Mozambique | 0.00 | 0.00 | 0.00 | 3.14 | 5.68 | 10.71 | 18.67 | 25.05 | 30.68 | 32.46 | 29.21 | 21.00 | 9.13 | 2.80 | 4.42 |
| Rwanda | 0.00 | 0.00 | 0.00 | 3.82 | 6.73 | 11.14 | 17.19 | 24.93 | 34.57 | 38.83 | 36.88 | 24.21 | 11.99 | 3.97 | 4.69 |
| Seychelles | 0.00 | 0.00 | 0.00 | 0.05 | 0.10 | 0.21 | 0.44 | 0.80 | 1.17 | 1.10 | 0.73 | 0.52 | 0.30 | 0.12 | 0.10 |
| Somalia | 0.00 | 0.00 | 0.00 | 83.53 | 127.42 | 213.94 | 270.69 | 188.17 | 200.54 | 221.98 | 185.49 | 120.10 | 47.73 | 8.26 | 104.32 |
| United Republic of Tanzania | 0.00 | 0.00 | 0.00 | 15.82 | 28.56 | 55.15 | 93.39 | 134.64 | 167.22 | 160.58 | 133.99 | 94.55 | 50.46 | 16.68 | 18.44 |
| Timor-Leste | 0.00 | 0.00 | 0.00 | 0.41 | 0.62 | 1.38 | 2.83 | 3.50 | 4.01 | 5.59 | 6.58 | 5.67 | 2.42 | 0.78 | 0.72 |
| Uganda | 0.00 | 0.00 | 0.00 | 2023.67 | 2848.80 | 4344.03 | 6082.42 | 7094.44 | 7773.54 | 7354.97 | 7050.48 | 5316.32 | 2142.56 | 516.77 | 2834.95 |
| Zambia | 0.00 | 0.00 | 0.00 | 7.98 | 12.31 | 20.62 | 40.13 | 72.93 | 77.68 | 63.86 | 48.51 | 43.83 | 24.95 | 9.41 | 7.53 |
| Botswana | 0.00 | 0.00 | 0.00 | 25.61 | 55.84 | 88.12 | 122.23 | 165.83 | 216.06 | 156.34 | 96.09 | 61.24 | 30.31 | 8.83 | 30.84 |
| Lesotho | 0.00 | 0.00 | 0.00 | 0.61 | 1.27 | 2.28 | 3.58 | 5.38 | 7.27 | 7.96 | 7.25 | 4.51 | 1.80 | 0.47 | 0.74 |
| Namibia | 0.00 | 0.00 | 0.00 | 1.23 | 2.27 | 4.54 | 8.42 | 12.30 | 16.04 | 16.74 | 15.15 | 10.11 | 5.09 | 1.70 | 1.76 |
| South Africa | 0.00 | 0.00 | 0.00 | 7.90 | 14.28 | 27.49 | 48.31 | 67.14 | 83.05 | 80.33 | 59.62 | 40.01 | 19.84 | 7.05 | 15.99 |
| Eswatini | 0.00 | 0.00 | 0.00 | 4.14 | 7.00 | 13.77 | 24.19 | 33.64 | 38.30 | 38.87 | 32.86 | 25.00 | 11.27 | 2.43 | 7.39 |
| Zimbabwe | 0.00 | 0.00 | 0.00 | 46.38 | 86.54 | 178.12 | 310.62 | 345.40 | 376.32 | 397.99 | 328.42 | 226.80 | 103.84 | 31.49 | 75.86 |
| Viet Nam | 0.00 | 0.00 | 0.00 | 8.86 | 15.60 | 29.64 | 52.35 | 77.11 | 101.84 | 100.08 | 80.85 | 53.91 | 27.90 | 11.52 | 20.92 |
| Benin | 0.00 | 0.00 | 0.00 | 63.67 | 112.85 | 223.17 | 386.04 | 505.54 | 583.06 | 539.32 | 412.94 | 309.63 | 158.88 | 64.61 | 89.33 |
| Burkina Faso | 0.00 | 0.00 | 0.00 | 9.09 | 15.08 | 27.33 | 47.30 | 70.11 | 85.48 | 77.67 | 59.57 | 48.90 | 24.70 | 8.25 | 11.44 |
| Cameroon | 0.00 | 0.00 | 0.00 | 0.58 | 1.02 | 1.78 | 2.84 | 3.76 | 4.67 | 4.78 | 3.92 | 2.88 | 1.18 | 0.35 | 0.62 |
| Cabo Verde | 0.00 | 0.00 | 0.00 | 0.05 | 0.07 | 0.11 | 0.17 | 0.24 | 0.28 | 0.29 | 0.25 | 0.17 | 0.08 | 0.04 | 0.08 |
| Chad | 0.00 | 0.00 | 0.00 | 6.75 | 11.77 | 21.03 | 35.54 | 49.01 | 61.09 | 62.60 | 58.91 | 41.47 | 18.13 | 5.87 | 7.33 |
| Côte d'Ivoire | 0.00 | 0.00 | 0.00 | 10.06 | 18.09 | 31.88 | 51.39 | 66.11 | 78.78 | 77.27 | 63.34 | 41.19 | 18.74 | 5.76 | 10.31 |
| Gambia | 0.00 | 0.00 | 0.00 | 0.52 | 0.95 | 1.75 | 2.83 | 3.75 | 4.43 | 4.74 | 4.32 | 3.27 | 1.88 | 0.64 | 0.62 |
| Ghana | 0.00 | 0.00 | 0.00 | 345.78 | 638.64 | 1280.97 | 2014.71 | 2268.50 | 2487.18 | 2494.37 | 2382.67 | 1901.18 | 733.84 | 107.71 | 603.46 |
| Guinea | 0.00 | 0.00 | 0.00 | 6.12 | 10.38 | 17.73 | 29.61 | 41.01 | 53.00 | 59.34 | 52.22 | 37.83 | 18.01 | 6.67 | 5.67 |
| Guinea-Bissau | 0.00 | 0.00 | 0.00 | 0.46 | 0.74 | 1.28 | 2.12 | 2.93 | 3.60 | 3.83 | 3.54 | 2.71 | 1.24 | 0.36 | 0.52 |
| Liberia | 0.00 | 0.00 | 0.00 | 1.02 | 1.97 | 3.97 | 6.95 | 7.42 | 8.69 | 7.86 | 6.66 | 4.96 | 2.27 | 0.79 | 1.50 |
| Mali | 0.00 | 0.00 | 0.00 | 9.75 | 17.60 | 33.55 | 55.64 | 77.34 | 94.27 | 92.63 | 77.52 | 56.59 | 25.45 | 7.18 | 11.70 |
| Mauritania | 0.00 | 0.00 | 0.00 | 14.85 | 28.33 | 58.87 | 108.98 | 154.65 | 199.40 | 213.55 | 187.47 | 134.76 | 69.71 | 27.54 | 22.96 |
| Niger | 0.00 | 0.00 | 0.00 | 7.31 | 12.15 | 19.86 | 29.68 | 41.95 | 56.91 | 62.20 | 56.38 | 41.97 | 22.28 | 6.08 | 9.84 |
| Nigeria | 0.00 | 0.00 | 0.00 | 291.94 | 440.23 | 729.25 | 1196.58 | 2110.21 | 2623.12 | 2646.42 | 2110.87 | 1411.28 | 917.47 | 316.85 | 339.93 |
| Sao Tome and Principe | 0.00 | 0.00 | 0.00 | 0.09 | 0.18 | 0.34 | 0.57 | 0.72 | 0.88 | 0.84 | 0.66 | 0.43 | 0.23 | 0.08 | 0.10 |
| Senegal | 0.00 | 0.00 | 0.00 | 27.12 | 47.34 | 84.42 | 137.05 | 189.44 | 237.59 | 233.42 | 192.03 | 139.97 | 62.85 | 18.48 | 34.61 |
| Sierra Leone | 0.00 | 0.00 | 0.00 | 3.41 | 5.64 | 10.35 | 17.44 | 21.18 | 23.26 | 23.73 | 22.73 | 17.75 | 9.33 | 3.82 | 2.78 |
| Togo | 0.00 | 0.00 | 0.00 | 0.29 | 0.52 | 0.95 | 1.53 | 2.01 | 2.45 | 2.50 | 2.38 | 1.80 | 0.66 | 0.18 | 0.33 |
| Fiji | 0.00 | 0.00 | 0.00 | 36.26 | 68.80 | 128.65 | 229.51 | 403.89 | 581.09 | 600.28 | 492.06 | 357.37 | 138.50 | 29.63 | 76.77 |
| Kiribati | 0.00 | 0.00 | 0.00 | 0.06 | 0.11 | 0.21 | 0.33 | 0.51 | 0.70 | 0.70 | 0.47 | 0.29 | 0.13 | 0.03 | 0.09 |
| Marshall Islands | 0.00 | 0.00 | 0.00 | 0.05 | 0.11 | 0.20 | 0.34 | 0.50 | 0.68 | 0.72 | 0.58 | 0.33 | 0.10 | 0.03 | 0.08 |
| Micronesia (Federated States of) | 0.00 | 0.00 | 0.00 | 0.09 | 0.16 | 0.33 | 0.62 | 0.97 | 1.49 | 1.80 | 1.10 | 0.69 | 0.26 | 0.11 | 0.18 |
| Papua New Guinea | 0.00 | 0.00 | 0.00 | 63.73 | 92.90 | 164.32 | 257.32 | 292.32 | 307.34 | 302.52 | 255.16 | 200.05 | 83.96 | 29.88 | 88.60 |
| Samoa | 0.00 | 0.00 | 0.00 | 12.16 | 19.74 | 38.87 | 83.35 | 111.27 | 132.51 | 133.72 | 125.71 | 96.50 | 54.47 | 32.20 | 24.08 |
| Solomon Islands | 0.00 | 0.00 | 0.00 | 0.00 | 0.00 | 0.00 | 0.01 | 0.01 | 0.01 | 0.01 | 0.00 | 0.00 | 0.00 | 0.00 | 0.00 |
| Tonga | 0.00 | 0.00 | 0.00 | 10.85 | 18.20 | 36.15 | 70.28 | 107.10 | 130.97 | 133.72 | 121.65 | 112.91 | 84.60 | 48.19 | 25.01 |
| American Samoa | 0.00 | 0.00 | 0.00 | 0.00 | 0.00 | 0.00 | 0.00 | 0.00 | 0.00 | 0.00 | 0.00 | 0.00 | 0.00 | 0.00 | 0.00 |
| Vanuatu | 0.00 | 0.00 | 0.00 | 0.00 | 0.00 | 0.01 | 0.01 | 0.01 | 0.02 | 0.02 | 0.01 | 0.01 | 0.00 | 0.00 | 0.00 |
| Bermuda | 0.00 | 0.00 | 0.00 | 0.00 | 0.00 | 0.00 | 0.01 | 0.01 | 0.02 | 0.02 | 0.02 | 0.02 | 0.01 | 0.00 | 0.00 |
| Cook Islands | 0.00 | 0.00 | 0.00 | 0.00 | 0.00 | 0.00 | 0.00 | 0.00 | 0.00 | 0.00 | 0.00 | 0.00 | 0.00 | 0.00 | 0.00 |
| Armenia | 0.00 | 0.00 | 0.00 | 316.16 | 571.71 | 958.60 | 1548.44 | 2501.68 | 4237.52 | 4784.23 | 5144.02 | 3559.17 | 2188.93 | 954.73 | 824.84 |
| Azerbaijan | 0.00 | 0.00 | 0.00 | 11.73 | 20.06 | 36.79 | 74.10 | 137.58 | 232.03 | 236.73 | 159.98 | 76.43 | 48.83 | 19.61 | 26.33 |
| Greenland | 0.00 | 0.00 | 0.00 | 3.59 | 7.44 | 12.76 | 24.52 | 56.09 | 65.94 | 68.10 | 67.04 | 51.73 | 17.89 | 1.57 | 11.34 |
| Georgia | 0.00 | 0.00 | 0.00 | 50.90 | 95.89 | 194.42 | 400.18 | 757.25 | 1426.99 | 1828.28 | 1723.41 | 1156.85 | 717.23 | 456.04 | 169.14 |
| Guam | 0.00 | 0.00 | 0.00 | 14.95 | 25.12 | 50.47 | 101.54 | 154.68 | 219.05 | 223.60 | 176.51 | 120.24 | 41.81 | 13.49 | 37.12 |
| Kazakhstan | 0.00 | 0.00 | 0.00 | 4887.20 | 7326.18 | 12537.34 | 20452.46 | 25731.56 | 38051.28 | 38881.76 | 31199.56 | 16327.09 | 8556.37 | 2343.90 | 8435.71 |
| Monaco | 0.00 | 0.00 | 0.00 | 1.37 | 3.43 | 8.59 | 20.32 | 42.07 | 64.07 | 71.71 | 78.66 | 87.08 | 33.40 | 7.86 | 9.32 |
| Nauru | 0.00 | 0.00 | 0.00 | 1.56 | 2.03 | 2.94 | 3.81 | 3.97 | 3.76 | 2.86 | 1.34 | 0.59 | 0.18 | 0.03 | 1.71 |
| Kyrgyzstan | 0.00 | 0.00 | 0.00 | 0.13 | 0.18 | 0.32 | 0.55 | 0.77 | 0.84 | 0.65 | 0.42 | 0.21 | 0.11 | 0.03 | 0.26 |
| Niue | 0.00 | 0.00 | 0.00 | 0.00 | 0.00 | 0.00 | 0.00 | 0.00 | 0.00 | 0.00 | 0.00 | 0.00 | 0.00 | 0.00 | 0.00 |
| Northern Mariana Islands | 0.00 | 0.00 | 0.00 | 12.65 | 22.84 | 70.97 | 132.08 | 231.73 | 224.72 | 245.34 | 134.30 | 66.61 | 20.84 | 5.37 | 67.38 |
| Mongolia | 0.00 | 0.00 | 0.00 | 9.48 | 15.42 | 33.05 | 57.78 | 69.48 | 59.90 | 42.61 | 25.17 | 20.58 | 9.66 | 1.63 | 20.06 |
| Palau | 0.00 | 0.00 | 0.00 | 0.01 | 0.03 | 0.06 | 0.12 | 0.15 | 0.17 | 0.17 | 0.17 | 0.15 | 0.04 | 0.01 | 0.05 |
| Puerto Rico | 0.00 | 0.00 | 0.00 | 467.12 | 948.65 | 2441.04 | 3850.58 | 3569.76 | 5116.49 | 6599.83 | 7380.75 | 6906.88 | 3212.02 | 1255.52 | 2067.23 |
| Tajikistan | 0.00 | 0.00 | 0.00 | 21.32 | 29.20 | 46.65 | 73.98 | 96.46 | 98.91 | 75.49 | 43.66 | 23.01 | 8.49 | 1.43 | 32.36 |
| Saint Kitts and Nevis | 0.00 | 0.00 | 0.00 | 3.47 | 6.27 | 11.45 | 18.02 | 24.02 | 31.87 | 34.44 | 24.11 | 12.26 | 6.00 | 1.30 | 8.45 |
| San Marino | 0.00 | 0.00 | 0.00 | 0.11 | 0.19 | 0.34 | 0.57 | 0.90 | 1.06 | 0.91 | 0.61 | 0.43 | 0.16 | 0.02 | 0.36 |
| Turkmenistan | 0.00 | 0.00 | 0.00 | 64.36 | 79.56 | 118.00 | 169.56 | 220.02 | 258.45 | 173.58 | 78.12 | 28.66 | 12.50 | 2.09 | 80.30 |
| Uzbekistan | 0.00 | 0.00 | 0.00 | 408.22 | 512.73 | 721.41 | 1017.15 | 1363.91 | 1562.58 | 1027.01 | 450.85 | 148.35 | 53.17 | 4.25 | 479.68 |
| Tokelau | 0.00 | 0.00 | 0.00 | 0.01 | 0.01 | 0.01 | 0.02 | 0.02 | 0.03 | 0.02 | 0.02 | 0.01 | 0.00 | 0.00 | 0.01 |
| Tuvalu | 0.00 | 0.00 | 0.00 | 0.15 | 0.18 | 0.24 | 0.35 | 0.47 | 0.49 | 0.37 | 0.21 | 0.12 | 0.04 | 0.01 | 0.18 |
| United States Virgin Islands | 0.00 | 0.00 | 0.00 | 0.59 | 0.97 | 1.55 | 2.70 | 4.48 | 5.25 | 4.46 | 2.91 | 2.22 | 0.73 | 0.08 | 1.79 |
| Albania | 0.00 | 0.00 | 0.00 | 10.44 | 13.78 | 21.76 | 37.15 | 66.52 | 98.18 | 84.96 | 44.60 | 24.92 | 9.98 | 2.06 | 27.56 |
| South Sudan | 0.00 | 0.00 | 0.00 | 31.59 | 48.57 | 69.67 | 91.66 | 118.44 | 114.00 | 76.62 | 38.46 | 21.11 | 7.78 | 1.37 | 41.46 |
| Bosnia and Herzegovina | 0.00 | 0.00 | 0.00 | 18.66 | 29.85 | 52.35 | 76.10 | 131.04 | 199.53 | 182.48 | 107.42 | 48.49 | 19.19 | 3.99 | 57.09 |
| Bulgaria | 0.00 | 0.00 | 0.00 | 85.80 | 137.89 | 236.53 | 350.66 | 546.89 | 750.03 | 739.27 | 469.84 | 260.74 | 97.60 | 16.62 | 240.72 |
| Croatia | 0.00 | 0.00 | 0.00 | 42.97 | 75.57 | 116.02 | 175.17 | 324.32 | 444.14 | 441.69 | 271.29 | 120.97 | 58.82 | 11.97 | 132.58 |
| Czechia | 0.00 | 0.00 | 0.00 | 167.78 | 282.46 | 565.10 | 855.66 | 1121.69 | 1341.87 | 1303.89 | 930.84 | 572.32 | 217.32 | 31.30 | 483.13 |
| Hungary | 0.00 | 0.00 | 0.00 | 143.18 | 257.99 | 547.19 | 714.22 | 946.03 | 1123.15 | 1299.76 | 838.80 | 443.24 | 177.88 | 31.12 | 437.24 |
| North Macedonia | 0.00 | 0.00 | 0.00 | 15.65 | 24.15 | 34.92 | 52.08 | 82.45 | 108.14 | 98.64 | 58.98 | 27.51 | 9.62 | 1.23 | 33.55 |
| Montenegro | 0.00 | 0.00 | 0.00 | 2.02 | 3.36 | 5.41 | 7.89 | 12.64 | 18.10 | 17.19 | 10.17 | 4.33 | 1.75 | 0.34 | 5.48 |
| Poland | 0.00 | 0.00 | 0.00 | 67.08 | 117.57 | 185.00 | 241.79 | 364.02 | 597.67 | 744.70 | 437.55 | 190.15 | 66.95 | 24.57 | 188.23 |
| Romania | 0.00 | 0.00 | 0.00 | 127.36 | 202.77 | 400.47 | 587.34 | 915.13 | 971.53 | 1231.61 | 765.49 | 353.16 | 137.64 | 32.29 | 377.00 |
| Sudan | 0.00 | 0.00 | 0.00 | 234.09 | 308.81 | 358.28 | 426.45 | 531.34 | 472.39 | 417.33 | 244.32 | 133.20 | 52.94 | 12.11 | 205.32 |
| Serbia | 0.00 | 0.00 | 0.00 | 38.54 | 66.88 | 108.62 | 154.79 | 245.02 | 343.55 | 412.68 | 268.61 | 108.47 | 45.78 | 8.19 | 112.49 |
| Slovakia | 0.00 | 0.00 | 0.00 | 7.78 | 13.51 | 23.38 | 29.63 | 43.44 | 68.81 | 79.11 | 45.16 | 19.37 | 6.69 | 1.46 | 21.45 |
| Slovenia | 0.00 | 0.00 | 0.00 | 4.52 | 8.01 | 13.42 | 18.88 | 32.58 | 45.17 | 46.09 | 27.80 | 12.14 | 5.70 | 1.36 | 14.10 |
| Belarus | 0.00 | 0.00 | 0.00 | 155.69 | 235.08 | 355.16 | 512.03 | 775.92 | 1214.07 | 1039.86 | 553.92 | 228.24 | 85.50 | 22.03 | 342.93 |
| Estonia | 0.00 | 0.00 | 0.00 | 33.81 | 48.43 | 80.31 | 134.73 | 200.88 | 263.80 | 218.35 | 139.76 | 76.70 | 37.40 | 7.10 | 80.76 |
| Latvia | 0.00 | 0.00 | 0.00 | 39.80 | 56.82 | 95.95 | 163.66 | 265.79 | 357.47 | 283.79 | 160.56 | 83.86 | 47.87 | 7.14 | 99.29 |
| China | 0.00 | 0.00 | 0.00 | 8200.57 | 8906.23 | 12840.16 | 22053.41 | 38054.58 | 39576.40 | 32591.36 | 22734.75 | 10836.69 | 4560.56 | 1177.82 | 12590.14 |
| Lithuania | 0.00 | 0.00 | 0.00 | 10.74 | 15.94 | 27.85 | 48.26 | 84.90 | 132.67 | 111.26 | 60.70 | 30.52 | 16.48 | 3.58 | 34.29 |
| Republic of Moldova | 0.00 | 0.00 | 0.00 | 46.62 | 67.23 | 99.71 | 137.07 | 215.29 | 325.83 | 265.84 | 144.07 | 46.47 | 24.23 | 4.70 | 87.88 |
| Russian Federation | 0.00 | 0.00 | 0.00 | 3472.22 | 5069.53 | 7625.46 | 11025.40 | 15538.91 | 24017.10 | 20062.23 | 10653.55 | 4657.90 | 1831.76 | 538.12 | 6906.42 |
| Ukraine | 0.00 | 0.00 | 0.00 | 729.68 | 1078.87 | 1504.67 | 2224.19 | 3607.27 | 5489.80 | 4738.10 | 2636.41 | 1051.17 | 547.95 | 146.43 | 1531.99 |
| Brunei Darussalam | 0.00 | 0.00 | 0.00 | 13.94 | 19.58 | 26.37 | 35.31 | 46.01 | 44.82 | 31.93 | 16.62 | 6.68 | 1.90 | 0.13 | 15.47 |
| Japan | 0.00 | 0.00 | 0.00 | 233.58 | 403.31 | 773.14 | 1379.67 | 2002.22 | 2493.69 | 2484.11 | 2180.39 | 1718.63 | 801.87 | 182.25 | 943.84 |
| Republic of Korea | 0.00 | 0.00 | 0.00 | 810.07 | 1602.15 | 2597.15 | 4861.96 | 7184.51 | 8869.96 | 7297.07 | 3969.28 | 2665.28 | 1055.20 | 99.16 | 2617.26 |
| Singapore | 0.00 | 0.00 | 0.00 | 75.19 | 165.79 | 211.69 | 293.61 | 410.89 | 453.55 | 370.89 | 237.25 | 135.45 | 43.30 | 4.54 | 157.86 |
| Democratic People's Republic of Korea | 0.00 | 0.00 | 0.00 | 110.52 | 146.17 | 202.00 | 362.40 | 518.00 | 469.10 | 365.40 | 209.71 | 160.20 | 78.25 | 14.07 | 166.68 |
| Australia | 0.00 | 0.00 | 0.00 | 503.83 | 775.79 | 1246.41 | 2278.38 | 3259.27 | 3830.12 | 3059.55 | 1980.86 | 1283.51 | 452.16 | 45.58 | 1217.98 |
| New Zealand | 0.00 | 0.00 | 0.00 | 49.58 | 74.57 | 135.99 | 266.62 | 394.38 | 479.49 | 364.08 | 237.21 | 159.25 | 60.52 | 7.73 | 146.83 |
| Andorra | 0.00 | 0.00 | 0.00 | 1.99 | 5.57 | 14.83 | 33.26 | 57.56 | 84.56 | 88.61 | 81.51 | 67.66 | 30.57 | 5.76 | 8.02 |
| Austria | 0.00 | 0.00 | 0.00 | 82.45 | 188.08 | 434.94 | 1073.38 | 2282.33 | 3326.45 | 3650.37 | 4105.06 | 4315.15 | 2946.49 | 632.68 | 352.57 |
| Belgium | 0.00 | 0.00 | 0.00 | 146.59 | 354.50 | 815.92 | 1821.54 | 3378.85 | 5334.79 | 6828.99 | 8099.25 | 6277.11 | 2024.68 | 283.46 | 552.47 |
| Cyprus | 0.00 | 0.00 | 0.00 | 29.19 | 67.30 | 134.63 | 241.78 | 395.84 | 590.27 | 760.70 | 841.24 | 721.26 | 296.95 | 41.79 | 70.68 |
| Denmark | 0.00 | 0.00 | 0.00 | 297.25 | 665.78 | 1758.45 | 3956.46 | 7752.77 | 10373.79 | 11868.66 | 13041.40 | 11651.79 | 3955.71 | 472.27 | 1328.11 |
| Finland | 0.00 | 0.00 | 0.00 | 123.14 | 253.58 | 522.88 | 954.38 | 1820.63 | 2959.98 | 4137.84 | 5156.70 | 5211.53 | 1965.16 | 365.75 | 608.58 |
| Taiwan (Province of China) | 0.00 | 0.00 | 0.00 | 162.16 | 404.11 | 887.32 | 1661.59 | 3074.68 | 5444.21 | 6932.40 | 7293.96 | 5188.58 | 3753.45 | 3197.89 | 412.48 |
| France | 0.00 | 0.00 | 0.00 | 753.21 | 1893.43 | 4689.47 | 11128.89 | 19381.23 | 28629.22 | 34992.13 | 40815.02 | 37245.74 | 15927.95 | 3590.86 | 3084.31 |
| Germany | 0.00 | 0.00 | 0.00 | 5778.46 | 13265.34 | 32658.10 | 77375.19 | 172517.80 | 286435.77 | 338985.79 | 368796.21 | 295884.12 | 195854.69 | 38300.19 | 28396.97 |
| Greece | 0.00 | 0.00 | 0.00 | 89.19 | 246.77 | 649.35 | 1483.16 | 2878.24 | 5183.85 | 8139.96 | 10763.46 | 11735.11 | 5960.09 | 1783.72 | 579.35 |
| Iceland | 0.00 | 0.00 | 0.00 | 2.31 | 5.37 | 11.23 | 22.59 | 39.17 | 61.19 | 74.54 | 79.45 | 73.66 | 33.61 | 7.95 | 7.89 |
| Ireland | 0.00 | 0.00 | 0.00 | 129.27 | 356.96 | 809.21 | 1691.07 | 2899.08 | 4286.71 | 5212.20 | 6175.40 | 4915.09 | 1778.58 | 224.80 | 542.14 |
| Israel | 0.00 | 0.00 | 0.00 | 49.00 | 100.94 | 222.42 | 428.42 | 664.35 | 1094.09 | 1661.78 | 2203.17 | 1924.05 | 642.32 | 153.07 | 195.93 |
| Italy | 0.00 | 0.00 | 0.00 | 505.73 | 1248.28 | 3459.61 | 8099.09 | 15398.90 | 21734.71 | 25048.38 | 29052.97 | 27883.85 | 14783.00 | 4479.00 | 2535.64 |
| Luxembourg | 0.00 | 0.00 | 0.00 | 14.54 | 28.13 | 57.11 | 110.03 | 187.00 | 271.88 | 342.03 | 359.03 | 297.08 | 147.19 | 29.22 | 57.94 |
| Malta | 0.00 | 0.00 | 0.00 | 5.24 | 12.37 | 28.33 | 51.40 | 77.66 | 126.48 | 173.65 | 219.91 | 249.34 | 93.17 | 21.14 | 21.29 |
| Netherlands | 0.00 | 0.00 | 0.00 | 174.04 | 395.88 | 1016.77 | 2843.57 | 5413.23 | 8252.66 | 9878.16 | 11449.22 | 11915.72 | 5031.03 | 889.24 | 915.87 |
| Norway | 0.00 | 0.00 | 0.00 | 105.36 | 220.14 | 493.40 | 1169.58 | 2230.18 | 3278.37 | 3885.50 | 4100.09 | 4023.41 | 1722.12 | 486.41 | 369.76 |
| Portugal | 0.00 | 0.00 | 0.00 | 265.83 | 666.09 | 1681.27 | 3222.26 | 5341.91 | 8389.51 | 11763.78 | 14829.22 | 12909.83 | 5717.56 | 1298.09 | 1445.31 |
| Spain | 0.00 | 0.00 | 0.00 | 4508.06 | 12227.94 | 30350.82 | 58627.26 | 104809.02 | 157357.78 | 191082.90 | 214459.75 | 204521.17 | 94244.51 | 25496.92 | 22983.26 |
| Sweden | 0.00 | 0.00 | 0.00 | 128.74 | 229.20 | 493.04 | 1089.65 | 1968.86 | 2846.54 | 3452.64 | 3990.12 | 4112.32 | 1912.13 | 428.65 | 523.82 |
| Switzerland | 0.00 | 0.00 | 0.00 | 102.42 | 221.18 | 446.87 | 931.64 | 1758.40 | 2499.81 | 2607.03 | 2691.89 | 2945.47 | 1618.98 | 356.16 | 370.33 |
| United Kingdom | 0.00 | 0.00 | 0.00 | 568.46 | 1250.23 | 2313.68 | 5073.16 | 10065.81 | 17979.32 | 22939.66 | 27881.88 | 39180.52 | 28027.00 | 18231.77 | 1387.16 |
| Argentina | 0.00 | 0.00 | 0.00 | 312.65 | 628.44 | 1410.48 | 2674.82 | 4034.39 | 5625.85 | 6654.87 | 6782.09 | 5478.96 | 2490.51 | 509.10 | 978.74 |
| Chile | 0.00 | 0.00 | 0.00 | 247.51 | 559.11 | 1325.64 | 3150.34 | 5739.20 | 8737.68 | 9633.77 | 8889.23 | 7414.04 | 3358.62 | 620.42 | 671.94 |
| Uruguay | - | - | - | - | - | - | - | - | - | - | - | - | - | - | - |

S Table12 Age distribution of DALYs (per 100,000) for hip osteoarthritis in different countries in 2019.

| location_name | 15 to 19 | 20 to 24 | 25 to 29 | 30 to 34 | 35 to 39 | 40 to 44 | 45 to 49 | 50 to 54 | 55 to 59 | 60 to 64 | 65 to 69 | 70 to 74 | 75 to 79 | 80 plus | All Ages |
| --- | --- | --- | --- | --- | --- | --- | --- | --- | --- | --- | --- | --- | --- | --- | --- |
| Afghanistan | 0.00 | 0.00 | 0.00 | 0.03 | 0.04 | 0.06 | 0.15 | 0.19 | 0.15 | 0.13 | 0.19 | 0.25 | 0.28 | 0.27 | 0.05 |
| Albania | 0.00 | 0.00 | 0.00 | 3.52 | 4.34 | 6.20 | 10.79 | 22.81 | 39.91 | 47.63 | 38.60 | 35.99 | 34.29 | 26.11 | 18.55 |
| Algeria | 0.00 | 0.00 | 0.00 | 0.16 | 0.28 | 0.46 | 0.80 | 1.30 | 1.90 | 2.60 | 3.40 | 3.64 | 4.54 | 4.20 | 0.51 |
| American Samoa | 0.00 | 0.00 | 0.00 | 0.00 | 0.00 | 0.00 | 0.00 | 0.00 | 0.00 | 0.00 | 0.00 | 0.00 | 0.00 | 0.00 | 0.00 |
| Andorra | 0.00 | 0.00 | 0.00 | 0.61 | 1.61 | 4.08 | 9.78 | 20.03 | 34.78 | 47.70 | 59.75 | 79.79 | 101.92 | 173.79 | 4.98 |
| Angola | 0.00 | 0.00 | 0.00 | 1.98 | 3.08 | 5.27 | 8.25 | 13.05 | 18.01 | 20.61 | 22.13 | 19.55 | 23.10 | 18.95 | 4.05 |
| Antigua and Barbuda | 0.00 | 0.00 | 0.00 | 0.00 | 0.01 | 0.02 | 0.03 | 0.04 | 0.06 | 0.09 | 0.10 | 0.11 | 0.11 | 0.09 | 0.02 |
| Argentina | 0.00 | 0.00 | 0.00 | 98.74 | 182.01 | 374.55 | 750.68 | 1375.50 | 2319.04 | 3696.08 | 5438.48 | 7235.84 | 8900.80 | 12389.02 | 634.63 |
| Armenia | 0.00 | 0.00 | 0.00 | 107.21 | 179.63 | 273.49 | 453.68 | 860.41 | 1749.85 | 2707.71 | 4471.46 | 5147.85 | 7534.71 | 12055.92 | 525.03 |
| Australia | 0.00 | 0.00 | 0.00 | 156.12 | 221.42 | 318.28 | 626.89 | 1092.11 | 1572.78 | 1722.82 | 1653.73 | 1774.20 | 1699.01 | 1212.34 | 856.18 |
| Austria | 0.00 | 0.00 | 0.00 | 25.58 | 53.93 | 119.48 | 318.03 | 789.16 | 1357.56 | 1929.30 | 2928.74 | 4915.10 | 9573.82 | 18311.88 | 253.88 |
| Azerbaijan | 0.00 | 0.00 | 0.00 | 3.94 | 6.36 | 10.37 | 21.49 | 47.51 | 95.63 | 134.87 | 142.29 | 113.20 | 172.08 | 237.83 | 13.52 |
| Bahamas | 0.00 | 0.00 | 0.00 | 11.20 | 18.28 | 31.04 | 54.33 | 84.88 | 130.66 | 179.06 | 199.43 | 210.63 | 239.30 | 250.47 | 51.15 |
| Bahrain | 0.00 | 0.00 | 0.00 | 0.10 | 0.29 | 0.49 | 0.70 | 1.02 | 1.41 | 1.60 | 1.30 | 0.98 | 0.75 | 0.64 | 0.33 |
| Bangladesh | 0.00 | 0.00 | 0.00 | 0.29 | 0.45 | 0.75 | 1.08 | 1.49 | 1.99 | 2.49 | 2.89 | 3.62 | 4.11 | 4.51 | 0.60 |
| Barbados | 0.00 | 0.00 | 0.00 | 1.80 | 3.20 | 5.35 | 10.33 | 18.47 | 32.51 | 53.19 | 75.58 | 101.53 | 100.92 | 118.44 | 14.42 |
| Belarus | 0.00 | 0.00 | 0.00 | 52.90 | 74.23 | 99.01 | 146.31 | 264.20 | 495.11 | 590.49 | 488.51 | 336.98 | 301.60 | 297.35 | 225.21 |
| Belgium | 0.00 | 0.00 | 0.00 | 45.36 | 102.31 | 224.05 | 538.23 | 1169.17 | 2166.90 | 3600.48 | 5755.27 | 7116.63 | 6527.86 | 8080.09 | 402.60 |
| Belize | 0.00 | 0.00 | 0.00 | 4.52 | 6.18 | 9.40 | 13.34 | 18.17 | 22.86 | 25.73 | 28.68 | 28.08 | 29.32 | 29.98 | 11.39 |
| Benin | 0.00 | 0.00 | 0.00 | 21.35 | 36.21 | 64.99 | 117.24 | 186.04 | 263.27 | 325.30 | 360.38 | 431.50 | 511.36 | 681.12 | 46.96 |
| Bermuda | 0.00 | 0.00 | 0.00 | 0.00 | 0.00 | 0.00 | 0.00 | 0.00 | 0.01 | 0.01 | 0.02 | 0.02 | 0.03 | 0.04 | 0.00 |
| Bhutan | 0.00 | 0.00 | 0.00 | 0.03 | 0.05 | 0.07 | 0.09 | 0.12 | 0.17 | 0.22 | 0.29 | 0.38 | 0.44 | 0.42 | 0.05 |
| Bolivia (Plurinational State of) | 0.00 | 0.00 | 0.00 | 0.43 | 0.69 | 1.12 | 1.84 | 2.89 | 4.36 | 6.22 | 7.71 | 8.53 | 8.58 | 6.52 | 1.35 |
| Bosnia and Herzegovina | 0.00 | 0.00 | 0.00 | 6.30 | 9.37 | 14.58 | 22.26 | 44.74 | 80.01 | 102.14 | 92.42 | 69.32 | 65.35 | 49.51 | 37.95 |
| Botswana | 0.00 | 0.00 | 0.00 | 8.29 | 16.63 | 23.45 | 33.97 | 57.21 | 92.53 | 93.58 | 88.88 | 93.94 | 109.00 | 111.84 | 15.16 |
| Brazil | 0.00 | 0.00 | 0.00 | 2.79 | 4.46 | 7.28 | 12.48 | 20.11 | 30.14 | 41.56 | 51.26 | 58.20 | 61.81 | 62.35 | 11.68 |
| Brunei Darussalam | 0.00 | 0.00 | 0.00 | 4.53 | 5.90 | 7.26 | 10.34 | 16.12 | 18.76 | 17.39 | 12.21 | 7.74 | 5.90 | 2.22 | 7.23 |
| Bulgaria | 0.00 | 0.00 | 0.00 | 28.95 | 43.12 | 64.67 | 100.89 | 187.25 | 306.22 | 418.82 | 418.41 | 389.63 | 349.37 | 228.52 | 180.01 |
| Burkina Faso | 0.00 | 0.00 | 0.00 | 3.09 | 4.88 | 8.14 | 14.63 | 26.12 | 38.33 | 46.31 | 50.57 | 65.22 | 75.11 | 80.02 | 6.00 |
| Burundi | 0.00 | 0.00 | 0.00 | 0.29 | 0.50 | 0.78 | 1.26 | 2.08 | 3.26 | 4.75 | 6.01 | 6.18 | 6.53 | 6.44 | 0.50 |
| Cabo Verde | 0.00 | 0.00 | 0.00 | 0.02 | 0.02 | 0.03 | 0.05 | 0.09 | 0.13 | 0.18 | 0.22 | 0.24 | 0.27 | 0.40 | 0.05 |
| Cambodia | 0.00 | 0.00 | 0.00 | 1.92 | 3.07 | 4.84 | 7.99 | 12.52 | 18.55 | 25.38 | 31.55 | 36.39 | 39.21 | 39.48 | 4.96 |
| Cameroon | 0.00 | 0.00 | 0.00 | 0.20 | 0.31 | 0.51 | 0.85 | 1.37 | 2.09 | 2.90 | 3.51 | 4.16 | 3.95 | 3.91 | 0.32 |
| Canada | 0.00 | 0.00 | 0.00 | 3.53 | 6.29 | 11.47 | 21.35 | 35.98 | 53.48 | 72.66 | 91.89 | 109.78 | 118.37 | 117.79 | 33.50 |
| Central African Republic | 0.00 | 0.00 | 0.00 | 0.05 | 0.08 | 0.13 | 0.24 | 0.40 | 0.57 | 0.75 | 0.81 | 0.83 | 0.80 | 0.67 | 0.09 |
| Chad | 0.00 | 0.00 | 0.00 | 2.30 | 3.85 | 6.27 | 11.11 | 18.39 | 27.47 | 36.81 | 48.32 | 52.99 | 52.69 | 53.02 | 3.86 |
| Chile | 0.00 | 0.00 | 0.00 | 76.94 | 162.43 | 344.89 | 878.85 | 1944.58 | 3588.76 | 5343.66 | 7238.27 | 9927.58 | 12160.16 | 15418.46 | 420.40 |
| China | 0.00 | 0.00 | 0.00 | 3121.35 | 3466.52 | 4596.05 | 7908.06 | 15201.73 | 17967.79 | 19231.49 | 18734.28 | 13508.18 | 11230.60 | 6684.53 | 7924.84 |
| Colombia | 0.00 | 0.00 | 0.00 | 0.46 | 0.76 | 1.17 | 2.04 | 3.64 | 5.64 | 7.58 | 9.44 | 10.66 | 11.81 | 14.26 | 1.89 |
| Comoros | 0.00 | 0.00 | 0.00 | 1.20 | 2.41 | 5.15 | 10.47 | 18.31 | 28.42 | 40.42 | 47.95 | 76.23 | 73.92 | 85.38 | 3.67 |
| Congo | 0.00 | 0.00 | 0.00 | 0.12 | 0.22 | 0.41 | 0.75 | 1.14 | 1.59 | 2.15 | 2.71 | 3.36 | 3.46 | 3.37 | 0.25 |
| Cook Islands | 0.00 | 0.00 | 0.00 | 0.00 | 0.00 | 0.00 | 0.00 | 0.00 | 0.00 | 0.00 | 0.00 | 0.00 | 0.00 | 0.00 | 0.00 |
| Costa Rica | 0.00 | 0.00 | 0.00 | 0.28 | 0.42 | 0.69 | 1.11 | 1.80 | 2.71 | 3.72 | 4.54 | 5.12 | 5.20 | 4.86 | 1.03 |
| Croatia | 0.00 | 0.00 | 0.00 | 14.39 | 23.74 | 32.57 | 50.24 | 110.01 | 179.65 | 247.80 | 240.68 | 179.65 | 208.11 | 159.75 | 97.58 |
| Cuba | 0.00 | 0.00 | 0.00 | 66.02 | 101.04 | 206.50 | 560.50 | 1167.29 | 1862.98 | 2323.18 | 3558.21 | 5043.83 | 6227.21 | 6213.41 | 476.04 |
| Cyprus | 0.00 | 0.00 | 0.00 | 9.12 | 19.36 | 36.94 | 71.14 | 137.46 | 241.98 | 403.42 | 596.71 | 812.98 | 946.96 | 1031.29 | 45.33 |
| Czechia | 0.00 | 0.00 | 0.00 | 56.56 | 88.05 | 154.90 | 243.83 | 379.35 | 541.27 | 734.80 | 822.28 | 847.75 | 768.85 | 439.14 | 350.53 |
| C么te d'Ivoire | 0.00 | 0.00 | 0.00 | 3.39 | 5.73 | 9.38 | 15.76 | 24.40 | 34.97 | 46.38 | 54.28 | 55.84 | 58.59 | 58.48 | 5.10 |
| Democratic People's Republic of Korea | 0.00 | 0.00 | 0.00 | 41.18 | 55.26 | 70.94 | 130.10 | 211.39 | 219.60 | 214.71 | 160.17 | 181.72 | 189.89 | 90.17 | 103.51 |
| Democratic Republic of the Congo | 0.00 | 0.00 | 0.00 | 6.69 | 11.62 | 17.66 | 33.72 | 55.79 | 83.38 | 119.31 | 138.97 | 186.94 | 175.36 | 237.35 | 11.92 |
| Denmark | 0.00 | 0.00 | 0.00 | 91.38 | 191.68 | 480.35 | 1157.85 | 2656.57 | 4179.79 | 6340.23 | 9666.31 | 13998.99 | 13538.14 | 14359.30 | 977.89 |
| Djibouti | 0.00 | 0.00 | 0.00 | 1.97 | 3.46 | 5.75 | 9.34 | 13.90 | 18.65 | 25.33 | 29.24 | 38.25 | 30.03 | 21.58 | 4.19 |
| Dominica | 0.00 | 0.00 | 0.00 | 0.00 | 0.00 | 0.00 | 0.00 | 0.01 | 0.01 | 0.02 | 0.02 | 0.03 | 0.04 | 0.06 | 0.00 |
| Dominican Republic | 0.00 | 0.00 | 0.00 | 435.61 | 724.97 | 1237.86 | 1733.76 | 2302.85 | 3227.23 | 4201.44 | 5359.42 | 6494.81 | 5724.72 | 5284.58 | 1482.86 |
| Ecuador | 0.00 | 0.00 | 0.00 | 4.05 | 6.84 | 11.53 | 21.25 | 35.91 | 55.56 | 72.78 | 90.38 | 107.64 | 121.45 | 143.16 | 14.57 |
| Egypt | 0.00 | 0.00 | 0.00 | 280.81 | 530.96 | 1034.29 | 1961.94 | 3596.00 | 5674.08 | 8214.46 | 12754.86 | 13676.20 | 10696.38 | 6859.41 | 1272.13 |
| El Salvador | 0.00 | 0.00 | 0.00 | 2.66 | 4.42 | 7.94 | 13.14 | 18.31 | 24.95 | 34.65 | 46.87 | 59.07 | 70.34 | 72.57 | 11.31 |
| Equatorial Guinea | 0.00 | 0.00 | 0.00 | 0.86 | 1.26 | 1.82 | 3.00 | 4.70 | 6.98 | 9.88 | 14.98 | 16.62 | 22.89 | 27.88 | 1.23 |
| Eritrea | 0.00 | 0.00 | 0.00 | 0.04 | 0.07 | 0.11 | 0.20 | 0.32 | 0.48 | 0.61 | 0.68 | 0.66 | 0.74 | 0.59 | 0.06 |
| Estonia | 0.00 | 0.00 | 0.00 | 11.21 | 15.16 | 22.43 | 38.43 | 68.52 | 107.72 | 124.18 | 125.88 | 116.02 | 135.53 | 100.31 | 61.26 |
| Eswatini | 0.00 | 0.00 | 0.00 | 1.34 | 2.02 | 3.59 | 6.66 | 11.47 | 16.34 | 23.30 | 30.93 | 38.88 | 41.37 | 31.30 | 3.76 |
| Ethiopia | 0.00 | 0.00 | 0.00 | 22.28 | 36.81 | 65.70 | 110.94 | 161.15 | 221.14 | 307.25 | 426.10 | 616.38 | 734.32 | 729.16 | 37.56 |
| Fiji | 0.00 | 0.00 | 0.00 | 12.72 | 22.77 | 38.56 | 71.57 | 145.33 | 245.27 | 342.04 | 417.92 | 487.89 | 430.59 | 294.73 | 40.84 |
| Finland | 0.00 | 0.00 | 0.00 | 37.63 | 72.03 | 142.55 | 277.14 | 629.66 | 1203.68 | 2208.54 | 3743.56 | 6051.43 | 6499.32 | 10853.30 | 469.58 |
| France | 0.00 | 0.00 | 0.00 | 229.00 | 539.44 | 1269.76 | 3285.56 | 6683.28 | 11595.34 | 18593.23 | 29934.48 | 44148.73 | 53828.86 | 112192.96 | 2348.24 |
| Gabon | 0.00 | 0.00 | 0.00 | 0.25 | 0.34 | 0.55 | 0.95 | 1.40 | 1.86 | 2.17 | 2.34 | 2.10 | 2.62 | 2.78 | 0.68 |
| Gambia | 0.00 | 0.00 | 0.00 | 0.18 | 0.30 | 0.52 | 0.88 | 1.39 | 1.98 | 2.83 | 3.71 | 4.45 | 5.92 | 6.37 | 0.34 |
| Georgia | 0.00 | 0.00 | 0.00 | 17.21 | 29.84 | 54.14 | 115.92 | 259.74 | 583.30 | 1035.92 | 1505.07 | 1680.57 | 2482.59 | 6193.94 | 116.23 |
| Germany | 0.00 | 0.00 | 0.00 | 1765.11 | 3827.00 | 8916.52 | 22663.24 | 59524.00 | 116849.85 | 180652.13 | 266135.35 | 342558.55 | 642795.36 | 1039345.85 | 21591.54 |
| Ghana | 0.00 | 0.00 | 0.00 | 117.21 | 200.37 | 368.85 | 608.41 | 831.60 | 1103.01 | 1495.82 | 2111.99 | 2691.26 | 2388.21 | 1135.23 | 316.58 |
| Greece | 0.00 | 0.00 | 0.00 | 28.04 | 72.07 | 181.77 | 442.79 | 1003.87 | 2133.17 | 4302.37 | 7513.14 | 12958.81 | 18703.72 | 45820.00 | 450.91 |
| Greenland | 0.00 | 0.00 | 0.00 | 1.10 | 2.06 | 3.30 | 6.82 | 20.03 | 30.27 | 39.37 | 47.33 | 56.09 | 55.47 | 44.08 | 6.34 |
| Grenada | 0.00 | 0.00 | 0.00 | 0.02 | 0.03 | 0.06 | 0.14 | 0.26 | 0.39 | 0.50 | 0.58 | 0.70 | 0.80 | 0.51 | 0.10 |
| Guam | 0.00 | 0.00 | 0.00 | 5.20 | 8.37 | 15.08 | 31.07 | 55.76 | 93.24 | 131.10 | 157.69 | 176.46 | 140.96 | 161.51 | 22.85 |
| Guatemala | 0.00 | 0.00 | 0.00 | 6.79 | 10.52 | 14.45 | 20.76 | 30.01 | 44.56 | 59.92 | 71.21 | 77.89 | 80.43 | 70.89 | 14.51 |
| Guinea | 0.00 | 0.00 | 0.00 | 2.07 | 3.38 | 5.35 | 9.20 | 15.23 | 23.72 | 35.36 | 43.88 | 49.82 | 54.18 | 65.17 | 3.14 |
| Guinea-Bissau | 0.00 | 0.00 | 0.00 | 0.16 | 0.24 | 0.38 | 0.66 | 1.08 | 1.62 | 2.28 | 2.97 | 3.55 | 3.69 | 3.37 | 0.26 |
| Guyana | 0.00 | 0.00 | 0.00 | 17.34 | 32.09 | 50.24 | 72.08 | 102.21 | 160.02 | 239.23 | 295.95 | 254.00 | 241.21 | 318.40 | 62.20 |
| Haiti | 0.00 | 0.00 | 0.00 | 38.96 | 58.99 | 76.95 | 112.16 | 160.74 | 222.18 | 307.85 | 394.39 | 482.76 | 505.94 | 461.61 | 73.53 |
| Honduras | 0.00 | 0.00 | 0.00 | 1.18 | 1.92 | 3.33 | 5.99 | 9.52 | 13.77 | 18.26 | 22.49 | 26.18 | 26.24 | 20.03 | 2.95 |
| Hungary | 0.00 | 0.00 | 0.00 | 47.84 | 79.83 | 150.97 | 205.43 | 320.30 | 452.48 | 734.42 | 752.82 | 676.92 | 647.16 | 448.56 | 319.06 |
| Iceland | 0.00 | 0.00 | 0.00 | 0.71 | 1.52 | 3.02 | 6.55 | 13.45 | 24.80 | 40.15 | 60.14 | 90.31 | 117.40 | 261.33 | 5.40 |
| India | 0.00 | 0.00 | 0.00 | 183.83 | 348.29 | 537.61 | 566.71 | 967.21 | 2325.03 | 4534.62 | 4659.23 | 4686.04 | 4851.67 | 5297.29 | 268.12 |
| Indonesia | 0.00 | 0.00 | 0.00 | 62.14 | 119.01 | 165.48 | 175.24 | 224.12 | 309.01 | 477.61 | 463.55 | 398.98 | 354.77 | 247.02 | 163.57 |
| Iran (Islamic Republic of) | 0.00 | 0.00 | 0.00 | 160.24 | 155.29 | 239.41 | 448.95 | 769.35 | 1132.03 | 1862.65 | 3041.42 | 5391.03 | 7084.60 | 11683.94 | 469.54 |
| Iraq | 0.00 | 0.00 | 0.00 | 0.97 | 1.39 | 2.64 | 4.94 | 6.47 | 8.16 | 10.67 | 13.21 | 16.64 | 17.99 | 22.15 | 2.16 |
| Ireland | 0.00 | 0.00 | 0.00 | 39.32 | 100.77 | 221.06 | 497.63 | 1004.51 | 1744.42 | 2764.89 | 4459.83 | 5676.71 | 5854.54 | 6299.84 | 355.74 |
| Israel | 0.00 | 0.00 | 0.00 | 15.37 | 29.51 | 61.30 | 126.53 | 232.47 | 448.61 | 882.21 | 1552.67 | 2163.36 | 2057.05 | 4159.42 | 130.19 |
| Italy | 0.00 | 0.00 | 0.00 | 165.66 | 386.42 | 989.84 | 2428.99 | 5314.99 | 8674.16 | 13253.87 | 21737.30 | 33480.31 | 46427.24 | 79265.05 | 1956.91 |
| Jamaica | 0.00 | 0.00 | 0.00 | 0.56 | 0.95 | 1.69 | 3.73 | 7.08 | 11.46 | 16.23 | 20.03 | 28.34 | 32.11 | 57.36 | 2.52 |
| Japan | 0.00 | 0.00 | 0.00 | 79.49 | 129.09 | 227.40 | 421.99 | 712.90 | 1040.66 | 1369.30 | 1680.35 | 2090.38 | 2364.79 | 2318.27 | 832.85 |
| Jordan | 0.00 | 0.00 | 0.00 | 0.25 | 0.41 | 0.80 | 1.47 | 2.23 | 2.92 | 3.09 | 3.73 | 4.86 | 5.10 | 2.98 | 0.84 |
| Kazakhstan | 0.00 | 0.00 | 0.00 | 1640.19 | 2320.34 | 3448.55 | 5810.74 | 8749.45 | 15502.57 | 22179.80 | 28162.70 | 24864.57 | 30969.29 | 31111.89 | 4641.95 |
| Kenya | 0.00 | 0.00 | 0.00 | 98.53 | 152.64 | 247.31 | 436.05 | 733.18 | 1161.78 | 1643.48 | 2087.02 | 2435.05 | 2855.04 | 3259.48 | 226.87 |
| Kiribati | 0.00 | 0.00 | 0.00 | 0.02 | 0.04 | 0.06 | 0.10 | 0.19 | 0.30 | 0.40 | 0.39 | 0.38 | 0.38 | 0.25 | 0.04 |
| Kuwait | 0.00 | 0.00 | 0.00 | 0.50 | 1.01 | 1.68 | 2.30 | 3.70 | 4.21 | 4.68 | 4.14 | 5.31 | 4.91 | 7.93 | 0.86 |
| Kyrgyzstan | 0.00 | 0.00 | 0.00 | 0.04 | 0.06 | 0.09 | 0.16 | 0.27 | 0.35 | 0.37 | 0.37 | 0.30 | 0.37 | 0.36 | 0.14 |
| Lao People's Democratic Republic | 0.00 | 0.00 | 0.00 | 10.40 | 13.75 | 21.43 | 33.94 | 45.85 | 53.48 | 64.45 | 69.65 | 70.05 | 81.14 | 65.82 | 24.12 |
| Latvia | 0.00 | 0.00 | 0.00 | 13.38 | 17.91 | 26.65 | 47.08 | 89.92 | 143.99 | 159.64 | 143.24 | 126.17 | 172.26 | 99.35 | 74.87 |
| Lebanon | 0.00 | 0.00 | 0.00 | 1.32 | 1.99 | 3.09 | 5.48 | 8.88 | 15.64 | 23.58 | 36.79 | 46.57 | 55.93 | 86.62 | 4.04 |
| Lesotho | 0.00 | 0.00 | 0.00 | 0.20 | 0.38 | 0.61 | 1.01 | 1.86 | 3.12 | 4.72 | 6.46 | 6.50 | 6.07 | 5.35 | 0.39 |
| Liberia | 0.00 | 0.00 | 0.00 | 0.34 | 0.61 | 1.13 | 2.08 | 2.69 | 3.83 | 4.66 | 5.77 | 6.79 | 7.20 | 8.47 | 0.74 |
| Libya | 0.00 | 0.00 | 0.00 | 2.64 | 4.69 | 8.49 | 16.60 | 26.52 | 44.18 | 64.66 | 101.71 | 112.24 | 155.77 | 128.98 | 11.55 |
| Lithuania | 0.00 | 0.00 | 0.00 | 3.60 | 4.98 | 7.73 | 13.83 | 28.80 | 53.94 | 62.58 | 53.42 | 45.03 | 58.20 | 48.69 | 25.43 |
| Luxembourg | 0.00 | 0.00 | 0.00 | 4.46 | 8.05 | 15.66 | 32.31 | 65.04 | 111.09 | 182.07 | 259.33 | 343.66 | 482.90 | 859.10 | 37.79 |
| Madagascar | 0.00 | 0.00 | 0.00 | 8.09 | 13.87 | 24.34 | 43.06 | 72.26 | 111.07 | 154.79 | 183.94 | 226.77 | 206.94 | 252.61 | 16.26 |
| Malawi | 0.00 | 0.00 | 0.00 | 0.42 | 0.69 | 1.14 | 2.11 | 3.30 | 4.99 | 7.48 | 9.66 | 10.60 | 9.71 | 9.47 | 0.73 |
| Malaysia | 0.00 | 0.00 | 0.00 | 9.88 | 15.74 | 24.73 | 43.46 | 78.80 | 124.61 | 178.11 | 213.47 | 223.11 | 201.05 | 228.45 | 26.11 |
| Maldives | 0.00 | 0.00 | 0.00 | 0.00 | 0.00 | 0.01 | 0.01 | 0.01 | 0.02 | 0.02 | 0.02 | 0.02 | 0.03 | 0.04 | 0.00 |
| Mali | 0.00 | 0.00 | 0.00 | 3.29 | 5.71 | 10.10 | 17.38 | 28.73 | 42.32 | 55.04 | 65.08 | 74.30 | 75.88 | 67.52 | 6.28 |
| Malta | 0.00 | 0.00 | 0.00 | 1.60 | 3.58 | 7.70 | 15.11 | 26.95 | 51.35 | 92.24 | 159.88 | 290.43 | 307.50 | 605.78 | 15.80 |
| Marshall Islands | 0.00 | 0.00 | 0.00 | 0.02 | 0.04 | 0.06 | 0.11 | 0.18 | 0.29 | 0.41 | 0.47 | 0.42 | 0.29 | 0.24 | 0.04 |
| Mauritania | 0.00 | 0.00 | 0.00 | 4.98 | 8.88 | 16.88 | 32.75 | 56.77 | 89.19 | 129.53 | 167.93 | 194.71 | 234.09 | 315.60 | 12.91 |
| Mauritius | 0.00 | 0.00 | 0.00 | 0.04 | 0.07 | 0.11 | 0.16 | 0.28 | 0.45 | 0.62 | 0.84 | 0.86 | 0.82 | 0.75 | 0.23 |
| Mexico | 0.00 | 0.00 | 0.00 | 9.78 | 17.22 | 31.54 | 57.63 | 86.97 | 119.58 | 158.89 | 188.28 | 238.85 | 310.57 | 353.21 | 39.47 |
| Micronesia (Federated States of) | 0.00 | 0.00 | 0.00 | 0.03 | 0.05 | 0.10 | 0.20 | 0.35 | 0.63 | 1.03 | 0.92 | 0.92 | 0.78 | 1.07 | 0.09 |
| Monaco | 0.00 | 0.00 | 0.00 | 0.42 | 0.97 | 2.33 | 5.90 | 14.51 | 26.18 | 38.52 | 57.87 | 103.11 | 112.91 | 242.76 | 7.28 |
| Mongolia | 0.00 | 0.00 | 0.00 | 3.24 | 4.86 | 9.47 | 16.82 | 24.23 | 24.83 | 24.11 | 21.67 | 28.94 | 32.34 | 18.80 | 9.74 |
| Montenegro | 0.00 | 0.00 | 0.00 | 0.67 | 1.07 | 1.50 | 2.26 | 4.31 | 7.35 | 9.69 | 9.02 | 6.39 | 6.17 | 4.43 | 3.61 |
| Morocco | 0.00 | 0.00 | 0.00 | 13.01 | 25.17 | 46.21 | 73.99 | 121.50 | 177.50 | 250.91 | 243.30 | 207.41 | 204.54 | 182.69 | 51.55 |
| Mozambique | 0.00 | 0.00 | 0.00 | 1.03 | 1.77 | 3.02 | 5.52 | 8.83 | 13.07 | 18.61 | 24.55 | 28.05 | 28.13 | 28.54 | 2.23 |
| Myanmar | 0.00 | 0.00 | 0.00 | 2.93 | 4.78 | 7.58 | 12.93 | 20.41 | 28.15 | 38.03 | 50.83 | 61.73 | 60.97 | 63.57 | 9.50 |
| Namibia | 0.00 | 0.00 | 0.00 | 0.40 | 0.70 | 1.25 | 2.40 | 4.31 | 6.93 | 9.92 | 13.49 | 14.59 | 17.17 | 20.05 | 0.95 |
| Nauru | 0.00 | 0.00 | 0.00 | 0.55 | 0.67 | 0.90 | 1.18 | 1.44 | 1.59 | 1.63 | 1.14 | 0.80 | 0.57 | 0.29 | 0.75 |
| Nepal | 0.00 | 0.00 | 0.00 | 0.60 | 0.99 | 1.56 | 2.30 | 3.17 | 4.24 | 5.54 | 6.96 | 7.14 | 6.99 | 6.30 | 1.28 |
| Netherlands | 0.00 | 0.00 | 0.00 | 53.28 | 114.65 | 279.69 | 839.45 | 1875.04 | 3353.66 | 5255.07 | 8322.12 | 13848.42 | 16604.33 | 25714.31 | 655.58 |
| New Zealand | 0.00 | 0.00 | 0.00 | 16.01 | 22.52 | 36.48 | 75.28 | 132.28 | 192.20 | 202.35 | 203.99 | 227.91 | 215.70 | 132.55 | 103.35 |
| Nicaragua | 0.00 | 0.00 | 0.00 | 0.13 | 0.20 | 0.28 | 0.42 | 0.66 | 0.97 | 1.28 | 1.60 | 1.71 | 1.76 | 1.53 | 0.32 |
| Niger | 0.00 | 0.00 | 0.00 | 2.48 | 3.97 | 6.02 | 9.39 | 15.82 | 25.68 | 36.85 | 46.77 | 54.07 | 64.93 | 54.50 | 5.18 |
| Nigeria | 0.00 | 0.00 | 0.00 | 103.67 | 151.50 | 232.83 | 392.55 | 813.93 | 1202.74 | 1608.85 | 1842.03 | 1909.40 | 2549.24 | 2245.84 | 179.94 |
| Niue | 0.00 | 0.00 | 0.00 | 0.00 | 0.00 | 0.00 | 0.00 | 0.00 | 0.00 | 0.00 | 0.00 | 0.00 | 0.00 | 0.00 | 0.00 |
| North Macedonia | 0.00 | 0.00 | 0.00 | 5.29 | 7.51 | 9.76 | 15.05 | 28.10 | 43.83 | 55.38 | 51.52 | 39.95 | 33.52 | 15.39 | 20.88 |
| Northern Mariana Islands | 0.00 | 0.00 | 0.00 | 4.43 | 7.58 | 21.43 | 41.29 | 84.08 | 95.99 | 142.50 | 117.70 | 94.44 | 68.22 | 58.22 | 35.54 |
| Norway | 0.00 | 0.00 | 0.00 | 34.05 | 67.05 | 138.85 | 347.08 | 766.34 | 1310.65 | 2075.47 | 3144.33 | 4981.57 | 5564.46 | 9234.84 | 260.66 |
| Oman | 0.00 | 0.00 | 0.00 | 5.27 | 7.97 | 10.06 | 13.58 | 16.89 | 22.47 | 26.73 | 27.74 | 33.32 | 29.98 | 20.40 | 4.50 |
| Pakistan | 0.00 | 0.00 | 0.00 | 849.27 | 1380.74 | 2327.46 | 3560.84 | 4733.88 | 5942.51 | 6998.30 | 7524.57 | 7763.07 | 7738.72 | 6926.66 | 1561.66 |
| Palau | 0.00 | 0.00 | 0.00 | 0.00 | 0.01 | 0.02 | 0.04 | 0.05 | 0.07 | 0.10 | 0.15 | 0.21 | 0.14 | 0.12 | 0.03 |
| Palestine | 0.00 | 0.00 | 0.00 | 1.19 | 1.63 | 2.49 | 4.16 | 7.55 | 12.08 | 15.66 | 19.52 | 20.52 | 19.45 | 15.03 | 3.07 |
| Panama | 0.00 | 0.00 | 0.00 | 1.35 | 2.37 | 4.23 | 8.20 | 13.90 | 20.76 | 29.08 | 38.42 | 48.02 | 55.18 | 74.86 | 5.63 |
| Papua New Guinea | 0.00 | 0.00 | 0.00 | 22.84 | 32.80 | 51.93 | 83.45 | 108.27 | 132.78 | 172.26 | 200.94 | 244.44 | 229.38 | 245.67 | 43.66 |
| Paraguay | 0.00 | 0.00 | 0.00 | 0.08 | 0.11 | 0.17 | 0.29 | 0.45 | 0.67 | 0.90 | 1.10 | 1.18 | 1.32 | 1.33 | 0.26 |
| Peru | 0.00 | 0.00 | 0.00 | 5.06 | 8.56 | 14.65 | 25.21 | 41.36 | 58.69 | 81.74 | 100.10 | 113.77 | 124.42 | 161.07 | 18.86 |
| Philippines | 0.00 | 0.00 | 0.00 | 8.42 | 13.93 | 25.09 | 52.64 | 95.55 | 145.70 | 200.01 | 234.49 | 247.49 | 263.50 | 329.06 | 21.01 |
| Poland | 0.00 | 0.00 | 0.00 | 23.17 | 38.10 | 52.72 | 71.02 | 124.77 | 241.93 | 423.25 | 401.65 | 292.27 | 225.80 | 261.85 | 132.78 |
| Portugal | 0.00 | 0.00 | 0.00 | 82.99 | 191.70 | 466.09 | 962.20 | 1854.11 | 3434.20 | 6216.15 | 10401.71 | 14364.73 | 18085.14 | 34776.54 | 1090.22 |
| Puerto Rico | 0.00 | 0.00 | 0.00 | 160.72 | 307.40 | 689.56 | 1123.83 | 1238.08 | 2140.19 | 3834.70 | 6823.63 | 10729.22 | 11657.18 | 17208.76 | 1600.17 |
| Qatar | 0.00 | 0.00 | 0.00 | 0.35 | 0.47 | 0.62 | 0.85 | 1.00 | 1.22 | 1.11 | 0.86 | 0.66 | 0.41 | 0.16 | 0.34 |
| Republic of Korea | 0.00 | 0.00 | 0.00 | 260.67 | 476.37 | 719.10 | 1432.85 | 2539.18 | 3743.01 | 4000.30 | 2959.88 | 3128.75 | 3311.71 | 1862.46 | 1640.71 |
| Republic of Moldova | 0.00 | 0.00 | 0.00 | 15.62 | 21.01 | 27.87 | 39.46 | 73.38 | 132.46 | 150.94 | 127.44 | 68.62 | 85.70 | 63.54 | 56.74 |
| Romania | 0.00 | 0.00 | 0.00 | 42.51 | 63.85 | 111.48 | 168.56 | 309.68 | 390.82 | 692.61 | 685.24 | 533.69 | 495.75 | 445.45 | 272.43 |
| Russian Federation | 0.00 | 0.00 | 0.00 | 1203.09 | 1656.77 | 2184.61 | 3219.63 | 5317.91 | 9694.58 | 11342.97 | 9625.26 | 6996.49 | 5934.77 | 5183.98 | 4480.79 |
| Rwanda | 0.00 | 0.00 | 0.00 | 1.26 | 2.13 | 3.17 | 5.16 | 8.87 | 14.80 | 22.48 | 31.41 | 32.95 | 37.85 | 41.49 | 2.41 |
| Saint Kitts and Nevis | 0.00 | 0.00 | 0.00 | 1.20 | 2.06 | 3.37 | 5.40 | 8.52 | 13.36 | 19.75 | 20.82 | 17.25 | 19.71 | 14.76 | 4.63 |
| Saint Lucia | 0.00 | 0.00 | 0.00 | 0.15 | 0.29 | 0.54 | 0.97 | 1.63 | 2.33 | 3.03 | 3.60 | 4.46 | 4.81 | 3.72 | 0.82 |
| Saint Vincent and the Grenadines | 0.00 | 0.00 | 0.00 | 1.54 | 2.46 | 4.17 | 6.91 | 11.39 | 18.94 | 28.47 | 34.76 | 35.57 | 38.54 | 37.56 | 8.18 |
| Samoa | 0.00 | 0.00 | 0.00 | 4.27 | 6.57 | 11.92 | 25.95 | 40.49 | 56.57 | 77.27 | 107.82 | 132.95 | 171.03 | 333.59 | 13.43 |
| San Marino | 0.00 | 0.00 | 0.00 | 0.04 | 0.06 | 0.09 | 0.17 | 0.31 | 0.43 | 0.49 | 0.45 | 0.51 | 0.55 | 0.51 | 0.26 |
| Sao Tome and Principe | 0.00 | 0.00 | 0.00 | 0.03 | 0.06 | 0.10 | 0.17 | 0.27 | 0.39 | 0.51 | 0.58 | 0.60 | 0.73 | 0.88 | 0.05 |
| Saudi Arabia | 0.00 | 0.00 | 0.00 | 16.24 | 36.71 | 79.18 | 146.99 | 217.92 | 292.03 | 410.05 | 512.94 | 476.35 | 474.54 | 656.50 | 50.93 |
| Senegal | 0.00 | 0.00 | 0.00 | 9.07 | 15.12 | 25.07 | 42.07 | 70.27 | 105.64 | 139.04 | 164.66 | 189.78 | 195.86 | 187.34 | 18.98 |
| Serbia | 0.00 | 0.00 | 0.00 | 12.96 | 20.99 | 30.44 | 44.63 | 83.80 | 139.38 | 233.63 | 238.92 | 162.16 | 163.68 | 106.26 | 80.97 |
| Seychelles | 0.00 | 0.00 | 0.00 | 0.02 | 0.03 | 0.07 | 0.14 | 0.29 | 0.50 | 0.63 | 0.61 | 0.69 | 0.90 | 1.15 | 0.06 |
| Sierra Leone | 0.00 | 0.00 | 0.00 | 1.13 | 1.81 | 3.04 | 5.42 | 7.82 | 10.48 | 14.17 | 19.33 | 23.80 | 28.45 | 36.77 | 1.49 |
| Singapore | 0.00 | 0.00 | 0.00 | 24.31 | 49.24 | 59.40 | 87.23 | 145.94 | 194.58 | 206.35 | 179.34 | 160.84 | 137.38 | 87.99 | 93.37 |
| Slovakia | 0.00 | 0.00 | 0.00 | 2.60 | 4.21 | 6.44 | 8.45 | 14.75 | 27.99 | 44.80 | 40.54 | 29.23 | 24.16 | 20.61 | 14.39 |
| Slovenia | 0.00 | 0.00 | 0.00 | 1.51 | 2.52 | 3.68 | 5.40 | 11.03 | 18.28 | 26.15 | 25.05 | 18.37 | 20.58 | 19.63 | 10.36 |
| Solomon Islands | 0.00 | 0.00 | 0.00 | 0.00 | 0.00 | 0.00 | 0.00 | 0.00 | 0.00 | 0.00 | 0.00 | 0.00 | 0.00 | 0.00 | 0.00 |
| Somalia | 0.00 | 0.00 | 0.00 | 28.15 | 41.09 | 62.03 | 81.40 | 67.37 | 85.91 | 127.53 | 154.02 | 157.12 | 145.02 | 79.29 | 50.34 |
| South Africa | 0.00 | 0.00 | 0.00 | 2.59 | 4.35 | 7.54 | 13.83 | 23.39 | 35.36 | 48.14 | 57.32 | 63.82 | 67.22 | 67.40 | 8.78 |
| South Sudan | 0.00 | 0.00 | 0.00 | 10.44 | 15.22 | 20.03 | 27.44 | 41.87 | 48.57 | 43.73 | 31.85 | 27.76 | 23.71 | 14.16 | 20.45 |
| Spain | 0.00 | 0.00 | 0.00 | 1380.22 | 3489.97 | 8286.25 | 17242.84 | 36390.48 | 63929.36 | 102087.82 | 158187.56 | 243472.35 | 318618.08 | 788914.62 | 16735.99 |
| Sri Lanka | 0.00 | 0.00 | 0.00 | 0.05 | 0.09 | 0.17 | 0.33 | 0.62 | 1.10 | 1.73 | 2.47 | 3.01 | 2.96 | 3.19 | 0.18 |
| Sudan | 0.00 | 0.00 | 0.00 | 78.37 | 100.04 | 111.13 | 142.28 | 212.21 | 227.26 | 257.54 | 199.10 | 163.90 | 146.31 | 99.41 | 110.06 |
| Suriname | 0.00 | 0.00 | 0.00 | 14.17 | 21.50 | 34.28 | 62.68 | 106.75 | 149.66 | 182.54 | 222.72 | 252.09 | 274.79 | 270.67 | 59.01 |
| Sweden | 0.00 | 0.00 | 0.00 | 41.53 | 69.37 | 140.05 | 328.36 | 689.63 | 1161.23 | 1868.02 | 3056.63 | 5078.13 | 6125.65 | 7557.24 | 405.00 |
| Switzerland | 0.00 | 0.00 | 0.00 | 31.91 | 63.00 | 124.14 | 276.42 | 618.34 | 1032.41 | 1389.65 | 1916.80 | 3324.38 | 5182.72 | 9848.66 | 266.72 |
| Syrian Arab Republic | 0.00 | 0.00 | 0.00 | 3.03 | 10.47 | 23.88 | 53.48 | 117.36 | 192.64 | 349.13 | 670.70 | 1088.94 | 2062.31 | 4279.97 | 38.10 |
| Taiwan (Province of China) | 0.00 | 0.00 | 0.00 | 60.25 | 149.26 | 301.58 | 571.38 | 1218.12 | 2514.81 | 4136.76 | 6037.59 | 6577.19 | 10476.31 | 27549.60 | 278.50 |
| Tajikistan | 0.00 | 0.00 | 0.00 | 7.21 | 9.57 | 13.48 | 21.86 | 33.83 | 41.08 | 42.53 | 36.54 | 31.23 | 27.15 | 15.40 | 15.48 |
| Thailand | 0.00 | 0.00 | 0.00 | 218.74 | 456.32 | 842.95 | 1688.50 | 3228.86 | 5604.02 | 8415.20 | 10047.09 | 9878.53 | 10590.10 | 14546.38 | 1285.00 |
| Timor-Leste | 0.00 | 0.00 | 0.00 | 0.15 | 0.23 | 0.46 | 0.94 | 1.34 | 1.76 | 3.14 | 4.98 | 6.50 | 6.13 | 5.63 | 0.42 |
| Togo | 0.00 | 0.00 | 0.00 | 0.10 | 0.17 | 0.28 | 0.47 | 0.75 | 1.10 | 1.49 | 2.01 | 2.38 | 2.01 | 1.72 | 0.17 |
| Tokelau | 0.00 | 0.00 | 0.00 | 0.00 | 0.00 | 0.00 | 0.01 | 0.01 | 0.01 | 0.01 | 0.02 | 0.01 | 0.01 | 0.01 | 0.01 |
| Tonga | 0.00 | 0.00 | 0.00 | 3.81 | 6.15 | 10.94 | 22.02 | 38.78 | 56.11 | 77.55 | 105.46 | 157.00 | 269.24 | 513.36 | 15.08 |
| Trinidad and Tobago | 0.00 | 0.00 | 0.00 | 7.49 | 12.91 | 19.59 | 30.40 | 49.93 | 91.00 | 141.70 | 200.74 | 222.64 | 205.73 | 207.69 | 36.26 |
| Tunisia | 0.00 | 0.00 | 0.00 | 0.49 | 0.87 | 1.57 | 3.10 | 7.06 | 13.90 | 23.60 | 35.06 | 48.33 | 70.22 | 91.07 | 3.04 |
| Turkey | 0.00 | 0.00 | 0.00 | 24.93 | 27.31 | 44.85 | 66.34 | 103.32 | 159.57 | 235.22 | 293.78 | 405.50 | 392.63 | 644.39 | 57.70 |
| Turkmenistan | 0.00 | 0.00 | 0.00 | 21.74 | 25.38 | 32.94 | 49.30 | 75.46 | 106.71 | 99.20 | 69.32 | 42.13 | 44.00 | 27.91 | 42.05 |
| Tuvalu | 0.00 | 0.00 | 0.00 | 0.05 | 0.06 | 0.07 | 0.11 | 0.17 | 0.21 | 0.22 | 0.18 | 0.16 | 0.13 | 0.05 | 0.10 |
| Uganda | 0.00 | 0.00 | 0.00 | 671.49 | 904.31 | 1243.11 | 1825.66 | 2535.92 | 3352.81 | 4277.43 | 5928.14 | 7130.00 | 6661.10 | 5338.90 | 1440.01 |
| Ukraine | 0.00 | 0.00 | 0.00 | 252.00 | 351.42 | 434.01 | 660.36 | 1236.44 | 2210.94 | 2662.69 | 2345.13 | 1539.17 | 1739.52 | 1350.48 | 1025.44 |
| United Arab Emirates | 0.00 | 0.00 | 0.00 | 4.64 | 8.66 | 14.04 | 16.93 | 17.42 | 13.14 | 11.80 | 8.82 | 4.83 | 3.24 | 1.74 | 6.02 |
| United Kingdom | 0.00 | 0.00 | 0.00 | 180.00 | 370.34 | 636.56 | 1476.69 | 3419.43 | 7141.79 | 12266.58 | 21571.75 | 49406.90 | 94023.17 | 377690.18 | 1003.11 |
| United Republic of Tanzania | 0.00 | 0.00 | 0.00 | 5.24 | 8.87 | 15.73 | 27.69 | 47.66 | 70.83 | 93.29 | 116.70 | 133.09 | 166.25 | 186.66 | 9.63 |
| United States of America | 0.00 | 0.00 | 0.00 | 5.50 | 10.15 | 18.94 | 35.94 | 61.61 | 93.57 | 128.51 | 157.64 | 178.22 | 187.82 | 187.40 | 51.43 |
| United States Virgin Islands | 0.00 | 0.00 | 0.00 | 0.20 | 0.31 | 0.45 | 0.81 | 1.59 | 2.19 | 2.57 | 2.64 | 3.34 | 2.58 | 1.00 | 1.26 |
| Uruguay | 0.00 | 0.00 | 0.00 | 7.35 | 15.34 | 36.40 | 85.59 | 207.09 | 491.60 | 964.00 | 1592.94 | 2413.76 | 3624.54 | 6732.29 | 85.75 |
| Uzbekistan | 0.00 | 0.00 | 0.00 | 137.66 | 163.38 | 202.55 | 295.96 | 469.31 | 642.46 | 585.18 | 394.77 | 215.89 | 183.66 | 48.51 | 227.44 |
| Vanuatu | 0.00 | 0.00 | 0.00 | 0.00 | 0.00 | 0.00 | 0.00 | 0.00 | 0.01 | 0.01 | 0.01 | 0.01 | 0.01 | 0.01 | 0.00 |
| Venezuela (Bolivarian Republic of) | 0.00 | 0.00 | 0.00 | 17.74 | 30.52 | 50.80 | 80.35 | 130.00 | 197.90 | 283.94 | 346.32 | 348.31 | 328.06 | 311.16 | 66.20 |
| Viet Nam | 0.00 | 0.00 | 0.00 | 3.22 | 5.60 | 9.78 | 17.80 | 29.76 | 44.98 | 57.00 | 62.66 | 63.87 | 73.11 | 88.44 | 11.74 |
| Yemen | 0.00 | 0.00 | 0.00 | 0.60 | 0.91 | 1.17 | 1.62 | 2.07 | 2.65 | 3.46 | 4.21 | 4.54 | 5.01 | 3.16 | 1.12 |
| Zambia | 0.00 | 0.00 | 0.00 | 2.64 | 3.86 | 5.80 | 11.72 | 25.79 | 33.23 | 37.25 | 41.99 | 61.04 | 81.10 | 100.45 | 3.72 |
| Zimbabwe | 0.00 | 0.00 | 0.00 | 15.16 | 26.60 | 49.22 | 90.12 | 121.66 | 163.04 | 234.98 | 286.20 | 317.99 | 340.60 | 347.32 | 38.71 |
